# Supplementary material for: The effect of training doctors in communication skills on women’s satisfaction with doctor–woman relationship during labour and delivery: a stepped wedge cluster randomised trial in Damascus
Source: BMJ Open. 2013 Aug 14;3(8):e002674. doi: 10.1136/bmjopen-2013-002674 (PMC3752047; doi:10.1136/bmjopen-2013-002674)
Supplement: Author's manuscript [file bmjopen-2013-002674.draft_revisions.pdf]

Women's satisfaction with doctors ' interpersonal and communication skills at labour and delivery: a Stepped Wedge Cluster Randomized Trial in Damascus

Journal:	*BMJ Open*
Manuscript ID:	bmjopen-2013-002674
Article Type:	Research
Date Submitted by the Author:	05-Feb-2013

Complete List of Authors:	Bashour, Hyam; Faculty of Medicine, Damascus university ,<br>Kanaan, Mona; University of York, Department of Health Sciences<br>Kharouf, Mayada; Damascus University, Department of Family and Community Medicine<br>Abdulsalam, Asmaa; Damascus University, Department of Obstetrics and Gynecology<br>Tabbaa, Mohammed; Damascus University, Department of Obstetrics and Gynecology<br>Cheikha, Salah; Damascus University, Department of Obstetrics and Gynecology

**Primary Subject Heading**:	Obstetrics and gynaecology
Secondary Subject Heading:	Public health, Patient-centred medicine
Keywords:	Maternal medicine < OBSTETRICS, EPIDEMIOLOGY, Quality in health care < HEALTH SERVICES ADMINISTRATION & MANAGEMENT

SCHOLARONE™ Manuscripts

For peer review only

Figure 1: A graphical representation of the overall satisfaction question of the women stratified by hospital. Data presented represent proportion in each category of overall satisfaction at the different time periods.

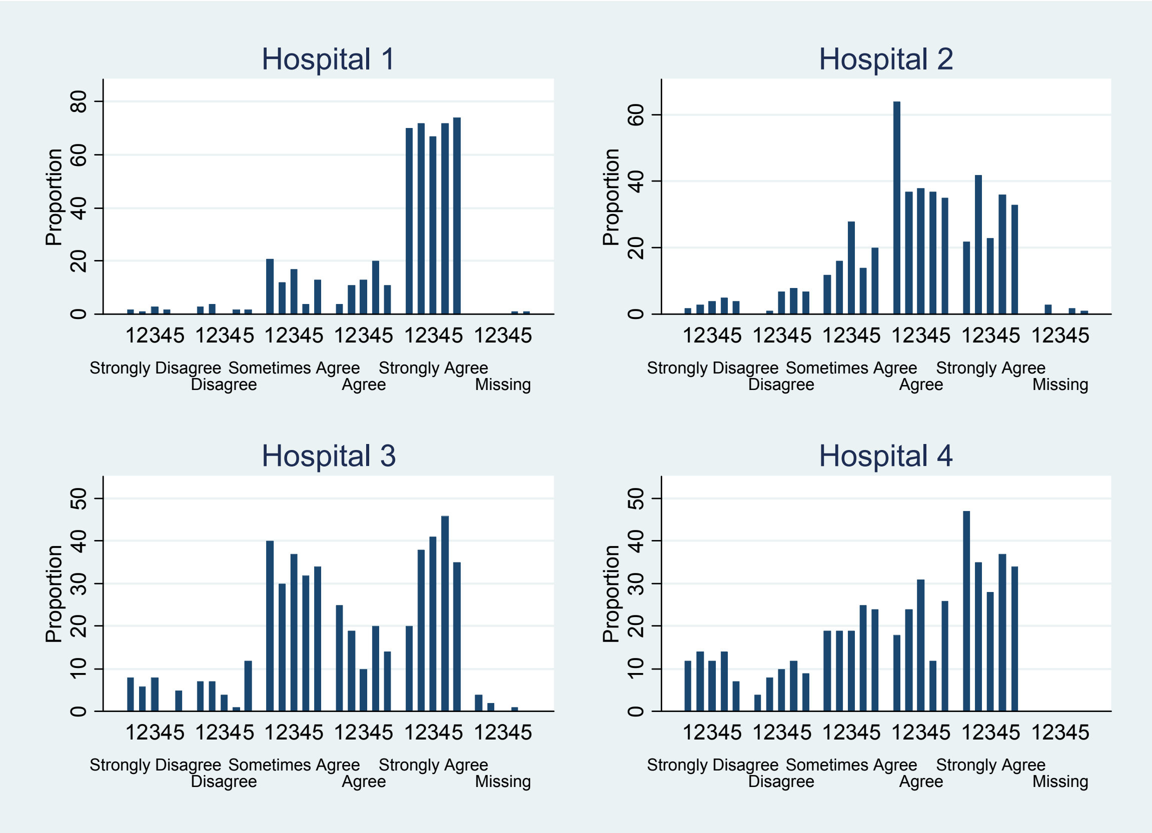

For peer review only - http://bmjopen.bmj.com/site/about/guidelines.xhtml

Additional File One: Schema of the study design*

Hospital	Time				
	1	2	3	4	5
1					
2					
3					
4					

Time 1: Baseline measurement
Time 2: Follow the implementation of intervention at randomly selected hospitals 1
Time 3: Follow the implementation of intervention at randomly selected hospitals 2
Time 4: Follow the implementation of intervention at randomly selected hospitals 3
Time 5: Follow the implementation of intervention at randomly selected hospitals 4

* Shaded area refer to post-intervention

For peer review only

Additional File Two:

A graphical representation of the overall satisfaction outcome score of the women stratified by hospital and time of intervention. Data presented are for complete MMISS only. The rows represent the four hospitals and the columns the five randomisation points. For presentation purposes, the hospitals were coded such that Hospital 1 represented the hospital that had the intervention delivered first which was "time 2" and so forth; "time 1" was the baseline. The title of each graph indicates the hospital and time point presented, for example, a title of 2,3 indicates that this represents the score distribution in Hospital 2 at time 3 (the first intervention period for this hospital).

For peer review only

For peer review only - http://bmjopen.bmj.com/site/about/guidelines.xhtml

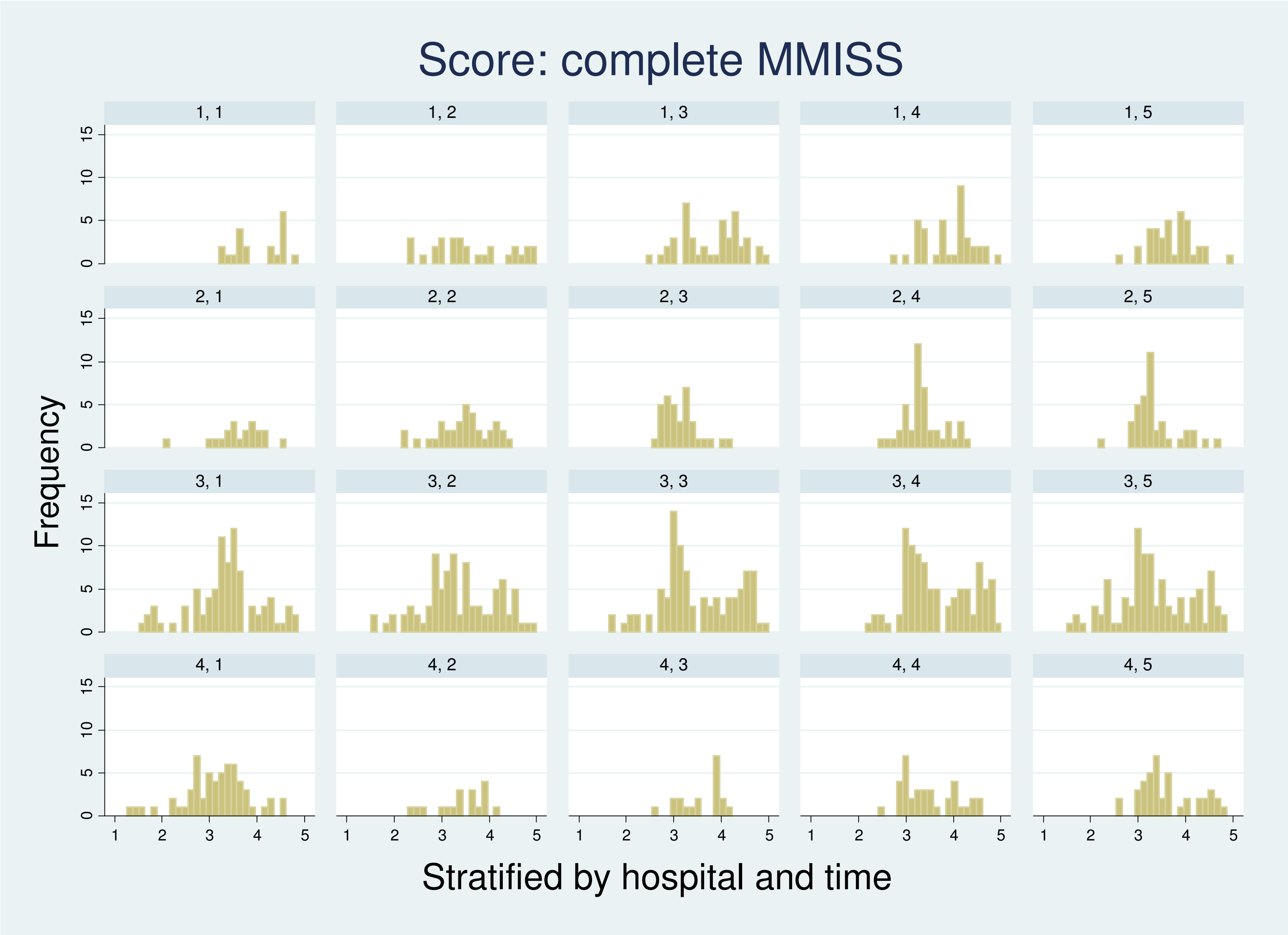
Score: complete MMISS
1, 1
1, 2
1, 3
1, 4
1, 5
2, 1
2, 2
2, 3
2, 4
2, 5
3, 1
3, 2
3, 3
3, 4
3, 5
4, 1
4, 2
4, 3
4, 4
4, 5
Frequency
0
5
10
15
1
2
3
4
5
Stratified by hospital and time

For peer review only

For peer review only - http://bmjopen.bmj.com/site/about/guidelines.xhtml

Women's satisfaction with doctors ' interpersonal and communication skills at labour and delivery: a Stepped Wedge Cluster Randomized Trial in Damascus

Hyam N Bashour[1] *, Mona Kanaan[2], Mayada H Kharouf[1], Asmaa A Abdulsalam [3], Mohammed A Tabbaa[3], Salah A Cheikha[3]

[1] Department of Family and Community Medicine, Faculty of Medicine, Damascus University, Damascus, Syria,

[2] Department of Health Sciences, University of York, York, United Kingdom

[3] Department of Obstetrics and Gynaecology, Faculty of Medicine, Damascus University, Damascus, Syria

* Corresponding author: hbashour@scs-net.org

Details:

HNB: PhD, hbashour@scs-net.org

MK: PhD, mona.kanaan@york.ac.uk

MHK: MSc, mkharouf@scs-net.org

AAA: MD asma@scs-net.org

MT: MD mmtabbaa@hotmail.com

SAC: MD senioreyad@hotmail.com

For peer review only - http://bmjopen.bmj.com/site/about/guidelines.xhtml

Abstract

Objectives: To determine the effect of training residents in interpersonal and communication skills on women's satisfaction with doctor-woman relationship in labour and delivery rooms**.**

Design: a Stepped Wedge Cluster Randomized trial

Setting: Four tertiary-care teaching maternity hospitals in Damascus, Syria

Participants: Two thousands women who gave birth to a living baby in the four study hospitals and consented to participate in the intervention took part in the study. Women with difficult labour and high-risk pregnancies were excluded**.** All were interviewed at home after discharge.

Interventions: A specially designed training package in communication skills that was delivered to all resident doctors at the four hospitals.

Primary and secondary outcome measures: The main outcome measure was women's satisfaction with interpersonal relationships in labour and delivery rooms measured via a series of questions on a Likert scale modified from the Medical Interview Satisfaction Scale. Secondary objectives focused the communicative behaviour of residents serving in labour and delivery rooms using an observation checklist.

Results: At the individual level, the mean for the average satisfaction score was 3.23 (SD: 0.72) out of a possible score of a 5 in the control group and 3.42 (SD: 0.73) in the intervention group. Using generalised linear mixed models, we were not able to detect a difference between the intervention and control group on the overall satisfaction of women; the 95% Confidence Interval associated with the effect of the intervention ranged from 0.08 to 0.15.

Conclusions: In spite of the slight changes in the observed residents' communication skills, the training package in communication skills does not seem to be associated with higher overall satisfaction scores of women**.**

Trial registration Number: ISRCTN80243969

For peer review only - http://bmjopen.bmj.com/site/about/guidelines.xhtml

Funding: Wellcome Trust

Key words: Women's satisfaction, Communication skills, Training, Stepped Wedge Cluster Randomized trial

For peer review only

For peer review only - http://bmjopen.bmj.com/site/about/guidelines.xhtml

Article Summary

'Article focus'

- The study aims to determine the effect of training residents in interpersonal and communication skills on women's satisfaction with doctor-woman relationship in labour and delivery rooms
- The context of the trial focuses on busy teaching maternity hospitals
- This work presents some expertise in a rather rare design of trials

'Key messages'

- In the context of a highly crowded and stressful environment where middle and low class Syrian women deliver, a specially designed training package in interpersonal and communication skills for residents did not achieve an overall improvement in women satisfaction with labour and delivery.
- Despite the lack of evidence from this study, the need to improve the interpersonal skills of medical doctors and obstetricians specifically should be reinforced, as good communication is central to quality health care

For peer review only

For peer review only - http://bmjopen.bmj.com/site/about/guidelines.xhtml

Background

The growing body of literature linking communication skills to a host of valued patient outcomes, including satisfaction, adherence, and positive health indicators, is drawing increasing attention to the centrality of the interpersonal skills of healthcare providers to the delivery of quality medical care. As a result, communication in health-care, particularly between health-care professionals and patients, has attracted an increasing amount of attention at official and professional levels in recent years. Many academic and statutory bodies have all been involved in initiatives to promote good communication including that for maternity care [1-3]. These initiatives are in part a response to increasing evidence from researchers that the quality of the interaction between patients and their care providers may have a significant effect on a variety of aspects of patient well being. These include satisfaction, knowledge and understanding, adherence with advice or treatment, quality of life, and psychological and other health outcomes [4-5].

Maternity care is an area of health-care in which the importance of good communication has received particular attention [3]. Studies of women's views of maternity care suggest that good communication is central in determining whether women are satisfied with the care that they receive or not [6-7]. Observational studies of communication between midwives and women during labour have highlighted the misunderstandings that can occur when communication is poor at this time and identified areas where communication could be improved [8].

Despite the acknowledged importance of communication in maternity care and the official recognition that communication is not always as good as it should be, there have been few evaluations of strategies to improve communication between women and their care providers. Previous reviews of doctor-patient communication have not included any studies carried out in maternity care [4-5]. The review carried out by Rowe in 2002 [9] aimed to fill in that gap and thus identified and reviewed trials of the effectiveness of interventions aimed at improving communication between health professionals and women in maternity care; what we can call it as doctor-woman communication. The review identified trials largely in the area of antenatal care, but also identified a major gap in knowledge relating to communication in a number of key areas of delivery and

For peer review only - http://bmjopen.bmj.com/site/about/guidelines.xhtml

postnatal care; mainly in labour. They recommended that trials of interventions to improve communication between carers and women in labour and in the postnatal period would be particularly useful. All the studies in that review were carried out in developed countries, mainly in the United Kingdom.

In Syria, previous work both in public hospitals and at the community level, showed that continuous support of women during labour and delivery is virtually non-existing. Companionship is not allowed at hospitals, however in a population based study, women reported the existence of a companion at delivery in 37% of the cases [10]. Furthermore Syrian women expressed their dissatisfaction with being left alone and with being attended to by care providers who are not equipped with good communication skills. It was evident that Syrian women taking part in the study were left without any social and emotional support during a critical period in their lives. In most occasions, they were not allowed to be accompanied by their relatives when in labour and delivery. However, they seem to be cared for by doctors and midwives with poor communication skills and little interpersonal competences. There were many instances in which doctors and midwives themselves were sources of dissatisfaction [11].

Therefore, the main objective of this trial was to evaluate the effect of training resident doctors in interpersonal and communication skills on women's satisfaction with doctor-woman relationship in labour and delivery rooms using a modified version of the Medical Interview Satisfaction Scale (MISS-21). The secondary objective of this study was to measure the change in communicative behaviour of the residents after the training in communication skills using an observation checklist.

For peer review only

For peer review only - http://bmjopen.bmj.com/site/about/guidelines.xhtml

Methods

Study design:

A stepped wedge cluster randomized design was used in this trial [12-13]. This particular design as reviewed by Brown and Lilford [13] involves sequential roll-out of the intervention to individuals or clusters over a number of time periods but the order in which participants receive the intervention is determined at random. In this trial the designed training package (the intervention) was implemented in the four study hospitals (clusters) at four time periods; randomly selected, after an initial baseline measurement (See additional File one for a schema of the trial design). This resulted in observing 10 clusters under the control (pre-intervention) periods and another 10 clusters under the intervention periods. The timing of the implementation of the training package in each cluster (hospital) was randomized, however all eligible resident doctors in the study hospitals received the intervention by the end of the study.

Participants:

Participants in this study included care providers and women delivering at four public maternity hospitals in Damascus and its surroundings, Syria. The study hospitals included three hospitals affiliated to the Ministry of Health (MOH) and one university teaching hospital. The hospitals were the Maternity Teaching hospital at Damascus University (15000 deliveries each year), Al-Zahrawi hospital, which is the largest MOH hospital in Damascus (13000 deliveries each year), Douma hospital, which is a MOH district hospital in rural Damascus (7000 deliveries per year), and Harasta hospital, a MOH district hospital in rural Damascus (5000 deliveries per year) [14].

Care providers to undertake the training intervention were all residents registered for the year of field work (2008-2009) in the four hospitals. Midwives were excluded from this study since they had no role in the delivery process, except for assisting doctors, according to policies at those hospitals.

For peer review only - http://bmjopen.bmj.com/site/about/guidelines.xhtml

Women were included if they had normal vaginal birth or by C-section and gave birth to a living baby at the four hospitals during the study period. Women using the study public hospitals come largely from middle and low socio-economic backgrounds.

Informed consents were obtained from both residents and women participating in the study. The study protocol was approved by the Institutional Review Board of Damascus University. All study hospitals approved to take part of this research.

Intervention

The intervention consisted of exposing all residents in the four study hospitals to a training package in interpersonal and communication skills, using a specially designed training package that has been developed by the study team in cooperation with an international expert in the field. The objectives of the training package were as follows: to recognize the impact of effective communication on women and child health during labour and delivery; to identify characteristics and principles of effective communication; to recognize and be able to overcome barriers to effective communication; and to enhance and reinforce the interpersonal communication skills of health providers, and improve their interactions with patients in general and women in labour in particular.

The content of the package included a story from a labour room. Issues regarding attitudes of health providers, overview of doctor-woman communication, non-verbal communication, building rapport, listening skills, effective interviewing, counselling and persuasion, and the ideal maternity ward were all explored. The methodology of the workshop was based on sharing concepts and ideas; self-assessment by means of tests and checklists; brainstorming, group work and plenary discussion; learning by experience; role-play; games and ice breakers. The training package is available for readers upon request.

All residents in the four hospitals received the intervention; their total number came to 137. The training was carried out by a national trainer experienced in communication skills together with members of the research team who observed and facilitated the training. In total, nine training workshops took place at the Faculty of Medicine, Damascus University. The duration of each workshop was 20 hours in total delivered over three

For peer review only - http://bmjopen.bmj.com/site/about/guidelines.xhtml

For peer review only

days. Daily attendance and evaluation as well as overall evaluation of the training workshop by the trainees were very positive.

Data Collection

Data on women's satisfaction was collected prior to the intervention and at each instance of the four randomisation points from all hospitals. Participating women were interviewed at their homes within two weeks after delivery and data were collected on demographic information (age, education, work status, husband's age, husband's education, husband's occupation and home ownership), information about the pregnancy and delivery (whether the mother is multiparous/nulliparous, gender of the newborn, type of delivery), and a modified version of the Medical Interview Satisfaction Scale (MISS) [15].

Furthermore, at each randomisation point data were collected to describe the communicative behaviour of care-providers. Trained observers were asked to fill in a checklist recording observations of communicative behaviour of residents at the labour and delivery rooms level rather than individual residents throughout the different hospital shifts. Three shifts were observed per day which amounted to a total of 24 hours observation of each delivery room at each point of time. We have chosen this approach as we were more interested in the change of the behaviour at the service level rather than the actual change in the behaviour of an individual resident. Furthermore, this approach was expected to reduce problems that could arise if residents were observed individually. Thus, scores from observation were collected at the level of a round rather than scores for each resident. Pre- and post-training measurements were carried out at all four hospitals in each point of time, thus the observation was carried out on an average of two to three weeks after the implementation of the training package in each hospital, with some variance between large and small hospitals. The training took longer time in the large hospitals because of the nature of the workshop and the necessity to implement it two to three times to cover all residents. Observers and field workers were blinded to the specific objectives of the study. Qualitative data collection through six focus group discussions was also carried out prior to the field work and through the design phase of this project. The qualitative methods aimed to understand the work

For peer review only

environment of the residents and get their views concerning the importance of designing a training package in communication skills.

Outcomes

The primary outcome of this study was women's satisfaction with interpersonal and communication skills of doctors working in labour and delivery rooms, which could be seen as a patient outcome in the Ong's model [4]. The theoretical model for doctor-patient/woman communication as suggested by Ong and colleagues (1995) is a useful model to conceptualize an interventional research that aims to improve communication. It consists of background variables, actual content of communication as process indicators as well as patient outcomes including satisfaction in the short time. Women's satisfaction was measured using a Likert scale questionnaire investigating the communication skills of residents attending her. The questionnaire was based on the patient satisfaction questionnaire MISS-21 [15]; the adapted questionnaire is referred to as the Modified Medical Interview Satisfaction Scale (MMISS). The validation exercise of our measurement tool which was implemented in Arabic language is to be reported elsewhere. The questionnaire had a total of 21 questions looking at the communication skills of the doctors in this setup. Women were asked to indicate their level of satisfaction with communicative practices of the attending resident on a 5-point Likert scale with 1 indicating strongly disagree to 5 indicating strongly agree. There were 8 negatively worded questions; the codes of these questions were reversed for analyses purposes. A score was then calculated by summing up the women's answers to the MMISS questions except for the first one, which was a general satisfaction question. Therefore, the highest possible score was a 100 and the lowest 20 where higher values indicate that women were satisfied with the services provided to them. An average score was then calculated by dividing the score by the number of questions [16]. Two scenarios were investigated. In the first scenario, the average score was computed for participants who provided information for the twenty items on the MMISS questionnaire and is referred to as the average score for complete MMISS. In the second scenario, the average score was computed for items with a response and is referred to as the average score only.

For peer review only - http://bmjopen.bmj.com/site/about/guidelines.xhtml

The secondary outcome measured the communicative behaviour of residents serving in labour and delivery rooms using an observation checklist developed by Al-Galaa study in Egypt. The checklist was tested and validated by Sholkamy and colleagues in 2003 [17]. The checklist could be considered as a process evaluation in this work; it constituted of 31 questions. It included items such as whether the attending doctor greeted the woman, identified himself/herself, explained the procedures of the medical examination he/she was about to undertake and communicated the findings, responded to the woman's questions, gave clear instructions about the different stages of labour, delivery and what is expected from the woman and whether the doctor gave any guidance for the next steps post delivery.

Sample size

Power calculations were carried out as in Hussey and Hughes (2007) [12]. A pilot study of 10 women resulted in a mean score of 3.165 (63.3 points on the satisfaction scale) and a standard deviation of 0.71. Based on a cluster size of a 100 at each step and hospital, a mean of 3.15 (63 points) and a standard deviation of 0.75 points, a difference of 0.2 (4 points) could be detected with 90% power given a significance level of 0.05. Therefore, the total sample size needed for the study was 2000 women (100 women x 4 hospitals x 5 time points) not accounting for non-response rate.

The total number of residents in the four hospitals who received the intervention was 137. They were distributed as follows: 85 in the Maternity Teaching Hospital; 31 in Al-Zahrawi Hospital; 7 in Douma Hospital; and 14 in Harasta Hospital.

Statistical methods

Baseline characteristics of the women were summarised using counts (percentages) for categorical variables, means (standard deviations (SD)) and medians (inter-quartile ranges) for continuous variables. Comparison of the main and secondary outcomes between the control (pre-intervention) and intervention periods of the study was carried out at the individual level. Regression analysis was done using Generalized Linear Mixed Model (GLMM) to determine the size and direction of the difference between the control and intervention periods of the study. Estimates of the difference and 95% confidence intervals were calculated. All analyses were adjusted

For peer review only - http://bmjopen.bmj.com/site/about/guidelines.xhtml

for clustering and time of the intervention. To determine our estimates, we used a variety of statistical techniques that took into account the study design as suggested by Hussey and Hughes in 2007 [12]. Therefore, we reported estimates based on using one of the following estimation methods Linear Mixed Models (LMM) where equal sizes of clusters is a must, Generalised Estimating Equations (GEE) and Generalized Linear Latent and Mixed Models (GLLAMM) to ensure the robustness of the estimates specifically if the assumption of normality for the linear model is not met. In each case, the following model was fitted to the data:

$$y_{ijk} = \mu + C_j + S_k + X_{jk}\theta + e_{ijk} \qquad (1)$$

In model (1). y_{ijk} is the outcome of unit i (for example, woman satisfaction score for the primary outcome) in hospital j (j = 1, …, 4) at time point k (k = 1, …,5), C_j is a random effect for hospital j such that $C_j \sim N(0, \tau^2)$, S_k is a fixed effect corresponding to time point k ($S_1=0$ for identifiability), X_{jk} is an indicator of the intervention mode in hospital j at time point k (1=post-/intervention; 0= pre-intervention/control), θ is the treatment effect, e_{ijk} are random errors distributed as $N(0, \sigma^2)$. Therefore, an estimate of θ that is significantly different from zero would indicate an intervention effect. We used multiple imputations in the statistical models in order to address missing data.

Statistical analyses were carried out using STATA software (College Station, Texas) and the R language (www.r-project.org).

For peer review only

For peer review only - http://bmjopen.bmj.com/site/about/guidelines.xhtml

Results

A total of 2000 women were assessed for their satisfaction with patient-woman relationship. All women approached agreed to participate in the trial.

Table 1 shows the characteristics of the women across the different randomisation's points of time. The mean age of the women was 25 years (SD: 6 years). Approximately 95% were housewives. Three quarters had primary education only, 13% were illiterate whereas 12% had high school or further education qualifications. However, there was a differential in the distribution between hospitals with the percent illiterate ranging from 3.6% to 23%. This reflected the fact that two of the hospitals served an urban population whereas the other two served a rural population. The husband's mean age was 31 years (SD: 6.76 years). The vast majority of husbands were self-employed (41%), followed by being a manual worker (35%), and clerk (18%). The distribution of the husband's education was similar to that of the woman's education. Almost half owned their house whereas the other half lived in shared accommodation. A quarter of the women were nulliparous and only 2 women had a C-section

[Table 1 here]

The percentage of women agreed or strongly agreed with the statement of overall satisfaction with doctors' communication skills during labour/delivery ranged from 51% to 83% between hospitals in the control periods and 58% to 85% between hospitals in the intervention periods. These percentages were higher in the intervention periods for all hospitals except for hospital 2 which decreased from 83% to 67% in the intervention periods. Figure 1 gives a graphical representation of these percentages. For presentation purposes, the hospitals were coded such that hospital 1 represented the hospital that had the intervention delivered first which was time 2 and so forth; time 1 was the baseline. The x-axis represents the different overall satisfaction categories at each time point (1 to 5). Based on this figure, women were generally satisfied with their birthing experience; inspection of this figure does not reveal striking differences between the control and intervention

For peer review only - http://bmjopen.bmj.com/site/about/guidelines.xhtml

periods. Table 2 also details the views of women on specific questions with regard to doctors' communication skills. The majority of women pointed-out that doctors did not identify themselves prior to the medical examination; the percentages were similar between the control and intervention periods. One third of the women in the intervention period agreed/strongly agreed that the doctors' greeted them at the onset of the consultation compared to a fifth in the control periods. Around 40% of the women agreed/strongly agreed that the doctor looked at them when he/she talked to the woman in the control periods compared to 60% in intervention periods. Similar percentages were observed for whether the doctor listened to the woman with concern and without interruption. Almost 40% of the women agreed/strongly agreed that the doctor showed an interest in them in the control periods versus a third in the intervention periods. Forty-two percent of the women in the intervention periods agreed/strongly agreed that the doctor used some terms to calm them down and used his/her hands to assist/reassure them compared with 36% and 32% in the control periods, respectively. A small percentage reported that the doctor insulted them (6% control periods versus 3% intervention periods) or shouted/screamed at them (13% control periods versus 7% intervention periods). Only a quarter of the women agreed/strongly agreed that the doctor explained the examination steps before the clinical exams in the control periods compared to 29% in the intervention periods. A third agreed/strongly agreed that the doctor explained the results from the clinical exam in the control periods compared to a 41% in the intervention periods, however, Hospitals 1 and 2 had a decrease in the percentages who agreed/strongly agreed whereas Hospitals 3 and 4 had an increase. Only a quarter of those who responded to the questions whether the doctors explained alternative choices or helped the woman in making decisions agreed/strongly agreed to these statements.

For peer review only

[Table 2 here]

Table 3 provides summary statistics for the intervention and control periods stratified by hospital for the two outcome scores, i.e. average score for MMISS and average score for complete MMISS. The mean score for the average score was 3.23 (SD: 0.72) and 3.42 (SD: 0.73) in the control and intervention periods, respectively. Furthermore, the mean score for the average score in the complete MMISS was 3.43 (SD: 0.71) in the control

For peer review only - http://bmjopen.bmj.com/site/about/guidelines.xhtml

periods and 3.54 (SD: 0.66) in the intervention periods (See additional file 2 for a graphical representation of the average score for complete MMISS).

[Table 3 here]

Regression estimates for the average score on intervention and time using LMM, GEE and GLLAMM techniques to adjust for the clustering by hospital are given in Table 4. The effect of the intervention was an average increase of 0.03 points; however, there was no evidence for statistical significance (95% Confidence Interval (CI): -0.08, 0.15 using GLAMM). However, the estimates using complete MMISS implied that an average decrease of 0.13 in the intervention arm compared to the control arm (equivalent to 2.6 points on the original scale). As before, the effect was not statistically significant with a 95% CI (-0.29, 0.04) using GLAMM. We also used multiple imputations to account for missing data. The estimates (data not shown) were similar to those reported before.

[Table 4 here]

Table 5 gives the numbers and percentages of when an item on the observational checklist, concerning communication between doctor and woman during labour/delivery, was not observed. The majority of doctors did not identify themselves, explained the steps that of labour and delivery, explained to the woman the stages of labour and her role in it, did not give any instructions about the steps that follow delivery or how to care for her new born. In nine out of the 31 items, the proportion of no was consistently lower in the intervention periods compared to the control periods across the hospitals. The proportion of doctors who called the woman by her name or title increased from 75% to 85% and those who asked for permission to examine the woman in general from 39% to 51% and before the vaginal examination from 38% to 58%. The practice of covering the woman

For peer review only - http://bmjopen.bmj.com/site/about/guidelines.xhtml

during delivery increased in the intervention periods to 81% from 41% though there were variations across hospitals; asking the woman to bend her knees increased to 85% from 61%; relaying information to other doctors in the team increased to 83% from 54%; and congratulated the woman on her delivery increased to 60% from 47% in the control periods. Using a regression approach similar to that described above, scores at the shift level were were compared pre and post intervention adjusting for clustering at the hospital level. This showed no evidence of statistical significance with an estimate for the observational checklist of -0.01 (95% CI: -0.03, 0.02) for the intervention parameter.

[Table 5 here]

Discussion

Maternity care is an area of health care in which the importance of good communication has received particular attention as good communication skills are essential for high quality, effective and safe medical practice [1-3]. This trial was designed to determine the effectiveness of training residents at maternity wards in interpersonal and communication skills on women's satisfaction with doctor-woman relationship in labour and delivery rooms in four teaching hospitals in Damascus. Syrian women are largely delivered by medical graduates with little or no training in communication and interpersonal skills. Results of this trial did not provide evidence that the intervention improve women's satisfaction as measured by a modified version of the Medical Interview Satisfaction Scale. Communicative behaviour of the residents measured by an observational checklist was poor before the intervention and showed a slight improvement though not statistically significant after training on some selected items such as asking the woman's permission for the exam or asking her to bend her knees.

The reasons for those negative findings of our trial could be multifaceted. First, we have no reason to think that the negative findings are due to measurement errors having validated the tool and trained the field workers. However, the large numbers of care providers at maternity wards including midwives, nurses and junior doctors

For peer review only - http://bmjopen.bmj.com/site/about/guidelines.xhtml

and even servants in the wards could play a role on the overall satisfaction of women, given that midwives and nurses were not part of our target group for training as their role is mainly to assist doctors rather than taking a full charge of delivery. Furthermore, the policy implemented at Syrian public hospitals of not administering pain relief could also confound the impact of the intervention on satisfaction as women would definitely prefer it [11]. The training package was tailored to our setting and was informed by previous research findings; it allowed for Ong's useful model to conceptualize an interventional research that aims to improve communication and it ensured that the key tasks in communication are covered using effective teaching methods [4, 18]. However, lack of opportunity to reinforce the training could have played a negative role. Brown and colleagues suggested that communication skills programmes may need to be longer, more intensive, teach a broader range of skills, provide ongoing performance feedback, utilize patient feedback and use a variety of instruments to measure change in communication skills [19]. Furthermore the environment in which residents and doctors work in maternity wards is well characterized by long hours, crowded wards, pressure due to organizational hierarchy at hospital, lack of healthy working relations with nurses and midwives [20]. All those factors could have left our target group of residents under pressure, but more importantly could have left women feeling unsatisfied. A recent article by Berridge et al (2010) highlighted those areas that deeply examine communication within delivery suite, as well [21].

The interpretation of the findings should also consider the women status in a community within which the expectations of the women are very low but also where culture and gender issues are critical. Previous research findings showed that some means of communication (eye to eye communication for example) are not acceptable if gender of the provider is different from that of woman [11]. We also argue that the overall positive satisfaction of women in our observation can be due to the childbirth event by itself as being a pleasing experience as well as to getting the service free of charge in public hospitals by those women who come from a rather poor socio-economic class [11]. It was surprising to see that the very positive evaluation of the training sessions by the trainees did not translate to improved satisfaction of the women. However, we fully

For peer review only - http://bmjopen.bmj.com/site/about/guidelines.xhtml

support Bingham that stressed the importance of carrying out patient satisfaction surveys to help hospitals change or improve their childbirth policy [22].

Strengths and limitations

The key strength of the study was in its stepped-wedge cluster randomised design characterized by being ethical and practical [13], which resulted in the training package being delivered to all residents thus complementing their medical training in communication skills that is missing from their curriculum. Furthermore, the study had a 100% response rate among women but there were some missing values on some of the questions. High response rate is very common in our culture. Conducting the trial in a developing country certainly contributes to the field as compared to the literature that comes mainly from developed countries [1-3, 9].

One of the study limitations is being unable to link women satisfaction with a specific doctors' behaviour of doctor-woman relationship as we observed actions rather than individual residents' behaviour for ethical as well as practical reasons. Furthermore, the selection of public hospitals only in this study might have impacted the generalizability of the findings to other settings; however this setting is very common in our region [23].

Conclusions

In the context of a highly crowded and stressful environment where middle and low class Syrian women deliver, a specially designed training package in interpersonal and communication skills for residents did not achieve an overall improvement in women satisfaction with labour and delivery. However, certain items in doctors' behaviour have improved. It would be worth investigating whether the package would improve women satisfaction in less stressful settings, but also it is worth looking at other possible interventions in maternity care practice such as doctor-midwife collaboration or attendance of birth companion in such setting.

Despite the lack of evidence from this study, the need to improve the interpersonal skills of medical doctors and obstetricians specifically should be reinforced, as good communication is central to quality health care [24].

For peer review only

For peer review only - http://bmjopen.bmj.com/site/about/guidelines.xhtml

Competing Interest

The authors declare no competing interests.

Authors' contributions

HNB and AAA developed the study concept and aims then initiated the project. MKH, MT, SAC and MK assisted further in the refinement of the concept and development of the protocol. HHB, MKH and AAA oversaw collection of data. MK did the statistical analysis. HHB and MK drafted the manuscript. All authors read and approved the final manuscript.

Acknowledgments

Our thanks are due to Mrs Hoda Hallab who worked with the team to develop the training package and deliver the training at kick-off point. We are also grateful to the directors of the hospitals for facilitating the field work.

This paper is part of a larger regional research project on Choices and Challenges in Changing Childbirth in the Arab Region sponsored by the Center for Research on Population and Health at the American University of Beirut, Lebanon, with generous support from the Wellcome Trust (UK) [Grant Ref:074986].

For peer review only

For peer review only - http://bmjopen.bmj.com/site/about/guidelines.xhtml

References

1- Teutsch C. **Patient-doctor communication.** Med Clin North Am 2003; **87**:1115-45.

2- Department of Health. Changing childbirth. Part 1. Report of the Expert Maternity Group. London: HMSO, 1993.

3- Department of Health. Changing childbirth. Part 2: Survey of good communication practice in maternity services. London: HMSO, 1993.

4- Ong LML, de Haes JCJM, Hoos AM, Lammes FB. **Doctor-patient communication: a review of the literature**. Soc Sci Med 1995; **40**: 903-918.

5- Stewart MA. **Effective physician-patient communication and health outcomes: a review**. Can Med Ass J 1995; **152**: 1423-1433.

6- Garcia J, Redshaw M, Fitzsimons B, Keene J. First Class Delivery. A National Survey of Women's Views of Maternity Care. London: Audit Commission, 1998.

7- Kabakian-Khasholian T, Campbell O, Shediac-Rizkallah M, Ghorayeb F. (2000) **Women's experiences with maternity care: satisfaction or passivity?** Soc Sci Med, **51**:103-113.

8- Garcia J, Garforth S. Parents and new-born babies in the labour ward. In: Garcia J, Kilpatrick R, Richards M (eds.) The Politics of Maternity Care. Services for Childbearing Women in Twentieth-Century Britain. Oxford: Oxford University Press, 1990: 163-182.

9- Rowe RE, Garcia J, Macfarlane AJ, et al. **Improving communication between health professional and women in maternity care: a structured review**. Health Expect 2002; **5**: 63-83.

10- Bashour H, Abdulsalam A, Al-Faisal W, Cheikha S. **The patterns and determinants of maternity care in Damascus, Syria**. East Mediterr Health J. 2008; **14**: 398-405.

11- Hafez R, Bashour H, Abdulsalam A. Childbirth and its discontent: Experiences of Syrian Women. A paper presented at the Annaul meeting of Reproductive Health Working Group (RHWG) Annual Meeting, Lebanon, July 11-13, 2004.

For peer review only - http://bmjopen.bmj.com/site/about/guidelines.xhtml

12- Hussey A. and Hughes J.P. **Design and Analysis of stepped wedge cluster randomized trials**. Contemp Clin Trials 2007; **28**:182-191.

13- Brown CA and Lilford RJ. **The stepped wedge trial design: a systematic review**. BMC Med Res Methodol 2006, **6**:54.

14- Syrian Ministry of Health. www.moh.gov.sy

15- Meakin R and Weinman J. **The Medical Interview Satisfaction Scale (MISS-21) adapted for British general practice**. Fam Pract 2002; **19**:257-263.

16- Holloway S, Porteous M, Cetnarskyj R, Anderson E, Rush R, Fry A, Gorman D, Steel M, Campbell H. 2004. **Patient satisfaction with two different models of cancer genetic services in south-east Scotland**. *Br J Cancer* **90**: 582–589.

17- Sholkamy H, Khalil K, Cherine M, Elnoury A, Breebaart M, Hassanein N. An observation checklist for facility-based normal labour and delivery practices: The Galaa Study. Monographs in Reproductive Health No. 5. Cairo; The Population Council, 2003.

18- Maguire P and Pitceathly C**. Key communication skills and how to acquire them**. BMJ 2002; **325**:697-700.

19- Brown JB, Boles M, Mullooly JP, Levinson W. **Effect of clinician communication skills training on patient satisfaction. A randomized, controlled trial**. Ann Intern Med 1999; **131**:822–9.

20- Abdulasalam A. **Communication Skills! What does it mean to doctors and women: A qualitative study in Damascus maternity teaching hospitals**. Damascus University Journal for Health Sciences 2009; **25**:189-203. (In Arabic)

21- Berridge E, Mackintosh N and Freeth D. **Supporting patient safety: examining communication within delivery suite teams through contrasting approaches to research observation**. Midwifery 2010; **26**: 512-519.

22- Bingham D. **Helping hospitals change. Part 2: Childbirth education techniques to empower women**. J Perinatal Edu 2005; **14**: 51-55.

For peer review only - http://bmjopen.bmj.com/site/about/guidelines.xhtml

23- Choices and Challenges in Changing Childbirth Research Network. **Routines in facility-based maternity care: evidence from the Arab World**. BJOG: an International Journal of Obstetrics and Gynaecology 2005; **112**, 1270-1276.

24- Audit Commission. What seems to be the matter: Communication between hospitals and patients. HMSO, London. 1993.

For peer review only

For peer review only - http://bmjopen.bmj.com/site/about/guidelines.xhtml

Figure Legends:

Tables:

For peer review only

For peer review only - http://bmjopen.bmj.com/site/about/guidelines.xhtml

Table 1: Demographic Characteristics of the women across the time periods of the study'

	1	2	3	4	5
N	400	400	400	400	400
Mean age (SD)	24.79 (5.71)	25.12 (6.11)	25.07 (6.4)	24.97 (5.74)	25.37(6.04)
Median age (Q1, Q3)*	24 (20, 28)	24 (20, 29)	24 (20, 29.5)	24 (20, 29)	24 (20, 29)
Occupation: Housewife	377 (94)	377 (94)	387 (97)	378 (94)	383 (96)
Education					
Illiterate/Read and Write	53 (13)	51 (13)	43 (11)	53 (13)	42 (11)
Primary	291 (74)	306 (77)	304 (76)	305 (77)	300 (76)
High School +	51 (13)	42 (11)	53 (13)	38 (10)	54 (14)
Mean husband's age (SD)	30.98 (6.89)	31.19 (7.18)	31 (6.82)	30.16 (6.35)	31.07 (6.53)
Median husband's age (Q1, Q3)	30 (26, 35)	30 (26, 35)	30 (26, 35)	29 (26, 34)	30 (26, 35)
Husband's Occupation					
Labourer (manual worker)	152 (39)	131 (33)	134 (34)	143 (36)	136 (34)
Clerk	72 (18)	71 (18)	64 (16)	80 (20)	71 (18)
(Self-employed)	144 (37)	163 (41)	175 (44)	166 (42)	175 (44)
Other	24 (6)	33 (8)	24 (6)	7 (2)	13 (3)
Husband's Education					
Illiterate/Read and Write	55 (14)	48 (12)	41 (10)	37 (9)	33 (8)
Primary	299 (76)	295 (74)	310 (78)	310 (79)	310 (79)

For peer review only - http://bmjopen.bmj.com/site/about/guidelines.xhtml

High School +	41 (10)	53 (13)	45 (11)	46 (12)	50 (13)
Home Ownership					
Owns home	192 (49)	197 (49)	217 (55)	214 (55)	197 (50)
Share	201 (51)	202 (51)	179 (45)	178 (45)	198 (50)
Parity: Nulliparous	100 (25)	120 (30)	124 (31)	97 (24)	88 (22)
New born gender: Male	215 (56)	193 (49)	179 (45)	198 (52)	202 (52)

* Q1 is the first quartile and Q3 is the third quartile.

! Numbers represent counts (percentage) unless otherwise stated.

For peer review only

Table 2: Numbers (and percentages) of women who agreed/strongly agreed with various questions concerning their labour/delivery experience. The first two columns give the numbers comparing all the intervention clusters to the control clusters; the other columns give the corresponding numbers stratified by hospital.*

	Control Period	Intervention Period	Control Period				Intervention Period			
	All	All	1	2	3	4	1	2	3	4
N	1000	1000	100	200	300	400	400	300	200	100
Overall satisfaction with doctors' communication skills during labour/delivery	619 (62)	713 (72)	74 (74)	163 (83)	150 (51)	232 (58)	338 (85)	200 (67)	115 (58)	60 (60)
Did the doctors identify themselves?	132 (13)	118 (12)	1 (1)	11 (6)	94 (31)	26 (7)	35 (9)	7 (2)	62 (31)	14 (14)
Did the doctor greet you?	208 (21)	323 (32)	36 (36)	45 (23)	86 (29)	41 (10)	183 (46)	55 (19)	63 (32)	22 (22)
Did the doctor look at you when talking to you?	434 (43)	599 (60)	61 (61)	158 (79)	94 (31)	121 (30)	305 (76)	181 (61)	78 (39)	35 (35)
Did the doctor show interest in you as a person?	393 (39)	329 (33)	47 (47)	42 (21)	139 (46)	165 (41)	188 (47)	21 (7)	83 (42)	37 (37)
Did the doctor insult you?	55 (6)	29 (3)	2 (2)	3 (2)	15 (5)	35 (9)	5 (1)	19 (6)	1 (1)	4 (4)
Did the doctor use humour to comfort you?	78 (8)	118 (12)	19 (19)	13 (7)	34 (11)	12 (3)	70 (18)	7 (2)	34 (17)	7 (7)
Did the doctor use any terms to calm you down?	363 (36)	419 (42)	44 (44)	102 (51)	109 (36)	108 (27)	200 (50)	116 (39)	73 (37)	30 (30)

For peer review only - http://bmjopen.bmj.com/site/about/guidelines.xhtml

For peer review only

Did the doctor use his/her hands to assist you or to reassure you?	314 (32)	409 (42)	52 (54)	115 (58)	79 (26)	68 (17)	200 (52)	137 (46)	57 (29)	15 (16)
Did the doctor shout or scream at you?	125 (13)	71 (7)	6 (6)	15 (8)	28 (9)	76 (19)	18 (5)	31 (10)	10 (5)	12 (12)
Did the doctor listen to you with concern and without interrupting you?	413 (46)	551 (58)	57 (62)	108 (65)	98 (33)	150 (44)	313 (79)	116 (45)	73 (37)	49 (50)
Was the doctor engaged in other issues, so you felt being unattended and annoyed?	165 (17)	67 (7)	3 (3)	20 (10)	75 (25)	67 (17)	12 (3)	19 (6)	27 (14)	9 (9)
Did you feel let down by the way he/she was dealing with you?	66 (7)	31 (3)	2 (2)	10 (5)	10 (3)	44 (11)	10 (3)	19 (6)	1 (1)	1 (1)
Did the doctor explain all the steps before doing the clinical exam?	249 (25)	278 (29)	17 (17)	88 (45)	41 (14)	103 (26)	70 (18)	128 (47)	46 (23)	34 (35)
Did the doctor explain the findings from the clinical exam?	326 (34)	399 (41)	42 (68)	82 (42)	61 (20)	141 (36)	181 (46)	101 (37)	63 (32)	54 (56)
Were you annoyed by the doctor explaining all the findings?	40 (5)	73 (10)	4 (8)	18 (13)	3 (1)	15 (5)	29 (11)	39 (18)	2 (1)	3 (3)
Did the doctor explain all the alternative choices to you?	176 (23)	145 (17)	12 (27)	44 (35)	94 (31)	26 (9)	37 (10)	31 (16)	66 (33)	11 (13)
Did the doctor help you in	175	158	12	25	97	41	52	16	75	15

For peer review only

making decisions?	(29)	(23)	(44)	(29)	(33)	(21)	(19)	(9)	(38)	(35)
Was the doctor annoyed from your questions and avoided answering them?	122 (14)	54 (6)	9 (11)	14 (9)	35 (12)	64 (19)	20 (6)	21 (8)	6 (3)	7 (7)
Did the doctor use medical terms in explanation, thus they were not understood?	77 (8)	48 (5)	0 (0)	6 (3)	55 (19)	16 (5)	1 (0)	5 (2)	37 (19)	5 (6)
Did the doctor give you conflicting medical information?	31 (3)	12 (1)	1 (1)	3 (2)	6 (2)	21 (6)	0 (0)	9 (3)	2 (1)	1 (1)

* Due to missing information some of the percentages do not tie up with the total number given.

Table 3: Summary statistics for the outcome of women satisfaction computed as the average score and Average Score for Complete MMISS . The first two columns give the numbers comparing all the intervention clusters to the control clusters; the other columns give the corresponding numbers stratified by hospital

For peer review only

	Control Period	Intervention Period	Control Period				Intervention Period			
	All	All	1	2	3	4	1	2	3	4
Average Score										
N	1000	1000	100	200	300	400	400	300	200	100
Mean (SD)	3.23 (0.72)	3.42 (0.73)	3.57 (0.72)	3.43 (0.59)	3.33 (0.78)	3.06 (0.67)	3.54 (0.73)	3.25 (0.66)	3.48 (0.77)	3.33 (0.71)
Median (Q1, Q3)	3.3 (2.8,3.7)	3.3 (3.0, 4.1)	3.6 (3.1, 4.3)	3.5 (3.1, 3.8)	3.3 (2.9, 3.9)	3.2 (2.6, 3.5)	3.5 (3.0, 4.2)	3.2 (2.9, 3.7)	3.4 (3.0, 4.2)	3.3 (3.0, 3.7)
Average Score for Complete MMISS										
N	472	498	20	55	265	132	148	118	191	41
Mean (SD)	3.43 (0.71)	3.54 (0.66)	4.04 (0.54)	3.52 (0.56)	3.42 (0.77)	3.33 (0.62)	3.77 (0.62)	3.29 (0.43)	3.50 (0.77)	3.62 (0.60)
Median (Q1, Q3)	3.4 (3.0,3.9)	3.3 (3.1, 4.1)	4.1 (3.6,4.6)	3.6 (3.2, 3.9)	3.3 (2.9, 4.1)	3.4 (3.0, 3.8)	3.8 (3.3, 4.2)	3.2 (3.0, 3.4)	3.35 (3.1, 4.2)	3.4 (3.2, 4)

For peer review only

Table 4: Regression analysis estimates for the average score and average score for complete MMISS on intervention and time controlling for clustering by hospital using three estimation procedures LMM, GLAMM and GEE. Estimates are presented together with 95% confidence interval (CI) limits as subscripts (${}_{Lower}Est_{Upper}$).

	Average Score			Average Score Complete MMISS		
	LMM	GLAMM	GEE	LMM	GLAMM	GEE
	${}_{Lower}Est_{Upper}$	${}_{Lower}Est_{Upper}$	${}_{Lower}Est_{Upper}$	${}_{Lower}Est_{Upper}$	${}_{Lower}Est_{Upper}$	${}_{Lower}Est_{Upper}$
**Fixed-effects**						
intervention	${}_{-0.09}0.03_{0.14}$	${}_{-0.08}0.03_{0.15}$	${}_{-0.08}0.03_{0.14}$	${}_{-0.29}-0.13_{0.03}$	${}_{-0.29}-0.13_{0.04}$	${}_{-0.26}-0.10_{0.06}$
time: 2	${}_{-0.08}0.02_{0.13}$	${}_{-0.08}0.02_{0.12}$	${}_{-0.08}0.02_{0.12}$	${}_{-0.10}0.04_{0.19}$	${}_{-0.10}0.05_{0.19}$	${}_{-0.10}0.05_{0.19}$
time: 3	${}_{-0.14}-0.03_{0.09}$	${}_{-0.14}-0.03_{0.08}$	${}_{-0.14}-0.03_{0.08}$	${}_{-0.02}0.12_{0.27}$	${}_{-0.02}0.12_{0.27}$	${}_{-0.03}0.12_{0.27}$
time: 4	${}_{-0.08}0.05_{0.18}$	${}_{-0.08}0.05_{0.18}$	${}_{-0.08}0.05_{0.18}$	${}_{0.13}0.31_{0.49}$	${}_{0.12}0.30_{0.49}$	${}_{0.10}0.28_{0.47}$
time: 5	${}_{-0.14}0.01_{0.16}$	${}_{-0.15}0.01_{0.16}$	${}_{-0.14}0.01_{0.15}$	${}_{-0.02}0.18_{0.38}$	${}_{-0.03}0.17_{0.37}$	${}_{-0.05}0.15_{0.35}$
constant	${}_{3.14}3.32_{3.50}$	${}_{3.16}3.32_{3.48}$	${}_{3.16}3.32_{3.48}$	${}_{3.20}3.84_{3.67}$	${}_{3.23}3.44_{3.64}$	${}_{3.28}3.43_{3.59}$
**Random-effects**						
	SD	Var		SD	Var	
**hospital**	${}_{0.07}0.17_{0.40}$	0. 02 (0.016)		${}_{0.09}0.22_{0.51}$	0.03 (0.026)	
**Residual**	${}_{0.68}0.71_{0.73}$	0.51 (0.016)		${}_{0.64}0.67_{0.70}$	0.45 (0.02)	

For peer review only

Table 5: Numbers (and percentages) of "No" for items on the observational checklist concerning communication between doctor and woman during labour/delivery, unless otherwise stated. Due to missing information some of the percentages do not tie up with the total number given. The first two columns give the numbers comparing all the intervention clusters to the control clusters; the other columns give the corresponding numbers stratified by hospital.

	Control Period	Intervention Period	Control Period				Intervention Period			
Hospital	All	All	1	2	3	4	1	2	3	4
N	565	631	98	194	106	167	326	218	38	49
1. Did the doctor identify himself/herself?	539 (100)	621 (99)	90 (100)	177 (99)	105 (100)	167 (100)	320 (99)	215 (100)	38 (100)	48 (98)
2. Did the doctor call the woman by her name?	132 (24)	99 (16)	22 (24)	48 (27)	22 (21)	40 (24)	52 (16)	30 (14)	6 (16)	11 (22)
3. Did the doctor take the woman's permission to examine her?	326 (61)	305 (49)	53 (59)	113 (64)	61 (58)	99 (60)	183 (57)	77 (36)	18 (47)	27 (55)
4. Did the doctor explain the plan he/she will follow?	497 (92)	577 (92)	81 (90)	168 (95)	98 (93)	150 (90)	296 (92)	203 (94)	35 (92)	43 (88)
Before starting the vaginal examination, did the doctor										
5. take her permission for the exam?	335 (62)	262 (42)	58 (65)	115 (65)	58 (55)	104 (63)	155 (48)	68 (32)	14 (37)	25 (51)
6. close the door?	472 (88)	568 (91)	85 (96)	136 (77)	87 (83)	164 (99)	313 (97)	180 (84)	26 (68)	49 (100)
7. cover the woman during the exam?	315	116	20	97	79	119	32	21	21	42

For peer review only

For peer review only - http://bmjopen.bmj.com/site/about/guidelines.xhtml

	(59)	(18)	(23)	(55)	(75)	(72)	(10)	(10)	(55)	(91)
8. ask the woman to bend her knees?	210 (39)	79 (13)	34 (38)	76 (43)	31 (30)	69 (42)	48 (15)	34 (16)	6 (16)	7 (14)
After completing the vaginal examination, did the doctor										
9. tell the woman the results of the exam?	418 (78)	504 (81)	73 (82)	157 (89)	74 (70)	114 (69)	270 (84)	174 (81)	31 (82)	29 (59)
10. explain the next steps to be followed?	486 (91)	586 (94)	73 (83)	168 (95)	98 (94)	147 (89)	302 (94)	204 (95)	34 (89)	46 (94)
11. relay the findings to other team members?	241 (45)	102 (16)	17 (19)	87 (49)	45 (43)	92 (59)	41 (13)	30 (14)	12 (36)	19 (39)
12. relay the findings to the midwives/nurses?	393 (80)	579 (93)	60 (83)	154 (87)	70 (71)	109 (75)	312 (97)	211 (98)	32 (84)	24 (49)
13. Did the doctor give instructions about eating and drinking?	463 (87)	516 (83)	77 (89)	148 (84)	90 (86)	148 (90)	261 (81)	183 (85)	33 (87)	39 (80)
14. Did the doctor give instructions about movement?	335 (63)	428 (70)	65 (77)	112 (62)	59 (57)	99 (60)	222 (71)	155 (72)	26 (68)	25 (51)
15. Was the woman asked any questions at this stage?	168 (32)	94 (15)	11 (14)	82 (46)	26 (25)	49 (30)	43 (14)	38 (18)	6 (16)	7 (14)
16. Did the woman ask any questions at this stage?	292 (56)	281 (46)	34 (44)	123 (68)	52 (52)	83 (51)	152 (49)	93 (44)	13 (35)	23 (47)
17. Did the doctor respond to her queries?	275 (63)	342 (64)	32 (44)	96 (70)	59 (65)	88 (65)	168 (65)	124 (64)	21 (64)	29 (63)
18. Was the doctor responsive to the woman's pain?	285 (59)	333 (57)	35 (47)	95 (58)	54 (59)	101 (65)	162 (53)	119 (58)	22 (73)	30 (63)
19. Was the woman encouraged at this stage?	186 (40)	239 (42)	17 (25)	59 (38)	33 (36)	77 (52)	115 (40)	85 (42)	16 (53)	23 (48)

For peer review only

For peer review only - http://bmjopen.bmj.com/site/about/guidelines.xhtml

20. Was the woman told about the proximity of labour?	242 (55)	363 (66)	25 (39)	79 (54)	49 (55)	89 (63)	180 (65)	136 (68)	20 (71)	27 (60)
21. Was the woman told about the stages of labour?	399 (95)	533 (98)	49 (83)	133 (96)	85 (97)	132 (96)	269 (98)	197 (99)	28 (100)	39 (98)
22. Was the woman told about her role during labour?	347 (83)	508 (94)	29 (51)	114 (83)	80 (90)	124 (91)	256 (93)	190 (95)	25 (89)	37 (93)
23. During delivery, was the woman asked to push?	26 (6)	23 (4)	6 (11)	6 (4)	3 (3)	11 (8)	13 (5)	7 (4)	3 (11)	0 (0)
24. Did they explain to the woman when and how to push?	237 (57)	230 (42)	30 (54)	76 (55)	48 (55)	83 (61)	114 (41)	85 (43)	15 (54)	16 (40)
25. Did the woman ask any questions at this stage?	284 (68)	296 (55)	35 (63)	100 (73)	59 (67)	90 (66)	166 (61)	95 (48)	15 (54)	20 (50)
26. Did the doctor respond to her queries?	264 (77)	330 (72)	36 (68)	77 (77)	66 (81)	85 (78)	160 (71)	127 (73)	17 (81)	26 (68)
27. Did the doctor inform the mother about her baby's status?	307 (75)	386 (72)	26 (49)	92 (68)	72 (83)	117 (86)	171 (63)	161 (82)	25 (89)	29 (74)
28. Was the woman congratulated after the delivery?	217 (53)	214 (40)	26 (47)	63 (46)	49 (58)	79 (58)	102 (37)	81 (41)	10 (36)	21 (53)
29. Was the woman instructed about the process after the delivery?	392 (95)	515 (96)	51 (93)	127 (93)	82 (95)	132 (97)	267 (97)	182 (92)	27 (96)	39 (98)
30. Was the woman instructed about how to care for her newborn?	409 (99)	534 (99)	53 (98)	134 (98)	86 (100)	136 (100)	273 (100)	194 (98)	28 (100)	39 (100)
31. Was the woman reassured the she is going to be fine?	373 (90)	511 (95)	34 (62)	125 (91)	82 (95)	132 (97)	261 (96)	184 (93)	28 (100)	38 (95)
Mean (SD) of the average score	0.34 (0.13)	0.39 (0.10)	0.41 (0.13)	0.31 (0.13)	0.35 (0.10)	0.31 (0.13)	0.39 (0.10)	0.40 (0.10)	0.38 (0.13)	0.38 (0.10)
Mean (SD) of the average score for	0.34	0.39	0.40	0.36	0.35	0.32(0	0.39	0.39(0	0.38	0.38

For peer review only - http://bmjopen.bmj.com/site/about/guidelines.xhtml

complete checklist	(0.10)	(0.09)	(0.13)	(0.10)	(0.10)	.10)	(0.09)	.08)	(0.08)	(0.11)

For peer review only

For peer review only - http://bmjopen.bmj.com/site/about/guidelines.xhtml

Description of additional files

Additional File one: **Schema of the study design**

Additional File two: **A graphical representation of the overall satisfaction outcome score of the women stratified by hospital and time of intervention. Data presented are for complete MMISS only**

For peer review only

For peer review only - http://bmjopen.bmj.com/site/about/guidelines.xhtml

The effect of training doctors in communication skills on women's satisfaction with labour and delivery: a stepped wedge cluster randomised trial in Damascus

Journal:	*BMJ Open*
Manuscript ID:	bmjopen-2013-002674.R1
Article Type:	Research
Date Submitted by the Author:	29-May-2013
Complete List of Authors:	Bashour, Hyam; Faculty of Medicine, Damascus university ,<br>Kanaan, Mona; University of York, Department of Health Sciences<br>Kharouf, Mayada; Damascus University, Department of Family and Community Medicine<br>Abdulsalam, Asmaa; Damascus University, Department of Obstetrics and Gynecology<br>Tabbaa, Mohammed; Damascus University, Department of Obstetrics and Gynecology<br>Cheikha, Salah; Damascus University, Department of Obstetrics and Gynecology
**Primary Subject Heading**:	Obstetrics and gynaecology
Secondary Subject Heading:	Public health, Patient-centred medicine
Keywords:	Maternal medicine < OBSTETRICS, EPIDEMIOLOGY, Quality in health care < HEALTH SERVICES ADMINISTRATION & MANAGEMENT

SCHOLARONE™
Manuscripts

For peer review only

The effect of training doctors in communication skills on women's satisfaction with labour and delivery: a stepped wedge cluster randomised trial in Damascus

Hyam N Bashour[1] *, Mona Kanaan[2], Mayada H Kharouf[1], Asmaa A Abdulsalam [3], Mohammed A Tabbaa[3], Salah A Cheikha[3]

[1] Department of Family and Community Medicine, Faculty of Medicine, Damascus University, Damascus, Syria,

[2] Department of Health Sciences, University of York, York, United Kingdom

[3] Department of Obstetrics and Gynaecology, Faculty of Medicine, Damascus University, Damascus, Syria

* Corresponding author: hbashour@scs-net.org

Details:

HNB: PhD, hbashour@scs-net.org

MK: PhD, mona.kanaan@york.ac.uk

MHK: MSc, mkharouf@scs-net.org

AAA: MD asma@scs-net.org

MT: MD mmtabbaa@hotmail.com

SAC: MD senioreyad@hotmail.com

For peer review only

For peer review only - http://bmjopen.bmj.com/site/about/guidelines.xhtml

Abstract

Objectives: To determine the effect of training residents in interpersonal and communication skills on women's satisfaction with doctor-woman relationship in labour and delivery rooms**.**

Design: A stepped wedge cluster randomised trial

Setting: Four tertiary-care teaching maternity hospitals in Damascus, Syria

Participants: Two thousand women who gave birth to a living baby in the four study hospitals and consented to participate in the intervention took part in the study. Women with difficult labour and high-risk pregnancies were excluded**.** All were interviewed at home after discharge.

Interventions: A specially designed training package in communication skills that was delivered to all resident doctors at the four hospitals.

Primary outcome measures: The main outcome measure was women's satisfaction with interpersonal relationships in labour and delivery rooms measured via a series of questions on a Likert scale modified from the Medical Interview Satisfaction Scale.

Results: At the individual level, the mean for the average satisfaction score was 3.23 (SD: 0.72) out of a possible score of a 5 in the control group and 3.42 (SD: 0.73) in the intervention group. Using generalised linear mixed models, we were not able to detect a difference between the mean for the average satisfaction score of women in the intervention arm and that of women in the control arm; the 95% confidence interval associated with the effect of the intervention ranged from -0.08 to 0.15.

Conclusions: In spite of the slight changes in the observed residents' communication skills, the training package in communication skills does not seem to be associated with higher satisfaction scores of women**.** This raises the question whether training individuals without further structural changes in the delivery of care and without further reinforcement of the training can have an impact on improving the quality of doctor-patient communication.

Trial registration Number: ISRCTN80243969

For peer review only - http://bmjopen.bmj.com/site/about/guidelines.xhtml

Funding: Wellcome Trust

Key words: Women's satisfaction, Communication skills, Training, Stepped Wedge Cluster Randomised trial

For peer review only

For peer review only - http://bmjopen.bmj.com/site/about/guidelines.xhtml

Article Summary

'Article focus'

- The study aims to determine the effect of training residents in interpersonal and communication skills on women's satisfaction with doctor-woman relationship in labour and delivery rooms
- The context of the trial focuses on busy teaching maternity hospitals
- This work presents some expertise in a rather rare design of trials

'Key messages'

- In the context of a highly crowded and stressful environment where middle and low class Syrian women deliver, a specially designed training package in interpersonal and communication skills for residents did not achieve an overall improvement in women's satisfaction with labour and delivery.
- This study raises questions as to whether training individuals without more structural changes in the delivery of care can have an impact on improving the quality of doctor-patient communication. These are important questions to address and call for further research.
- Despite the lack of evidence from this study, the need to improve the interpersonal skills of medical doctors and obstetricians specifically should be reinforced, as good communication is central to quality health care.

Strengths and limitations of this study

The key strength of the study was in its stepped-wedge cluster randomised design characterized by being ethical and practical. One of the study limitations is being unable to link women's satisfaction with a specific doctor's behaviour relating to doctor-woman relationship as we observed actions rather than individual resident's behaviour for ethical as well as practical reasons. This is unavoidable due to the design's key considerations.

For peer review only - http://bmjopen.bmj.com/site/about/guidelines.xhtml

Background

There is a growing body of literature linking communication skills to a host of valued patient outcomes, including satisfaction, adherence, and positive health indicators [1]. As a result, communication in health-care, particularly between health-care professionals and patients, has attracted an increasing amount of attention at official and professional levels in recent years. Many academic and statutory bodies have been involved in initiatives to promote good communication including that for maternity care [2-3]. These initiatives are in part a response to increasing evidence from researchers that the quality of the interaction between patients and their care providers may have a significant effect on a variety of aspects of patient's well-being. These include satisfaction, knowledge and understanding, adherence with advice or treatment, quality of life, and psychological and other health outcomes [4-5].

Maternity care is an area of health-care in which the importance of good communication has received particular attention [3]. Studies of women's views of maternity care suggest that good communication is central in determining whether women are satisfied with the care that they receive or not as communication gives them the opportunity to be better informed, ask questions, and it signals understanding and respect [6-7]. Observational studies of communication between midwives and women during labour have highlighted the misunderstandings that can occur when communication is poor at this time and identified areas where communication could be improved [8].

Despite the acknowledged importance of communication in maternity care and the official recognition that communication is not always as good as it should be there have been few evaluations of strategies to improve communication between women and their care providers. Previous reviews of doctor-patient communication have not included any studies carried out in maternity care [4-5]. The review carried out by Rowe in 2002 [9] aimed to fill in that gap and thus identified and reviewed trials of the effectiveness of interventions aimed at improving communication between health professionals and women in maternity care. The review identified trials largely in the area of antenatal care but also identified a major gap in knowledge relating to communication in a number of key areas of labour, delivery and postnatal care. They recommended that trials

For peer review only - http://bmjopen.bmj.com/site/about/guidelines.xhtml

of interventions to improve communication between carers and women in labour and in the postnatal period would be particularly useful. All the studies in that review were carried out in developed countries, mainly in the United Kingdom.

Although good progress in reducing maternal mortality has been achieved in Syria; maternity care is still characterised by fragmentation of care, lack of protocols including those related to pain relief in labour, over-medicalisation of care, strong and even informal role of private sector, complex relationships between skilled attendants, and variation of quality of care received [10].

In Syria previous work in public hospitals and at the community level showed that continuous support of women during labour and delivery is virtually non-existing. Companionship is not allowed at hospitals, however in a population based study, women reported the existence of a companion at delivery in 37% of the cases [11]. Syrian women expressed their dissatisfaction with being left alone and with being attended to by care providers who are not equipped with good communication skills. It was evident that Syrian women taking part in the study were left without any social and emotional support during a critical period in their lives. In most occasions, they were not allowed to be accompanied by their relatives when in labour and delivery. There were many instances in which doctors and midwives themselves were sources of dissatisfaction due to lack of respect for patients and discrimination which is mainly related to the patient's socioeconomic status [12].

Furthermore, labour and delivery care is largely provided in overcrowded hospitals mainly by medical graduates with little or no training in communication and interpersonal skills and where midwives and nurses roles are typically marginal. These poor conditions might impact on women's satisfaction in the delivery process. This is in addition to the following issues: Syrian public hospital policies of not administering pain relief; the low societal status of women especially those from lower socioeconomic backgrounds; and the restrictive relations between females and males outside the family context. For example, previous research findings showed that in delivery suites eye-to-eye contact is not acceptable if the gender of the service provider is male [12].

Therefore, the main objective of this trial was to evaluate the effect of training resident doctors, as the main providers of care in teaching public maternity hospitals, in interpersonal and communication skills on women's

For peer review only - http://bmjopen.bmj.com/site/about/guidelines.xhtml

satisfaction with doctor-woman relationship in labour and delivery rooms using a modified version of the Medical Interview Satisfaction Scale (MISS-21) [13]. The secondary objective of this study was to measure the change in communicative behaviour of residents at labour and delivery rooms level using items of the Al-Galaa observational checklist [14].

For peer review only

For peer review only - http://bmjopen.bmj.com/site/about/guidelines.xhtml

Methods

Study design:

A stepped wedge cluster randomised design was used in this trial [15-16]. This particular design as reviewed by Brown and Lilford [16] involves sequential roll-out of the intervention to individuals or clusters over a number of time periods but the order in which participants receive the intervention is determined at random. In this trial, the roll-out of the training package was carried out at four time-points separated by two months each in addition to a baseline time-point; see supporting file one for a diagram of the trial design.

This resulted in observing 10 clusters under the control arm and another 10 clusters under the intervention arm. Each hospital contributed 5 clusters of participants. At baseline each hospital contributed a cluster to the control arm whereas at the last time-period each hospital contributed a cluster to the intervention arm. The timing of the implementation of the training package in each hospital determined when clusters from a hospital switched from the control arm to the intervention arm. A coded list of the hospitals was produced and a statistician blinded to the coding allocated randomly the timing of the introduction of the intervention to each hospital. By the end of the study, all eligible resident doctors in the study hospitals received the intervention.

Participants:

Participants in this study included care providers and women delivering at four public maternity hospitals in Damascus and its surroundings, Syria. The study hospitals included three hospitals affiliated to the Ministry of Health (MOH) and one university teaching hospital. The hospitals were the Maternity Teaching hospital at Damascus University (15000 deliveries each year), Al-Zahrawi hospital, which is the largest MOH hospital in Damascus (13000 deliveries each year), Douma hospital, which is a MOH district hospital in rural Damascus (7000 deliveries per year), and Harasta hospital, an MOH district hospital in rural Damascus (5000 deliveries per year) [17].

For peer review only - http://bmjopen.bmj.com/site/about/guidelines.xhtml

Care providers to undertake the training intervention were all residents registered for the year of field work (2008-2009) in the four hospitals. Midwives were excluded from this study since they had no role in the delivery process, except for assisting doctors, according to policies at those hospitals.

Women were included if they had normal vaginal birth or by C-section and gave birth to a living baby at the four hospitals during the study period. Women using the study public hospitals come largely from middle and low socio-economic backgrounds.

Informed consent was obtained from residents and women participating in the study. The study protocol was approved by the Institutional Review Board of Damascus University. All study hospitals agreed to take part in this research.

Intervention

The intervention consisted of exposing all residents in the four study hospitals to a training package in interpersonal and communication skills, using a specially designed training package that has been developed by the study team in cooperation with an international expert in the field. The objectives of the training package were: to recognize the impact of effective communication on women and child health during labour and delivery; to identify characteristics and principles of effective communication; to recognize and be able to overcome barriers to effective communication; and to enhance and reinforce the interpersonal communication skills of health providers, and improve their interactions with patients in general and women in labour in particular.

The content of the package included a story from a labour room. Issues regarding attitudes of health providers, overview of doctor-woman communication, non-verbal communication, building rapport, listening skills, effective interviewing, counselling and persuasion, and the ideal maternity ward were all explored. The methodology of the workshop was based on sharing concepts and ideas; self-assessment by means of tests and checklists; brainstorming, group work and plenary discussion; learning by experience; role-play; games and ice breakers. The training package is available for readers upon request.

For peer review only - http://bmjopen.bmj.com/site/about/guidelines.xhtml

All residents in the four hospitals received the intervention; their total number was 137. The training was carried out by a national trainer experienced in communication skills together with members of the research team who observed and facilitated the training. In total, nine training workshops took place at the Faculty of Medicine, Damascus University. The duration of each workshop was 20 hours in total delivered over three days. The rate of daily attendance ranged from 78 to 100%. A formal evaluation of the training workshops was carried out. The feedback from the evaluation was very positive with 97% of respondents saying that they would recommend the workshop. However, 82% envisaged barriers to implementation such as time pressure, overloaded work, and hospital's routine.

Data Collection

Data on women's satisfaction was collected prior to the intervention and at each instance of the four randomisation points from all hospitals. Data collection took place between April 2008 and January 2009. Participating women were interviewed at their homes within two weeks after delivery. Data were collected on demographic information (age, education, work status, husband's age, husband's education, husband's occupation and home ownership), information about the pregnancy and delivery (whether the mother is multiparous/nulliparous, gender of the newborn, type of delivery), and women were asked to complete a modified version of the Medical Interview Satisfaction Scale [13].

Furthermore, at each randomisation point data were collected to describe the communicative behaviour of care-providers. Trained observers were asked to fill in a checklist recording observations of communicative behaviour of residents at the labour and delivery rooms level rather than individual residents throughout the different hospital shifts using the Al-Galaa checklist [14]. Three shifts were observed per day which amounted to a total of 24 hours observation of each delivery room at each point of time. We have chosen this approach as we were more interested in the change of the behaviour at the service level rather than the actual change in the behaviour of an individual resident. Furthermore, this approach was expected to reduce problems that could arise if residents were observed individually. Thus, scores from observation were collected at the level of a round rather than scores for each resident. Pre- and post-training measurements were carried out in all four

For peer review only - http://bmjopen.bmj.com/site/about/guidelines.xhtml

hospitals at each point of time, thus the observation was carried out on an average of two to three weeks after the implementation of the training package in each hospital, with some variance between large and small hospitals. The training took longer time in the large hospitals because of the nature of the workshop and the necessity to implement it two to three times to cover all residents. Observers and field workers were blinded to the specific objectives of the study.

Qualitative data collection through six focus group discussions was also carried out prior to the field work to serve the design phase of this project. The qualitative methods aimed to understand the work environment of the residents and get their views concerning the importance of designing a training package in communication skills.

Outcomes

The primary outcome of this study was women's satisfaction with interpersonal and communication skills of doctors working in labour and delivery rooms, which could be seen as a patient outcome in the Ong's model [4]. The theoretical model for doctor-patient/woman communication as suggested by Ong and colleagues (1995) is a useful model to conceptualize an interventional research that aims to improve communication. It consists of background variables, actual content of communication as process indicators as well as patient outcomes including satisfaction in the short time. Women's satisfaction was measured using a Likert scale questionnaire investigating the communication skills of residents attending her. The questionnaire was based on the patient satisfaction questionnaire MISS-21 [13, 18]; the adapted questionnaire is referred to as the Modified Medical Interview Satisfaction Scale (MMISS). The validation exercise of our measurement tool which was implemented in Arabic language is to be reported elsewhere. The questionnaire had a total of 21 questions looking at the communication skills of the doctors in this setup. Women were asked to indicate their level of satisfaction with communicative practices of the attending resident on a 5-point Likert scale with 1 indicating strongly disagree to 5 indicating strongly agree. There were 8 negatively worded questions; the codes of these questions were reversed for analyses purposes. A score was then calculated by summing up the women's answers to the MMISS questions except for the first one, which was a general satisfaction question.

For peer review only - http://bmjopen.bmj.com/site/about/guidelines.xhtml

Therefore, the highest possible score was a 100 and the lowest 20 where higher values indicate that women were satisfied with the services provided to them. An average score was then calculated by dividing the score by the number of questions. Two scenarios were investigated. In the first scenario, the average score was computed for participants who provided information for the twenty items on the MMISS questionnaire and is referred to as the average score for complete MMISS. In the second scenario, the average score was computed for items with a response and is referred to as the average score only.

The secondary outcome measured the communicative behaviour of residents serving in labour and delivery rooms using sections related to communication of the observational checklist developed by Al-Galaa study in Egypt by Sholkamy and colleagues in 2003 [14]. The Al-Galaa study checklist was developed to document normal labour and delivery practices in an Egyptian hospital and covered areas such as management of labour, communication, postpartum and neonatal care; the instrument was tested and validated [14]. The checklist could be considered as a process evaluation in this work. It constituted of 31 questions and included items such as whether the attending doctor greeted the woman, identified himself/herself, explained the procedures of the medical examination he/she was about to undertake and communicated the findings, responded to the woman's questions, gave clear instructions about the different stages of labour, delivery and what is expected from the woman and whether the doctor gave any guidance with regard to post-delivery.

Sample size

Power calculations were carried out as in Hussey and Hughes (2007) [15]. A pilot study of 10 women resulted in a mean score of 3.165 (63.3 points on the satisfaction scale) and a standard deviation of 0.71. Based on a cluster size of a 100 at each step and hospital, a mean of 3.15 (63 points) and a standard deviation of 0.75 points, a difference of 0.2 (4 points) could be detected with 90% power given a significance level of 0.05. Therefore, the total sample size needed for the study was 2000 women (100 women x 4 hospitals x 5 time points) not accounting for non-response rate.

For peer review only - http://bmjopen.bmj.com/site/about/guidelines.xhtml

The total number of all residents in the four hospitals was 137. They were distributed as follows: 85 in the Maternity Teaching Hospital; 31 in Al-Zahrawi Hospital; 7 in Douma Hospital; and 14 in Harasta Hospital. They all received the intervention when it was offered at their respective hospitals.

Statistical methods

Baseline characteristics of the women were summarised using counts (percentages) for categorical variables and means (standard deviations (SD)) for continuous variables.

Statistical analysis was based on the principle of intention to treat. Comparison of the main and secondary outcomes between the control and intervention arms of the study was carried out at the individual level. A Generalized Linear Mixed Model (GLMM) was used to determine the size and direction of the difference between the control and intervention arms for the main and secondary outcomes. Estimates of the difference and 95% confidence intervals were calculated. All analyses were adjusted for clustering and time of the intervention. Additional analyses of the main outcome were conducted controlling for demographic variables. Furthermore, we used multiple imputations in order to address missing data.

Statistical analyses were carried out using STATA software (College Station, Texas) and the R language (www.r-project.org).

For peer review only - http://bmjopen.bmj.com/site/about/guidelines.xhtml

Results

A total of 2000 women delivering in the four hospitals were assessed for their satisfaction with patient-woman relationship. Figure1 describes the flow of participants using a CONSORT diagram [19]. All women approached agreed to participate in the trial.

[Figure 1: here]

Table 1 shows the characteristics of the women by treatment arm. The mean age of the women was 25 years (SD: 6 years). Approximately 95% were housewives. Three quarters had primary education only, 13% were illiterate whereas 12% had high school or further education qualifications. However, there was a differential in the distribution between hospitals with the percent illiterate ranging from 3.6% to 23%. This reflected the fact that two of the hospitals served an urban population whereas the other two served a rural population. The husband's mean age was 31 years (SD: 6.76 years). The vast majority of husbands were self-employed (41%), followed by being a manual worker (35%), and clerk (18%). The distribution of the husband's education was similar to that of the woman's education. Almost half owned their house whereas the other half lived in shared accommodation. A quarter of the women were nulliparous and only 2 women had a C-section

[Table 1 here]

The percentage of women agreed or strongly agreed with the statement of overall satisfaction with doctors' communication skills during labour/delivery ranged from 51% to 83% between hospitals in the control arm and 58% to 85% between hospitals in the intervention arm. These percentages were higher in the intervention arm for all hospitals except for Hospital 2 which decreased from 83% to 67% in the intervention arm. For presentation purposes, the hospitals were coded such that Hospital 1 represented the hospital that had the intervention delivered first and so forth.

For peer review only - http://bmjopen.bmj.com/site/about/guidelines.xhtml

For peer review only

Table 2 details the views of women on specific questions with regard to doctors' communication skills. The majority of women pointed-out that doctors did not identify themselves prior to the medical examination; the percentages were similar between the control and intervention arm. One third of the women in the intervention arm agreed/strongly agreed that the doctors' greeted them at the onset of the consultation compared to a fifth in the control arm. Around 40% of the women agreed/strongly agreed that the doctor looked at them when he/she talked to the woman in the control arm compared to 60% in intervention arm. Similar percentages were observed for whether the doctor listened to the woman with concern and without interruption. Almost 40% of the women agreed/strongly agreed that the doctor showed an interest in them in the control arm versus a third in the intervention arm. Forty-two percent of the women in the intervention arm agreed/strongly agreed that the doctor used some terms to calm them down and used his/her hands to assist/reassure them compared with 36% and 32% in the control arm, respectively. A small percentage reported that the doctor insulted them (6% control arm versus 3% intervention arm) or shouted/screamed at them (13% control arm versus 7% intervention arm). Only a quarter of the women agreed/strongly agreed that the doctor explained the examination steps before the clinical exams in the control arm compared to 29% in the intervention arm. A third agreed/strongly agreed that the doctor explained the results from the clinical exam in the control arm compared to a 41% in the intervention arm, however, Hospitals 1 and 2 had a decrease in the percentages who agreed/strongly agreed whereas Hospitals 3 and 4 had an increase. Only a quarter of those who responded to the questions whether the doctors explained alternative choices or helped the woman in making decisions agreed/strongly agreed to these statements.

[Table 2 here]

Table 3 provides summary statistics for the intervention and control arm for the two outcome scores: average score for MMISS and average score for complete MMISS. The median and interquartile range (Q1,Q3) score for the average score was 3.3 (2.8,3.7) in the control arm and 3.3 (3.0, 4.1) in the intervention arm; see Appendix for a graphical representation of the average score. Furthermore, the median and interquartile range

For peer review only - http://bmjopen.bmj.com/site/about/guidelines.xhtml

score for the average score of the complete MMISS was 3.4 (3.0, 3.9) in the control arm and 3.3 (3.1, 4.1) in the intervention arm (See additional file 2 for a graphical representation of the average score for complete MMISS).

[Table 3 here]

Regression estimates for the average score on intervention and time using GLMM are given in Table 3. The effect of the intervention was an average increase of 0.03 points; however, there was no evidence for statistical significance (95% CI: -0.08, 0.15). The estimates using complete MMISS implied that an average decrease of 0.13 in the intervention arm compared to the control arm (equivalent to 2.6 points on the original scale). Nevertheless, the effect was not statistically significant with a 95% CI (-0.29, 0.04). We also carriedadditional analyses adjusting for the woman's demographic characteristics. The results of the intervention effect were similar to the main analysis and none of the demographic variables was a significant predictor. We also used multiple imputations to account for missing data. The estimates of this analysis (data not shown) were similar to those reported here.

Table 4 reports on the secondary outcome, the observational checklist, and gives the numbers and percentages of when an item on the observational checklist, concerning communication between doctor and woman during labour/delivery, was not observed. Based on Table 4, the majority of doctors did not identify themselves, did not explain the steps of labour and delivery, did not explain to the woman the stages of labour and her role in it, and did not give any instructions about the steps that follow delivery or how to care for her new born. In nine out of the 31 items, the proportion of negative responses was consistently lower in the intervention arm compared to the control arm across the hospitals; data shown in supplementary file. In the intervention arm the proportion of doctors who called the woman by her name or title increased from 75% to 85%, those who asked for permission to examine the woman from 39% to 51%, and before the vaginal examination from 38% to 58%

For peer review only - http://bmjopen.bmj.com/site/about/guidelines.xhtml

compared to the control arm. The practice of covering the woman during delivery increased in the intervention arm to 81% from 41% though there were variations across hospitals; asking the woman to bend her knees increased to 85% from 61%; relaying information to other doctors in the team increased to 83% from 54%; and congratulated the woman on her delivery increased to 60% from 47% in the control arm.

Using GLMM regression, scores of the observational checklist at the shift level were compared pre and post intervention adjusting for clustering at the hospital level. This showed no evidence of statistical significance with an estimate of -0.01 (95% CI: -0.03, 0.02) for the intervention parameter.

[Table 4 here]

Discussion

Maternity care is an area of health care in which the importance of good communication has received particular attention as good communication skills are essential for high quality, effective and safe medical practice [1-3]. This trial was designed to determine the effectiveness of training residents at maternity wards in interpersonal and communication skills on women's satisfaction with doctor-woman relationship in labour and delivery rooms in four teaching hospitals in Damascus. Syrian women are largely delivered by medical graduates with little or no training in communication and interpersonal skills. Results of this trial did not provide evidence that the intervention improve women's satisfaction as measured by a modified version of the Medical Interview Satisfaction Scale. Communicative behaviour of the residents measured by an observational checklist was poor before the intervention and showed a slight improvement though not statistically significant after training on some selected items such as asking the woman's permission for the exam or asking her to bend her knees.

The reasons for those negative findings of our trial could be multifaceted. First, we have no reason to think that the negative findings are due to measurement errors having validated the tool and trained the field workers. However, the large numbers of care providers at maternity wards including midwives, nurses and junior doctors

For peer review only - http://bmjopen.bmj.com/site/about/guidelines.xhtml

and even servants in the wards could play a role on the overall satisfaction of women, given that midwives and nurses were not part of our target group for training as their role is mainly to assist doctors rather than taking a full charge of delivery. Furthermore, the policy implemented at Syrian public hospitals of not administering pain relief could also confound the impact of the intervention on satisfaction as women would definitely prefer it [11]. The training package was tailored to our setting and was informed by previous research findings; it allowed for Ong's useful model to conceptualize an interventional research that aims to improve communication and it ensured that the key tasks in communication are covered using effective teaching methods [4, 20]. However, lack of opportunity to reinforce the training could have played a negative role. Brown and colleagues suggested that communication skills programmes may need to be longer, more intensive, teach a broader range of skills, provide ongoing performance feedback, utilize patient feedback and use a variety of instruments to measure change in communication skills [21]. Furthermore, the working environment in these maternity wards is well characterized by long hours, crowded wards, pressure due to organizational hierarchy at hospital, and tension in work relationships between residents, nurses and midwives [22]. All those factors could have left our target group of residents under pressure, but more importantly could have left women feeling unsatisfied. A recent article by Berridge et al (2010) highlighted how poor communication in the delivery suites can compromise safe and efficient care and humane relationships [23].

The interpretation of the findings should also consider the women's status in a community within which the expectations of the women are very low but also where culture and gender issues are critical. Previous research findings showed that some means of communication, for example eye-to-eye communication, are not acceptable if gender of the provider is different from that of woman [12]. We also argue that the overall positive satisfaction of women in our observation can be due to the childbirth event by itself as being a pleasing experience as well as to getting the service free of charge in public hospitals by those women who come from a rather poor socio-economic class [12]. It was surprising to see that the very positive evaluation of the training sessions by the trainees did not translate to improved satisfaction of the women. However, we fully

For peer review only - http://bmjopen.bmj.com/site/about/guidelines.xhtml

support Bingham that stressed the importance of carrying out patient satisfaction surveys to help hospitals change or improve their childbirth policy [24].

Our study enrolled all public teaching maternity hospitals in Damascus. Therefore, our findings can be generalisable to overcrowded public delivery settings in Syria and in settings in developingcountries with similar characteristics of over crowdedness, over worked residents who are not trained in good communication skills and where the role of nurses and midwives is marginal. These findings suggest that training residents only without a structural change in the system and without further reinforcement might not be sufficient to improve women's satisfaction with the service provided.

Strengths and limitations

The key strength of the study was in its stepped-wedge cluster randomised design characterized by being ethical and practical [16], which resulted in the training package being delivered to all residents serving at the hospital thus complementing their medical training in communication skills that is missing from their curriculum. Furthermore, the study had a 100% response rate among women but there were some missing values on some of the questions. High response rate is very common in our culture. Conducting the trial in a developing country certainly contributes to the field as compared to the literature that comes mainly from developed countries [1-3, 9]. One of the study limitations is being unable to link women satisfaction with a specific doctor's behaviour of doctor-woman relationship as we observed actions rather than individual resident's behaviour for ethical as well as practical reasons. This is unavoidable due to the design's key considerations that all hospitals would receive the intervention eventually. Residents are the sole providers of care at labour and delivery in the teaching public hospitals in our setting and midwives are only marginally involved in care. Therefore, exclusions of nurses and midwives in this study should not be a major limitation in the current setting. The relatively short duration of the training and the lack of follow up within the hospital setting could have contributed to the negative findings in this study. Furthermore, the selection of public hospitals only in this study might have impacted the generalisability of the findings to other settings; however this setting is common in our region [25].

For peer review only - http://bmjopen.bmj.com/site/about/guidelines.xhtml

Conclusions

In the context of a highly crowded and stressful environment where middle and low class Syrian women deliver, a specially designed training package in interpersonal and communication skills for residents did not achieve an overall improvement in women's satisfaction with labour and delivery. However, certain items in doctors' behaviour have improved. It would be worth investigating whether the package would improve women's satisfaction in less stressful settings, but also it is worth looking at other possible interventions in maternity care practice such as doctor-midwife collaboration or attendance of birth companion in such settings.

Despite the lack of evidence from this study, the need to improve the interpersonal skills of medical doctors and obstetricians specifically should be reinforced, as good communication is central to quality health care [26].

Competing Interest

The authors declare no competing interests.

Authors' contributions

HNB and AAA developed the study concept and aims then initiated the project. MKH, MT, SAC and MK assisted further in the refinement of the concept and development of the protocol. HNB, MKH and AAA oversaw collection of data. MK did the statistical analysis. HNB and MK drafted the manuscript. All authors read and approved the final manuscript.

Acknowledgments

For peer review only

For peer review only - http://bmjopen.bmj.com/site/about/guidelines.xhtml

Our thanks are due to Mrs Hoda Hallab who worked with the team to develop the training package and deliver the training at inception. We are also grateful to the directors of the hospitals for facilitating the field work.

This paper is part of a larger regional research project on Choices and Challenges in Changing Childbirth in the Arab Region sponsored by the Center for Research on Population and Health at the American University of Beirut, Lebanon, with generous support from the Wellcome Trust (UK) [Grant Ref:074986].

For peer review only

For peer review only - http://bmjopen.bmj.com/site/about/guidelines.xhtml

References

1- Teutsch C. **Patient-doctor communication.** Med Clin North Am 2003; **87**:1115-45.

2- Department of Health. Changing childbirth. Part 1. Report of the Expert Maternity Group. London: HMSO, 1993.

3- Department of Health. Changing childbirth. Part 2: Survey of good communication practice in maternity services. London: HMSO, 1993.

4- Ong LML, de Haes JCJM, Hoos AM, Lammes FB. **Doctor-patient communication: a review of the literature**. Soc Sci Med 1995; **40**: 903-918.

5- Stewart MA. **Effective physician-patient communication and health outcomes: a review**. Can Med Ass J 1995; **152**: 1423-1433.

6- Garcia J, Redshaw M, Fitzsimons B, Keene J. First Class Delivery. A National Survey of Women's Views of Maternity Care. London: Audit Commission, 1998.

7- Kabakian-Khasholian T, Campbell O, Shediac-Rizkallah M, Ghorayeb F. (2000) **Women's experiences with maternity care: satisfaction or passivity?** Soc Sci Med, **51**:103-113.

8- Garcia J, Garforth S. Parents and new-born babies in the labour ward. In: Garcia J, Kilpatrick R, Richards M (eds.) The Politics of Maternity Care. Services for Childbearing Women in Twentieth-Century Britain. Oxford: Oxford University Press, 1990: 163-182.

9- Rowe RE, Garcia J, Macfarlane AJ, et al. **Improving communication between health professional and women in maternity care: a structured review**. Health Expect 2002; **5**: 63-83.

10- DeJong J, Bashour H, Kaddour A. Women's Health: progress and unaddressed issues. In: Jabbour S, Giacaman R, Khawaja M, Nuwayhid I (eds) Public Health in the Arab World. Cambridge University press 2012:

11- Bashour H, Abdulsalam A, Al-Faisal W, Cheikha S. **The patterns and determinants of maternity care in Damascus, Syria**. East Mediterr Health J. 2008; **14**: 398-405.

For peer review only - http://bmjopen.bmj.com/site/about/guidelines.xhtml

12- Hafez R, Bashour H, Abdulsalam A. Childbirth and its discontent: Experiences of Syrian Women. A paper presented at the Annual meeting of Reproductive Health Working Group (RHWG), Lebanon, July 11-13, 2004.

~~15 16~~ 13- Meakin R and Weinman J. **The Medical Interview Satisfaction Scale (MISS-21) adapted for British general practice**. Fam Pract 2002; **19**:257-263.

~~17 18~~ 14- Sholkamy H, Khalil K, Cherine M, Elnoury A, Breebaart M, Hassanein N. An observation checklist for facility-based normal labour and delivery practices: The Galaa Study. Monographs in Reproductive Health No. 5. Cairo; The Population Council, 2003.

15- Hussey A. and Hughes J.P. **Design and Analysis of stepped wedge cluster randomized trials**. Contemp Clin Trials 2007; **28**:182-191.

~~14~~ 16- Brown CA and Lilford RJ. **The stepped wedge trial design: a systematic review**. BMC Med Res Methodol 2006, **6**:54.

~~15~~- 17 Syrian Ministry of Health. www.moh.gov.sy

18- Holloway S, Porteous M, Cetnarskyj R, Anderson E, Rush R, Fry A, Gorman D, Steel M, Campbell H. 2004. **Patient satisfaction with two different models of cancer genetic services in south-east Scotland**. *Br J Cancer* **90**: 582–589.

19- Moher D, Hopewell S, Schulz KF, Montori V, Gøtzsche PC, Devereaux PJ, Elbourne D, Egger M, Altman DG, for the CONSORT Group. CONSORT 2010 Explanation and Elaboration: updated guidelines for reporting parallel group randomised trial. BMJ 2010;340:c869.

20- Maguire P and Pitceathly C. **Key communication skills and how to acquire them**. BMJ 2002; **325**:697-700.

21- Brown JB, Boles M, Mullooly JP, Levinson W. **Effect of clinician communication skills training on patient satisfaction. A randomized, controlled trial**. Ann Intern Med 1999; **131**:822–9.

22- Abdulasalam A. **Communication Skills! What does it mean to doctors and women: A qualitative study in Damascus maternity teaching hospitals**. Damascus University Journal for Health Sciences 2009; **25**:189-203. (In Arabic)

For peer review only

23- Berridge E, Mackintosh N and Freeth D. **Supporting patient safety: examining communication within delivery suite teams through contrasting approaches to research observation**. Midwifery 2010; **26**: 512-519.

24- Bingham D. **Helping hospitals change. Part 2: Childbirth education techniques to empower women**. J Perinatal Edu 2005; **14**: 51-55.

25- Choices and Challenges in Changing Childbirth Research Network. **Routines in facility-based maternity care: evidence from the Arab World**. BJOG: an International Journal of Obstetrics and Gynaecology 2005; **112**, 1270-1276.

26- Audit Commission. What seems to be the matter: Communication between hospitals and patients. HMSO, London. 1993.

For peer review only

For peer review only - http://bmjopen.bmj.com/site/about/guidelines.xhtml

Figures:

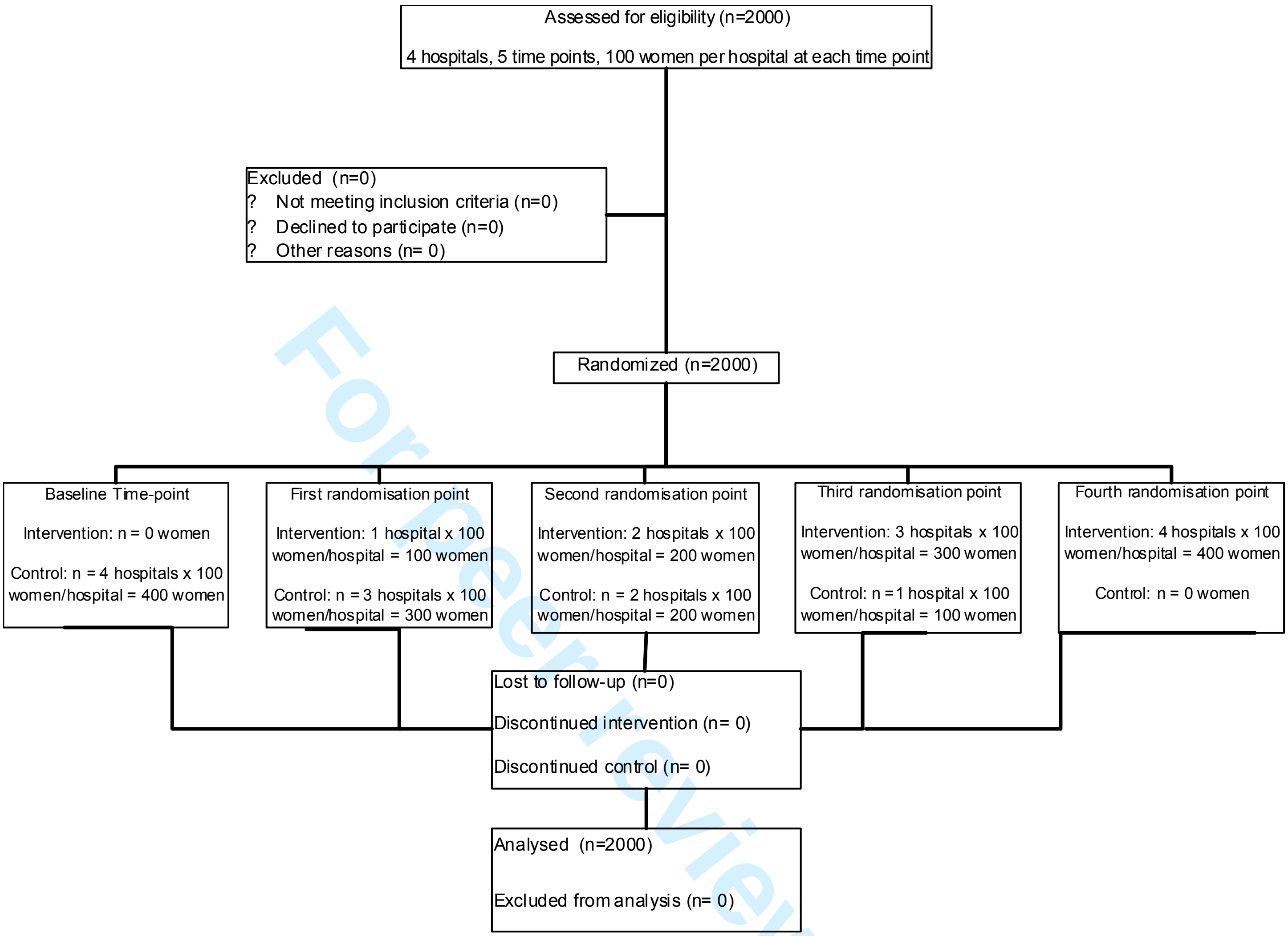

For peer review only

Figure 1: Flow of Participants

Tables:

For peer review only - http://bmjopen.bmj.com/site/about/guidelines.xhtml

Table 1: Demographic characteristics of the women by intervention arm. Numbers represent counts (percentage) unless otherwise stated.

	Control N = 1000	Intervention N = 1000		Control N = 1000	Intervention N = 1000
Mean age (SD)	25.02 (6.11)	25.10 (5.89)	Woman's Education		
Occupation: Housewife	949 (95)	953 (95)	Illiterate/Read and Write	140 (14)	102 (10)
Home Ownership: Share	488 (49)	470 (48)	Primary	745 (74)	761 (77)
Parity: Nulliparous	266 (27)	263 (27)	High School +	110 (11)	128 (13)
New born gender: Male	507 (52)	480 (49)	Husband's Education		
Mean husband's age (SD)	30.90 (6.91)	30.85 (6.62)	Illiterate/Read and Write	113 (11)	101 (10)
Husband's Occupation			Primary	781 (79)	743 (76)
Labourer (manual worker)	359 (36)	337 (34)	High School +	100 (10)	135 (14)
Clerk	160 (16)	198 (20)			
Self-employed	391 (40)	432 (44)			
Other	80 (8)	21 (2)			

For peer review only - http://bmjopen.bmj.com/site/about/guidelines.xhtml

Table 2: Numbers (percentages) of women who agreed/strongly agreed with various questions concerning their labour/delivery experience. The numbers compare all the intervention clusters to the control clusters.*

Item	Control Period N=1000	Intervention Period N=1000	Item	Control Period N=1000	Intervention Period N=1000
Overall satisfaction with doctors' communication skills during labour/delivery	619 (62)	713 (72)	Did the doctor listen to you with concern and without interrupting you?	413 (46)	551 (58)
Did the doctors identify themselves?	132 (13)	118 (12)	Was the doctor engaged in other issues, so you felt being unattended and annoyed?	165 (17)	67 (7)
Did the doctor greet you?	208 (21)	323 (32)	Did you feel let down by the way he/she was dealing with you?	66 (7)	31 (3)
Did the doctor look at you when talking to you?	434 (43)	599 (60)	Did the doctor explain all the steps before doing the clinical exam?	249 (25)	278 (29)
Did the doctor show interest in you as a person?	393 (39)	329 (33)	Did the doctor explain the findings from the clinical exam?	326 (34)	399 (41)
Did the doctor insult you?	55 (6)	29 (3)	Were you annoyed by the doctor explaining all the findings?	40 (5)	73 (10)
Did the doctor use humour to comfort you?	78 (8)	118 (12)	Did the doctor explain all the alternative choices to you?	176 (23)	145 (17)
Did the doctor use any terms to calm you down?	363 (36)	419 (42)	Did the doctor help you in making decisions?	175 (29)	158 (23)
Did the doctor use his/her hands to assist you or to reassure you?	314 (32)	409 (42)	Was the doctor annoyed from your questions and avoided answering them?	122 (14)	54 (6)
Did the doctor shout or scream at you?	125 (13)	71 (7)	Did the doctor use medical terms in explanation, thus they were not understood?	77 (8)	48 (5)

* Due to missing information some of the percentages do not tie up with the total number given.

For peer review only

For peer review only - http://bmjopen.bmj.com/site/about/guidelines.xhtml

Table 3: Summary statistics for the outcome of women satisfaction computed as the average score and average score for Complete MMISS. Regression analysis estimates for the average score and average score for complete MMISS on intervention and time controlling for clustering by hospital using GLMM. Estimates are presented together with 95% confidence interval (CI) limits as subscripts ($_{Lower}Est_{Upper}$).

	Average Score	Average Score Complete MMISS
Control: Median (Q1, Q3)	3.3 (2.8, 3.7)	3.4 (3.0, 3.9)
Intervention: Median (Q1, Q3)	3.3 (3.0, 4.1)	3.3 (3.1, 4.1)
GLMM Estimates	$_{Lower}Est_{Upper}$	$_{Lower}Est_{Upper}$
**Fixed-effects**		
intervention	$_{-0.08}0.03_{0.15}$	$_{-0.29}-0.13_{0.04}$
time: 2	$_{-0.08}0.02_{0.12}$	$_{-0.10}0.05_{0.19}$
time: 3	$_{-0.14}-0.03_{0.08}$	$_{-0.02}0.12_{0.27}$
time: 4	$_{-0.08}0.05_{0.18}$	$_{0.12}0.30_{0.49}$
time: 5	$_{-0.15}0.01_{0.16}$	$_{-0.03}0.17_{0.37}$
constant	$_{3.16}3.32_{3.48}$	$_{3.23}3.44_{3.64}$
**Random-effects**	Variance (SE)	Variance (SE)
**Hospital**	0. 02 (0.016)	0.03 (0.026)
**Residual**	0.51 (0.016)	0.45 (0.02)

For peer review only - http://bmjopen.bmj.com/site/about/guidelines.xhtml

Table 4: Numbers (and percentages) of negative responses for items on the observational checklist concerning communication between doctor and woman during labour/delivery, unless otherwise stated. Due to missing information some of the percentages do not tie up with the total number given. The columns compare the numbers all the intervention clusters to the control clusters.

	Control Period N = 565	Intervention Period N=631		Control Period N=565	Intervention Period N=631
Mean (SD) of the average score	0.34 (0.13)	0.39 (0.10)			
Mean (SD) of the average score for complete checklist	0.34 (0.10)	0.39 (0.09)			
Checklist Item			Checklist Item		
Did the doctor identify himself/herself?	539 (100)	621 (99)	Was the woman asked any questions at this stage?	168 (32)	94 (15)
Did the doctor call the woman by her name?	132 (24)	99 (16)	Did the woman ask any questions at this stage?	292 (56)	281 (46)
Did the doctor take the woman's permission to examine her?	326 (61)	305 (49)	Did the doctor respond to her queries?	275 (63)	342 (64)
Did the doctor explain the plan he/she will follow?	497 (92)	577 (92)	Was the doctor responsive to the woman's pain?	285 (59)	333 (57)
Before starting the vaginal examination, did the doctor			Was the woman encouraged at this stage?	186 (40)	239 (42)
take her permission for the exam?	335 (62)	262 (42)	Was the woman told about the proximity of labour?	242 (55)	363 (66)
close the door?	472 (88)	568 (91)	Was the woman told about the stages of labour?	399 (95)	533 (98)
cover the woman during the exam?	315 (59)	116 (18)	Was the woman told about her role during labour?	347 (83)	508 (94)
ask the woman to bend her knees?	210 (39)	79 (13)	During delivery, was the woman asked to push?	26 (6)	23 (4)
After completing the vaginal examination, did the doctor			Did they explain to the woman when and how to push?	237 (57)	230 (42)
tell the woman the results of the exam?	418 (78)	504 (81)	Did the woman ask any questions at this stage?	284 (68)	296 (55)
explain the next steps to be followed?	486 (91)	586 (94)	Did the doctor respond to her queries?	264 (77)	330 (72)
relay the findings to other team members?	241 (45)	102 (16)	Did the doctor inform the mother about her baby's status?	307 (75)	386 (72)
relay the findings to the midwives/nurses?	393 (80)	579 (93)	Was the woman congratulated after the delivery?	217 (53)	214 (40)
Did the doctor give instructions about eating and drinking?	463 (87)	516 (83)	Was the woman instructed about the process after the delivery?	392 (95)	515 (96)
Did the doctor give instructions about movement?	335 (63)	428 (70)	Was the woman instructed about how to care for her newborn?	409 (99)	534 (99)

For peer review only

For peer review only - http://bmjopen.bmj.com/site/about/guidelines.xhtml

Appendix:

Figure 2 gives the distribution of the average score by treatment arm.

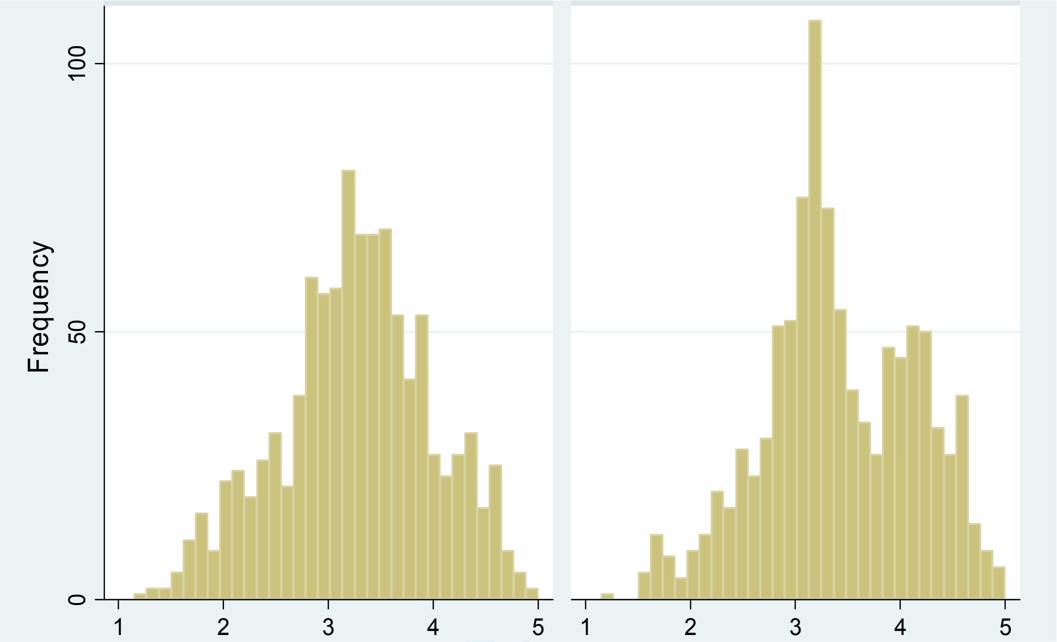

(a) (b)

Figure 2: Distribution of the average score by treatment arm (a) Control and (b) Intervention.

For peer review only - http://bmjopen.bmj.com/site/about/guidelines.xhtml

Description of supporting files

Additional File one: **Diagram of the study design**

Additional File two: **A graphical representation of the overall satisfaction outcome score of the women stratified by hospital and time of intervention. Data presented are for complete MMISS only**

Additional File three: **Results by intervention period.**

Additional File four**: Trial Protocol.**

For peer review only

For peer review only - http://bmjopen.bmj.com/site/about/guidelines.xhtml

~~Communication skills training and w~~~~Women's satisfaction with~~ ~~labour and doctors ' interpersonal and communication skills at labour and delivery~~The effect of training doctors in communication skills on women's satisfaction with labour and delivery: a ~~Stepped~~ stepped ~~Wedge~~ wedge ~~Cluster~~ cluster ~~R~~r~~andomized~~ randomised ~~Trial~~ trial in Damascus

Formatted

Formatted: Font: (Default) Arial, 16 pt

Formatted: Font: (Default) Arial, 16 pt

Formatted: Font: Not Bold

Hyam N Bashour[1] *, Mona Kanaan[2], Mayada H Kharouf[1], Asmaa A Abdulsalam [3], Mohammed A Tabbaa[3], Salah A Cheikha[3]

[1] Department of Family and Community Medicine, Faculty of Medicine, Damascus University, Damascus, Syria,

[2] Department of Health Sciences, University of York, York, United Kingdom

[3] Department of Obstetrics and Gynaecology, Faculty of Medicine, Damascus University, Damascus, Syria

* Corresponding author: hbashour@scs-net.org

Details:

HNB: PhD, hbashour@scs-net.org

MK: PhD, mona.kanaan@york.ac.uk

MHK: MSc, mkharouf@scs-net.org

AAA: MD asma@scs-net.org

MT: MD mmtabbaa@hotmail.com

For peer review only

For peer review only - http://bmjopen.bmj.com/site/about/guidelines.xhtml

SAC: MD senioreyad@hotmail.com

For peer review only

Abstract

Objectives: To determine the effect of training residents in interpersonal and communication skills on women's satisfaction with doctor-woman relationship in labour and delivery rooms.

Design: ~~a~~ A ~~Stepped~~ stepped ~~Wedge~~ wedge ~~Cluster~~ cluster ~~Randomized~~ ~~R~~randomised trial

Setting: Four tertiary-care teaching maternity hospitals in Damascus, Syria

Participants: Two thousand~~s~~ women who gave birth to a living baby in the four study hospitals and consented to participate in the intervention took part in the study. Women with difficult labour and high-risk pregnancies were excluded. All were interviewed at home after discharge.

Interventions: A specially designed training package in communication skills that was delivered to all resident doctors at the four hospitals.

Primary ~~and secondary~~ outcome measures: The main outcome measure was women's satisfaction with interpersonal relationships in labour and delivery rooms measured via a series of questions on a Likert scale modified from the Medical Interview Satisfaction Scale. ~~Secondary objectives focused on the communicative behaviour of residents serving in labour and delivery rooms using an observational checklist.~~

Results: At the individual level, the mean for the average satisfaction score was 3.23 (SD: 0.72) out of a possible score of a 5 in the control group and 3.42 (SD: 0.73) in the intervention group. Using generalised linear mixed models, we were not able to detect a difference between mean for the average satisfaction score of women in the intervention arm and that of women in the control arm~~the intervention and control group on the mean for the overall satisfaction of women~~; the 95% ~~Confidence~~ confidence ~~Interval~~ interval associated with the effect of the intervention ranged from -0.08 ~~-~~to ~~-~~0.15.

Conclusions: In spite of the slight changes in the observed residents' communication skills, the training package in communication skills does not seem to be associated with higher ~~overall~~ satisfaction scores of women. This raises the question whether training individuals without further structural changes in the delivery

For peer review only

1 of care and without further reinforcement of the training provided can have an impact on improving the quality

2 of doctor-patient communication.

3 **Trial registration Number: ISRCTN80243969**

4 **Funding:** Wellcome Trust

5

6 **Key words**: Women's satisfaction, Communication skills, Training, Stepped Wedge Cluster Randomized

7 Randomised trial

8

For peer review only

Article Summary

'Article focus'

- The study aims to determine the effect of training residents in interpersonal and communication skills on women's satisfaction with doctor-woman relationship in labour and delivery rooms
- The context of the trial focuses on busy teaching maternity hospitals
- This work presents some expertise in a rather rare design of trials

'Key messages'

- In the context of a highly crowded and stressful environment where middle and low class Syrian women deliver, a specially designed training package in interpersonal and communication skills for residents did not achieve an overall~~all~~ improvement in women's satisfaction with labour and delivery.
- This study raises questions as to whether training individuals without more structural changes in the delivery of care can have an impact on improving the quality of doctor-patient communication. These are important questions to address and call for further research.
- Despite the lack of evidence from this study, the need to improve the interpersonal skills of medical doctors and obstetricians specifically should be reinforced, as good communication is central to quality health care.

Strengths and limitations of this study

- The key strength of the study was in its stepped-wedge cluster randomised design characterized by being ethical and practical.
- One of the study limitations is being unable to link women's satisfaction with a specific doctor's behaviour ~~of~~relating to doctor-woman relationship as we observed actions rather than individual

Formatted: Default Paragraph Font, Font: (Default) Arial, 10 pt, Bold, English (U.S.)

Formatted: Font: (Default) +Headings CS (Times New Roman), 12 pt, Font color: Auto

Formatted: Justified

Formatted: Font: (Default) Arial, 10 pt, Bold, Font color: Auto

Formatted: Justified, Space After: 6 pt, Line spacing: Double

Formatted: Font: (Default) +Headings CS (Times New Roman), 12 pt, Font color: Auto

Formatted: List Paragraph, Line spacing: Double, Bulleted + Level: 1 + Aligned at: 0.25" + Indent at: 0.5"

Formatted: List Paragraph, Left, Bulleted + Level: 1 + Aligned at: 0.25" + Indent at: 0.5"

Formatted: List Paragraph, Line spacing: Double, Bulleted + Level: 1 + Aligned at: 0.25" + Indent at: 0.5"

Formatted: Font: (Default) +Headings CS (Times New Roman), 12 pt, Font color: Auto

Formatted: List Paragraph, Line spacing: Double, Bulleted + Level: 1 + Aligned at: 0.25" + Indent at: 0.5"

Formatted: Font: (Default) +Headings CS (Times New Roman), 12 pt, Font color: Auto

Formatted: Font: (Default) +Headings CS (Times New Roman), 12 pt, Font color: Auto

Formatted: Font: (Default) +Headings CS (Times New Roman), 12 pt, Font color: Auto

For peer review only
For peer review only - http://bmjopen.bmj.com/site/about/guidelines.xhtml

resident's[2] behaviour for ethical as well as practical reasons. This is unavoidable due to the design's key considerations.

Formatted: Font: (Default) +Headings CS (Times New Roman), 12 pt, Font color: Auto

Formatted: Font: Arial, 16 pt, Bold, Font color: Auto

Formatted: Font: (Default) +Headings CS (Times New Roman), 12 pt

For peer review only - http://bmjopen.bmj.com/site/about/guidelines.xhtml

Formatted: List Paragraph, Left

Formatted: Left, Indent: Left: 0.25"

Background

Formatted: Font: Arial, 16 pt, Bold, Font color: Auto

Formatted: Font: Arial, 16 pt, Bold

There is a ~~–~~growing body of literature linking communication skills to a host of valued patient outcomes, including satisfaction, adherence, and positive health indicators~~, is drawing increasing attention to the centrality of the interpersonal skills of healthcare providers to the delivery of quality medical care~~ [1]. As a result, communication in health-care, particularly between health-care professionals and patients, has attracted an increasing amount of attention at official and professional levels in recent years. Many academic and statutory bodies have ~~all~~ been involved in initiatives to promote good communication including that for maternity care [~~1~~2-3]. These initiatives are in part a response to increasing evidence from researchers that the quality of the interaction between patients and their care providers may have a significant effect on a variety of aspects of patient's well-~~-~~being. These include satisfaction, knowledge and understanding, adherence with advice or treatment, quality of life, and psychological and other health outcomes [4-5].

Maternity care is an area of health-care in which the importance of good communication has received particular attention [3]. Studies of women's views of maternity care suggest that good communication is central in determining whether women are satisfied with the care that they receive or not as communication gives them the opportunity to be better informed, ask questions, and it signals understanding and respect [6-7]. Observational studies of communication between midwives and women during labour have highlighted the misunderstandings that can occur when communication is poor at this time and identified areas where communication could be improved [8].

Despite the acknowledged importance of communication in maternity care and the official recognition that communication is not always as good as it should be~~,~~ there have been few evaluations of strategies to improve communication between women and their care providers. Previous reviews of doctor-patient communication have not included any studies carried out in maternity care [4-5]. The review carried out by Rowe in 2002 [9]

For peer review only - http://bmjopen.bmj.com/site/about/guidelines.xhtml

aimed to fill in that gap and thus identified and reviewed trials of the effectiveness of interventions aimed at improving communication between health professionals and women in maternity care~~, what this is what we can refer to call it as doctor-woman communication~~. The review identified trials largely in the area of antenatal care~~,~~ but also identified a major gap in knowledge relating to communication in a number of key areas of labour, delivery and postnatal care~~; mainly in labour~~. They recommended that trials of interventions to improve communication between carers and women in labour and in the postnatal period would be particularly useful. All the studies in that review were carried out in developed countries, mainly in the United Kingdom.

~~Maternity care in Syria, A~~Although ~~had achieved~~ good progress in reducing maternal mortality has been achieved in Syria~~,~~; maternity care ~~in Syria~~ is still characteris~~z~~ed by fragmentation of care, lack of protocols including those related to pain relief in labour, over-medicali~~z~~sation of care, strong and even informal role of private sector, complex relationships between skilled attendants, and variation of quality of care received [10].

In Syria~~,~~ previous work ~~both~~ in public hospitals and at the community level~~,~~ showed that continuous support of women during labour and delivery is virtually non-existing. Companionship is not allowed at hospitals, however in a population based study, women reported the existence of a companion at delivery in 37% of the cases [~~10~~11]. ~~Furthermore,~~ Syrian women expressed their dissatisfaction with being left alone and with being attended to by care providers who are not equipped with good communication skills. It was evident that Syrian women taking part in the study were left without any social and emotional support during a critical period in their lives. In most occasions, they were not allowed to be accompanied by their relatives when in labour and delivery. ~~However, they seem to be cared for by doctors and midwives with poor communication skills and little interpersonal competences.~~ There were many instances in which doctors and midwives themselves were sources of dissatisfaction due to lack of respect for patients and ~~bad or biased~~discrimination which is mainly related to patient's socioeconomic status ~~treatment~~ [~~11~~12].

Furthermore, ~~l~~Labour and delivery care is largely provided in overcrowded hospitals mainly by medical graduates with little or no training in communication and interpersonal skills and where midwives and nurses roles are typically marginal. These poor conditions might impact on women's satisfaction in the delivery process. This is in addition to the following issues: Syrian public hospital policies of not administering pain

Comment [m1]: By biased do you mean discriminate.

Formatted: Font: Times New Roman, Not Bold, Font color: Auto

Formatted: Font: Times New Roman, 12 pt, Font color: Auto

Comment [m2]: Hyam would you be able to provide some background information to address Jocelyn's comment about The paper could be strengthened in my view by describing more at the beginning of the paper the overall organization of maternity care in the country and its deficiencies, that have been the subject of some research. Particularly, the overcrowded nature of the public hospitals with high patient populations *(Do you have some statistics that explain the ratio of patients to doctors, waiting list)* and the delegation of much of the care given to women to residents (as well as the limited role of midwives) should be elaborated on. Moreover the issue, only appearing later in the paper, about the lack of pain relief given to women should be elaborated on earlier in the paper.

Formatted: Font: 12 pt

Formatted: Font: Times New Roman, Not Bold, Font color: Auto

Formatted: Font: 12 pt

relief; the low societal status of women especially those from lower socioeconomic backgrounds; and the restrictive relations between females and males outside the family context. For example, previous research findings showed that in delivery suites eye-to-eye contact is not acceptable if the gender of the service provider is male [12].

Therefore, the main objective of this trial was to evaluate the effect of training resident doctors, as the main providers of care in teaching public maternity hospitals, in interpersonal and communication skills on women's satisfaction with doctor-woman relationship in labour and delivery rooms using a modified version of the Medical Interview Satisfaction Scale (MISS-21) [13]. The secondary objective of this study was to measure the change in communicative behaviour of ~~the~~ residents at labour and delivery rooms level ~~after the training in communication skills~~ using ~~an observation checklist~~items of the Al-Galaa observational checklist [14].

Formatted: Font: 12 pt
Formatted: Font: 12 pt
Formatted: Font: 12 pt
Formatted: Font: 12 pt
Formatted: Font: 12 pt
Formatted: Font: 12 pt
Formatted: Font: 12 pt
Formatted: Font: 12 pt
Formatted: Font: Not Bold

For peer review only

For peer review only - http://bmjopen.bmj.com/site/about/guidelines.xhtml

Methods

Study design:

A stepped wedge cluster ~~randomized~~ randomised design was used in this trial [~~12~~~~13~~15-~~13~~~~14~~16]. This particular design as reviewed by Brown and Lilford [~~13~~~~14~~16] involves sequential roll-out of the intervention to individuals or clusters over a number of time periods but the order in which participants receive the intervention is determined at random. In this trial, the roll-out of the training package was carried out at four time-~~-~~points separated by two months each in addition to a baseline time-point; see supporting file one for a diagram of the trial design. ~~The stepped wedge design is efficient as clusters act as their own control, so fewer clusters are needed. All clusters receive intervention eventually in different point of time [14].~~

~~In this trial the designed training package (the intervention under study) was implemented in the four study hospitals (clusters) at four randomly selected time periods; randomly selected, after an initial baseline measurement (See the additional Filesupporting file one for a schema diagram of the trial design).~~ This resulted in observing 10 clusters under the control ~~(pre intervention) periods~~ arm and another 10 clusters under the intervention ~~periods~~arm. Each hospital contributed 5 clusters of participants. At baseline each hospital contributed a cluster to the control arm whereas at the last time-~~-~~period each hospital contributed a cluster to the intervention arm. The timing of the implementation of the training package in each hospital ~~cluster (hospital) was randomized~~~~randomised~~determined when clusters from a hospital switched from the control arm to the intervention arm. ~~Randomisation was carried out by a~~ A coded list of the hospitals was produced and a statistician ~~who was not aware of~~blinded to the ~~hospitals~~coding~~' names~~ allocated randomly the timing of the introduction of the intervention ~~is introduced~~ to each hospital. By the end of the study, ~~but code.~~ , ~~however~~ ~~a~~all~~All~~ eligible resident doctors in the study hospitals received the intervention~~ by the end of the study~~.

Formatted: Font: 12 pt

Formatted: Font: (Default) Times New Roman, 12 pt, Font color: Auto, English (U.K.)

Formatted: Font: (Default) Times New Roman, 12 pt, Font color: Auto, English (U.K.)

Formatted: Font: (Default) Times New Roman, 12 pt, Font color: Auto, English (U.K.)

Formatted: Font: (Default) Times New Roman, 12 pt, Font color: Auto, English (U.K.)

Formatted: Font: Times New Roman, 12 pt, Font color: Auto, Not Highlight

Formatted: Font: Times New Roman, 12 pt, Font color: Auto, Not Highlight

Participants:

Participants in this study included care providers and women delivering at four public maternity hospitals in Damascus and its surroundings, Syria. The study hospitals included three hospitals affiliated to the Ministry of Health (MOH) and one university teaching hospital. The hospitals were the Maternity Teaching hospital at

For peer review only

For peer review only - http://bmjopen.bmj.com/site/about/guidelines.xhtml

Damascus University (15000 deliveries each year), Al-Zahrawi hospital, which is the largest MOH hospital in Damascus (13000 deliveries each year), Douma hospital, which is a MOH district hospital in rural Damascus (7000 deliveries per year), and Harasta hospital, an MOH district hospital in rural Damascus (5000 deliveries per year) [~~14~~~~15~~17].

Care providers to undertake the training intervention were all residents registered for the year of field work (2008-2009) in the four hospitals. Midwives were excluded from this study since they had no role in the delivery process, except for assisting doctors, according to policies at those hospitals.

Women were included if they had normal vaginal birth or by C-section and gave birth to a living baby at the four hospitals during the study period. Women using the study public hospitals come largely from middle and low socio-economic backgrounds.

Informed consent~~s~~ ~~were~~ was obtained from ~~both~~ residents and women participating in the study. The study protocol was approved by the Institutional Review Board of Damascus University. All study hospitals ~~approved~~ agreed to take part ~~of~~ in this research.

Intervention

The intervention consisted of exposing all residents in the four study hospitals to a training package in interpersonal and communication skills, using a specially designed training package that has been developed by the study team in cooperation w

ith an international expert in the field. The objectives of the training package were ~~as follows~~: to recognize the impact of effective communication on women and child health during labour and delivery; to identify characteristics and principles of effective communication; to recognize and be able to overcome barriers to effective communication; and to enhance and reinforce the interpersonal communication skills of health providers, and improve their interactions with patients in general and women in labour in particular.

For peer review only

The content of the package included a story from a labour room. Issues regarding attitudes of health providers, overview of doctor-woman communication, non-verbal communication, building rapport, listening skills, effective interviewing, counselling and persuasion, and the ideal maternity ward were all explored. The methodology of the workshop was based on sharing concepts and ideas; self-assessment by means of tests and checklists; brainstorming, group work and plenary discussion; learning by experience; role-play; games and ice breakers. The training package is available for readers upon request.

All residents in the four hospitals received the intervention; their total number ~~came to~~was 137. The training was carried out by a national trainer experienced in communication skills together with members of the research team who observed and facilitated the training. In total, nine training workshops took place at the Faculty of Medicine, Damascus University. The duration of each workshop was 20 hours in total delivered over three days. The rate of d~~D~~aily ~~attendance~~ attendance ~~was monitored achieving a rate of~~ranged from 78 to 100% ~~for different hospitals~~. A formal ~~and~~ evaluation of the training workshops was carried out. The feedback from the evaluation was ~~and showed~~ very positive ~~responses, as~~with 97%- of ~~the~~ respondents saying~~said~~ that they would recommend the ~~course~~workshop. However, 82% ~~admitted foreseeing~~envisaged barriers to implementation such as time pressure; overloaded work; and hospitals' routine. ~~as well as overall evaluation of the training workshop by the trainees were very positive.~~

Data Collection

Data on women's satisfaction was collected prior to the intervention and at each instance of the four randomisation points from all hospitals. Data collection took place between April 2008 and January 2009. Participating women were interviewed at their homes within two weeks after delivery. ~~and d~~Data were collected on demographic information (age, education, work status, husband's age, husband's education, husband's occupation and home ownership), information about the pregnancy and delivery (whether the mother is multiparous/nulliparous, gender of the newborn, type of delivery), and women were asked to complete a modified version of the Medical Interview Satisfaction Scale ~~(MISS)~~ [~~15~~16~~6~~3].

Formatted: Font: 12 pt

Formatted: Font: (Default) Times New Roman, 12 pt, Not Italic

For peer review only - http://bmjopen.bmj.com/site/about/guidelines.xhtml

For peer review only

Furthermore, at each randomisation point data were collected to describe the communicative behaviour of care-providers. Trained observers were asked to fill in a checklist recording observations of communicative behaviour of residents at the labour and delivery rooms level rather than individual residents throughout the different hospital shifts using the Al-Galaa checklist [14]. Three shifts were observed per day which amounted to a total of 24 hours observation of each delivery room at each point of time. We have chosen this approach as we were more interested in the change of the behaviour at the service level rather than the actual change in the behaviour of an individual resident. Furthermore, this approach was expected to reduce problems that could arise if residents were observed individually. Thus, scores from observation were collected at the level of a round rather than scores for each resident. Pre- and post-training measurements were carried out in~~at~~ all four hospitals at~~in~~ each point of time, thus the observation was carried out on an average of two to three weeks after the implementation of the training package in each hospital, with some variance between large and small hospitals. The training took longer time in the large hospitals because of the nature of the workshop and the necessity to implement it two to three times to cover all residents. Observers and field workers were blinded to the specific objectives of the study.

Qualitative data collection through six focus group discussions was also carried out prior to the field work to serve ~~and through~~ the design phase of this project. The qualitative methods aimed to understand the work environment of the residents and get their views concerning the importance of designing a training package in communication skills.

Outcomes

The primary outcome of this study was women's satisfaction with interpersonal and communication skills of doctors working in labour and delivery rooms, which could be seen as a patient outcome in the Ong's model [~~4~~54]. The theoretical model for doctor-patient/woman communication as suggested by Ong and colleagues (1995) is a useful model to conceptualize an interventional research that aims to improve communication. It consists of background variables, actual content of communication as process indicators as well as patient outcomes including satisfaction in the short time. Women's satisfaction was measured using a Likert scale

For peer review only

For peer review only - http://bmjopen.bmj.com/site/about/guidelines.xhtml

questionnaire investigating the communication skills of residents attending her. The questionnaire was based on the patient satisfaction questionnaire MISS-21 [15162, 17813-18]; the adapted questionnaire is referred to as the Modified Medical Interview Satisfaction Scale (MMISS). The validation exercise of our measurement tool which was implemented in Arabic language is to be reported elsewhere. The questionnaire had a total of 21 questions looking at the communication skills of the doctors in this setup. Women were asked to indicate their level of satisfaction with communicative practices of the attending resident on a 5-point Likert scale with 1 indicating strongly disagree to 5 indicating strongly agree. There were 8 negatively worded questions; the codes of these questions were reversed for analyses purposes. A score was then calculated by summing up the women's answers to the MMISS questions except for the first one, which was a general satisfaction question. Therefore, the highest possible score was a 100 and the lowest 20 where higher values indicate that women were satisfied with the services provided to them. An average score was then calculated by dividing the score by the number of questions. [16]. Two scenarios were investigated. In the first scenario, the average score was computed for participants who provided information for the twenty items on the MMISS questionnaire and is referred to as the average score for complete MMISS. In the second scenario, the average score was computed for items with a response and is referred to as the average score only.

The secondary outcome measured the communicative behaviour of residents serving in labour and delivery rooms using sections related to communication of the observational checklist developed by Al-Galaa study in Egypt by Sholkamy and colleagues in 2003 [14]. The Al-Galaa study checklist was developed to document normal labour and delivery practices in an Egyptian hospital and covered areas such as management of labour, communication, postpartum and neonatal care; the instrument was tested and validated [14]. The checklist could be considered as a process evaluation in this workThe secondary outcome measured the communicative behaviour of residents serving in labour and delivery rooms using sections related to communication of the an observation checklist developed by Al-Galaa study in Egypt. The That checklist was tested and validated by Sholkamy and colleagues in 2003 [1718]. The checklist could be considered as a process evaluation in this work. I; it constituted of 31 questions and . It included items such as whether the attending doctor greeted the

Formatted: Font: 12 pt, Not Highlight

For peer review only - http://bmjopen.bmj.com/site/about/guidelines.xhtml

woman, identified himself/herself, explained the procedures of the medical examination he/she was about to undertake and communicated the findings, responded to the woman's questions, gave clear instructions about the different stages of labour, delivery and what is expected from the woman and whether the doctor gave any guidance for the next steps with regard to post delivery.

Sample size

Power calculations were carried out as in Hussey and Hughes (2007) [121315]. A pilot study of 10 women resulted in a mean score of 3.165 (63.3 points on the satisfaction scale) and a standard deviation of 0.71. Based on a cluster size of a 100 at each step and hospital, a mean of 3.15 (63 points) and a standard deviation of 0.75 points, a difference of 0.2 (4 points) could be detected with 90% power given a significance level of 0.05. Therefore, the total sample size needed for the study was 2000 women (100 women x 4 hospitals x 5 time points) not accounting for non-response rate.

The total number of all residents in the four hospitals was 137. They were distributed as follows: 85 in the Maternity Teaching Hospital; 31 in Al-Zahrawi Hospital; 7 in Douma Hospital; and 14 in Harasta Hospital. They all received the intervention when it was offered at their respective hospitals.

~~The total number of all residents in the four hospitals who received the intervention was 137. They were distributed as follows: 85 in the Maternity Teaching Hospital; 31 in Al-Zahrawi Hospital; 7 in Douma Hospital; and 14 in Harasta Hospital.~~

Statistical methods

Baseline characteristics of the women were summarised using counts (percentages) for categorical variables, and means (standard deviations (SD)) ~~and medians (inter-quartile ranges)~~ for continuous variables.

Statistical analysis was based on the principle of intention to treat. Comparison of the main and secondary outcomes between the control ~~(pre-intervention)~~ and intervention armsperiods of the study was carried out at the individual level. ~~Regression analysis was done using~~ A Generalized Linear Mixed Model (GLMM) was used to determine the size and direction of the difference between the control and intervention arms for the main

Formatted: Font: 12 pt, Not Highlight

Formatted: Body Text, Justify Low, Indent: First line: 0"

Formatted: Font: (Default) Times New Roman, 12 pt, Font color: Auto

Formatted: Normal, Justified, Don't adjust space between Latin and Asian text, Don't adjust space between Asian text and numbers

Formatted: Font: Times New Roman, 12 pt, Font color: Auto

Formatted: Font: Times New Roman, 12 pt, Font color: Auto

Formatted: Font: Times New Roman, 12 pt, Font color: Auto

For peer review only

For peer review only - http://bmjopen.bmj.com/site/about/guidelines.xhtml

and secondary outcomesperiods of the study. Estimates of the difference and 95% confidence intervals were calculated. All analyses were adjusted for clustering and time of the intervention. Additional analyses of the main outcome were conducted controlling for demographic variables. To determine our estimates, we used a variety of statistical techniques that took into account the study design as suggested by Hussey and Hughes in 2007 [121315]. Therefore, we reported estimates based on using one of the following estimation methods Linear Mixed Models (LMM) where equal sizes of clusters is a must, Generalised Estimating Equations (GEE) and Generalized Linear Latent and Mixed Models (GLLAMM) to ensure the robustness of the estimates specifically if the assumption of normality for the linear model is not met. In each case, the following model was fitted to the data:

$$y_{ijk} = \mu + C_j + S_k + X_{jk}\theta + e_{ijk} \qquad (1)$$

In model (1), y_{ijk} is the outcome of unit i (for example, woman satisfaction score for the primary outcome) in hospital j (j = 1, …, 4) at time point k (k = 1, …,5), C_j is a random effect for hospital j such that $C_j \sim N(0, \tau^2)$, S_k is a fixed effect corresponding to time point k (S_1=0 for identifiability), X_{jk} is an indicator of the intervention mode in hospital j at time point k (1=post /intervention; 0= pre intervention/control), θ is the treatment effect, e_{ijk} are random errors distributed as $N(0, \sigma^2)$. Therefore, an estimate of θ that is significantly different from zero would indicate an intervention effect. Furthermore, wWe used multiple imputations in the statistical models in order to address missing data.

Statistical analyses were carried out using STATA software (College Station, Texas) and the R language (www.r-project.org).

Formatted: Font: Times New Roman, 12 pt, Font color: Auto

Formatted: Font: Times New Roman, 12 pt

Formatted: Font: 12 pt

Formatted: Justified, Indent: First line: 0"

For peer review only

Results

A total of 2000 women delivering in the four hospitals were assessed for their satisfaction with patient-woman relationship. Figure1 describes the flow of participants using a CONSORT diagram [19]. All women approached agreed to participate in the trial.

[Figure 1~~11~~: here]

Table 1 shows the characteristics of the women ~~across the different randomisation's points of time~~by treatment arm. The mean age of the women was 25 years (SD: 6 years). Approximately 95% were housewives. Three quarters had primary education only, 13% were illiterate whereas 12% had high school or further education qualifications. However, there was a differential in the distribution between hospitals with the percent illiterate ranging from 3.6% to 23%. This reflected the fact that two of the hospitals served an urban population whereas the other two served a rural population. The husband's mean age was 31 years (SD: 6.76 years). The vast majority of husbands were self-employed (41%), followed by being a manual worker (35%), and clerk (18%). The distribution of the husband's education was similar to that of the woman's education. Almost half owned their house whereas the other half lived in shared accommodation. A quarter of the women were nulliparous and only 2 women had a C-section

[Table 1 here]

The percentage of women agreed or strongly agreed with the statement of overall satisfaction with doctors' communication skills during labour/delivery ranged from 51% to 83% between hospitals in the control ~~periods~~arm and 58% to 85% between hospitals in the intervention ~~periods~~arm. These percentages were higher in the intervention ~~periods~~arm for all hospitals except for ~~hospital~~ Hospital 2 which decreased from 83% to 67% in the intervention ~~periods~~arm. ~~Figure 1 gives a graphical representation of these percentages. For presentation purposes, the hospitals were coded such that hospital 1 represented the hospital that had the intervention~~

Formatted: Font: Times New Roman, 12 pt, Font color: Auto

Formatted: Line spacing: Double

Formatted: Font: Times New Roman, 12 pt, Font color: Auto

Formatted: Font: 12 pt

Formatted: Font: Bold, Italic, Expanded by 0.5 pt

Formatted: Centered

Formatted: Font: (Default) Arial, Italic, Expanded by 0.5 pt

Formatted: Font: (Default) Arial, 9 pt, Italic, Font color: Auto, Expanded by 0.5 pt

Formatted: Font: (Default) Arial, Italic, Expanded by 0.5 pt

Formatted: Font: Italic, Expanded by 0.5 pt

For peer review only

For peer review only - http://bmjopen.bmj.com/site/about/guidelines.xhtml

delivered first which was time 2 and so forth; time 1 was the baseline. The x-axis represents the different overall satisfaction categories at each time point (1 to 5). Based on this figure, women were generally satisfied with their birthing experience; inspection of this figure does not reveal striking differences between the control and intervention periods. For presentation purposes, the hospitals were coded such that Hospital 1 represented the hospital that had the intervention delivered first which was time 2 and so forth.; time 1 was the baseline.

Table 2 also details the views of women on specific questions with regard to doctors' communication skills. The majority of women pointed-out that doctors did not identify themselves prior to the medical examination; the percentages were similar between the control and intervention periodsarm. One third of the women in the intervention period arm agreed/strongly agreed that the doctors' greeted them at the onset of the consultation compared to a fifth in the control periodsarm. Around 40% of the women agreed/strongly agreed that the doctor looked at them when he/she talked to the woman in the control periodsarm compared to 60% in intervention periodsarm. Similar percentages were observed for whether the doctor listened to the woman with concern and without interruption. Almost 40% of the women agreed/strongly agreed that the doctor showed an interest in them in the control periodsarm versus a third in the intervention periodsarm. Forty-two percent of the women in the intervention periodsarm agreed/strongly agreed that the doctor used some terms to calm them down and used his/her hands to assist/reassure them compared with 36% and 32% in the control periodsarm, respectively. A small percentage reported that the doctor insulted them (6% control periodsarm versus 3% intervention periodsarm) or shouted/screamed at them (13% control periodsarm versus 7% intervention periodsarm). Only a quarter of the women agreed/strongly agreed that the doctor explained the examination steps before the clinical exams in the control periodsarm compared to 29% in the intervention periodsarm. A third agreed/strongly agreed that the doctor explained the results from the clinical exam in the control periodsarm compared to a 41% in the intervention periodsarm, however, Hospitals 1 and 2 had a decrease in the percentages who agreed/strongly agreed whereas Hospitals 3 and 4 had an increase. Only a quarter of those who responded to the questions whether the doctors explained alternative choices or helped the woman in making decisions agreed/strongly agreed to these statements.

For peer review only

[Table 2 here]

Table 3 provides summary statistics for the intervention and control ~~periods~~arm ~~stratified by hospital~~ for the two outcome scores: ~~, i.e.~~ average score for MMISS and average score for complete MMISS. The median and interquartile range (Q1,Q3) score for the average score was 3.3 (2.8,3.7) in the control arm ~~3.23 (SD: 0.72)~~ and 3.3 (3.0, 4.1) ~~3.42 (SD: 0.73) in the control and~~ in the intervention ~~periods~~arm~~, respectively~~; see Appendix for a graphical representation of the average score. Furthermore, the median and interquartile range score for the average score ~~in~~ of the complete MMISS was 3.4 (3.0, 3.9)~~3.43 (SD: 0.71)~~ in the control ~~periods~~arm and 3.3 (3.1, 4.1)~~3.54 (SD: 0.66)~~ in the intervention ~~periods~~arm (See additional file 2 for a graphical representation of the average score for complete MMISS).

Formatted: Font: (Default) Times New Roman, 12 pt, Not Bold, Font color: Auto

Formatted: Font: (Default) Times New Roman, 12 pt, Not Bold

Formatted: Font: (Default) Times New Roman, 12 pt, Not Bold

Formatted: Font: (Default) Times New Roman, 12 pt, Not Bold, Font color: Auto

Formatted: Font: (Default) Times New Roman, 12 pt, Not Bold, Font color: Auto

Formatted: Font: (Default) Times New Roman, 12 pt, Not Bold, Font color: Auto

Formatted: Font: (Default) Times New Roman, 12 pt, Not Bold

Formatted: Font: (Default) Times New Roman, 12 pt, Not Bold

Formatted: Font: (Default) Times New Roman, 12 pt, Not Bold

[Table 3 here]

Regression estimates for the average score on intervention and time using GLMM~~, GEE and GLLAMM techniques to adjust for the clustering by hospital~~ are given in Table 43. The effect of the intervention was an average increase of 0.03 points; however, there was no evidence for statistical significance (95% ~~Confidence Interval (~~CI~~)~~: -0.08, 0.15 ~~using GLAMM~~). T~~However, t~~he estimates using complete MMISS implied that an average decrease of 0.13 in the intervention arm compared to the control arm (equivalent to 2.6 points on the original scale). Nevertheless~~As before~~, the effect was not statistically significant with a 95% CI (-0.29, 0.04). ~~using GLAMM.~~ We also carried ~~an~~ additional analyses~~is~~ adjusting for the woman's demographic characteristics. The results of the intervention effect were similar to the main analysis and none of the demographic variables was a significant predictor. We also used multiple imputations to account for missing data. The estimates of this analysis (data not shown) were similar to those reported here. ~~before.~~

For peer review only - http://bmjopen.bmj.com/site/about/guidelines.xhtml

[Table 4 here]

Table 45 reports on the secondary outcome, the observational checklist, and gives the numbers and percentages of when an item on the observational checklist, concerning communication between doctor and woman during labour/delivery, was not observed. Based on Table 4, tThe majority of doctors did not identify themselves, did not explained the steps that of labour and delivery, did not explained to the woman the stages of labour and her role in it, and did not give any instructions about the steps that follow delivery or how to care for her new born. In nine out of the 31 items, the proportion of no negative responses was consistently lower in the intervention periodsarm compared to the control periodsarm across the hospitals; data shown in supplementary file. In the intervention arm tThe proportion of doctors who called the woman by her name or title increased from 75% to 85%, and those who asked for permission to examine the woman in general from 39% to 51%, and before the vaginal examination from 38% to 58% compared to the control arm. The practice of covering the woman during delivery increased in the intervention periodsarm to 81% from 41% though there were variations across hospitals; asking the woman to bend her knees increased to 85% from 61%; relaying information to other doctors in the team increased to 83% from 54%; and congratulated the woman on her delivery increased to 60% from 47% in the control periodsarm.

Using GLMM a regression approach similar to that described above, scores of the observational checklist at the shift level were were compared pre and post intervention adjusting for clustering at the hospital level. This showed no evidence of statistical significance with an estimate for the observational checklist of -0.01 (95% CI: -0.03, 0.02) for the intervention parameter.

[Table 5 4 here]

For peer review only

Discussion

Maternity care is an area of health care in which the importance of good communication has received particular attention as good communication skills are essential for high quality, effective and safe medical practice [1-3]. This trial was designed to determine the effectiveness of training residents at maternity wards in interpersonal and communication skills on women's satisfaction with doctor-woman relationship in labour and delivery rooms in four teaching hospitals in Damascus. Syrian women are largely delivered by medical graduates with little or no training in communication and interpersonal skills. Results of this trial did not provide evidence that the intervention improve women's satisfaction as measured by a modified version of the Medical Interview Satisfaction Scale. Communicative behaviour of the residents measured by an observational checklist was poor before the intervention and showed a slight improvement though not statistically significant after training on some selected items such as asking the woman's permission for the exam or asking her to bend her knees.

The reasons for those negative findings of our trial could be multifaceted. First, we have no reason to think that the negative findings are due to measurement errors having validated the tool and trained the field workers. However, the large numbers of care providers at maternity wards including midwives, nurses and junior doctors and even servants in the wards could play a role on the overall satisfaction of women, given that midwives and nurses were not part of our target group for training as their role is mainly to assist doctors rather than taking a full charge of delivery. Furthermore, the policy implemented at Syrian public hospitals of not administering pain relief could also confound the impact of the intervention on satisfaction as women would definitely prefer it [11]. The training package was tailored to our setting and was informed by previous research findings; it allowed for Ong's useful model to conceptualize an interventional research that aims to improve communication and it ensured that the key tasks in communication are covered using effective teaching methods [4, ~~18~~19]. However, lack of opportunity to reinforce the training could have played a negative role. Brown and colleagues suggested that communication skills programmes may need to be longer, more intensive, teach a broader range of skills, provide ongoing performance feedback, utilize patient feedback and use a variety of instruments to measure change in communication skills [~~19~~21~~0~~]. Furthermore, the working

For peer review only - http://bmjopen.bmj.com/site/about/guidelines.xhtml

environment in which residents and doctors work in these maternity wards is well characterized by long hours, crowded wards, pressure due to organizational hierarchy at hospital, and lack of healthy workingtension in work relationships with between residents, nurses and midwives [20231]. All those factors could have left our target group of residents under pressure, but more importantly could have left women feeling unsatisfied. A recent article by Berridge et al (2010) highlighted how poor communication in the delivery suites can compromise safe and efficient care and humane relationshipsthose areas that deeply examine communication within delivery suite, as well [2122].

The interpretation of the findings should also consider the women's status in a community within which the expectations of the women are very low but also where culture and gender issues are critical. Previous research findings showed that some means of communication, for example (eye to eye communication for example) are not acceptable if gender of the provider is different from that of woman [1112]. We also argue that the overall positive satisfaction of women in our observation can be due to the childbirth event by itself as being a pleasing experience as well as to getting the service free of charge in public hospitals by those women who come from a rather poor socio-economic class [1112]. It was surprising to see that the very positive evaluation of the training sessions by the trainees did not translate to improved satisfaction of the women. However, we fully support Bingham that stressed the importance of carrying out patient satisfaction surveys to help hospitals change orchange or improve theirimprove their childbirth policy [22243].

Our study enrolled all public teaching maternity hospitals in Damascus. Therefore, our findings can be generalisable to overcrowded public delivery settings in Syria and in settings in developing countries with similar characteristics of over crowdedness, over worked residents who are not trained in good communication skills and where the role of nurses and midwives is marginal. Our findings can be generalisable to overcrowded public delivery suites settings providing services to women from low socio economic background in developing countries where residents are not trained in good communication skills and where the role of nurses and midwives is marginal. These findings suggest that training residents only without a structural change in the

For peer review only - http://bmjopen.bmj.com/site/about/guidelines.xhtml

system and without further reinforcement might not be sufficient to improve women's satisfaction with the service provided.

Strengths and limitations

The key strength of the study was in its stepped-wedge cluster randomised design characterized by being ethical and practical [131416], which resulted in the training package being delivered to all residents serving at the hospital thus complementing their medical training in communication skills that is missing from their curriculum. Furthermore, the study had a 100% response rate among women but there were some missing values on some of the questions. High response rate is very common in our culture. Conducting the trial in a developing country certainly contributes to the field as compared to the literature that comes mainly from developed countries [1-3, 9]. The study potentially contributes to the experience conducting unfamiliar research design; namely the stepped wedge design [1416].

One of the study limitations is being unable to link women satisfaction with a specific doctors' behaviour of doctor-woman relationship as we observed actions rather than individual residents' behaviour for ethical as well as practical reasons. This is unavoidable due to the design's key considerations that all clusters (hospitals) would receive the intervention eventually and that clusters serves as their own control. Residents are the sole providers of care at labour and delivery in the teaching public hospitals in our setting and midwives are only marginally involved in care. Therefore, eExclusions of nurses and midwives in this study should not be a major limitation in the current setting. The relatively short duration of the training and the lack of follow up within the hospital setting could have contributed to the negative findings in this study. Furthermore, the selection of public hospitals only in this study might have impacted the generaliszability of the findings to other settings; however this setting is very common in our region [23254].

Conclusions

For peer review only

For peer review only - http://bmjopen.bmj.com/site/about/guidelines.xhtml

In the context of a highly crowded and stressful environment where middle and low class Syrian women deliver, a specially designed training package in interpersonal and communication skills for residents did not achieve an overall improvement in women's satisfaction with labour and delivery. However, certain items in doctors' behaviour have improved. It would be worth investigating whether the package would improve women's satisfaction in less stressful settings, but also it is worth looking at other possible interventions in maternity care practice such as doctor-midwife collaboration or attendance of birth companion in such settings.

Despite the lack of evidence from this study, the need to improve the interpersonal skills of medical doctors and obstetricians specifically should be reinforced, as good communication is central to quality health care [~~24~~2~~5~~6].

Competing Interest

The authors declare no competing interests.

Authors' contributions

HNB and AAA developed the study concept and aims then initiated the project. MKH, MT, SAC and MK assisted further in the refinement of the concept and development of the protocol. ~~HHB~~HNB, MKH and AAA oversaw collection of data. MK did the statistical analysis. ~~HHB~~ HNB and MK drafted the manuscript. All authors read and approved the final manuscript.

Acknowledgments

Our thanks are due to Mrs Hoda Hallab who worked with the team to develop the training package and deliver the training at ~~kick-off point~~inception. We are also grateful to the directors of the hospitals for facilitating the field work.

For peer review only - http://bmjopen.bmj.com/site/about/guidelines.xhtml

This paper is part of a larger regional research project on Choices and Challenges in Changing Childbirth in the Arab Region sponsored by the Center for Research on Population and Health at the American University of Beirut, Lebanon, with generous support from the Wellcome Trust (UK) [Grant Ref:074986].

For peer review only

References

1- Teutsch C. **Patient-doctor communication.** Med Clin North Am 2003; **87**:1115-45.

2- Department of Health. Changing childbirth. Part 1. Report of the Expert Maternity Group. London: HMSO, 1993.

3- Department of Health. Changing childbirth. Part 2: Survey of good communication practice in maternity services. London: HMSO, 1993.

4- Ong LML, de Haes JCJM, Hoos AM, Lammes FB. **Doctor-patient communication: a review of the literature**. Soc Sci Med 1995; **40**: 903-918.

5- Stewart MA. **Effective physician-patient communication and health outcomes: a review**. Can Med Ass J 1995; **152**: 1423-1433.

6- Garcia J, Redshaw M, Fitzsimons B, Keene J. First Class Delivery. A National Survey of Women's Views of Maternity Care. London: Audit Commission, 1998.

7- Kabakian-Khasholian T, Campbell O, Shediac-Rizkallah M, Ghorayeb F. (2000) **Women's experiences with maternity care: satisfaction or passivity?** Soc Sci Med, **51**:103-113.

8- Garcia J, Garforth S. Parents and new-born babies in the labour ward. In: Garcia J, Kilpatrick R, Richards M (eds.) The Politics of Maternity Care. Services for Childbearing Women in Twentieth-Century Britain. Oxford: Oxford University Press, 1990: 163-182.

9- Rowe RE, Garcia J, Macfarlane AJ, et al. **Improving communication between health professional and women in maternity care: a structured review**. Health Expect 2002; **5**: 63-83.

10- DeJong J, Bashour H, Kaddour A. Women's Health: progress and unaddressed issues. In: Jabbour S, Giacaman R, Khawaja M, Nuwayhid I (eds) Public Health in the Arab World. Cambridge University press 2012. ~~10- DeJong J, Bashour H, Kaddour A.~~

~~10~~11- Bashour H, Abdulsalam A, Al-Faisal W, Cheikha S. **The patterns and determinants of maternity care in Damascus, Syria**. East Mediterr Health J. 2008; **14**: 398-405.

For peer review only - http://bmjopen.bmj.com/site/about/guidelines.xhtml

~~11~~12- Hafez R, Bashour H, Abdulsalam A. Childbirth and its discontent: Experiences of Syrian Women. A paper presented at the ~~Annaul~~Annual meeting of Reproductive Health Working Group (RHWG) ~~Annual Meeting~~, Lebanon, July 11-13, 2004.

~~15 16~~ 13- Meakin R and Weinman J. **The Medical Interview Satisfaction Scale (MISS-21) adapted for British general practice**. Fam Pract 2002; **19**:257-263.

~~17 18~~ 14- Sholkamy H, Khalil K, Cherine M, Elnoury A, Breebaart M, Hassanein N. An observation checklist for facility-based normal labour and delivery practices: The Galaa Study. Monographs in Reproductive Health No. 5. Cairo; The Population Council, 2003.

~~12~~~~13~~15- Hussey A. and Hughes J.P. **Design and Analysis of stepped wedge cluster randomized trials**. Contemp Clin Trials 2007; **28**:182-191.

~~13~~~~14~~ 16- Brown CA and Lilford RJ. **The stepped wedge trial design: a systematic review**. BMC Med Res Methodol 2006, **6**:54.

~~14~~~~15~~- 17 Syrian Ministry of Health. www.moh.gov.sy

~~1516- Meakin R and Weinman J. **The Medical Interview Satisfaction Scale (MISS-21) adapted for British general practice**. Fam Pract 2002; **19**:257-263.~~

~~16~~178- Holloway S, Porteous M, Cetnarskyj R, Anderson E, Rush R, Fry A, Gorman D, Steel M, Campbell H. 2004. **Patient satisfaction with two different models of cancer genetic services in south-east Scotland**. *Br J Cancer* **90**: 582–589.

~~1718- Sholkamy H, Khalil K, Cherine M, Elnoury A, Breebaart M, Hassanein N. An observation checklist for facility-based normal labour and delivery practices: The Galaa Study. Monographs in Reproductive Health No. 5. Cairo; The Population Council, 2003.~~

~~18~~19- Moher D, Hopewell S, Schulz KF, Montori V, Gøtzsche PC, Devereaux PJ, Elbourne D, Egger M, Altman DG, for the CONSORT Group. CONSORT 2010 Explanation and Elaboration: updated guidelines for reporting parallel group randomised trial. BMJ 2010;340:c869.

Formatted: Font: (Default) Times New Roman, Not Bold, Font color: Auto, Strikethrough

Formatted: Font: (Default) Times New Roman, Not Bold, Font color: Auto, Strikethrough

Formatted: Font: (Default) Times New Roman, Not Bold, Font color: Auto, Strikethrough

Formatted: Font: (Default) Times New Roman, Not Bold, Font color: Auto, Strikethrough

Formatted: Font: (Default) Times New Roman, Not Bold, Font color: Auto, Strikethrough

Formatted: Font: (Default) Times New Roman, 12 pt, Not Bold

Formatted: Font: (Default) Times New Roman, 12 pt, Not Bold, No underline, Font color: Auto

Formatted: Font: (Default) Times New Roman, 12 pt, Not Bold, No underline

For peer review only

For peer review only - http://bmjopen.bmj.com/site/about/guidelines.xhtml

20- Maguire P and Pitceathly C. **Key communication skills and how to acquire them**. BMJ 2002; **325**:697-700.

19201- Brown JB, Boles M, Mullooly JP, Levinson W. **Effect of clinician communication skills training on patient satisfaction. A randomized, controlled trial**. Ann Intern Med 1999; **131**:822–9.

20212- Abdulasalam A. **Communication Skills! What does it mean to doctors and women: A qualitative study in Damascus maternity teaching hospitals**. Damascus University Journal for Health Sciences 2009; **25**:189-203. (In Arabic)

21223- Berridge E, Mackintosh N and Freeth D. **Supporting patient safety: examining communication within delivery suite teams through contrasting approaches to research observation**. Midwifery 2010; **26**: 512-519.

22234- Bingham D. **Helping hospitals change. Part 2: Childbirth education techniques to empower women**. J Perinatal Edu 2005; **14**: 51-55.

23245- Choices and Challenges in Changing Childbirth Research Network. **Routines in facility-based maternity care: evidence from the Arab World**. BJOG: an International Journal of Obstetrics and Gynaecology 2005; **112**, 1270-1276.

24256- Audit Commission. What seems to be the matter: Communication between hospitals and patients. HMSO, London. 1993.

For peer review only

For peer review only - http://bmjopen.bmj.com/site/about/guidelines.xhtml

Figure Legends:

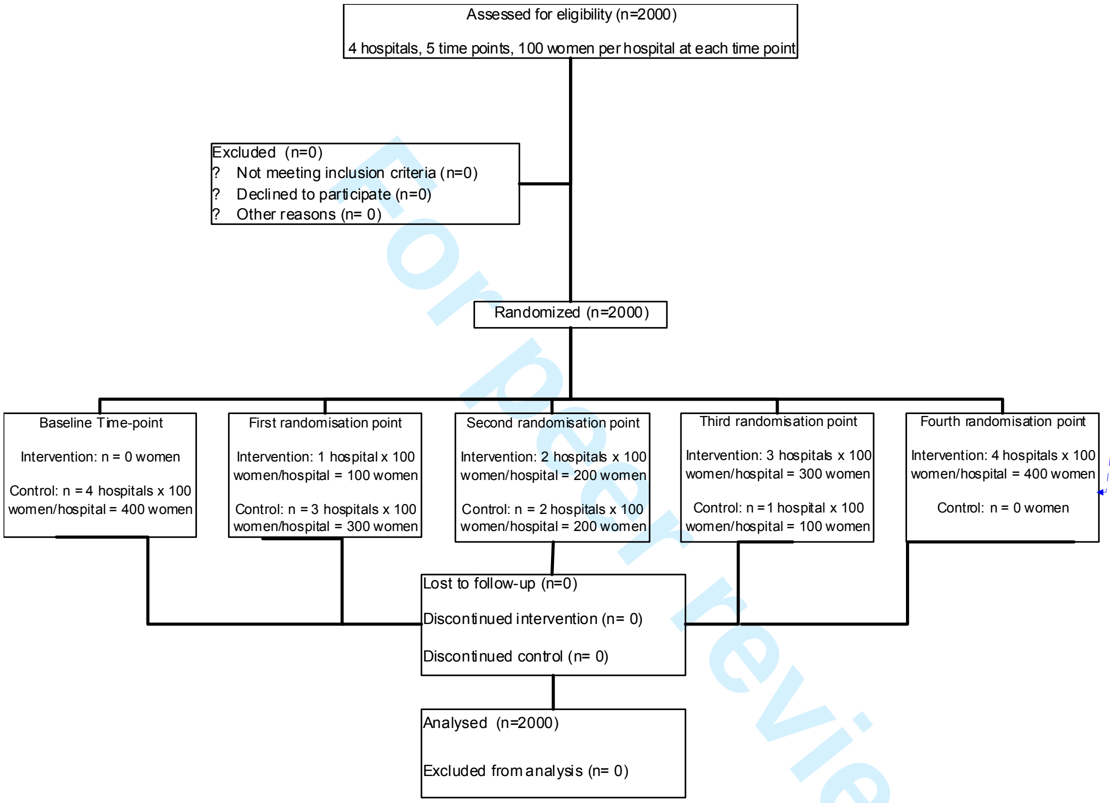

Formatted: No underline, Font color: Auto

Formatted: Keep with next

Figure 1: Flow of Participants

Formatted: Caption, Line spacing: single

Tables:

For peer review only - http://bmjopen.bmj.com/site/about/guidelines.xhtml

Table 1: Demographic Characteristics of the women across the time periods of the study[1]

	1	2
N	400	400
Mean age (SD)	24.79 (5.71)	25.12 (6.11)
Median age (Q1, Q3)*	24 (20, 28)	24 (20, 29)
Occupation: Housewife	377 (94)	377 (94)
Education		
Illiterate/Read and Write	53 (13)	51 (13)
Primary	291 (74)	306 (77)
High School +	51 (13)	42 (11)
Mean husband's age (SD)	30.98 (6.89)	31.19 (7.18)
Median husband's age (Q1, Q3)	30 (26, 35)	30 (26, 35)
Husband's Occupation		
Labourer (manual worker)	152 (39)	131 (33)
Clerk	72 (18)	71 (18)
(Self-employed)	144 (37)	163 (41)
Other	24 (6)	33 (8)
Husband's Education		
Illiterate/Read and Write	55 (14)	48 (12)
Primary	299 (76)	295 (74)

Formatted: Line spacing: 1.5 lines

Formatted Table

Formatted Table

For peer review only

For peer review only - http://bmjopen.bmj.com/site/about/guidelines.xhtml

~~High School +~~	~~41 (10)~~	~~53 (13)~~
~~Home Ownership~~		
~~Owns home~~	~~192 (49)~~	~~197 (49)~~
~~Share~~	~~201 (51)~~	~~202 (51)~~
~~Parity: Nulliparous~~	~~100 (25)~~	~~120 (30)~~
~~New born gender: Male~~	~~215 (56)~~	~~193 (49)~~

Table 1: Demographic characteristics of the women by intervention arm. Numbers represent counts (percentage) unless otherwise stated.

	Control N = 1000	Intervention N = 1000		Control N = 1000	Intervention N = 1000
Mean age (SD)	25.02 (6.11)	25.10 (5.89)	Woman's Education		
Occupation: Housewife	949 (95)	953 (95)	Illiterate/Read and Write	140 (14)	102 (10)
Home Ownership: Share	488 (49)	470 (48)	Primary	745 (74)	761 (77)
Parity: Nulliparous	266 (27)	263 (27)	High School +	110 (11)	128 (13)
New born gender: Male	507 (52)	480 (49)	Husband's Education		
Mean husband's age (SD)	30.90 (6.91)	30.85 (6.62)	Illiterate/Read and Write	113 (11)	101 (10)
Husband's Occupation			Primary	781 (79)	743 (76)
Labourer (manual worker)	359 (36)	337 (34)	High School +	100 (10)	135 (14)
Clerk	160 (16)	198 (20)			
Self-employed	391 (40)	432 (44)			

Formatted: Font: 10 pt, No underline, Font color: Auto

Formatted: Line spacing: single

Formatted: Font: 10 pt

Formatted Table

For peer review only

Other	80 (8)	21 (2)			

~~* Q1 is the first quartile and Q3 is the third quartile.~~

~~! Numbers represent counts (percentage) unless otherwise stated.~~

For peer review only

For peer review only - http://bmjopen.bmj.com/site/about/guidelines.xhtml

Table 2: Numbers (and percentages) of women who agreed/strongly agreed with various questions concerning their labour/delivery experience. The first two columns give Tthe numbers comparing compare all the intervention clusters to the control clusters; the other columns give the corresponding numbers stratified by hospital.*

Item	Control Period N=1000	Intervention Period N=1000
	All	All
N	1000	1000
Overall satisfaction with doctors' communication skills during labour/delivery	619 (62)	713 (72)
Did the doctors identify themselves?	132 (13)	118 (12)
Did the doctor greet you?	208 (21)	323 (32)
Did the doctor look at you when talking to you?	434 (43)	599 (60)
Did the doctor show interest in you as a person?	393 (39)	329 (33)
Did the doctor insult you?	55 (6)	29 (3)
Did the doctor use humour to comfort you?	78 (8)	118 (12)
Did the doctor use any terms to calm you down?	363 (36)	419 (42)
Did the doctor use his/her hands to assist you or to reassure you?	314 (32)	409 (42)
Did the doctor shout or scream at you?	125 (13)	71 (7)
Did the doctor listen to you with concern and without interrupting you?	413 (46)	551 (58)
Was the doctor engaged in other issues, so you felt being unattended and annoyed?	165 (17)	67 (7)
Did you feel let down by the way he/she was dealing with you?	66 (7)	31 (3)
Did the doctor explain all the steps before doing the clinical exam?	249 (25)	278 (29)

Item	Control Period N=1000	Intervention Period N=1000
Did the doctor listen to you with concern and without interrupting you?	413 (46)	551 (58)
Was the doctor engaged in other issues, so you felt being unattended and annoyed?	165 (17)	67 (7)
Did you feel let down by the way he/she was dealing with you?	66 (7)	31 (3)
Did the doctor explain all the steps before doing the clinical exam?	249 (25)	278 (29)
Did the doctor explain the findings from the clinical exam?	326 (34)	399 (41)
Were you annoyed by the doctor explaining all the findings?	40 (5)	73 (10)
Did the doctor explain all the alternative choices to you?	176 (23)	145 (17)
Did the doctor help you in making decisions?	175 (29)	158 (23)
Was the doctor annoyed from your questions and avoided answering them?	122 (14)	54 (6)
Did the doctor use medical terms in explanation, thus they were not understood?	77 (8)	48 (5)
Did the doctor give you conflicting medical information?	31 (3)	12 (1)

33

Formatted ... [1]
Formatted ... [4]
Formatted ... [2]
Formatted ... [5]
Formatted ... [6]
Formatted Table ... [3]
Formatted ... [7]
Formatted ... [8]
Formatted ... [9]
Formatted ... [10]
Formatted Table ... [11]
Formatted ... [12]
Formatted ... [14]
Formatted ... [13]
Formatted ... [15]
Formatted ... [16]
Formatted ... [17]
Formatted ... [19]
Formatted ... [18]
Formatted ... [20]
Formatted ... [21]
Formatted ... [22]
Formatted ... [24]
Formatted ... [23]
Formatted ... [25]
Formatted ... [26]
Formatted ... [27]
Formatted ... [29]
Formatted ... [28]
Formatted ... [30]
Formatted ... [31]
Formatted ... [32]
Formatted ... [34]
Formatted ... [33]
Formatted ... [35]
Formatted ... [36]
Formatted ... [37]
Formatted ... [39]
Formatted ... [38]
Formatted ... [40]
Formatted ... [41]
Formatted ... [42]
Formatted ... [44]
Formatted ... [43]
Formatted ... [45]
Formatted ... [46]
Formatted ... [47]
Formatted ... [49]
Formatted ... [48]
Formatted ... [50]
Formatted ... [51]
Formatted ... [52]
Formatted ... [54]
Formatted ... [53]
Formatted ... [55]
Formatted ... [56]
Formatted ... [57]
Formatted ... [59]
Formatted ... [58]
Formatted ... [60]

For peer review only

~~Did the doctor explain the findings from the clinical exam?~~	~~326 (34)~~	~~399 (41)~~
~~Were you annoyed by the doctor explaining all the findings?~~	~~40 (5)~~	~~73 (10)~~
~~Did the doctor explain all the alternative choices to you?~~	~~176 (23)~~	~~145 (17)~~
~~Did the doctor help you in making decisions?~~	~~175 (29)~~	~~158 (23)~~
~~Was the doctor annoyed from your questions and avoided answering them?~~	~~122 (14)~~	~~54 (6)~~
~~Did the doctor use medical terms in explanation, thus they were not understood?~~	~~77 (8)~~	~~48 (5)~~
~~Did the doctor give you conflicting medical information?~~	~~31 (3)~~	~~12 (1)~~

* Due to missing information some of the percentages do not tie up with the total number given.

Formatted: Line spacing: single

Formatted: Line spacing: single

Formatted: Line spacing: single

Formatted: Line spacing: single

Formatted: Line spacing: single

Formatted: Space After: 0 pt, Line spacing: single

Formatted: Line spacing: single

Formatted: Line spacing: single

For peer review only

For peer review only - http://bmjopen.bmj.com/site/about/guidelines.xhtml

Table 3: Summary statistics for the outcome of women satisfaction computed as the average score and Aaverrage Score score for Complete MMISS . The first two columns give the numbers comparing all the intervention clusters to the control clusters; the other columns give the corresponding numbers stratified by hospital

	Control Period	Intervention Period
	All	All
N	1000	1000
Mean (SD)	3.23 (0.72)	3.42 (0.73)
Median (Q1, Q3)	3.3 (2.8,3.7)	3.3 (3.0, 4.1)
N	472	498
Mean (SD)	3.43 (0.71)	3.54 (0.66)
Median (Q1, Q3)	3.4 (3.0,3.9)	3.3 (3.1, 4.1)

	Control Period	Intervention Period
	All	All
N	1000	1000
Mean (SD)	3.23 (0.72)	3.42 (0.73)
Median (Q1, Q3)	3.3 (2.8,3.7)	3.3 (3.0, 4.1)
N	472	498
Mean (SD)	3.43 (0.71)	3.54 (0.66)
Median (Q1, Q3)	3.4 (3.0,3.9)	3.3 (3.1, 4.1)

Complete MMISS.

Formatted ... [70]
Formatted ... [71]
Formatted ... [127]
Formatted ... [128]
Formatted ... [72]
Formatted ... [73]
Formatted ... [75]
Formatted ... [77]
Formatted ... [74]
Formatted ... [76]
Formatted ... [129]
Formatted ... [136]
Formatted Table ... [131]
Formatted ... [132]
Formatted ... [134]
Formatted ... [130]
Formatted ... [133]
Formatted ... [78]
Formatted ... [135]
Formatted ... [79]
Formatted ... [81]
Formatted ... [80]
Formatted ... [82]
Formatted ... [83]
Formatted ... [84]
Formatted ... [137]
Formatted ... [85]
Formatted ... [86]
Formatted ... [87]
Formatted ... [88]
Formatted ... [89]
Formatted ... [138]
Formatted ... [140]
Formatted ... [142]
Formatted ... [139]
Formatted ... [143]
Formatted ... [141]
Formatted ... [90]
Formatted ... [91]
Formatted ... [92]
Formatted ... [94]
Formatted ... [96]
Formatted ... [144]
Formatted ... [145]
Formatted ... [146]
Formatted ... [147]
Formatted ... [148]
Formatted ... [93]
Formatted ... [95]
Formatted ... [97]
Formatted ... [149]
Formatted ... [150]
Formatted ... [151]
Formatted ... [153]
Formatted ... [155]
Formatted ... [98]
Formatted ... [99]
Formatted ... [100]
Formatted ... [102]
Formatted ... [104]

For peer review only - http://bmjopen.bmj.com/site/about/guidelines.xhtml

~~Table 4:~~ Regression analysis estimates for the average score and average score for complete MMISS on intervention and time controlling for clustering by hospital using ~~three estimation procedures LMM,~~ GLAMM ~~and GEE~~. Estimates are presented together with 95% confidence interval (CI) limits as subscripts ($_{Lower}Est_{Upper}$).

	Average Score	Average Score Complete MMISS
Control: Median (Q1, Q3)	3.3 (2.8, 3.7)	3.4 (3.0, 3.9)
Intervention: Median (Q1, Q3)	3.3 (3.0, 4.1)	3.3 (3.1, 4.1)
GLMM Estimates	$_{Lower}Est_{Upper}$	$_{Lower}Est_{Upper}$
**Fixed-effects**		
intervention	$_{-0.08}0.03_{0.15}$	$_{-0.29}-0.13_{0.04}$
time: 2	$_{-0.08}0.02_{0.12}$	$_{-0.10}0.05_{0.19}$
time: 3	$_{-0.14}-0.03_{0.08}$	$_{-0.02}0.12_{0.27}$
time: 4	$_{-0.08}0.05_{0.18}$	$_{0.12}0.30_{0.49}$
time: 5	$_{-0.15}0.01_{0.16}$	$_{-0.03}0.17_{0.37}$
constant	$_{3.16}3.32_{3.48}$	$_{3.23}3.44_{3.64}$
**Random-effects**	Variance (SE)	Variance (SE)
**Hospital**	0. 02 (0.016)	0.03 (0.026)
**Residual**	0.51 (0.016)	0.45 (0.02)

Formatted: Font: 10 pt, No underline, Font color: Auto
Formatted: Justified, Line spacing: single
Formatted: Font: 10 pt
Formatted: Don't add space between paragraphs of the same style, Line spacing: single
Formatted Table
Formatted: Don't add space between paragraphs of the same style, Line spacing: single
Formatted: Don't add space between paragraphs of the same style, Line spacing: single
Formatted: Don't add space between paragraphs of the same style, Line spacing: single
Formatted: Don't add space between paragraphs of the same style, Line spacing: single
Formatted: Centered, Don't add space between paragraphs of the same style, Line spacing: single
Formatted Table
Formatted: Centered, Don't add space between paragraphs of the same style, Line spacing: single
Formatted: Don't add space between paragraphs of the same style, Line spacing: single
Formatted: Centered, Space Before: 0 pt, After: 0 pt, Don't add space between paragraphs of the same style, Line spacing: single
Formatted: Centered, Don't add space between paragraphs of the same style, Line spacing: single
Formatted: Don't add space between paragraphs of the same style, Line spacing: single
Formatted ... [187]
Formatted ... [188]
Formatted ... [189]
Formatted ... [190]
Formatted ... [191]
Formatted ... [192]
Formatted ... [193]
Formatted ... [194]
Formatted ... [196]
Formatted ... [195]
Formatted ... [197]
Formatted ... [198]
Formatted ... [199]
Formatted ... [200]
Formatted ... [201]

For peer review only

For peer review only - http://bmjopen.bmj.com/site/about/guidelines.xhtml

Table 54: Numbers (and percentages) of "No"negative responses for items on the observational checklist concerning communication between doctor and woman during labour/delivery, unless otherwise stated. Due to missing information some of the percentages do not tie up with the total number given. The first two columns give compare the numbers comparing all the intervention clusters to the control clusters.; the other columns give the corresponding numbers stratified by hospital.

	Control Period N = 565	Intervention Period N=631
Hospital	All	All
N	565	631
Mean (SD) of the average score	0.34 (0.13)	0.39 (0.10)
Mean (SD) of the average score for complete checklist	0.34 (0.10)	0.39 (0.09)
Checklist Item		
1. Did the doctor identify himself/herself?	539 (100)	621 (99)
2. Did the doctor call the woman by her name?	132 (24)	99 — (16)
3. Did the doctor take the woman's permission to examine her?	326 (61)	305 (49)
4. Did the doctor explain the plan he/she will follow?	497 (92)	577 (92)
Before starting the vaginal examination, did the doctor		
5. take her permission for the exam?	335 (62)	262 (42)
6. close the door?	472 (88)	568 (91)
7. cover the woman during the exam?	315 (59)	116 (18)
8. ask the woman to bend her knees?	210 (39)	79 (13)
After completing the vaginal examination, did the doctor		
9. tell the woman the results of the exam?	418 (78)	504 (81)
10. explain the next steps to be followed?	486 (91)	586 (94)
11. relay the findings to other team members?	241 (45)	102 (16)
12. relay the findings to the midwives/nurses?	393 (80)	579 (93)
13. Did the doctor give instructions about eating and drinking?	463 (87)	516 (83)
14. Did the doctor give instructions about movement?	335 (63)	428 (70)
15. Was the woman asked any questions at this stage?	168 (32)	94 (15)
16. Did the woman ask any questions at this stage?	292 (56)	281 (46)
17. Did the doctor respond to her queries?	275 (63)	342 (64)
18. Was the doctor responsive to the woman's pain?	285 (59)	333 (57)

	Control Period N=565	Intervention Period N=631
Checklist Item		
Was the woman asked any questions at this stage?	168 (32)	94 (15)
Did the woman ask any questions at this stage?	292 (56)	281 (46)
Did the doctor respond to her queries?	275 (63)	342 (64)
Was the doctor responsive to the woman's pain?	285 (59)	333 (57)
Was the woman encouraged at this stage?	186 (40)	239 (42)
Was the woman told about the proximity of labour?	242 (55)	363 (66)
Was the woman told about the stages of labour?	399 (95)	533 (98)
Was the woman told about her role during labour?	347 (83)	508 (94)
During delivery, was the woman asked to push?	26 (6)	23 (4)
Did they explain to the woman when and how to push?	237 (57)	230 (42)
Did the woman ask any questions at this stage?	284 (68)	296 (55)
Did the doctor respond to her queries?	264 (77)	330 (72)
Did the doctor inform the mother about her baby's status?	307 (75)	386 (72)
Was the woman congratulated after the delivery?	217 (53)	214 (40)
Was the woman instructed about the process after the delivery?	392 (95)	515 (96)
Was the woman instructed about how to care for her newborn?	409 (99)	534 (99)
Was the woman reassured that she is going to be fine?	373 (90)	511 (95)

Formatted ... [202]
Formatted ... [205]
Formatted ... [203]
Formatted ... [204]
Formatted ... [206]
Formatted ... [207]
Formatted ... [208]
Formatted Table ... [209]
Formatted ... [210]
Formatted ... [211]
Formatted ... [212]
Formatted ... [213]
Formatted ... [214]
Formatted ... [215]
Formatted ... [216]
Formatted ... [217]
Formatted ... [218]
Formatted ... [219]
Formatted ... [220]
Formatted ... [221]
Formatted ... [222]
Formatted ... [223]
Formatted ... [224]
Formatted ... [225]
Formatted ... [226]
Formatted ... [227]
Formatted ... [228]
Formatted ... [229]
Formatted ... [230]
Formatted ... [231]
Formatted ... [232]
Formatted ... [233]
Formatted ... [234]
Formatted ... [235]
Formatted ... [236]
Formatted ... [237]
Formatted ... [238]
Formatted ... [239]
Formatted ... [240]
Formatted ... [241]
Formatted ... [242]
Formatted ... [243]
Formatted ... [244]
Formatted ... [245]
Formatted ... [246]
Formatted ... [247]
Formatted ... [248]
Formatted ... [249]
Formatted ... [250]
Formatted ... [251]

For peer review only - http://bmjopen.bmj.com/site/about/guidelines.xhtml

19. Was the woman encouraged at this stage?	186 (40)	239 (42)
20. Was the woman told about the proximity of labour?	242 (55)	363 (66)
21. Was the woman told about the stages of labour?	399 (95)	533 (98)
22. Was the woman told about her role during labour?	347 (83)	508 (94)
23. During delivery, was the woman asked to push?	26 (6)	23 (4)
24. Did they explain to the woman when and how to push?	237 (57)	230 (42)
25. Did the woman ask any questions at this stage?	284 (68)	296 (55)
26. Did the doctor respond to her queries?	264 (77)	330 (72)
27. Did the doctor inform the mother about her baby's status?	307 (75)	386 (72)
28. Was the woman congratulated after the delivery?	217 (53)	214 (40)
29. Was the woman instructed about the process after the delivery?	392 (95)	515 (96)
30. Was the woman instructed about how to care for her newborn?	409 (99)	534 (99)
31. Was the woman reassured the she is going to be fine?	373 (90)	511 (95)
Mean (SD) of the average score	0.34 (0.13)	0.39 (0.10)
Mean (SD) of the average score for complete checklist	0.34 (0.10)	0.39 (0.09)

Formatted: Line spacing: single
Formatted: Line spacing: single
Formatted: Line spacing: single
Formatted: Line spacing: single
Formatted: Line spacing: single
Formatted: Line spacing: single
Formatted: Line spacing: single
Formatted: Line spacing: single
Formatted: Line spacing: single
Formatted: Line spacing: single
Formatted: Line spacing: single
Formatted: Line spacing: single
Formatted: Line spacing: single
Formatted: Line spacing: single
Formatted: Line spacing: single

For peer review only

For peer review only - http://bmjopen.bmj.com/site/about/guidelines.xhtml

Appendix:

Figure 2 gives the distribution of the average score by treatment arm.

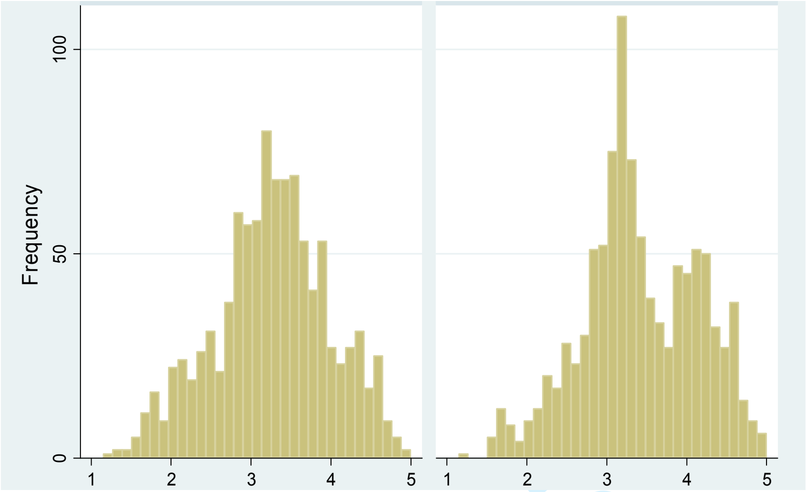

(a) (b)

Figure 2: Distribution of the average score by treatment arm (a) Control and (b) Intervention.

Description of ~~additional~~ supporting files

Additional File one: ~~Schema~~ Diagram **of the study design**

Additional File two: **A graphical representation of the overall satisfaction outcome score of the women stratified by hospital and time of intervention. Data presented are for complete MMISS only**

Additional File three: **Results by intervention period.**

Additional File four**: Trial Protocol.**

39

Formatted: Font: (Default) Times New Roman, 12 pt, Not Bold, No underline, Font color: Auto

Formatted: Font: (Default) Times New Roman, 12 pt, Not Bold

Formatted: Font: (Default) Arial, 16 pt, Bold, No underline, Font color: Auto

Formatted: Centered, Keep with next

Formatted: Caption, Centered, Line spacing: single

Formatted: Font: Bold

Formatted: Font: 12 pt, Bold, No underline, Font color: Auto

Formatted: Font: Bold

Formatted: Font: 12 pt, Bold, No underline, Font color: Auto

Formatted: Font: Bold

For peer review only

For peer review only - http://bmjopen.bmj.com/site/about/guidelines.xhtml

Page 33: [1] Formatted	Mona Kanaan	5/26/2013 7:23:00 AM

Font: 10 pt, No underline, Font color: Auto

Page 33: [2] Formatted	Mona Kanaan	5/25/2013 8:44:00 AM

Line spacing: single

Page 33: [3] Formatted Table	Mona Kanaan	5/25/2013 8:44:00 AM

Formatted Table

Page 33: [4] Formatted	Mona Kanaan	5/26/2013 7:23:00 AM

Font: 10 pt

Page 33: [5] Formatted	Mona Kanaan	5/26/2013 7:22:00 AM

Centered

Page 33: [6] Formatted	Mona Kanaan	5/25/2013 8:44:00 AM

Line spacing: single

Page 33: [7] Formatted	Mona Kanaan	5/26/2013 7:22:00 AM

Centered

Page 33: [8] Formatted	Mona Kanaan	5/25/2013 8:44:00 AM

Line spacing: single

Page 33: [9] Formatted	Mona Kanaan	5/25/2013 8:44:00 AM

Line spacing: single

Page 33: [10] Formatted	Mona Kanaan	5/25/2013 8:44:00 AM

Line spacing: single

Page 33: [11] Formatted Table	Mona Kanaan	5/25/2013 8:44:00 AM

Formatted Table

Page 33: [12] Formatted	Mona Kanaan	5/25/2013 8:45:00 AM

Line spacing: single

Page 33: [13] Formatted	Mona Kanaan	5/25/2013 8:45:00 AM

Centered

Page 33: [14] Formatted	Mona Kanaan	5/25/2013 8:45:00 AM

Line spacing: single

Page 33: [15] Formatted	Mona Kanaan	5/25/2013 8:45:00 AM

Centered

Page 33: [16] Formatted	Mona Kanaan	5/25/2013 8:44:00 AM

Line spacing: single

Page 33: [17] Formatted	Mona Kanaan	5/25/2013 8:45:00 AM

Line spacing: single

Page 33: [18] Formatted	Mona Kanaan	5/25/2013 8:45:00 AM

Centered

Page 33: [19] Formatted	Mona Kanaan	5/25/2013 8:45:00 AM

Line spacing: single

Page 33: [20] Formatted	Mona Kanaan	5/25/2013 8:45:00 AM

Centered

Page 33: [21] Formatted	Mona Kanaan	5/25/2013 8:44:00 AM

Line spacing: single

For peer review only

Page 33: [22] Formatted	Mona Kanaan	5/25/2013 8:45:00 AM

Line spacing: single

Page 33: [23] Formatted	Mona Kanaan	5/25/2013 8:45:00 AM

Centered

Page 33: [24] Formatted	Mona Kanaan	5/25/2013 8:45:00 AM

Line spacing: single

Page 33: [25] Formatted	Mona Kanaan	5/25/2013 8:45:00 AM

Centered

Page 33: [26] Formatted	Mona Kanaan	5/25/2013 8:44:00 AM

Line spacing: single

Page 33: [27] Formatted	Mona Kanaan	5/25/2013 8:45:00 AM

Line spacing: single

Page 33: [28] Formatted	Mona Kanaan	5/25/2013 8:45:00 AM

Centered

Page 33: [29] Formatted	Mona Kanaan	5/25/2013 8:45:00 AM

Line spacing: single

Page 33: [30] Formatted	Mona Kanaan	5/25/2013 8:45:00 AM

Centered

Page 33: [31] Formatted	Mona Kanaan	5/25/2013 8:44:00 AM

Line spacing: single

Page 33: [32] Formatted	Mona Kanaan	5/25/2013 8:45:00 AM

Space After: 0 pt, Line spacing: single

Page 33: [33] Formatted	Mona Kanaan	5/25/2013 8:45:00 AM

Centered, Space Before: 0 pt, After: 0 pt

Page 33: [34] Formatted	Mona Kanaan	5/25/2013 8:45:00 AM

Space After: 0 pt, Line spacing: single

Page 33: [35] Formatted	Mona Kanaan	5/25/2013 8:45:00 AM

Centered, Space Before: 0 pt, After: 0 pt

Page 33: [36] Formatted	Mona Kanaan	5/25/2013 8:44:00 AM

Line spacing: single

Page 33: [37] Formatted	Mona Kanaan	5/25/2013 8:45:00 AM

Line spacing: single

Page 33: [38] Formatted	Mona Kanaan	5/25/2013 8:45:00 AM

Centered

Page 33: [39] Formatted	Mona Kanaan	5/25/2013 8:45:00 AM

Line spacing: single

Page 33: [40] Formatted	Mona Kanaan	5/25/2013 8:45:00 AM

Centered

Page 33: [41] Formatted	Mona Kanaan	5/25/2013 8:44:00 AM

Line spacing: single

Page 33: [42] Formatted	Mona Kanaan	5/25/2013 8:45:00 AM

Line spacing: single

For peer review only

Page 33: [43] Formatted	Mona Kanaan	5/25/2013 8:45:00 AM

Centered

Page 33: [44] Formatted	Mona Kanaan	5/25/2013 8:45:00 AM

Line spacing: single

Page 33: [45] Formatted	Mona Kanaan	5/25/2013 8:45:00 AM

Centered

Page 33: [46] Formatted	Mona Kanaan	5/25/2013 8:44:00 AM

Line spacing: single

Page 33: [47] Formatted	Mona Kanaan	5/25/2013 8:45:00 AM

Line spacing: single

Page 33: [48] Formatted	Mona Kanaan	5/25/2013 8:45:00 AM

Centered

Page 33: [49] Formatted	Mona Kanaan	5/25/2013 8:45:00 AM

Line spacing: single

Page 33: [50] Formatted	Mona Kanaan	5/25/2013 8:45:00 AM

Centered

Page 33: [51] Formatted	Mona Kanaan	5/25/2013 8:44:00 AM

Line spacing: single

Page 33: [52] Formatted	Mona Kanaan	5/25/2013 8:45:00 AM

Line spacing: single

Page 33: [53] Formatted	Mona Kanaan	5/25/2013 8:45:00 AM

Centered

Page 33: [54] Formatted	Mona Kanaan	5/25/2013 8:45:00 AM

Line spacing: single

Page 33: [55] Formatted	Mona Kanaan	5/25/2013 8:45:00 AM

Centered

Page 33: [56] Formatted	Mona Kanaan	5/25/2013 8:44:00 AM

Line spacing: single

Page 33: [57] Formatted	Mona Kanaan	5/25/2013 8:45:00 AM

Line spacing: single

Page 33: [58] Formatted	Mona Kanaan	5/25/2013 8:45:00 AM

Centered

Page 33: [59] Formatted	Mona Kanaan	5/25/2013 8:45:00 AM

Line spacing: single

Page 33: [60] Formatted	Mona Kanaan	5/25/2013 8:45:00 AM

Centered

Page 33: [61] Formatted	Mona Kanaan	5/25/2013 8:44:00 AM

Line spacing: single

Page 33: [62] Formatted	Mona Kanaan	5/25/2013 8:44:00 AM

Space Before: 0 pt, After: 0 pt

Page 33: [63] Formatted	Mona Kanaan	5/25/2013 8:44:00 AM

Space After: 0 pt, Line spacing: single

For peer review only

Page 33: [64] Formatted	Mona Kanaan	5/25/2013 8:44:00 AM

Space Before: 0 pt, After: 0 pt

Page 33: [65] Formatted	Mona Kanaan	5/25/2013 8:44:00 AM

Space After: 0 pt, Line spacing: single

Page 33: [66] Formatted	Mona Kanaan	5/25/2013 8:44:00 AM

Space Before: 0 pt, After: 0 pt

Page 33: [67] Formatted	Mona Kanaan	5/25/2013 8:44:00 AM

Line spacing: single

Page 33: [68] Formatted	Mona Kanaan	5/25/2013 8:44:00 AM

Line spacing: single

Page 33: [69] Formatted	Mona Kanaan	5/25/2013 8:44:00 AM

Line spacing: single

Page 35: [70] Formatted	Mona Kanaan	5/26/2013 9:18:00 AM

Space Before: 0 pt, After: 0 pt, Line spacing: single

Page 35: [71] Formatted	Mona Kanaan	5/26/2013 9:17:00 AM

Font: 10 pt, No underline, Font color: Auto

Page 35: [71] Formatted	Mona Kanaan	5/26/2013 9:17:00 AM

Font: 10 pt, No underline, Font color: Auto

Page 35: [71] Formatted	Mona Kanaan	5/26/2013 9:17:00 AM

Font: 10 pt, No underline, Font color: Auto

Page 35: [71] Formatted	Mona Kanaan	5/26/2013 9:17:00 AM

Font: 10 pt, No underline, Font color: Auto

Page 35: [71] Formatted	Mona Kanaan	5/26/2013 9:17:00 AM

Font: 10 pt, No underline, Font color: Auto

Page 35: [72] Formatted	Mona Kanaan	5/26/2013 9:17:00 AM

Font: 10 pt

Page 35: [73] Formatted	Mona Kanaan	5/26/2013 9:17:00 AM

Font: 10 pt, No underline, Font color: Auto

Page 35: [74] Formatted	Mona Kanaan	5/26/2013 9:18:00 AM

Left, Space Before: 0 pt, After: 0 pt, Line spacing: single

Page 35: [75] Formatted	Mona Kanaan	5/26/2013 9:17:00 AM

Font: 10 pt

Page 35: [76] Formatted	Mona Kanaan	5/26/2013 9:17:00 AM

Font: 10 pt, No underline, Font color: Auto

Page 35: [76] Formatted	Mona Kanaan	5/26/2013 9:17:00 AM

Font: 10 pt, No underline, Font color: Auto

Page 35: [77] Formatted	Mona Kanaan	5/26/2013 9:17:00 AM

Font: 10 pt, No underline, Font color: Auto

Page 35: [77] Formatted	Mona Kanaan	5/26/2013 9:17:00 AM

Font: 10 pt, No underline, Font color: Auto

Page 35: [78] Formatted	Mona Kanaan	5/26/2013 9:17:00 AM

Font: 10 pt, No underline, Font color: Auto

For peer review only

Page 35: [78] Formatted	Mona Kanaan	5/26/2013 9:17:00 AM

Font: 10 pt, No underline, Font color: Auto

Page 35: [79] Formatted	Mona Kanaan	5/26/2013 9:17:00 AM

Font: 10 pt

Page 35: [80] Formatted	Mona Kanaan	5/26/2013 9:18:00 AM

Space Before: 0 pt, After: 0 pt, Line spacing: single

Page 35: [81] Formatted	Mona Kanaan	5/26/2013 9:17:00 AM

Font: 10 pt, No underline, Font color: Auto

Page 35: [82] Formatted	Mona Kanaan	5/26/2013 9:18:00 AM

Left, Space Before: 0 pt, After: 0 pt, Line spacing: single

Page 35: [83] Formatted	Mona Kanaan	5/26/2013 9:17:00 AM

Font: 10 pt

Page 35: [84] Formatted	Mona Kanaan	5/26/2013 9:17:00 AM

Font: 10 pt, No underline, Font color: Auto

Page 35: [84] Formatted	Mona Kanaan	5/26/2013 9:17:00 AM

Font: 10 pt, No underline, Font color: Auto

Page 35: [85] Formatted	Mona Kanaan	5/26/2013 9:17:00 AM

Font: 10 pt, No underline, Font color: Auto

Page 35: [86] Formatted	Mona Kanaan	5/26/2013 9:18:00 AM

Left, Space Before: 0 pt, After: 0 pt, Line spacing: single

Page 35: [87] Formatted	Mona Kanaan	5/26/2013 9:17:00 AM

Font: 10 pt

Page 35: [88] Formatted	Mona Kanaan	5/26/2013 9:17:00 AM

Font: 10 pt, No underline

Page 35: [88] Formatted	Mona Kanaan	5/26/2013 9:17:00 AM

Font: 10 pt, No underline

Page 35: [89] Formatted	Mona Kanaan	5/26/2013 9:17:00 AM

Font: 10 pt, No underline

Page 35: [89] Formatted	Mona Kanaan	5/26/2013 9:17:00 AM

Font: 10 pt, No underline

Page 35: [90] Formatted	Mona Kanaan	5/26/2013 9:17:00 AM

Font: 10 pt, No underline, Font color: Auto

Page 35: [91] Formatted	Mona Kanaan	5/26/2013 9:18:00 AM

Left, Space Before: 0 pt, After: 0 pt, Line spacing: single

Page 35: [92] Formatted	Mona Kanaan	5/26/2013 9:17:00 AM

Font: 10 pt

Page 35: [93] Formatted	Mona Kanaan	5/26/2013 9:17:00 AM

Font: 10 pt, No underline, Font color: Auto

Page 35: [93] Formatted	Mona Kanaan	5/26/2013 9:17:00 AM

Font: 10 pt, No underline, Font color: Auto

Page 35: [94] Formatted	Mona Kanaan	5/26/2013 9:17:00 AM

Font: 10 pt, No underline

For peer review only

Page 35: [94] Formatted	Mona Kanaan	5/26/2013 9:17:00 AM

Font: 10 pt, No underline

Page 35: [95] Formatted	Mona Kanaan	5/26/2013 9:17:00 AM

Font: 10 pt, No underline

Page 35: [95] Formatted	Mona Kanaan	5/26/2013 9:17:00 AM

Font: 10 pt, No underline

Page 35: [96] Formatted	Mona Kanaan	5/26/2013 9:17:00 AM

Font: 10 pt, No underline

Page 35: [96] Formatted	Mona Kanaan	5/26/2013 9:17:00 AM

Font: 10 pt, No underline

Page 35: [97] Formatted	Mona Kanaan	5/26/2013 9:17:00 AM

Font: 10 pt, No underline

Page 35: [97] Formatted	Mona Kanaan	5/26/2013 9:17:00 AM

Font: 10 pt, No underline

Page 35: [98] Formatted	Mona Kanaan	5/26/2013 9:17:00 AM

Font: 10 pt, No underline, Font color: Auto

Page 35: [99] Formatted	Mona Kanaan	5/26/2013 9:18:00 AM

Left, Space Before: 0 pt, After: 0 pt, Line spacing: single

Page 35: [100] Formatted	Mona Kanaan	5/26/2013 9:17:00 AM

Font: 10 pt

Page 35: [101] Formatted	Mona Kanaan	5/26/2013 9:17:00 AM

Font: 10 pt, No underline, Font color: Auto

Page 35: [101] Formatted	Mona Kanaan	5/26/2013 9:17:00 AM

Font: 10 pt, No underline, Font color: Auto

Page 35: [102] Formatted	Mona Kanaan	5/26/2013 9:17:00 AM

Font: 10 pt, No underline

Page 35: [102] Formatted	Mona Kanaan	5/26/2013 9:17:00 AM

Font: 10 pt, No underline

Page 35: [103] Formatted	Mona Kanaan	5/26/2013 9:17:00 AM

Font: 10 pt, No underline

Page 35: [103] Formatted	Mona Kanaan	5/26/2013 9:17:00 AM

Font: 10 pt, No underline

Page 35: [104] Formatted	Mona Kanaan	5/26/2013 9:17:00 AM

Font: 10 pt, No underline

Page 35: [104] Formatted	Mona Kanaan	5/26/2013 9:17:00 AM

Font: 10 pt, No underline

Page 35: [105] Formatted	Mona Kanaan	5/26/2013 9:17:00 AM

Font: 10 pt, No underline

Page 35: [105] Formatted	Mona Kanaan	5/26/2013 9:17:00 AM

Font: 10 pt, No underline

Page 35: [106] Formatted	Mona Kanaan	5/26/2013 9:17:00 AM

Font: 10 pt, No underline, Font color: Auto

For peer review only

Page 35: [107] Formatted	Mona Kanaan	5/26/2013 9:18:00 AM

Left, Space Before: 0 pt, After: 0 pt, Line spacing: single

Page 35: [108] Formatted	Mona Kanaan	5/26/2013 9:17:00 AM

Font: 10 pt

Page 35: [109] Formatted	Mona Kanaan	5/26/2013 9:17:00 AM

Font: 10 pt, No underline

Page 35: [109] Formatted	Mona Kanaan	5/26/2013 9:17:00 AM

Font: 10 pt, No underline

Page 35: [110] Formatted	Mona Kanaan	5/26/2013 9:17:00 AM

Font: 10 pt, No underline

Page 35: [110] Formatted	Mona Kanaan	5/26/2013 9:17:00 AM

Font: 10 pt, No underline

Page 35: [111] Formatted	Mona Kanaan	5/26/2013 9:17:00 AM

Font: 10 pt, No underline, Font color: Auto

Page 35: [112] Formatted	Mona Kanaan	5/26/2013 9:18:00 AM

Left, Space Before: 0 pt, After: 0 pt, Line spacing: single

Page 35: [113] Formatted	Mona Kanaan	5/26/2013 9:17:00 AM

Font: (Default) Times New Roman, 10 pt

Page 35: [114] Formatted	Mona Kanaan	5/26/2013 9:17:00 AM

Font: 10 pt, No underline, Font color: Auto

Page 35: [114] Formatted	Mona Kanaan	5/26/2013 9:17:00 AM

Font: 10 pt, No underline, Font color: Auto

Page 35: [115] Formatted	Mona Kanaan	5/26/2013 9:17:00 AM

Font: 10 pt, No underline

Page 35: [115] Formatted	Mona Kanaan	5/26/2013 9:17:00 AM

Font: 10 pt, No underline

Page 35: [116] Formatted	Mona Kanaan	5/26/2013 9:17:00 AM

Font: 10 pt, No underline

Page 35: [116] Formatted	Mona Kanaan	5/26/2013 9:17:00 AM

Font: 10 pt, No underline

Page 35: [117] Formatted	Mona Kanaan	5/26/2013 9:17:00 AM

Font: 10 pt, No underline

Page 35: [117] Formatted	Mona Kanaan	5/26/2013 9:17:00 AM

Font: 10 pt, No underline

Page 35: [118] Formatted	Mona Kanaan	5/26/2013 9:17:00 AM

Font: 10 pt, No underline

Page 35: [118] Formatted	Mona Kanaan	5/26/2013 9:17:00 AM

Font: 10 pt, No underline

Page 35: [119] Formatted	Mona Kanaan	5/26/2013 9:17:00 AM

Font: 10 pt, No underline, Font color: Auto

Page 35: [120] Formatted	Mona Kanaan	5/26/2013 9:18:00 AM

Left, Space Before: 0 pt, After: 0 pt, Line spacing: single

For peer review only

Page 35: [121] Formatted	Mona Kanaan	5/26/2013 9:17:00 AM

Font: (Default) Times New Roman, 10 pt

Page 35: [122] Formatted	Mona Kanaan	5/26/2013 9:17:00 AM

Font: 10 pt, No underline, Font color: Auto

Page 35: [122] Formatted	Mona Kanaan	5/26/2013 9:17:00 AM

Font: 10 pt, No underline, Font color: Auto

Page 35: [123] Formatted	Mona Kanaan	5/26/2013 9:17:00 AM

Font: 10 pt, No underline

Page 35: [123] Formatted	Mona Kanaan	5/26/2013 9:17:00 AM

Font: 10 pt, No underline

Page 35: [124] Formatted	Mona Kanaan	5/26/2013 9:17:00 AM

Font: 10 pt, No underline

Page 35: [124] Formatted	Mona Kanaan	5/26/2013 9:17:00 AM

Font: 10 pt, No underline

Page 35: [125] Formatted	Mona Kanaan	5/26/2013 9:17:00 AM

Font: 10 pt, No underline

Page 35: [125] Formatted	Mona Kanaan	5/26/2013 9:17:00 AM

Font: 10 pt, No underline

Page 35: [126] Formatted	Mona Kanaan	5/26/2013 9:17:00 AM

Font: 10 pt, No underline

Page 35: [126] Formatted	Mona Kanaan	5/26/2013 9:17:00 AM

Font: 10 pt, No underline

Page 35: [127] Formatted	Mona Kanaan	5/26/2013 9:18:00 AM

Space Before: 0 pt, After: 0 pt, Line spacing: single

Page 35: [128] Formatted	Mona Kanaan	5/26/2013 9:18:00 AM

Normal, Indent: First line: 0", Line spacing: single

Page 35: [129] Formatted	Mona Kanaan	5/26/2013 9:17:00 AM

Font: 10 pt

Page 35: [130] Formatted	Mona Kanaan	5/26/2013 9:18:00 AM

Space Before: 0 pt, After: 0 pt, Line spacing: single

Page 35: [131] Formatted Table	Mona Kanaan	5/25/2013 8:48:00 AM

Formatted Table

Page 35: [132] Formatted	Mona Kanaan	5/26/2013 9:17:00 AM

Font: 10 pt, No underline, Font color: Auto

Page 35: [133] Formatted	Mona Kanaan	5/26/2013 9:18:00 AM

Left, Space Before: 0 pt, After: 0 pt, Line spacing: single

Page 35: [134] Formatted	Mona Kanaan	5/26/2013 9:17:00 AM

Font: 10 pt

Page 35: [135] Formatted	Mona Kanaan	5/26/2013 9:17:00 AM

Font: 10 pt, No underline, Font color: Auto

Page 35: [135] Formatted	Mona Kanaan	5/26/2013 9:17:00 AM

Font: 10 pt, No underline, Font color: Auto

For peer review only

Page 35: [136] Formatted	Mona Kanaan	5/26/2013 9:17:00 AM

Font: 10 pt, No underline, Font color: Auto

Page 35: [136] Formatted	Mona Kanaan	5/26/2013 9:17:00 AM

Font: 10 pt, No underline, Font color: Auto

Page 35: [137] Formatted	Mona Kanaan	5/26/2013 9:17:00 AM

Font: 10 pt, No underline, Font color: Auto

Page 35: [137] Formatted	Mona Kanaan	5/26/2013 9:17:00 AM

Font: 10 pt, No underline, Font color: Auto

Page 35: [138] Formatted	Mona Kanaan	5/26/2013 9:17:00 AM

Font: 10 pt

Page 35: [139] Formatted	Mona Kanaan	5/26/2013 9:18:00 AM

Space Before: 0 pt, After: 0 pt, Line spacing: single

Page 35: [140] Formatted	Mona Kanaan	5/26/2013 9:17:00 AM

Font: 10 pt, No underline, Font color: Auto

Page 35: [141] Formatted	Mona Kanaan	5/26/2013 9:18:00 AM

Left, Space Before: 0 pt, After: 0 pt, Line spacing: single

Page 35: [142] Formatted	Mona Kanaan	5/26/2013 9:17:00 AM

Font: 10 pt

Page 35: [143] Formatted	Mona Kanaan	5/26/2013 9:17:00 AM

Font: 10 pt, No underline, Font color: Auto

Page 35: [143] Formatted	Mona Kanaan	5/26/2013 9:17:00 AM

Font: 10 pt, No underline, Font color: Auto

Page 35: [144] Formatted	Mona Kanaan	5/26/2013 9:17:00 AM

Font: 10 pt, No underline, Font color: Auto

Page 35: [145] Formatted	Mona Kanaan	5/26/2013 9:18:00 AM

Left, Space Before: 0 pt, After: 0 pt, Line spacing: single

Page 35: [146] Formatted	Mona Kanaan	5/26/2013 9:17:00 AM

Font: 10 pt

Page 35: [147] Formatted	Mona Kanaan	5/26/2013 9:17:00 AM

Font: 10 pt, No underline

Page 35: [147] Formatted	Mona Kanaan	5/26/2013 9:17:00 AM

Font: 10 pt, No underline

Page 35: [148] Formatted	Mona Kanaan	5/26/2013 9:17:00 AM

Font: 10 pt, No underline

Page 35: [148] Formatted	Mona Kanaan	5/26/2013 9:17:00 AM

Font: 10 pt, No underline

Page 35: [149] Formatted	Mona Kanaan	5/26/2013 9:17:00 AM

Font: 10 pt, No underline, Font color: Auto

Page 35: [150] Formatted	Mona Kanaan	5/26/2013 9:18:00 AM

Left, Space Before: 0 pt, After: 0 pt, Line spacing: single

Page 35: [151] Formatted	Mona Kanaan	5/26/2013 9:17:00 AM

Font: 10 pt

For peer review only

Page 35: [152] Formatted	Mona Kanaan	5/26/2013 9:17:00 AM

Font: 10 pt, No underline, Font color: Auto

Page 35: [152] Formatted	Mona Kanaan	5/26/2013 9:17:00 AM

Font: 10 pt, No underline, Font color: Auto

Page 35: [153] Formatted	Mona Kanaan	5/26/2013 9:17:00 AM

Font: 10 pt, No underline

Page 35: [153] Formatted	Mona Kanaan	5/26/2013 9:17:00 AM

Font: 10 pt, No underline

Page 35: [154] Formatted	Mona Kanaan	5/26/2013 9:17:00 AM

Font: 10 pt, No underline

Page 35: [154] Formatted	Mona Kanaan	5/26/2013 9:17:00 AM

Font: 10 pt, No underline

Page 35: [155] Formatted	Mona Kanaan	5/26/2013 9:17:00 AM

Font: 10 pt, No underline

Page 35: [155] Formatted	Mona Kanaan	5/26/2013 9:17:00 AM

Font: 10 pt, No underline

Page 35: [156] Formatted	Mona Kanaan	5/26/2013 9:17:00 AM

Font: 10 pt, No underline

Page 35: [156] Formatted	Mona Kanaan	5/26/2013 9:17:00 AM

Font: 10 pt, No underline

Page 35: [157] Formatted	Mona Kanaan	5/26/2013 9:17:00 AM

Font: 10 pt, No underline, Font color: Auto

Page 35: [158] Formatted	Mona Kanaan	5/26/2013 9:18:00 AM

Left, Space Before: 0 pt, After: 0 pt, Line spacing: single

Page 35: [159] Formatted	Mona Kanaan	5/26/2013 9:17:00 AM

Font: 10 pt

Page 35: [160] Formatted	Mona Kanaan	5/26/2013 9:17:00 AM

Font: 10 pt, No underline, Font color: Auto

Page 35: [160] Formatted	Mona Kanaan	5/26/2013 9:17:00 AM

Font: 10 pt, No underline, Font color: Auto

Page 35: [161] Formatted	Mona Kanaan	5/26/2013 9:17:00 AM

Font: 10 pt, No underline

Page 35: [161] Formatted	Mona Kanaan	5/26/2013 9:17:00 AM

Font: 10 pt, No underline

Page 35: [162] Formatted	Mona Kanaan	5/26/2013 9:17:00 AM

Font: 10 pt, No underline

Page 35: [162] Formatted	Mona Kanaan	5/26/2013 9:17:00 AM

Font: 10 pt, No underline

Page 35: [163] Formatted	Mona Kanaan	5/26/2013 9:17:00 AM

Font: 10 pt, No underline

Page 35: [163] Formatted	Mona Kanaan	5/26/2013 9:17:00 AM

Font: 10 pt, No underline

For peer review only

Page 35: [164] Formatted	Mona Kanaan	5/26/2013 9:17:00 AM
Font: 10 pt, No underline		
Page 35: [164] Formatted	Mona Kanaan	5/26/2013 9:17:00 AM
Font: 10 pt, No underline		
Page 35: [165] Formatted	Mona Kanaan	5/26/2013 9:17:00 AM
Font: 10 pt, No underline, Font color: Auto		
Page 35: [166] Formatted	Mona Kanaan	5/26/2013 9:18:00 AM
Left, Space Before: 0 pt, After: 0 pt, Line spacing: single		
Page 35: [167] Formatted	Mona Kanaan	5/26/2013 9:17:00 AM
Font: 10 pt		
Page 35: [168] Formatted	Mona Kanaan	5/26/2013 9:17:00 AM
Font: 10 pt, No underline		
Page 35: [168] Formatted	Mona Kanaan	5/26/2013 9:17:00 AM
Font: 10 pt, No underline		
Page 35: [169] Formatted	Mona Kanaan	5/26/2013 9:17:00 AM
Font: 10 pt, No underline		
Page 35: [169] Formatted	Mona Kanaan	5/26/2013 9:17:00 AM
Font: 10 pt, No underline		
Page 35: [170] Formatted	Mona Kanaan	5/26/2013 9:17:00 AM
Font: 10 pt, No underline, Font color: Auto		
Page 35: [171] Formatted	Mona Kanaan	5/26/2013 9:18:00 AM
Left, Space Before: 0 pt, After: 0 pt, Line spacing: single		
Page 35: [172] Formatted	Mona Kanaan	5/26/2013 9:17:00 AM
Font: 10 pt		
Page 35: [173] Formatted	Mona Kanaan	5/26/2013 9:17:00 AM
Font: 10 pt, No underline, Font color: Auto		
Page 35: [173] Formatted	Mona Kanaan	5/26/2013 9:17:00 AM
Font: 10 pt, No underline, Font color: Auto		
Page 35: [174] Formatted	Mona Kanaan	5/26/2013 9:17:00 AM
Font: 10 pt, No underline		
Page 35: [174] Formatted	Mona Kanaan	5/26/2013 9:17:00 AM
Font: 10 pt, No underline		
Page 35: [175] Formatted	Mona Kanaan	5/26/2013 9:17:00 AM
Font: 10 pt, No underline		
Page 35: [175] Formatted	Mona Kanaan	5/26/2013 9:17:00 AM
Font: 10 pt, No underline		
Page 35: [176] Formatted	Mona Kanaan	5/26/2013 9:17:00 AM
Font: 10 pt, No underline		
Page 35: [176] Formatted	Mona Kanaan	5/26/2013 9:17:00 AM
Font: 10 pt, No underline		
Page 35: [177] Formatted	Mona Kanaan	5/26/2013 9:17:00 AM
Font: 10 pt, No underline		

For peer review only

Page 35: [177] Formatted	Mona Kanaan	5/26/2013 9:17:00 AM

Font: 10 pt, No underline

Page 35: [178] Formatted	Mona Kanaan	5/26/2013 9:17:00 AM

Font: 10 pt, No underline, Font color: Auto

Page 35: [179] Formatted	Mona Kanaan	5/26/2013 9:18:00 AM

Left, Space Before: 0 pt, After: 0 pt, Line spacing: single

Page 35: [180] Formatted	Mona Kanaan	5/26/2013 9:17:00 AM

Font: 10 pt

Page 35: [181] Formatted	Mona Kanaan	5/26/2013 9:17:00 AM

Font: 10 pt, No underline, Font color: Auto

Page 35: [181] Formatted	Mona Kanaan	5/26/2013 9:17:00 AM

Font: 10 pt, No underline, Font color: Auto

Page 35: [182] Formatted	Mona Kanaan	5/26/2013 9:17:00 AM

Font: 10 pt, No underline

Page 35: [182] Formatted	Mona Kanaan	5/26/2013 9:17:00 AM

Font: 10 pt, No underline

Page 35: [183] Formatted	Mona Kanaan	5/26/2013 9:17:00 AM

Font: 10 pt, No underline

Page 35: [183] Formatted	Mona Kanaan	5/26/2013 9:17:00 AM

Font: 10 pt, No underline

Page 35: [184] Formatted	Mona Kanaan	5/26/2013 9:17:00 AM

Font: 10 pt, No underline

Page 35: [184] Formatted	Mona Kanaan	5/26/2013 9:17:00 AM

Font: 10 pt, No underline

Page 35: [185] Formatted	Mona Kanaan	5/26/2013 9:17:00 AM

Font: 10 pt, No underline

Page 35: [185] Formatted	Mona Kanaan	5/26/2013 9:17:00 AM

Font: 10 pt, No underline

Page 35: [186] Formatted	Mona Kanaan	5/26/2013 9:17:00 AM

Font: 10 pt

Page 36: [187] Formatted	Mona Kanaan	5/26/2013 9:22:00 AM

Centered, Don't add space between paragraphs of the same style, Line spacing: single

Page 36: [188] Formatted	Mona Kanaan	5/26/2013 9:22:00 AM

Don't add space between paragraphs of the same style, Line spacing: single

Page 36: [189] Formatted	Mona Kanaan	5/26/2013 9:22:00 AM

Centered, Don't add space between paragraphs of the same style, Line spacing: single

Page 36: [190] Formatted	Mona Kanaan	5/26/2013 9:22:00 AM

Don't add space between paragraphs of the same style, Line spacing: single

Page 36: [191] Formatted	Mona Kanaan	5/26/2013 9:22:00 AM

Centered, Don't add space between paragraphs of the same style, Line spacing: single

Page 36: [192] Formatted	Mona Kanaan	5/26/2013 9:22:00 AM

Don't add space between paragraphs of the same style, Line spacing: single

For peer review only

Page 36: [193] Formatted	Mona Kanaan	5/26/2013 9:22:00 AM

Centered, Don't add space between paragraphs of the same style, Line spacing: single

Page 36: [194] Formatted	Mona Kanaan	5/26/2013 9:22:00 AM

Don't add space between paragraphs of the same style

Page 36: [195] Formatted	Mona Kanaan	5/26/2013 9:22:00 AM

Don't add space between paragraphs of the same style, Line spacing: single

Page 36: [196] Formatted	Mona Kanaan	5/26/2013 9:22:00 AM

Don't add space between paragraphs of the same style

Page 36: [197] Formatted	Mona Kanaan	5/26/2013 9:22:00 AM

Don't add space between paragraphs of the same style, Line spacing: single

Page 36: [198] Formatted	Mona Kanaan	5/26/2013 9:22:00 AM

Centered, Don't add space between paragraphs of the same style, Line spacing: single

Page 36: [199] Formatted	Mona Kanaan	5/26/2013 9:22:00 AM

Don't add space between paragraphs of the same style, Line spacing: single

Page 36: [200] Formatted	Mona Kanaan	5/26/2013 9:22:00 AM

Centered, Don't add space between paragraphs of the same style, Line spacing: single

Page 36: [201] Formatted	Mona Kanaan	5/26/2013 9:22:00 AM

Don't add space between paragraphs of the same style, Line spacing: single

Page 37: [202] Formatted	Mona Kanaan	5/26/2013 9:19:00 AM

Font: 10 pt, No underline, Font color: Auto

Page 37: [203] Formatted	Mona Kanaan	5/25/2013 8:50:00 AM

Line spacing: single

Page 37: [204] Formatted	Mona Kanaan	5/26/2013 9:19:00 AM

Font: 10 pt, Bold, No underline, Font color: Auto

Page 37: [205] Formatted	Mona Kanaan	5/26/2013 9:19:00 AM

Font: 10 pt, No underline, Font color: Auto

Page 37: [206] Formatted	Mona Kanaan	5/26/2013 9:19:00 AM

Font: 10 pt, No underline, Font color: Auto, English (U.S.)

Page 37: [207] Formatted	Mona Kanaan	5/26/2013 9:19:00 AM

Font: 10 pt, English (U.S.)

Page 37: [208] Formatted	Mona Kanaan	5/25/2013 8:52:00 AM

Line spacing: single

Page 37: [209] Formatted Table	Mona Kanaan	5/26/2013 8:54:00 AM

Formatted Table

Page 37: [210] Formatted	Mona Kanaan	5/25/2013 8:52:00 AM

Line spacing: single

Page 37: [211] Formatted	Mona Kanaan	5/25/2013 8:52:00 AM

Line spacing: single

Page 37: [212] Formatted	Mona Kanaan	5/25/2013 8:52:00 AM

Line spacing: single

Page 37: [213] Formatted	Mona Kanaan	5/26/2013 8:49:00 AM

Indent: Left: 0.19", No bullets or numbering, Tab stops: Not at 0.38"

Page 37: [214] Formatted	Mona Kanaan	5/26/2013 8:49:00 AM

Indent: Left: 0.19", No bullets or numbering, Tab stops: Not at 0.38"

Page 37: [215] Formatted	Mona Kanaan	5/26/2013 8:52:00 AM

Indent: Left: 0.19", No bullets or numbering, Tab stops: Not at 0.38"

Page 37: [216] Formatted	Mona Kanaan	5/26/2013 8:54:00 AM

Left, Indent: Left: 0.19", Line spacing: single, No bullets or numbering

Page 37: [217] Formatted	Mona Kanaan	5/25/2013 8:52:00 AM

Line spacing: single

Page 37: [218] Formatted	Mona Kanaan	5/26/2013 8:54:00 AM

Left, Indent: Left: 0.18", Line spacing: single, No bullets or numbering

Page 37: [219] Formatted	Mona Kanaan	5/25/2013 8:52:00 AM

Line spacing: single

Page 37: [220] Formatted	Mona Kanaan	5/26/2013 8:54:00 AM

Left, Indent: Left: 0.18", Line spacing: single, No bullets or numbering

Page 37: [221] Formatted	Mona Kanaan	5/25/2013 8:52:00 AM

Line spacing: single

Page 37: [222] Formatted	Mona Kanaan	5/26/2013 8:54:00 AM

Left, Indent: Left: 0.18", Line spacing: single, No bullets or numbering

Page 37: [223] Formatted	Mona Kanaan	5/25/2013 8:52:00 AM

Line spacing: single

Page 37: [224] Formatted	Mona Kanaan	5/25/2013 8:52:00 AM

Line spacing: single

Page 37: [225] Formatted	Mona Kanaan	5/26/2013 8:54:00 AM

Indent: Left: 0.18", Line spacing: single, No bullets or numbering

Page 37: [226] Formatted	Mona Kanaan	5/25/2013 8:52:00 AM

Line spacing: single

Page 37: [227] Formatted	Mona Kanaan	5/26/2013 8:54:00 AM

Indent: Left: 0.18", Line spacing: single, No bullets or numbering

Page 37: [228] Formatted	Mona Kanaan	5/25/2013 8:52:00 AM

Line spacing: single

Page 37: [229] Formatted	Mona Kanaan	5/26/2013 8:54:00 AM

Indent: Left: 0.18", Line spacing: single, No bullets or numbering

Page 37: [230] Formatted	Mona Kanaan	5/25/2013 8:52:00 AM

Line spacing: single

Page 37: [231] Formatted	Mona Kanaan	5/26/2013 8:54:00 AM

Indent: Left: 0.18", Line spacing: single, No bullets or numbering

Page 37: [232] Formatted	Mona Kanaan	5/25/2013 8:52:00 AM

Line spacing: single

Page 37: [233] Formatted	Mona Kanaan	5/25/2013 8:52:00 AM

Left, Line spacing: single

Page 37: [234] Formatted	Mona Kanaan	5/25/2013 8:52:00 AM

Line spacing: single

For peer review only

Page 37: [235] Formatted	Mona Kanaan	5/26/2013 8:54:00 AM

Indent: Left: 0.18", Line spacing: single, No bullets or numbering

Page 37: [236] Formatted	Mona Kanaan	5/25/2013 8:52:00 AM

Line spacing: single

Page 37: [237] Formatted	Mona Kanaan	5/26/2013 8:54:00 AM

Indent: Left: 0.18", Line spacing: single, No bullets or numbering

Page 37: [238] Formatted	Mona Kanaan	5/25/2013 8:52:00 AM

Line spacing: single

Page 37: [239] Formatted	Mona Kanaan	5/26/2013 8:54:00 AM

Indent: Left: 0.18", Line spacing: single, No bullets or numbering

Page 37: [240] Formatted	Mona Kanaan	5/25/2013 8:52:00 AM

Line spacing: single

Page 37: [241] Formatted	Mona Kanaan	5/26/2013 8:54:00 AM

Indent: Left: 0.18", Line spacing: single, No bullets or numbering

Page 37: [242] Formatted	Mona Kanaan	5/25/2013 8:52:00 AM

Line spacing: single

Page 37: [243] Formatted	Mona Kanaan	5/26/2013 8:54:00 AM

Indent: Left: 0.18", Line spacing: single, No bullets or numbering

Page 37: [244] Formatted	Mona Kanaan	5/25/2013 8:52:00 AM

Line spacing: single

Page 37: [245] Formatted	Mona Kanaan	5/26/2013 8:54:00 AM

Indent: Left: 0.18", Line spacing: single, No bullets or numbering

Page 37: [246] Formatted	Mona Kanaan	5/25/2013 8:52:00 AM

Line spacing: single

Page 37: [247] Formatted	Mona Kanaan	5/25/2013 8:52:00 AM

Line spacing: single

Page 37: [248] Formatted	Mona Kanaan	5/25/2013 8:52:00 AM

Line spacing: single

Page 37: [249] Formatted	Mona Kanaan	5/25/2013 8:52:00 AM

Line spacing: single

Page 37: [250] Formatted	Mona Kanaan	5/25/2013 8:52:00 AM

Line spacing: single

Page 37: [251] Formatted	Mona Kanaan	5/25/2013 8:52:00 AM

Space After: 0 pt, Line spacing: single

For peer review only

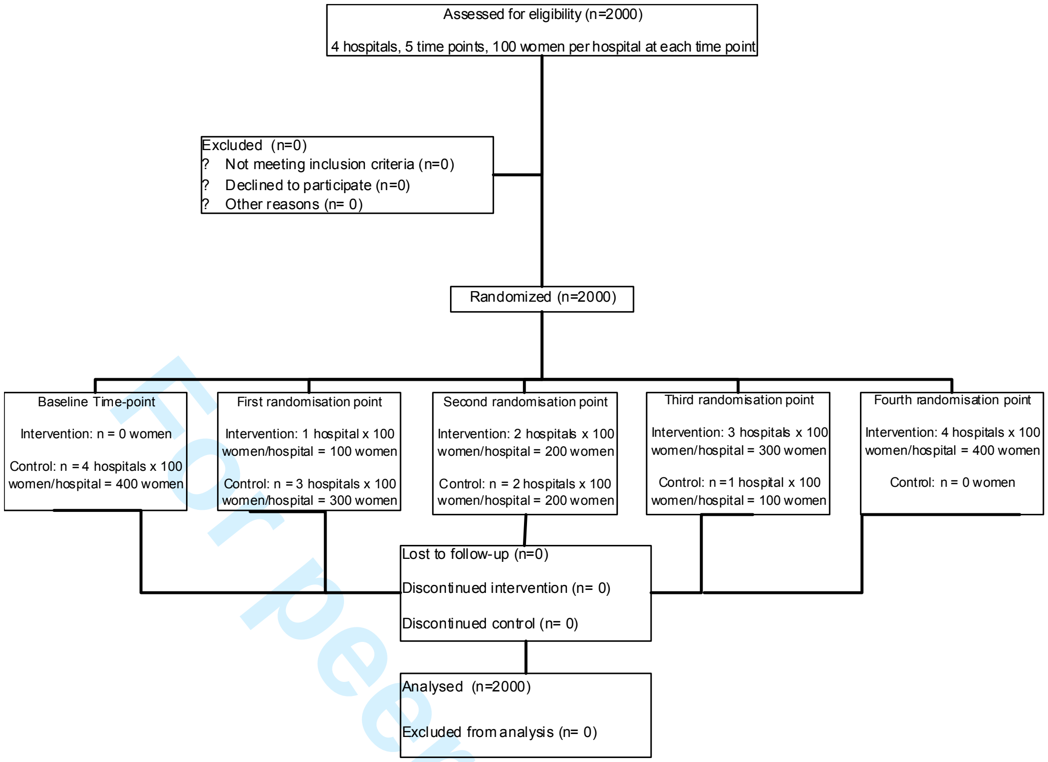

Figure 1: Flow of Participants

For peer review only

Figure 1: A graphical representation of the overall satisfaction question of the women stratified by hospital. Data presented represent proportion in each category of overall satisfaction at the different time periods.

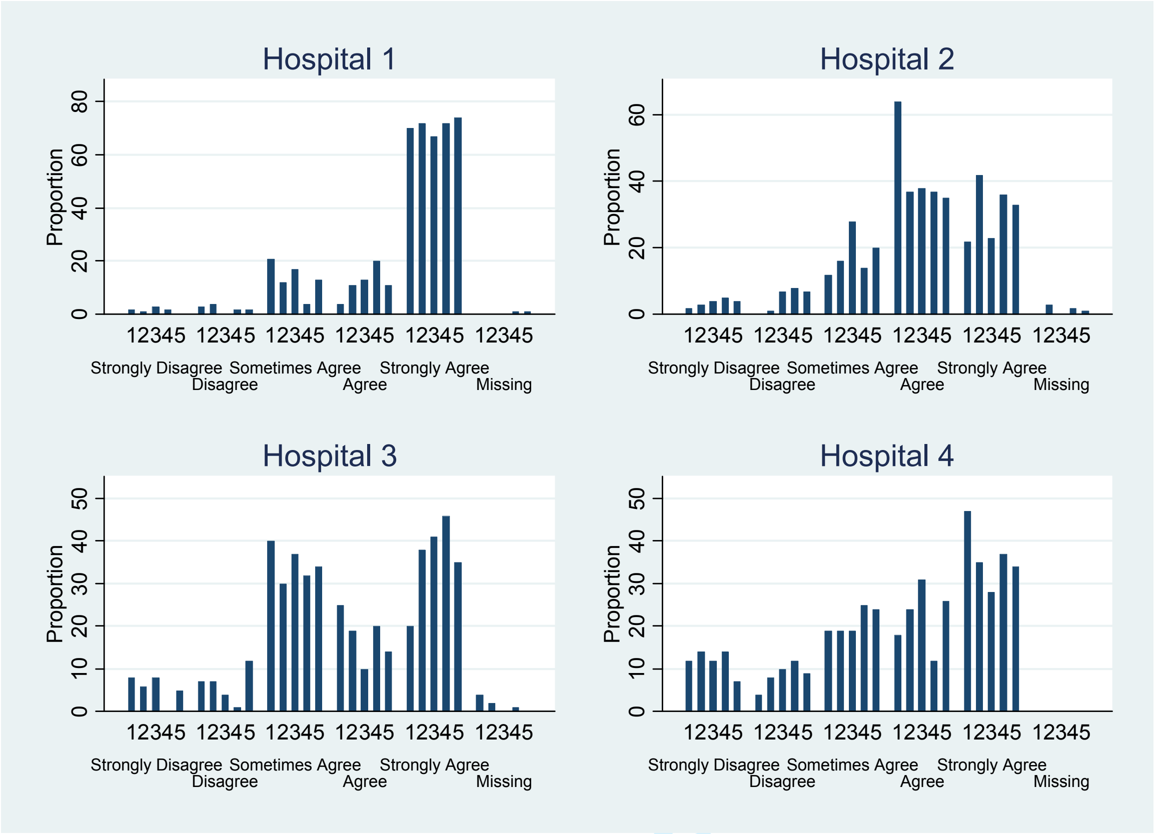

For peer review only - http://bmjopen.bmj.com/site/about/guidelines.xhtml

Additional File One: Schema of the study design*

Hospital	Time				
	1	2	3	4	5
1					
2					
3					
4					

Time 1: Baseline measurement
Time 2: Follow the implementation of intervention at randomly selected hospitals 1
Time 3: Follow the implementation of intervention at randomly selected hospitals 2
Time 4: Follow the implementation of intervention at randomly selected hospitals 3
Time 5: Follow the implementation of intervention at randomly selected hospitals 4

* Shaded area refer to post-intervention

For peer review only

Additional File Two:

A graphical representation of the overall satisfaction outcome score of the women stratified by hospital and time of intervention. Data presented are for complete MMISS only. The rows represent the four hospitals and the columns the five randomisation points. For presentation purposes, the hospitals were coded such that Hospital 1 represented the hospital that had the intervention delivered first which was "time 2" and so forth; "time 1" was the baseline. The title of each graph indicates the hospital and time point presented, for example, a title of 2,3 indicates that this represents the score distribution in Hospital 2 at time 3 (the first intervention period for this hospital).

For peer review only

For peer review only - http://bmjopen.bmj.com/site/about/guidelines.xhtml

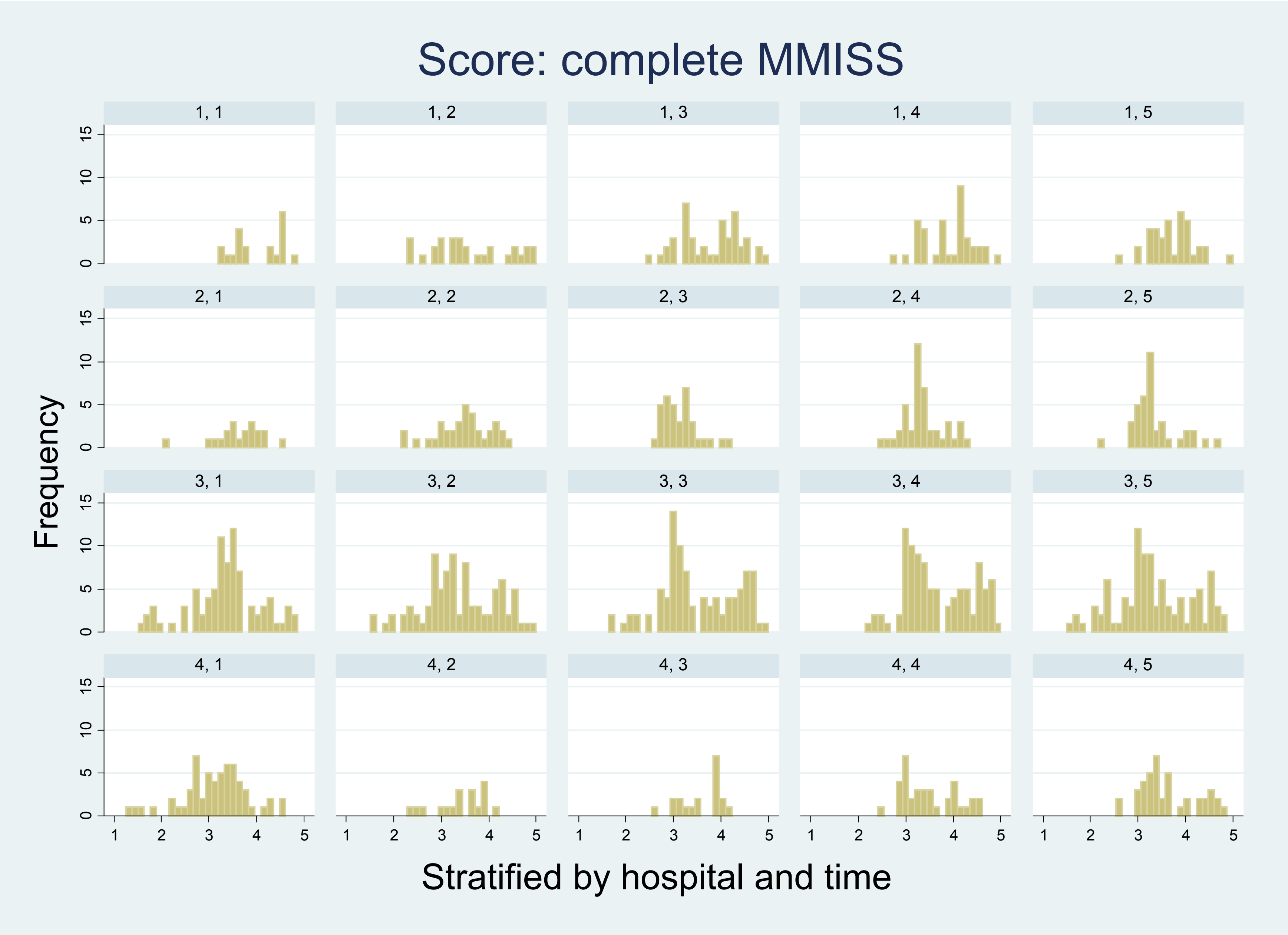

For peer review only - http://bmjopen.bmj.com/site/about/guidelines.xhtml

For peer review only

For peer review only - http://bmjopen.bmj.com/site/about/guidelines.xhtml

1 **Table 1: Demographic Characteristics of the women across the time periods of the study[!]**

	1	2	3	4	5
N	400	400	400	400	400
Mean age (SD)	24.79 (5.71)	25.12 (6.11)	25.07 (6.4)	24.97 (5.74)	25.37(6.04)
Median age (Q1, Q3)*	24 (20, 28)	24 (20, 29)	24 (20, 29.5)	24 (20, 29)	24 (20, 29)
Occupation: Housewife	377 (94)	377 (94)	387 (97)	378 (94)	383 (96)
Education					
Illiterate/Read and Write	53 (13)	51 (13)	43 (11)	53 (13)	42 (11)
Primary	291 (74)	306 (77)	304 (76)	305 (77)	300 (76)
High School +	51 (13)	42 (11)	53 (13)	38 (10)	54 (14)
Mean husband's age (SD)	30.98 (6.89)	31.19 (7.18)	31 (6.82)	30.16 (6.35)	31.07 (6.53)
Median husband's age (Q1, Q3)	30 (26, 35)	30 (26, 35)	30 (26, 35)	29 (26, 34)	30 (26, 35)
Husband's Occupation					
Labourer (manual worker)	152 (39)	131 (33)	134 (34)	143 (36)	136 (34)
Clerk	72 (18)	71 (18)	64 (16)	80 (20)	71 (18)
(Self-employed)	144 (37)	163 (41)	175 (44)	166 (42)	175 (44)
Other	24 (6)	33 (8)	24 (6)	7 (2)	13 (3)
Husband's Education					
Illiterate/Read and Write	55 (14)	48 (12)	41 (10)	37 (9)	33 (8)
Primary	299 (76)	295 (74)	310 (78)	310 (79)	310 (79)

For peer review only

High School +	41 (10)	53 (13)	45 (11)	46 (12)	50 (13)
Home Ownership					
Owns home	192 (49)	197 (49)	217 (55)	214 (55)	197 (50)
Share	201 (51)	202 (51)	179 (45)	178 (45)	198 (50)
Parity: Nulliparous	100 (25)	120 (30)	124 (31)	97 (24)	88 (22)
New born gender: Male	215 (56)	193 (49)	179 (45)	198 (52)	202 (52)

* Q1 is the first quartile and Q3 is the third quartile.

! Numbers represent counts (percentage) unless otherwise stated.

For peer review only - http://bmjopen.bmj.com/site/about/guidelines.xhtml

Table 2: Numbers (and percentages) of women who agreed/strongly agreed with various questions concerning their labour/delivery experience. The first two columns give the numbers comparing all the intervention clusters to the control clusters; the other columns give the corresponding numbers stratified by hospital.*

	Control Period	Intervention Period	Control Period				Intervention Period			
	All	All	1	2	3	4	1	2	3	4
N	1000	1000	100	200	300	400	400	300	200	100
Overall satisfaction with doctors' communication skills during labour/delivery	619 (62)	713 (72)	74 (74)	163 (83)	150 (51)	232 (58)	338 (85)	200 (67)	115 (58)	60 (60)
Did the doctors identify themselves?	132 (13)	118 (12)	1 (1)	11 (6)	94 (31)	26 (7)	35 (9)	7 (2)	62 (31)	14 (14)
Did the doctor greet you?	208 (21)	323 (32)	36 (36)	45 (23)	86 (29)	41 (10)	183 (46)	55 (19)	63 (32)	22 (22)
Did the doctor look at you when talking to you?	434 (43)	599 (60)	61 (61)	158 (79)	94 (31)	121 (30)	305 (76)	181 (61)	78 (39)	35 (35)
Did the doctor show interest in you as a person?	393 (39)	329 (33)	47 (47)	42 (21)	139 (46)	165 (41)	188 (47)	21 (7)	83 (42)	37 (37)
Did the doctor insult you?	55 (6)	29 (3)	2 (2)	3 (2)	15 (5)	35 (9)	5 (1)	19 (6)	1 (1)	4 (4)
Did the doctor use humour to comfort you?	78 (8)	118 (12)	19 (19)	13 (7)	34 (11)	12 (3)	70 (18)	7 (2)	34 (17)	7 (7)
Did the doctor use any terms to calm you down?	363 (36)	419 (42)	44 (44)	102 (51)	109 (36)	108 (27)	200 (50)	116 (39)	73 (37)	30 (30)

For peer review only

For peer review only - http://bmjopen.bmj.com/site/about/guidelines.xhtml

Did the doctor use his/her hands to assist you or to reassure you?	314 (32)	409 (42)	52 (54)	115 (58)	79 (26)	68 (17)	200 (52)	137 (46)	57 (29)	15 (16)
Did the doctor shout or scream at you?	125 (13)	71 (7)	6 (6)	15 (8)	28 (9)	76 (19)	18 (5)	31 (10)	10 (5)	12 (12)
Did the doctor listen to you with concern and without interrupting you?	413 (46)	551 (58)	57 (62)	108 (65)	98 (33)	150 (44)	313 (79)	116 (45)	73 (37)	49 (50)
Was the doctor engaged in other issues, so you felt being unattended and annoyed?	165 (17)	67 (7)	3 (3)	20 (10)	75 (25)	67 (17)	12 (3)	19 (6)	27 (14)	9 (9)
Did you feel let down by the way he/she was dealing with you?	66 (7)	31 (3)	2 (2)	10 (5)	10 (3)	44 (11)	10 (3)	19 (6)	1 (1)	1 (1)
Did the doctor explain all the steps before doing the clinical exam?	249 (25)	278 (29)	17 (17)	88 (45)	41 (14)	103 (26)	70 (18)	128 (47)	46 (23)	34 (35)
Did the doctor explain the findings from the clinical exam?	326 (34)	399 (41)	42 (68)	82 (42)	61 (20)	141 (36)	181 (46)	101 (37)	63 (32)	54 (56)
Were you annoyed by the doctor explaining all the findings?	40 (5)	73 (10)	4 (8)	18 (13)	3 (1)	15 (5)	29 (11)	39 (18)	2 (1)	3 (3)
Did the doctor explain all the alternative choices to you?	176 (23)	145 (17)	12 (27)	44 (35)	94 (31)	26 (9)	37 (10)	31 (16)	66 (33)	11 (13)
Did the doctor help you in	175	158	12	25	97	41	52	16	75	15

For peer review only

making decisions?	(29)	(23)	(44)	(29)	(33)	(21)	(19)	(9)	(38)	(35)
Was the doctor annoyed from your questions and avoided answering them?	122 (14)	54 (6)	9 (11)	14 (9)	35 (12)	64 (19)	20 (6)	21 (8)	6 (3)	7 (7)
Did the doctor use medical terms in explanation, thus they were not understood?	77 (8)	48 (5)	0 (0)	6 (3)	55 (19)	16 (5)	1 (0)	5 (2)	37 (19)	5 (6)
Did the doctor give you conflicting medical information?	31 (3)	12 (1)	1 (1)	3 (2)	6 (2)	21 (6)	0 (0)	9 (3)	2 (1)	1 (1)

* Due to missing information some of the percentages do not tie up with the total number given.

For peer review only

Table 3: Summary statistics for the outcome of women satisfaction computed as the average score and Average Score for Complete MMISS . The first two columns give the numbers comparing all the intervention clusters to the control clusters; the other columns give the corresponding numbers stratified by hospital

	Control Period	Intervention Period	Control Period				Intervention Period			
	All	All	1	2	3	4	1	2	3	4
Average Score										
N	1000	1000	100	200	300	400	400	300	200	100
Mean (SD)	3.23 (0.72)	3.42 (0.73)	3.57 (0.72)	3.43 (0.59)	3.33 (0.78)	3.06 (0.67)	3.54 (0.73)	3.25 (0.66)	3.48 (0.77)	3.33 (0.71)
Median (Q1, Q3)	3.3 (2.8,3.7)	3.3 (3.0, 4.1)	3.6 (3.1, 4.3)	3.5 (3.1, 3.8)	3.3 (2.9, 3.9)	3.2 (2.6, 3.5)	3.5 (3.0, 4.2)	3.2 (2.9, 3.7)	3.4 (3.0, 4.2)	3.3 (3.0, 3.7)
Average Score for Complete MMISS										
N	472	498	20	55	265	132	148	118	191	41
Mean (SD)	3.43 (0.71)	3.54 (0.66)	4.04 (0.54)	3.52 (0.56)	3.42 (0.77)	3.33 (0.62)	3.77 (0.62)	3.29 (0.43)	3.50 (0.77)	3.62 (0.60)
Median (Q1, Q3)	3.4 (3.0,3.9)	3.3 (3.1, 4.1)	4.1 (3.6,4.6)	3.6 (3.2, 3.9)	3.3 (2.9, 4.1)	3.4 (3.0, 3.8)	3.8 (3.3, 4.2)	3.2 (3.0, 3.4)	3.35 (3.1, 4.2)	3.4 (3.2, 4)

For peer review only

Table 4: Regression analysis estimates for the average score and average score for complete MMISS on intervention and time controlling for clustering by hospital using three estimation procedures LMM, GLAMM and GEE. Estimates are presented together with 95% confidence interval (CI) limits as subscripts (${}_{Lower}Est_{Upper}$).

	Average Score			Average Score Complete MMISS		
	LMM	GLAMM	GEE	LMM	GLAMM	GEE
	${}_{Lower}Est_{Upper}$	${}_{Lower}Est_{Upper}$	${}_{Lower}Est_{Upper}$	${}_{Lower}Est_{Upper}$	${}_{Lower}Est_{Upper}$	${}_{Lower}Est_{Upper}$
**Fixed-effects**						
intervention	${}_{-0.09}0.03_{0.14}$	${}_{-0.08}0.03_{0.15}$	${}_{-0.08}0.03_{0.14}$	${}_{-0.29}-0.13_{0.03}$	${}_{-0.29}-0.13_{0.04}$	${}_{-0.26}-0.10_{0.06}$
time: 2	${}_{-0.08}0.02_{0.13}$	${}_{-0.08}0.02_{0.12}$	${}_{-0.08}0.02_{0.12}$	${}_{-0.10}0.04_{0.19}$	${}_{-0.10}0.05_{0.19}$	${}_{-0.10}0.05_{0.19}$
time: 3	${}_{-0.14}-0.03_{0.09}$	${}_{-0.14}-0.03_{0.08}$	${}_{-0.14}-0.03_{0.08}$	${}_{-0.02}0.12_{0.27}$	${}_{-0.02}0.12_{0.27}$	${}_{-0.03}0.12_{0.27}$
time: 4	${}_{-0.08}0.05_{0.18}$	${}_{-0.08}0.05_{0.18}$	${}_{-0.08}0.05_{0.18}$	${}_{0.13}0.31_{0.49}$	${}_{0.12}0.30_{0.49}$	${}_{0.10}0.28_{0.47}$
time: 5	${}_{-0.14}0.01_{0.16}$	${}_{-0.15}0.01_{0.16}$	${}_{-0.14}0.01_{0.15}$	${}_{-0.02}0.18_{0.38}$	${}_{-0.03}0.17_{0.37}$	${}_{-0.05}0.15_{0.35}$
constant	${}_{3.14}3.32_{3.50}$	${}_{3.16}3.32_{3.48}$	${}_{3.16}3.32_{3.48}$	${}_{3.20}3.84_{3.67}$	${}_{3.23}3.44_{3.64}$	${}_{3.28}3.43_{3.59}$
**Random-effects**						
	SD	Var		SD	Var	
**hospital**	${}_{0.07}0.17_{0.40}$	0. 02 (0.016)		${}_{0.09}0.22_{0.51}$	0.03 (0.026)	
**Residual**	${}_{0.68}0.71_{0.73}$	0.51 (0.016)		${}_{0.64}0.67_{0.70}$	0.45 (0.02)	

For peer review only

For peer review only - http://bmjopen.bmj.com/site/about/guidelines.xhtml

Table 5: Numbers (and percentages) of "No" for items on the observational checklist concerning communication between doctor and woman during labour/delivery, unless otherwise stated. Due to missing information some of the percentages do not tie up with the total number given. The first two columns give the numbers comparing all the intervention clusters to the control clusters; the other columns give the corresponding numbers stratified by hospital.

	Control Period	Intervention Period	Control Period				Intervention Period			
Hospital	All	All	1	2	3	4	1	2	3	4
N	565	631	98	194	106	167	326	218	38	49
1. Did the doctor identify himself/herself?	539 (100)	621 (99)	90 (100)	177 (99)	105 (100)	167 (100)	320 (99)	215 (100)	38 (100)	48 (98)
2. Did the doctor call the woman by her name?	132 (24)	99 (16)	22 (24)	48 (27)	22 (21)	40 (24)	52 (16)	30 (14)	6 (16)	11 (22)
3. Did the doctor take the woman's permission to examine her?	326 (61)	305 (49)	53 (59)	113 (64)	61 (58)	99 (60)	183 (57)	77 (36)	18 (47)	27 (55)
4. Did the doctor explain the plan he/she will follow?	497 (92)	577 (92)	81 (90)	168 (95)	98 (93)	150 (90)	296 (92)	203 (94)	35 (92)	43 (88)
Before starting the vaginal examination, did the doctor										
5. take her permission for the exam?	335 (62)	262 (42)	58 (65)	115 (65)	58 (55)	104 (63)	155 (48)	68 (32)	14 (37)	25 (51)
6. close the door?	472 (88)	568 (91)	85 (96)	136 (77)	87 (83)	164 (99)	313 (97)	180 (84)	26 (68)	49 (100)

For peer review only - http://bmjopen.bmj.com/site/about/guidelines.xhtml

7. cover the woman during the exam?	315 (59)	116 (18)	20 (23)	97 (55)	79 (75)	119 (72)	32 (10)	21 (10)	21 (55)	42 (91)
8. ask the woman to bend her knees?	210 (39)	79 (13)	34 (38)	76 (43)	31 (30)	69 (42)	48 (15)	34 (16)	6 (16)	7 (14)
After completing the vaginal examination, did the doctor										
9. tell the woman the results of the exam?	418 (78)	504 (81)	73 (82)	157 (89)	74 (70)	114 (69)	270 (84)	174 (81)	31 (82)	29 (59)
10. explain the next steps to be followed?	486 (91)	586 (94)	73 (83)	168 (95)	98 (94)	147 (89)	302 (94)	204 (95)	34 (89)	46 (94)
11. relay the findings to other team members?	241 (45)	102 (16)	17 (19)	87 (49)	45 (43)	92 (59)	41 (13)	30 (14)	12 (36)	19 (39)
12. relay the findings to the midwives/nurses?	393 (80)	579 (93)	60 (83)	154 (87)	70 (71)	109 (75)	312 (97)	211 (98)	32 (84)	24 (49)
13. Did the doctor give instructions about eating and drinking?	463 (87)	516 (83)	77 (89)	148 (84)	90 (86)	148 (90)	261 (81)	183 (85)	33 (87)	39 (80)
14. Did the doctor give instructions about movement?	335 (63)	428 (70)	65 (77)	112 (62)	59 (57)	99 (60)	222 (71)	155 (72)	26 (68)	25 (51)
15. Was the woman asked any questions at this stage?	168 (32)	94 (15)	11 (14)	82 (46)	26 (25)	49 (30)	43 (14)	38 (18)	6 (16)	7 (14)
16. Did the woman ask any questions at this stage?	292 (56)	281 (46)	34 (44)	123 (68)	52 (52)	83 (51)	152 (49)	93 (44)	13 (35)	23 (47)
17. Did the doctor respond to her queries?	275 (63)	342 (64)	32 (44)	96 (70)	59 (65)	88 (65)	168 (65)	124 (64)	21 (64)	29 (63)
18. Was the doctor responsive to the woman's pain?	285 (59)	333 (57)	35 (47)	95 (58)	54 (59)	101 (65)	162 (53)	119 (58)	22 (73)	30 (63)

For peer review only - http://bmjopen.bmj.com/site/about/guidelines.xhtml

19. Was the woman encouraged at this stage?	186 (40)	239 (42)	17 (25)	59 (38)	33 (36)	77 (52)	115 (40)	85 (42)	16 (53)	23 (48)
20. Was the woman told about the proximity of labour?	242 (55)	363 (66)	25 (39)	79 (54)	49 (55)	89 (63)	180 (65)	136 (68)	20 (71)	27 (60)
21. Was the woman told about the stages of labour?	399 (95)	533 (98)	49 (83)	133 (96)	85 (97)	132 (96)	269 (98)	197 (99)	28 (100)	39 (98)
22. Was the woman told about her role during labour?	347 (83)	508 (94)	29 (51)	114 (83)	80 (90)	124 (91)	256 (93)	190 (95)	25 (89)	37 (93)
23. During delivery, was the woman asked to push?	26 (6)	23 (4)	6 (11)	6 (4)	3 (3)	11 (8)	13 (5)	7 (4)	3 (11)	0 (0)
24. Did they explain to the woman when and how to push?	237 (57)	230 (42)	30 (54)	76 (55)	48 (55)	83 (61)	114 (41)	85 (43)	15 (54)	16 (40)
25. Did the woman ask any questions at this stage?	284 (68)	296 (55)	35 (63)	100 (73)	59 (67)	90 (66)	166 (61)	95 (48)	15 (54)	20 (50)
26. Did the doctor respond to her queries?	264 (77)	330 (72)	36 (68)	77 (77)	66 (81)	85 (78)	160 (71)	127 (73)	17 (81)	26 (68)
27. Did the doctor inform the mother about her baby's status?	307 (75)	386 (72)	26 (49)	92 (68)	72 (83)	117 (86)	171 (63)	161 (82)	25 (89)	29 (74)
28. Was the woman congratulated after the delivery?	217 (53)	214 (40)	26 (47)	63 (46)	49 (58)	79 (58)	102 (37)	81 (41)	10 (36)	21 (53)
29. Was the woman instructed about the process after the delivery?	392 (95)	515 (96)	51 (93)	127 (93)	82 (95)	132 (97)	267 (97)	182 (92)	27 (96)	39 (98)
30. Was the woman instructed about how to care for her newborn?	409 (99)	534 (99)	53 (98)	134 (98)	86 (100)	136 (100)	273 (100)	194 (98)	28 (100)	39 (100)
31. Was the woman reassured the she is going to be fine?	373 (90)	511 (95)	34 (62)	125 (91)	82 (95)	132 (97)	261 (96)	184 (93)	28 (100)	38 (95)
Mean (SD) of the average score	0.34	0.39	0.41	0.31	0.35	0.31	0.39	0.40	0.38	0.38

For peer review only

For peer review only - http://bmjopen.bmj.com/site/about/guidelines.xhtml

	(0.13)	(0.10)	(0.13)	(0.13)	(0.10)	(0.13)	(0.10)	(0.10)	(0.13)	(0.10)
Mean (SD) of the average score for complete checklist	0.34 (0.10)	0.39 (0.09)	0.40 (0.13)	0.36 (0.10)	0.35 (0.10)	0.32(0 .10)	0.39 (0.09)	0.39(0 .08)	0.38 (0.08)	0.38 (0.11)

For peer review only

Research Proposal
Presented to CCCC/AUB from the Syrian Team
June 2007

Team members
Hyam Bashour
Asmaa Abdulsalam
Mayada Kharouf
Salah Cheikha
Mohammed Tabbaa
In collaboration with ***Mona Kanaan*** from FHS, AUB

Title:

Effect of doctors' training in interpersonal and communication skills on women's satisfaction at labor and delivery: a stepped wedge cluster randomized trial in Damascus

Introduction

The growing body of literature linking communication skills to a host of valued patient outcomes, including satisfaction, adherence, and positive health indicators, is drawing increasing attention to the centrality of the interpersonal skills of healthcare providers to the delivery of quality medical care. As a result, communication in health-care, particularly between health-care professionals and patients, has attracted an increasing amount of attention at official and professional levels in recent years. Many academic and statutory bodies have all been involved in initiatives to promote good communication (Teutsch, 2003). In the United Kingdom, guidance from the General Medical Council on undergraduate medical education places communication skills training at the heart of the curriculums although the extent of compliance with these recommendations is variable. More recently, in the National Health System (NHS) Plan, the UK Department of Health announced plans for new training in communication skills across all professions in the NHS. These initiatives are in part a response to increasing evidence from researchers that the quality of the interaction

For peer review only - http://bmjopen.bmj.com/site/about/guidelines.xhtml

between patients and their care providers may have a significant effect on a variety of aspects of patient well being. These include satisfaction, knowledge and understanding, compliance with advice or treatment, quality of life, and psychological and other health outcomes (Ong 1995, Stewart, 1995). In the United States of America, bodies such as the National Board of Medical Examiners and the Educational Commission for Foreign Medical graduates have proposed an examination in communication skills of the medical students (Teutsch, 2003).

In primary care literature, there are many examples of positive benefits for patients when physicians engage in a patient-centered approach to treatment and care (Jenkins and Fallowfield, 2002). Beck et al (2002) argue that a communicative provider-patient relationship is especially important in the management of chronic disease. They have classified behaviour into three key functions of the interaction: data gathering to understand the patient (gathering information), development of rapport and responding to the patient's emotions (developing a therapeutic relationship), and patient education and behavioural management (decision making and management). Figure 1 provides a schematic of the model developed by them. Beck et al in their work concluded that existing research is limited because of lack of consensus of what to measure.

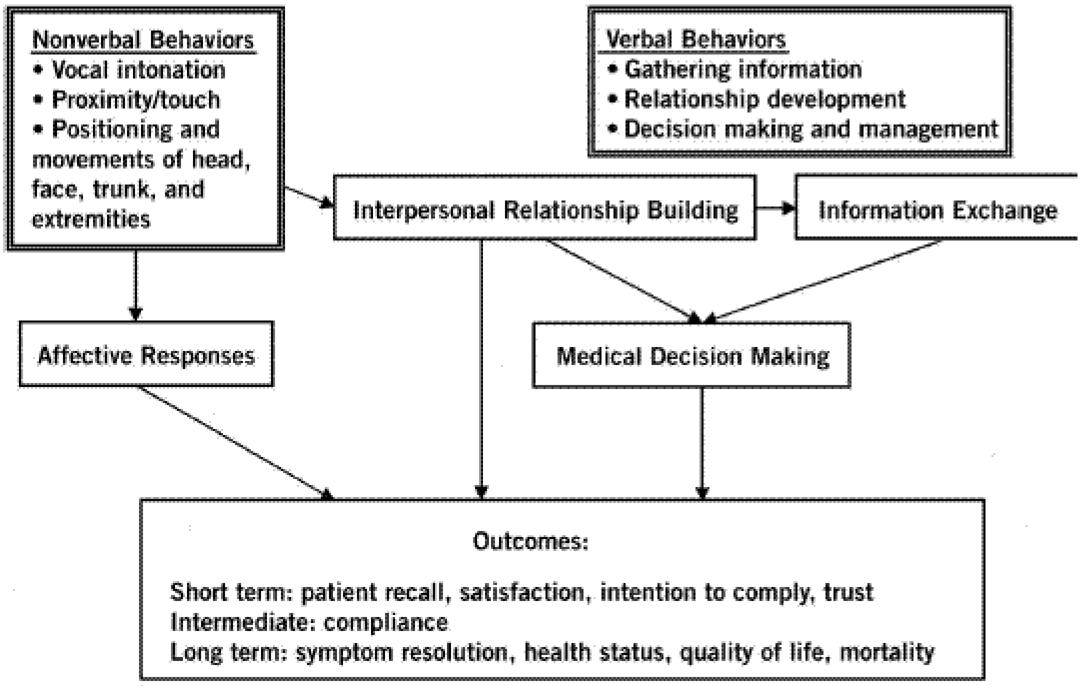

Figure 1. Domains of communication in the provider-patient relationship.

Maternity care is an area of health-care in which the importance of good communication has received particular attention. *Changing Childbirth*, the report of the Expert Maternity Group, commissioned in 1992 by the Department of Health to review maternity care in England and Wales, highlighted the importance of good communication and recognized that in many cases communication is not as good as it should be (Dept. of Health 1993). A separate volume outlined the principles of good communication and detailed examples of good communication practice (Dept. of Health , 1993).

Studies of women's views of maternity care suggest that good communication is central in determining whether women are satisfied with the care that they receive or not (Garcia, 1998, Kabakian-Khasholian et al, 2000). In Changing Childbirth, the provision of appropriate and accessible information was recognized as an important aspect of communication. Listening to women's preferences and concerns is also central (Dept of Health, 1993). Changing Childbirth was explicit in recognizing that there is no template for effective communication and good communication will always be difficult to define, but studies of women's views of maternity care support the idea that good communication is essentially a two-way process. Observational studies of communication between midwives and women during labour have highlighted the misunderstandings that can occur when communication is poor at this time and identified areas where communication could be improved (Garcia, 1990).

The report of the second national US survey of women childbearing's experiences showed the nearly all women reported receiving supportive care (comfort, emotional support, information) for some period while in labour by at least one person, mostly husbands (Childbirth Connection, 2006). Bingham (2005) stressed the importance of carrying out patient satisfaction surveys to help hospitals change its childbirth policy. The Cochrane authors, however, found that features of modern hospital environments, as currently organized, make it challenging for hospital staff to provide continuous labor support, in spite of the importance of continuous care (Hodnett *et al*, 2003).

Despite the acknowledged importance of communication in maternity care, and the official recognition that communication is not always as good as it should be, there have been few evaluations of strategies to improve communication between women and their care providers. Previous reviews of doctor-patient communication have not included any studies carried out in maternity care (Ong 1995, Stewart 1995). The review carried out by Rowe (2002) aimed

For peer review only

to fill in that gap and thus identified and reviewed trials of the effectiveness of interventions aimed at improving communication between health professionals and women in maternity care. The review identified no trials in the area of antenatal care, but also identified a major gap in knowledge relating to communication in a number of key areas of delivery and postnatal care. They recommended that trials of interventions to improve communication between carers and women in labor and in the postnatal period would be particularly useful. All the studies under review were carried out in developed countries, mainly in the United Kingdom.

The theoretical model for doctor-patient communication suggested by Ong and colleagues (1995) is a useful model to conceptualize an interventional research that aims to improve communication.

**Background variables**	**Actual content of communication (Process)**	**Patient outcomes**
Disease characteristics<br><br>Culture<br><br>Type of patient personality<br><br>Type of doctor personality<br><br>Doctor patient relationship	Communicative behavior including privacy<br><br>Instrumental (cure-oriented) vs. affective behaviors (care-oriented).	Short term: satisfaction, compliance, recall, denial, understanding, hope, etc<br><br>Intermediate and Long term: health status, psychological reaction

Rationale

In Syria, previous work by the researchers both in hospitals and at the community level, very clearly showed that continuous support of women during labor and delivery does not exist. Companionship is not allowed at hospitals, however in a population based study, women reported the existence of a companion at delivery in 37% of the cases (Bashour et al, in press). In that study, it was also evident that women do receive a very fragmented maternal care (only 37% of the studied women attended at delivery by the same person cared the antenatal period), which means that women are usually confronted with birth attendants

For peer review only

whom they meet for the first time at the time of delivery. Furthermore, the length of stay at the hospital for delivery was very short (one fourth of the women stayed for 1-2 hours only), and most probably this is due to the lack of comfort at the hospital.

Also Syrian women expressed very clearly their dissatisfaction with being left alone and they also expressed their negative experiences with being attended by care providers who are not equipped with good communication skills (Hafez *et al*, forthcoming publication). It was very clear that Syrian women are left without any social and emotional support during a critical period in their lives. In most occasions, they are not allowed to be accompanied by their relatives when in labor and delivery, but worse than this is that they seem to be cared of by doctors and midwives with poor communication skills and little interpersonal competences. There were many instances in which doctors and midwives themselves were sources of dissatisfaction.

Apart from women's views, we are fully aware that medical students in Syria, whether in the undergraduate or postgraduate levels, do not receive any formal teaching in communication skills (FOM, 2005). Furthermore, the importance of good communication is not a part of the clinical and ward training for postgraduate students in Obstetrics and Gynecology (Department of Ob/GYN, 2005).

A small scale qualitative study that was carried out in the Maternity Hospitals highlighted the importance of tackling the issue of communication skills in those hospitals (Abdusalam et al, 2006). It was evident that things might be better in private hospitals as compared to public hospitals, as doctors themselves stated. Doctors were themselves perpetrators of violence against women (verbal and physical). Although the word "violence" did not came in that study (described later in details in the annex), but this is indeed a very important aspect of violence against women in general terms.

Sethuraman 2001, described many clinical settings where patients are often considered as "non-persons". The setting we will work in study fits with this description. We will implement the intervention to improve the doctor-patient relationship and we will measure both the patient outcomes that is the woman's satisfaction, as well as the process (communicative behavior of the care provider) according to Ong's Model.

For peer review only

Methods

Participants:

Care providers including mainly residents working in the public maternity hospitals in Damascus and its surroundings. The hospitals are the Maternity Teaching hospital in Damascus University (15000 deliveries each year), Al-Zahrawi hospital, which is the largest Ministry of Health's hospital in Damascus (13000 deliveries each year), Douma hospital, which is a MOH district hospital in rural Damascus (7000 deliveries per year), Harasta hospital, a MOH district hospital in rural Damascus (5000 deliveries per year). Eligible participants in the intervention are residents registered for the year of field work in those hospitals. The total number of residents in the four hospitals is about 125 residents. They are distributed as follows: 75 in the Maternity Teaching Hospital; 24 in Al-Zahrawi Hospital; 12 in Douma Hospital; and 12 in Harasta Hospital.

Midwives were excluded from this study since they have no role in the delivery process, according to the directors at hospitals. As we work in teaching hospitals, we learnt that midwives are not allowed to participate in the delivery, to give the chance for residents to do all the work themselves; this is the training policy in those hospitals.

Women as potential beneficiaries from our intervention would include all women using the hospitals under study. We will target in our measurement all women with living babies who agreed to participate in the study, whether the delivery was normal vaginal or by C-section. Women using those hospitals are largely from middle and low socio-economic class.

Intervention:

The main and the only intervention in this study is the exposure of study participants (residents in the hospitals) to a training package in interpersonal and communication skills using a specially designed training package which will make sure that the key tasks in communication are covered using effective teaching methods (Maguire 2002). The training package will be developed by the study team in cooperation with experts in the field (one International expert and an Arab-speaking one). The training will be designed and presented as a package which is rather relevant to doctors in their future career. It will be advocated as

For peer review only

an essential training for doctors that teaches them the "how to" in the field of communication skill.

<u>Objectives:</u>

The main objective of this study is to test the effect of training residents in interpersonal and communication skills on women's satisfaction with patient-doctor relationship in labor and delivery rooms.

The secondary objective of this study is to measure the change in communicative behavior of the residents after the training in communication skills.

<u>Outcomes:</u>

Two outcomes will be measured in this research. Those are the women's satisfaction (short term outcome according to Ong model) as well as the communicative behaviour of doctors (a process indicator in Ong Model).

The main outcome of this study is the increased women's satisfaction with interpersonal relationships of doctors working in labor and delivery rooms. The secondary outcome is the positive change in communicative behavior of the care providers (residents) serving in labor and delivery rooms. This is also could be considered as a process evaluation, but we are hypothesizing that the training might not change the communicative behavior if constrained by environmental factors such as work load and time pressure.

The first outcome will be measured using a Likert scale questionnaire on women's satisfaction investigating the communication skills of residents attending her (See the attached in annex 1). The second outcome will be measured using an observation checklist (See the attached in annex 2). A specially designed questionnaire will also be used to collect data on potential factors that could determine the communicative behavior of the resident. (See the attached in annex 3).

Tools that were consulted to develop our tools in this study include: the patient satisfaction questionnaire, the MISS-21 (Meakin and Weinman, 2002). The observation checklist developed by Al-Galaa study in Egypt that was tested and validated (Sholkamy *et al*, 2003).

For peer review only

A validation exercise of the measurement tools is planned.. This will be carried out on a sample of 100 women and on 10 shifts of observation. It is planned to interview 100 women twice within a period of 15 days and also to have two observers in the same room during 10 shifts of observation

A baseline study will take place in all the hospitals to describe the communicative behavior of care-providers. Observation will be based on rooms (labour and delivery) rather than individual residents, throughout the shifts. This means that we are more interested in the service aspect rather than the actual change in the behavior of the resident; but also it means less vulnerability to problems that could arise if some one is under observation. Pre and post (training) measurements will be carried out for hospitals, based on scores collected from those who were observed in observation round, rather than on scores from each resident. It is also planned to repeat the observation two months as well 6 months after the intervention to evaluate waning of the intervention effect. The last part is an additional part of the study and not an actual part of it.

A baseline study will take place to collect data on women's satisfaction prior to the intervention. Then women will be recruited from all hospitals after each time point where the intervention is implemented in a randomly selected a hospital. Thus a total of five measurements of women's satisfaction are a must part of this study. In addition, it is planned to carry out one extra measurement 6 months after the last point of time of intervention. This will also allow an evaluation of the waning of the intervention.

Design:

A stepped wedge randomized design will be used in this study. This design involves sequential roll-out of an intervention to participants in our hospitals over a number of time periods. We embarked on this design for the following reasons: a) we are interested in measuring the impact of training at different points of time, thus to measure the sustainability of the effect or the waning of the intervention's effect. Stepped wedge designs offer a number of opportunities for data analysis, particularly for modeling the effect of time. Such an effect is expected, if "Training" was the intervention. b) Practically, it would be more practical to implement the intervention in stages to groups of doctors rather than having to deliver the intervention simultaneously for all the participants. c) Stepped Wedge designs as reviewed recently by Brown and Lilford (2006) are known for their ethical sensitivity. Although there

For peer review only

is no proof that our suggested intervention will work positively, it is expected that the intervention will do more good than harm, making this design more relevant.

All eligible doctors in the hospitals (clusters) included in this study (public hospitals in Damascus and its surrounding i.e., rural Damascus) will receive the intervention by the end of the study, but the intervention will be phased in over time, and thus the timing of the implementation of the training package in different hospitals will be randomized.

The design is illustrated in the following figure, where shaded cells represent the intervention periods and the blank cells represent the control periods. Timings in which each hospital will receive the intervention will be randomized.   Thus hospitals 1, 2, 3, and 4 in this illustration have no real order.   Their names will only be known after the randomization is done and communicated. It is expected that two months would be the time periods between intervention implementation and measurements. This estimate is based on the number of doctors in the hospitals as well as the number of women to be recruited each point of time for the outcome measurement.

**Hospital**	**Time**				
	1	2	3	4	5
1					
2					
3					
4					

Sample size

Based on the power calculation graphs presented in Annex 4 and the likelihood that a within-cluster analysis might be employed, we will consider a sample size of 100 per cluster (hospital) at a given time point. Therefore, the total sample size needed for the study is 2000 (= 100 women * 4 hospitals (clusters) * 5 (points of time)) not accounting for non-response rate. If we allow for a 10% non-response rate then the required sample size will be 2223 women. The details of power calculation are shown in annex 4.

For peer review only

Randomization:

The timing in which each hospital will receive the intervention (Stepping) will be randomized. A statistician who is not part of the study team will do the randomization. This will be done in a concealed manner as to prevent any problems from knowing in which hospital training would take place in the next stage.

Blinding

All observers and field workers will be blind to the objectives of the study. The objectives as well as the plan of analysis of the study will not be communicated with the observers or with the field workers.

Statistical Methods

The analysis plan will include the following activities:

a. Preliminary Analysis

A series of tables will be constructed which summarize the basic characteristics of the data collected. Examples of such tables are:

1. Tables that compare baseline characteristics at the individual, cluster level and at different time points will be provided. For continuous variables means and standard errors will be reported. Furthermore, for categorical variables numbers and percentages will be given. In addition, graphical presentations of the outcome variable will be plotted for a visual assessment of any trends within and between clusters.
2. Tables that describe the baseline demographic characteristics of the Physicians will be provided.
3. Tables that compare the scores of the Physicians prior and post training.
4. The intra-cluster correlation coefficient for the main outcome (possibly for other variables) will be reported.

b. Comparing the main outcome between intervention and control groups.

i. At the Hospital –level

For peer review only

Let Y_{ct} denote the mean of cluster *c* at time *t*. Based on equation (1) presented in Annex 4,

$$Y_{ct} = \mu + \alpha_c + \beta_t + I_{ct}\theta + \varepsilon_{ct} \qquad (3)$$

for c in 1, …, C, t in 1, …, T, and ε_{ct} are independently and identically distributed random errors that follow a $N(0,\sigma^2)$, see Hussey and Hughes (2007). Since the size of clusters is similar, then a Linear Mixed Model (LMM) approach will be used to investigate whether there is a treatment effect (H_0: $\theta = 0$). In addition, temporal changes in the response can be investigated by checking whether all the β_t's are significantly different than zero.

ii. **At the Individual-level**

A LMM can be used to estimate the parameters in equation (1) in Annex 4 and study their statistical significance. However, in case the responses are not normally distributed then a Generalized Estimating Equations (GEE), see Zeger and Liang (1986), and a Generalized Linear Mixed Model (GLMM), see McCullagh and Nelder (1989), will be used to estimate the above parameters. In case we decide to categorise the outcome variable, both GEE and GLMM can handle a categorical outcome.

Using GEE will require specifying the working correlation matrix, which will be estimated once the data is available. Other working correlation matrices will also be examined. However, it is known that GEE analysis is robust with respect to the choice of the correlation structure, see Diggle, Heagerty, Liang and Zeger (2002).

c. Adjusting for baseline characteristics and other covariates

Models (1) and (2) can be easily extended to account for baseline characteristics and other covariates including time-varying ones. Similar approaches to those outlined in part b. will be followed. Covariates will include the type of delivery whether normal or C-section.

d. Comparing the physicians' outcome pre and post intervention

Using a regression approach similar to that outlined above. Scores for the physicians will be compared pre and post intervention adjusting for clustering at the hospital level.

e. Software

The statistical analyses will be carried out using both the STATA package software (College Station, Texas) and the R language (www.r-project.org).

For peer review only

f. Missing data

In case of missing data, statistical tests will be carried out to assess whether the missing data are completely missing at random, missing at random, or informative. In addition, the data will be multiply imputed according to one or two available imputation techniques and the results will be compared, see Little and Rubin (2002).

Ethical considerations

The study protocol was submitted to the Institutional Review Board of the Damascus University and it was approved by them. Administrative approval will be taken from all hospital directors. Informed consent will be taken from both residents and all women participating in the study. We think that the choice of the design is relevant in terms of meeting the ethical obligation as to train all care providers. The informed consents are attached (annex 5).

Field work and practical issues

Preparatory work

The team did the following tasks in preparation of the proposal. Those tasks were carried out in 2005-2006.

1- Visited all the hospitals and got their preliminary approval. A formal letter will be sent at the start of the field work. All hospitals' directors received the idea very positively.
2- A small scale qualitative study was carried out to understand the context. A summary of the study is attached (Annex 6).
3- The observation checklist and the satisfaction questionnaire were tested in the field (See attached in Annex 7). Necessary modifications were made as well as time of implementation was calculated.

Data Collection

The study has two main phases; a preparatory one and the main intervention phase.

In the preparatory phase the following will be done:

1- Continue to meet the directors of hospitals to seek their support and explain the study objective and also to get the full description about the organization of the work in labor and

For peer review only

delivery rooms. Routine statistics will be reviewed as for the number of deliveries, number of residents and the job description of each one in terms of childbirth.

2- Prepare for the baseline survey in all hospitals. This survey will use the observation checklists but also will collect background information on the hospital and the workload of the doctors, relationship with colleagues and doctors and perception of the importance of communication skills. This background data will be analyzed by hospital and will not be linked with the observation checklist. Another survey on women satisfaction will be carried out at homes of women who gave birth at the hospitals.

3- Finalize the design of the training package with the help of international experts. The main principal investigator has already got in touch with Dr. Portela from WHO, who has developed a handbook to train care providers in communication skills and counseling as related to maternal and newborn care. The handbook and its accompanying training sessions were tested in developing countries settings. An Arab speaking expert will also be consulted (Dr. Tamar Kabakian from AUB has offered help in developing the training package), and the implementation of the intervention will be carried out by Arab speaking expert (Dr Tarif Bakdash from Syria who is a pediatrician with experience in communication skills teaching).

As for the main intervention phase the following will be done:

1- The randomization as to decide on the timing for the intervention implementation.

2- The implementation of the training. This will be carried out by the Arab speaking expert. The training will take place in three days. The training will be organized according to the number of trainees from doctors by the study team in cooperation with a focal point n each hospital named by the director. All residents in the study hospitals will be exposed to training, not necessary at a single point of time as the number of residents in some hospitals is very big to be trained at one time. The training will cover the following main themes:

- An overview of the doctor-patient communication
- Questioning skills
- Listening skills
- Non-verbal communication
- Answering skills
- Rapport building skills
- Counseling skills
- Skills of persuasion

Effective teaching methods will be used as suggested by Maguire and Pitceathly (2002).

The following steps will be carried out:

For peer review only

1- The Information Phase: Collecting evidence on deficiencies in communication skills (this will be based on the baseline observation)

2- The Sensitization Phase: Sensitizing the participants by using verbal and written examples to elicit their reaction.

3- The Practice Phase: Offering an opportunity to practice the skills under controlled conditions

4- The Feedback phase: Offering constructive feedback on the performance of participants.

3- measurement of outcomes: The observation will be carried out after the training in each hospital as well as 2 and 6 months afterwards. Rounds of observation will be done, where each round will include an observation in all rooms, for 3 days and for all shifts including day and night shifts. As for the women satisfaction questionnaire; this will be implemented as well in 4 points of times excluding the baseline. Nearly one hundred women will be recruited from each hospital. Also, future measurements at 2 and 6 months after the study are considered but will not be part of this study.

Problems anticipated

We are not very worried in this design about the spillover (contamination) effect. The reason for that is indeed the lack of opportunities for doctors from different hospitals to meet as the hospitals are separated by place as well as affiliation; and there is no common activities between them. So there is no need for a washout time between the intervention phases. However, we think that the management of the research will be difficult.

Importance of the study and expected outcomes

This study is important as it is the first to assess the effectiveness of training care providers in interpersonal and communication skills on women's satisfaction with the communicative behavior of doctors attending delivery in Syria. The study will not only provide evidence as to what is the effect of the training but also it will provide an opportunity to train residents in communication skills and of course will raise awareness among all concerned about the importance of this issue as a major part of better quality of care. It is hoped that the dissemination process that will follow this study will also give an opportunity as to communicate the matter with policy makers, academia and professional bodies about the great importance of communication skills in the field of maternity care.

For peer review only

Duration of the study

It is expected that the study will take about 18 months.

References

Bashour H, Abdulsalam A, Al-Faisal W and Cheikha S. The patterns and determinants of maternity care in Damascus, Syria. Eastern Mediterranean Health Journal (in Press).

Beck RS, Daughtridge R, Sloane PD. Physician-patient communication in the primary care office:a systematic review. J Am Board Fam Pract 2002;15(1):25-38.

Bingham D. Helping hospitals change. Part 2: Childbirth education techniques to empower women Journal of Perinatal education2005; 14: 51-55.

Brown CA and Lilford RJ. The stepped wedge trial design: a systematic review. BMC Medical Research Methodology 2006, 6:54.

Department of Health. Changing childbirth. Part 1. Report of the Expert Maternity Group. London: HMSO, 1993.

Department of Health. Changing childbirth. Part 2: Survey of good communication practice in maternity services. London: HMSO, 1993.

Diggle P.J., Heagerty P.J., Liang K.Y., and Zeger S.L. Analysis of longitudinal data. Second edition. Oxford University Press. 2002

Garcia J, Garforth S. Parents and new-born babies in the labour ward. In: Garcia J, Kilpatrick R, Richards M (eds.) The Politics of Maternity Care. Services for Childbearing Women in Twentieth-Century Britain. Oxford: Oxford University Press, 1990: 163-182.

Garcia J, Redshaw M, Fitzsimons B, Keene J. First Class Delivery. A National Survey of Women's Views of Maternity Care. London: Audit Commission, 1998.

Hodnett, E.D., Gates, S., Hofmeyr, G.J., & Sakala, C. Continuous support for women during childbirth (Cochrane Review). The Cochrane Library, 3. Oxford: Update Software, 2003.

Hussey A. and Hughes J.P. Design and Analysis of stepped wedge cluster randomized trials. Contemporary clinical trials 2007; 28:182-191.

Jenkins V, Fallowfield L. Can communication skills training alter physicians' beliefs and behavior in clinics? J Clin Oncol 2002;20(3):765-769.

For peer review only - http://bmjopen.bmj.com/site/about/guidelines.xhtml

Kabakian-Khasholian T, Campbell O, Shediac-Rizkallah M, Ghorayeb F. (2000) Women's experiences with maternity care: satisfaction or passivity? Social Science & Medicine, 51:103-113.

Little R.J.A. and Rubin D.B. Statistical Analysis with Missing data. Wiley & Sons. 2002

McCullagh, P. and Nelder J.A. Generalized Linear Models. Second edition. Chapman & Hall. 1989

Maguire P and Pitceathly C. Key communication skills and how to acquire them. BMJ 2002; 325:697-700.

Meakin R and Weinman J. The Medical Interview Satisfaction Scale (MISS-21) adapted for British general practice. Family Practice 2002; 19:257-263.

O' Cathain A, Walters SJ, Nicholl JP, Thomas KJ. Cluster randomized trial of Informed Choice leaflets. In: NHS Centre for Reviews and Dissemination Informed Choice in Maternity Care: an Evaluation of Evidence-Based Leaflets. York: NHS Centre for Reviews and Dissemination, University of York, 2001: 33-44.

Ong LML, de Haes JCJM, Hoos AM, Lammes FB. Doctor-patient communication: a review of the literature. Social Science and Medicine, 1995; 40: 903-918.

Rowe RE, Garcia J, Macfarlane AJ, et al. Improving communication between health professional and women in maternity care: a structured review. Health Expectations 2002; 5: 63-83.

Sethuraman KR. Communication skills in clinical practice. Jaypee 2001: 7.

Sholkamy H, Khalil K, Cherine M, Elnoury A, Breebaart M, Hassanein N. An observation checklist for facility-based normal labor and delivery practices: The Galaa Study. Monographs in Reproductive Health No. 5. Cairo; The Population Council, 2003.

Stewart MA. Effective physician-patient communication and health outcomes: a review. Canadian Medical Association Journal, 1995; 152: 1423-1433.

Zeger S.L. and Liang K.Y. Longitudinal data analysis using generalized linear models. Biometrika 1986; 73, 13-22.

For peer review only

Time frame

The following chart shows the time frame for the proposed study.

	Year 1												Year 2*							
	1	2	3	4	5	6	7	8	9	10	11	12	1	2	3	4	5	6	7	8
Preparatory	X	X																		
Base line survey (women And observation)			X	X																
Training 1					X															
Observation 1						X														
Women Survey1						X		X		X		X								
Training 2							X													
Observation 2								X												
Women Survey						X		X		X		X								
Training 3									X											
Observation 3										X										
Women Survey						X		X		X		X								
Training 4											X									
Observation 4												X								
Women Survey						X		X		X		X								
Data entry													X	X						
Data Analysis															X	X	X			
Writing and dissemination																	X	X		

For peer review only

* possible another measurement for women’s satisfaction and observation at 2 and 6 months but not as part of the project.

Estimated Budget

For peer review only

Appendices

1- **Questionnaire for Women's satisfaction with doctor's communication in Maternity hospitals (Arabic and English)**
2- **Observation Checklist (Arabic and English)**
3- ***Power calculation***
4- ***Summary from preparatory qualitative work***
5- ***Informed consents for women and doctors***

For peer review only

The effect of training doctors in communication skills on women's satisfaction with doctor-woman relationship during labour and delivery: a stepped wedge cluster randomised trial in Damascus

Journal:	*BMJ Open*
Manuscript ID:	bmjopen-2013-002674.R2
Article Type:	Research
Date Submitted by the Author:	02-Jul-2013
Complete List of Authors:	Bashour, Hyam; Faculty of Medicine, Damascus university ,<br>Kanaan, Mona; University of York, Department of Health Sciences<br>Kharouf, Mayada; Damascus University, Department of Family and Community Medicine<br>Abdulsalam, Asmaa; Damascus University, Department of Obstetrics and Gynecology<br>Tabbaa, Mohammed; Damascus University, Department of Obstetrics and Gynecology<br>Cheikha, Salah; Damascus University, Department of Obstetrics and Gynecology
**Primary Subject Heading**:	Obstetrics and gynaecology
Secondary Subject Heading:	Public health, Patient-centred medicine
Keywords:	Maternal medicine < OBSTETRICS, EPIDEMIOLOGY, Quality in health care < HEALTH SERVICES ADMINISTRATION & MANAGEMENT

For peer review only - http://bmjopen.bmj.com/site/about/guidelines.xhtml

The effect of training doctors in communication skills on women's satisfaction with doctor-woman relationship during labour and delivery: a stepped wedge cluster randomised trial in Damascus

Hyam N Bashour[1] *, Mona Kanaan[2], Mayada H Kharouf[1], AsmaaA Abdulsalam[3], Mohammed A Tabbaa[3], Salah A Cheikha[3]

[1]Department of Family and Community Medicine, Faculty of Medicine, Damascus University, Damascus, Syria,

[2] Department of Health Sciences, University of York, York, United Kingdom

[3]Departmentof Obstetrics and Gynaecology, Faculty of Medicine, Damascus University, Damascus, Syria

* Corresponding author: hbashour@scs-net.org

Details:

HNB: PhD, hbashour@scs-net.org

MK: PhD, mona.kanaan@york.ac.uk

MHK: MSc,mkharouf@scs-net.org

AAA: MD, asma@scs-net.org

MT: MD, mmtabbaa@hotmail.com

SAC:MD, senioreyad@hotmail.com

For peer review only - http://bmjopen.bmj.com/site/about/guidelines.xhtml

Abstract

Objectives: To determine the effect of training residents in interpersonal and communication skills on women's satisfaction with doctor-woman relationship in labour and delivery rooms

Design: Astepped wedge cluster randomised trial

Setting: Four tertiary-care teaching maternity hospitals in Damascus, Syria

Participants: Two thousand women who gave birth to a living baby in the four study hospitals and consented to participate in the intervention took part in the study. Women with difficult labour and high-risk pregnancies were excluded**.** All were interviewed at home after discharge.

Interventions: A specially designed training package in communication skills that was delivered to all resident doctors at the four hospitals.

Primary outcome measures: The main outcome measure was women's satisfaction with interpersonal relationships in labour and delivery rooms measured via a series of questions on a Likert scale modified from the Medical Interview Satisfaction Scale.

Results: At the individual level, the mean for the average satisfaction score was 3.23 (SD: 0.72) out of a possible score of a 5 in the control group and 3.42 (SD: 0.73) in the intervention group. Using generalised linear mixed models, we were not able to detect a difference between the mean for the average satisfaction score of women in the intervention arm and that of women in the control arm; the 95% confidence interval associated with the effect of the intervention ranged from-0.08 to 0.15.

Conclusions: In spite of the slight changes in the observed residents' communication skills, the training package in communication skills does not seem to be associated with higher satisfaction scores of women**.** This raises the question whether training individuals without further structural changes in the delivery of care and without further reinforcement of the training can have an impact on improving the quality of doctor-patient communication.

Trial Registration Number: ISRCTN80243969

For peer review only - http://bmjopen.bmj.com/site/about/guidelines.xhtml

For peer review only

Funding: Welcome Trust

Key words: Women's satisfaction, Communication skills, Training, Stepped Wedge Cluster Randomised trial

For peer review only

Article Summary

'Article Focus'

- The study aims to determine the effect of training residents in interpersonal and communication skills on women's satisfaction with the doctor-woman relationship in labour and delivery rooms
- The context of the trial focuses on teaching maternity hospitals with high patient volume
- There have been few RCTs testing interventions to improve maternal health care in the Arab region and in Syria in particular.

'Key Messages'

- In the context of a highly crowded and stressful environment where middle and low class Syrian women give birth, a specially designed training package in interpersonal and communication skills for residents did not achieve an overall improvement in women's satisfaction with the doctor-woman relationship in labour and delivery.
- This study raises questions as to whether training individuals without more structural changes in the delivery of care can have an impact on improving the quality of doctor-patient communication. These are important questions to address and call for further research.
- Despite the lack of evidence from this study, the need to improve the interpersonal skills of medical doctors and obstetricians specifically should be reinforced, as good communication is central to quality health care.

Strengths and Limitations of the Study

The key strength of the study was in its stepped-wedge cluster randomised design, which is known to be ethical and practical. The primary limitation of the study was its inability to link women's satisfaction, or dissatisfaction, with the behaviour of a specific doctor or specific doctors; for ethical and practical reasons, we observed actions rather than the behaviours of individual residents.. This was unavoidable due to the design's key considerations.

For peer review only - http://bmjopen.bmj.com/site/about/guidelines.xhtml
For peer review only

For peer review only

For peer review only - http://bmjopen.bmj.com/site/about/guidelines.xhtml

Background

There is a growing body of literature linking health providers' communication skills to a host of valued patient outcomes, including satisfaction, adherence, and positive health indicators [1]. As a result, communication in health-care, particularly between health-care professionals and patients, has attracted an increasing amount of attention at official and professional levels in recent years. Many academic and statutory bodies have been involved in initiatives to promote good communication including that for maternity care [2-3]. These initiatives are in part a response to increasing evidence from researchers that the quality of the interaction between patients and their care providers may have a significant effect on a variety of aspects of patient's well-being. These include satisfaction, knowledge and understanding, adherence with advice or treatment, quality of life, and psychological and other health outcomes [4-5].

Maternity care is an area of health-care in which the importance of good communication has received particular attention [3]. Studies of women's views of maternity care suggest that good communication is central in determining whether women are satisfied with the care that they receive or not; communication gives them the opportunity to be better informed and ask questions, and it signals understanding and respect[6-7]. Observational studies of communication between midwives and women during labour have highlighted the misunderstandings that can occur when communication is poor and have identified areas where communication could be improved [8].

Despite the acknowledged importance of communication in maternity care and the official recognition that communication is not always as good as it should be, there have been few evaluations of strategies to improve communication between women and their care providers. Previous reviews of doctor-patient communication have not included any studies carried out in maternity care [4-5]. The review carried out by Rowe in 2002 [9] aimed to fill in that gap and thus identified and reviewed trials of the effectiveness of interventions aimed at improving communication between health professionals and women in maternity care. The review identified trials largely in the area of antenatal care but also identified a major gap in knowledge relating to

For peer review only - http://bmjopen.bmj.com/site/about/guidelines.xhtml

communication in a number of key areas of labour, delivery and postnatal care. They recommended that trials of interventions to improve communication between carers and women in labour and in the postnatal period would be particularly useful. All the studies included in that review were carried out in developed countries, mainly in the United Kingdom.

Although good progress in reducing maternal mortality has been achieved in Syria; maternity care is still characterised by fragmentation of care, lack of protocols including those related to pain relief in labour, over-medicalisation of care, strong and even informal role of private sector, complex relationships between skilled attendants, and variation of quality of care received [10].

In Syria, previous work at both public hospitals and community level showed that continuous support of women during labour and delivery is virtually non-existing. Companionship is not allowed at hospitals, however, in a population-based study women reported the existence of a companion at delivery in 37% of the cases [11]. Syrian women expressed their dissatisfaction with being left alone and being attended by care providers who are not equipped with good communication skills. It was evident that Syrian women taking part in the study were left without any social and emotional support during a critical period in their lives. In most occasions, they were not allowed to be accompanied by their relatives when in labour and delivery. There were many instances in which doctors and midwives themselves were the source of dissatisfaction due to lack of respect for patients and discrimination which is mainly related to the patient's socioeconomic status [12].

Furthermore, labour and delivery care is largely provided in overcrowded hospitals mainly by medical graduates with little or no training in communication and interpersonal skills and where midwives and nurses roles are typically marginal. These poor conditions might well impact women's satisfaction in the delivery process. This is added to the fact that Syrian public hospital do not have a policy to administer pain relief; the low societal status of women especially those from lower socioeconomic backgrounds; and the restrictive relations between females and males outside the family context. For example, previous research findings showed that in the delivery suites, eye-to-eye contact is not acceptable if the gender of the service provider was male [12].

For peer review only - http://bmjopen.bmj.com/site/about/guidelines.xhtml

Therefore, the main objective of this trial was to evaluate the effect of training resident doctors, as the main providers of care in teaching public maternity hospitals, in interpersonal and communication skills on women's satisfaction with doctor-woman relationship in labour and delivery rooms using a modified version of the Medical Interview Satisfaction Scale(MISS-21) [13]. The secondary objective of this study was to measure the change in communicative behaviour of residents at labour and delivery rooms level using items of the Al-Galaa observational checklist[14].

For peer review only

For peer review only - http://bmjopen.bmj.com/site/about/guidelines.xhtml

Methods

Study design:

A stepped wedge cluster randomised design was used in this trial [15-16]. This particular design as reviewed by Brown and Lilford [16] involves sequential roll-out of the intervention to individuals or clusters over a number of time periods but the order in which participants receive the intervention is determined at random. In this trial, the roll-out of the training package was carried out at four time-points separated by two months each in addition to a baseline time-point; see supporting file one for a diagram of the trial design.

At baseline each hospital contributed a cluster to the control arm whereas at the last time-period each hospital contributed a cluster to the intervention arm. The timing of the implementation of the training package in each hospital determined when clusters switched from the control arm to the intervention arm. A coded list of the hospitals was produced and a statistician blinded to the coding allocated randomly the timing of the introduction of the intervention to each hospital. By the end of the study, all eligible resident doctors in the study hospitals received the intervention.

Participants:

Participants in this study included care providers and women delivering at four public maternity hospitals in Damascus and its surroundings, Syria. The study hospitals included three hospitals affiliated to the Ministry of Health (MOH) and one university teaching hospital. The hospitals were the Maternity Teaching hospital at Damascus University (15000 deliveries each year), Al-Zahrawi hospital, which is the largest MOH hospital in Damascus (13000 deliveries each year), Douma hospital, which is a MOH district hospital in rural Damascus (7000 deliveries per year), and Harasta hospital, a MOH district hospital in rural Damascus (5000 deliveries per year) [17].

Care providers to undertake the training intervention were all residents registered for the year of field work (2008-2009) in the four hospitals. Midwives were excluded from this study since they had no role in the delivery process, except for assisting doctors, according to policies at those hospitals.

For peer review only - http://bmjopen.bmj.com/site/about/guidelines.xhtml

Women were included if they had normal vaginal birth or by C-section and gave birth to a living baby at the four hospitals during the study period. Women using the study public hospitals come largely from middle and low socio-economic backgrounds.

Informed consents were obtained from residents and women participating in the study. The study protocol was approved by the Institutional Review Board at Damascus University. All study hospitals agreed to take part in this research.

Intervention

The intervention consisted of exposing all residents in the four study hospitals to a training package in interpersonal and communication skills, using a specially designed training package that has been developed by the study team in cooperation with an international expert in the field. The objectives of the training package were: to recognize the impact of effective communication on women and child health during labour and delivery; to identify characteristics and principles of effective communication; to recognize and be able to overcome barriers to effective communication; to enhance and reinforce the interpersonal communication skills of health providers, and improve their interactions with patients in general and women in labour in particular.

The content of the package included a story from a labour room. Issues regarding attitudes of health providers, overview of doctor-woman communication, non-verbal communication, building rapport, listening skills, effective interviewing, counselling and persuasion, and the ideal maternity ward were all explored. The methodology of the workshop was based on sharing concepts and ideas; self-assessment by means of tests and checklists; brainstorming, group work and plenary discussion; learning by experience; role-play; games and ice breakers. The training package is available for readers upon request.

All residents at the four hospitals received the intervention; their total number was 137. The training was carried out by a national trainer with experience in communication skills together with members of the research team who observed and facilitated the training. In total, nine training workshops took place at the Faculty of Medicine, Damascus University. The duration of each workshop was 20 hours in total, delivered over three days. The rate of daily attendance ranged from 78 to 100%. A formal evaluation of the training workshops

For peer review only - http://bmjopen.bmj.com/site/about/guidelines.xhtml

For peer review only

was carried out. The feedback from the evaluation was very positive with 97% of respondents saying that they would recommend the workshop. However, 82% envisaged barriers to implementation such as time pressure, overloaded work, and hospital's routine.

Data Collection

Data on women's satisfaction were collected prior to the intervention and at each instance of the four randomisation points from all hospitals. Data collection took place between April 2008 and January 2009. Participating women were interviewed at their homes within two weeks after delivery. Data were collected on demographic and socio-economic variables (age, education, work status, husband's age, husband's education, husband's occupation and home ownership), and about the pregnancy and delivery (whether the mother is multiparous/nulliparous, gender of the newborn, type of delivery), also women were asked to complete a modified version of the Medical Interview Satisfaction Scale [13].

Furthermore, at each randomisation point data were collected to describe the communicative behaviour of care-providers. Trained observers were asked to fill in a checklist recording observations of collective communicative behaviour of residents at the labour and delivery rooms rather than the individual residents throughout the different hospital shifts using the Al-Galaa checklist [14]. Three shifts were observed per day which amounted to a total of 24-hours observation of each delivery room at each point of time. We have chosen this approach as we were more interested in the change of the behaviour at the service level rather than the actual change in the behaviour of an individual resident. Furthermore, this approach was expected to reduce problems that could arise if residents were observed individually. Thus, scores from observation were collected at the level of the shift's round rather than scores for each resident. Pre- and post-training measurements were carried out in all four hospitals at each point of time, thus the observation was carried out on an average of two to three weeks after the implementation of the training package in each hospital, with some variance between large and small hospitals. The training took longer time at large hospitals because of the nature of the workshop and the necessity to implement it two to three times to cover all residents. Observers and field workers were blind to the specific objectives of the study.

For peer review only

For peer review only - http://bmjopen.bmj.com/site/about/guidelines.xhtml

Qualitative data collection using six focus group discussions also took place prior to the field work to serve the design phase of this project. The qualitative methods aimed to understand the work environment of the residents and get their views concerning the importance of designing a training package in communication skills.

Outcomes

The primary outcome of this study was women's satisfaction with interpersonal and communication skills of doctors working in labour and delivery rooms, which could be seen as a patient outcome in the Ong's model [4]. The theoretical model for doctor-patient/woman communication as suggested by Ong and colleagues (1995) is a useful model to conceptualize an interventional research that aims to improve communication. It consists of background variables, actual content of communication as process indicators as well as patient outcomes including satisfaction in the short time. Women's satisfaction was measured using a Likert scale questionnaire investigating the communication skills of residents attending her. The questionnaire was based on the patient satisfaction questionnaire MISS-21 [13, 18]; the adapted questionnaire is referred to as the Modified Medical Interview Satisfaction Scale (MMISS).The validation exercise of our measurement tool which was implemented in Arabic language is to be reported elsewhere. The questionnaire had a total of 21 questions looking at the communication skills of the doctors in our setup. Women were asked to indicate their levels of satisfaction with communicative practices of the attending resident on a 5-point Likert scale with 1 indicating strongly disagree to 5 indicating strongly agree. There were eight negatively worded questions; the codes of these questions were reversed for analyses purposes. A score was then calculated by summing up the women's answers to the MMISS questions except for the first one, which was a general satisfaction question. Therefore, the highest possible score was one hundred and the lowest was 20, where higher values indicated that women were satisfied with the services provided to them. An average score was then calculated by dividing the score by the number of questions. Two scenarios were investigated. In the first scenario, the average score was computed for participants who provided information for the twenty items on the MMISS questionnaire and

For peer review only - http://bmjopen.bmj.com/site/about/guidelines.xhtml

is referred to as the average score for complete MMISS. In the second scenario, the average score was computed for items with a response and is referred to as the average score only.

The secondary outcome measured the communicative behaviour of residents serving in labour and delivery rooms using sections related to communication of the observational checklist developed by Al-Galaa study in Egypt by Sholkamy and colleagues in 2003 [14]. The Al-Galaa study checklist was developed to document normal labour and delivery practices in an Egyptian hospital and covered areas such as management of labour, communication, postpartum and neonatal care; the instrument was tested and validated [14]. The checklist could be considered as a process evaluation in this work. It constituted of 31 questions and included items such as whether the attending doctor greeted the woman, identified himself/herself, explained the procedures of the medical examination he/she was about to undertake and communicated the findings, responded to the woman's questions, gave clear instructions about the different stages of labour/delivery and what was expected from the woman and whether the doctor gave any guidance with regard to post-delivery.

Sample size

Power calculations were carried out as in Hussey and Hughes (2007) [15]. A pilot study of 10 women resulted in a mean score of 3.165 (63.3 points on the satisfaction scale) and a standard deviation of 0.71. Based on a cluster size of a 100 at each step and hospital, a mean of 3.15 (63 points) and a standard deviation of 0.75 points, a difference of 0.2 (4 points) could be detected with 90% power given a significance level of 0.05. Therefore, the total sample size needed for the study was 2000 women (100 women x 4 hospitals x 5 time points) not accounting for non-response rate.

The total number of all residents in the four hospitals was 137. They were distributed as follows: 85 in the Maternity Teaching Hospital; 31 in Al-Zahrawi Hospital; 7 in Douma Hospital; and 14 in Harasta Hospital. They all received the intervention when it was offered at their respective hospitals.

Statistical methods

Baseline characteristics of the women were summarised using counts (percentages) for categorical variables and means (standard deviations (SD)) for continuous variables.

For peer review only - http://bmjopen.bmj.com/site/about/guidelines.xhtml

Statistical analysis was based on the principle of intention to treat. Comparison of the main and secondary outcomes between the control and intervention arms of the study was carried out at the individual level. A Generalized Linear Mixed Model (GLMM) was used to determine the size and direction of the difference between the control and intervention arms for the main and secondary outcomes. Estimates of the difference and 95% confidence intervals were calculated. All analyses were adjusted for clustering and time of the intervention. Additional analyses of the main outcome were conducted controlling for demographic variables. Furthermore, we used multiple imputations in order to address missing data.

Statistical analyses were carried out using STATA software (College Station, Texas) and the R language (www.r-project.org).

For peer review only - http://bmjopen.bmj.com/site/about/guidelines.xhtml

Results

A total of 2000 women delivering in the four hospitals were assessed for their satisfaction with doctor-woman relationship. Figure1 describes the flow of participants using a CONSORT diagram [19]. All women approached agreed to participate in the trial.

[Figure 1: here]

Table 1 shows the characteristics of the women by treatment arm. The mean age of the women was 25 years (SD: 6 years). Approximately 95% were housewives. Three quarters had primary education only, 13% were illiterate whereas 12% had high school or further education qualifications. However, there was a differential in the distribution between hospitals with the percent illiterate ranging from 3.6% to 23%. This reflected the fact that two of the hospitals served an urban population whereas the other two served a rural population. The husband's mean age was 31 years (SD: 6.76 years). The vast majority of husbands were self-employed (41%), followed by being a manual worker (35%), and clerk (18%). The distribution of the husband's education was similar to that of the woman's education. Almost half owned their house whereas the other half lived in shared accommodation. A quarter of the women were nulliparous and only 2 women had a C-section

[Table 1 here]

The percentage of women agreed or strongly agreed with the statement of overall satisfaction with doctors' communication skills during labour/delivery ranged from 51% to 83% between hospitals in the control arm and 58% to 85% between hospitals in the intervention arm. These percentages were higher in the intervention arm for all hospitals except for Hospital 2 which decreased from 83% to 67% in the intervention arm. For presentation purposes, the hospitals were coded such that Hospital 1 represented the hospital that had the intervention delivered first and so forth.

For peer review only - http://bmjopen.bmj.com/site/about/guidelines.xhtml
For peer review only

Table 2 details the views of women on specific questions with regard to doctors' communication skills. The majority of women pointed-out that doctors did not identify themselves prior to the medical examination; the percentages were similar between the control and intervention arm. One third of the women in the intervention arm agreed/strongly agreed that the doctors' greeted them at the onset of the consultation compared to a fifth in the control arm. Around 40% of the women agreed/strongly agreed that the doctor looked at them when he/she talked to the woman in the control arm compared to 60% in intervention arm. Similar percentages were observed for whether the doctor listened to the woman with concern and without interruption. Almost 40% of the women agreed/strongly agreed that the doctor showed an interest in them in the control arm versus a third in the intervention arm. Forty-two percent of the women in the intervention arm agreed/strongly agreed that the doctor used some terms to calm them down and used his/her hands to assist/reassure them compared with 36% and 32% in the control arm, respectively. A small percentage reported that the doctor insulted them (6% control arm versus 3% intervention arm) or shouted/screamed at them (13% control arm versus 7% intervention arm). Only a quarter of the women agreed/strongly agreed that the doctor explained the examination steps before the clinical exams in the control arm compared to 29% in the intervention arm. A third agreed/strongly agreed that the doctor explained the results from the clinical exam in the control arm compared to a 41% in the intervention arm, however, Hospitals 1 and 2 had a decrease in the percentages who agreed/strongly agreed whereas Hospitals 3 and 4 had an increase. Only a quarter of those who responded to the questions whether the doctors explained alternative choices or helped the woman in making decisions agreed/strongly agreed to these statements.

[Table 2 here]

Table 3 provides summary statistics for the intervention and control arm for the two outcome scores: average score for MMISS and average score for complete MMISS. The median and interquartile range (Q1,Q3) score for the average score was 3.3 (2.8,3.7) in the control arm and 3.3 (3.0, 4.1) in the intervention arm; see Appendix for a graphical representation of the average score. Furthermore, the median and interquartile range

For peer review only - http://bmjopen.bmj.com/site/about/guidelines.xhtml

score for the average score of the complete MMISS was 3.4 (3.0, 3.9) in the control arm and 3.3 (3.1, 4.1) in the intervention arm (See additional file 2 for a graphical representation of the average score for complete MMISS).

[Table 3 here]

Regression estimates for the average score on intervention and time using GLMM are given in Table 3. The effect of the intervention was an average increase of 0.03 points; however, there was no evidence for statistical significance (95% CI: -0.08, 0.15). The estimates using complete MMISS implied that an average decrease of 0.13 in the intervention arm compared to the control arm (equivalent to 2.6 points on the original scale). Nevertheless, the effect was not statistically significant with a 95% CI (-0.29, 0.04).We also carried additional analyses adjusting for the woman's demographic characteristics. The results of the intervention effect were similar to the main analysis and none of the demographic variables was a significant predictor. We also used multiple imputations to account for missing data. The estimates of this analysis (data not shown) were similar to those reported here.

Table 4 reports on the secondary outcome, the observational checklist, and gives the numbers and percentages of when an item on the observational checklist, concerning communication between doctor and woman during labour/delivery, was not observed. Based on Table 4, the majority of doctors did not identify themselves, did not explain the steps of labour and delivery, did not explain to the woman the stages of labour and her role in it, and did not give any instructions about the steps that follow delivery or how to care for her new born. In nine out of the 31 items, the proportion of negative responses was consistently lower in the intervention arm compared to the control arm across the hospitals; data shown in supplementary file. In the intervention arm the proportion of doctors who called the woman by her name or title increased from 75% to 85%, those who asked for permission to examine the woman from 39% to 51%, and before the vaginal examination from 38% to 58%

For peer review only - http://bmjopen.bmj.com/site/about/guidelines.xhtml

compared to the control arm. The practice of covering the woman during delivery increased in the intervention arm to 81% from 41% though there were variations across hospitals; asking the woman to bend her knees increased to 85% from 61%; relaying information to other doctors in the team increased to 83% from 54%; and congratulated the woman on her delivery increased to 60% from 47% in the control arm.

Using GLMM regression, scores of the observational checklist at the shift level were compared pre and post intervention adjusting for clustering at the hospital level. This showed no evidence of statistical significance with an estimate of -0.01 (95% CI: -0.03, 0.02) for the intervention parameter.

[Table 4here]

Discussion

Maternity care is an area of healthcare in which the importance of good communication has received particular attention as good communication skills are essential for high quality, effective and safe medical practice [1-3].This trial was designed to determine the effectiveness of training residents at maternity wards in interpersonal and communication skills on women's satisfaction with doctor-woman relationship in labour and delivery rooms in four teaching hospitals in Damascus. Syrian women are largely delivered by medical graduates with little or no training in communication and interpersonal skills. Results of this trial did not provide evidence that the intervention improved women's satisfaction as measured by a modified version of the Medical Interview Satisfaction Scale. Communicative behaviour of the residents measured by an observational checklist was poor before the intervention and showed a slight improvement though not statistically significant after training on some selected items such as asking the woman's permission for the exam or asking her to bend her knees. Even violent behaviours were also noted and reported such as screaming and shouting.

The reasons for those negative findings of our trial could be multifaceted. First, we have no reason to think that the negative findings are due to measurement errors having validated the tool and trained the field workers.

For peer review only - http://bmjopen.bmj.com/site/about/guidelines.xhtml

However, the large numbers of care providers at maternity wards including midwives, nurses and junior doctors and even cleaners in the wards could have played a role on the overall satisfaction of women, given that midwives and nurses were not part of our target group for training as their role is mainly to assist doctors rather than taking a full charge of delivery. Furthermore, the policy implemented at Syrian public hospitals of not administering pain relief might have also confound the impact of the intervention on satisfaction as women would definitely prefer it [11]. The training package was tailored to our setting and was informed by previous research findings; it allowed for Ong's useful model to conceptualize an interventional research that aimed to improve communication and it ensured that the key tasks in communication are covered using effective teaching methods [4, 20]. However, lack of opportunity to reinforce the training could have played a negative role. Brown and colleagues suggested that communication skills programmes may need to be longer, more intensive, teach a broader range of skills, provide ongoing performance feedback, utilize patient feedback and use a variety of instruments to measure change in communication skills [21]. Furthermore, the working environment in these maternity wards is well characterized by long hours, crowded wards, high volume of patients, pressure due to organizational hierarchy at hospital, and tension in work relationships between residents, nurses and midwives [22]. All those factors could have left our target group of residents under pressure, but more importantly could have left women feeling unsatisfied. A recent article by Berridge et al (2010) highlighted how poor communication in the delivery suites can compromise safe and efficient care and humane relationships [23].

The interpretation of the findings should also consider the women's status in a community in which the expectations of the women are very low but also where culture and gender issues are critical. Previous research findings showed that some means of communication, for example eye-to-eye communication, are not acceptable if gender of the provider is different from that of woman [12]. We also argue that the overall positive satisfaction of women in our observation can be due to the childbirth event by itself as being a pleasing experience as well as getting the service free of charge in public hospitals by those women who come from a poor socio-economic class [12]. It was surprising to see that the very positive evaluation of the training

For peer review only - http://bmjopen.bmj.com/site/about/guidelines.xhtml

sessions by the trainees was not translated to improved satisfaction of the women. However, we fully support Bingham [24] who stressed the importance of carrying out patient satisfaction surveys to help hospitals change or improve their childbirth policy.

Our study enrolled all public teaching maternity hospitals in Damascus. Therefore, our findings can be generalisable to overcrowded public delivery settings in Syria and to settings in developing countries with similar characteristics of over crowdedness, over worked residents who are not trained in good communication skills and where the role of nurses and midwives is marginal. These findings suggest that training residents only without a structural change in the system and without further reinforcement might not be sufficient to improve women's satisfaction with the service provided.

Strengths and limitations

The key strength of the study was in its stepped-wedge cluster randomised design characterized by being ethical and practical [16], which resulted in that the training package being delivered to all residents serving at the hospital thus complementing their medical training in communication skills that is missing from their curriculum. Furthermore, the study had a 100% response rate among women but there were some missing values on some of the questions. High response rate is very common in our culture. Conducting the trial in a developing country certainly contributes to the field as compared to the literature that comes mainly from developed countries [1-3, 9]. The primary limitation of the study was its inability to link women's satisfaction, or dissatisfaction, with the behaviour of a specific doctor or specific doctors; for ethical and practical reasons, we observed actions rather than the behaviours of individual residents. This is unavoidable due to the design's key considerations that all hospitals would receive the intervention eventually. Residents are the sole providers of care at labour and delivery in the teaching public hospitals in our setting and midwives are only marginally involved in care. Therefore, exclusions of nurses and midwives in this study should not be a major limitation in the current setting. The relatively short duration of the training and the lack of follow up within the hospital setting could have contributed to the negative findings in this study. Furthermore, the selection of public

For peer review only - http://bmjopen.bmj.com/site/about/guidelines.xhtml

For peer review only

hospitals only in this study might have impacted the generalisability of the findings to other settings; however this setting is common in our region [25].

Conclusions

In the context of a highly crowded and stressful environment where middle and low class Syrian women deliver, a specially designed training package in interpersonal and communication skills for residents did not achieve an overall improvement in women's satisfaction with doctor-woman relationship in labour and delivery rooms. However, certain items in doctors' behaviour have improved. It would be worth investigating whether the package would improve women's satisfaction in less stressful settings, but also it is worth looking at other possible interventions in maternity care practice such as doctor-midwife collaboration or attendance of birth companion in such settings.

Despite the lack of evidence from this study, the need to improve the interpersonal skills of medical doctors and obstetricians specifically should be reinforced, as good communication is central to quality health care [26].

Competing Interest

The authors declare no competing interests.

Authors' contributions

HNB and AAA developed the study concept and aims then initiated the project. MKH, MT, SAC and MK assisted further in the refinement of the concept and development of the protocol. HNB, MKH and AAA oversaw collection of data. MK did the statistical analysis. HNB and MK drafted the manuscript. All authors read and approved the final manuscript.

For peer review only - http://bmjopen.bmj.com/site/about/guidelines.xhtml

Acknowledgments

Our thanks are due to Mrs Hoda Hallab who worked with the team to develop the training package and deliver the training at inception. We are also grateful to the directors of the hospitals for facilitating the field work. The effort of Nick Bashour in reviewing the English language of this paper is highly appreciated.

This paper is part of a larger regional research project on Choices and Challenges in Changing Childbirth in the Arab Region sponsored by the Center for Research on Population and Health at the American University of Beirut, Lebanon, with generous support from the Wellcome Trust (UK)[Grant Ref:074986].

For peer review only

For peer review only - http://bmjopen.bmj.com/site/about/guidelines.xhtml

References

1- Teutsch C. **Patient-doctor communication.** Med Clin North Am 2003;**87**:1115-45.

2- Department of Health. Changing childbirth. Part 1. Report of the Expert Maternity Group. London: HMSO, 1993.

3- Department of Health. Changing childbirth. Part 2: Survey of good communication practice in maternity services. London: HMSO, 1993.

4- Ong LML, de Haes JCJM, Hoos AM, Lammes FB. **Doctor-patient communication: a review of the literature**. Soc Sci Med1995; **40**: 903-918.

5- Stewart MA. **Effective physician-patient communication and health outcomes: a review**. CanMedAssJ 1995; **152**: 1423-1433.

6- Garcia J, Redshaw M, Fitzsimons B, Keene J. First Class Delivery. A National Survey of Women's Views of Maternity Care. London: Audit Commission, 1998.

7- Kabakian-Khasholian T, Campbell O, Shediac-Rizkallah M, Ghorayeb F. (2000) **Women's experiences with maternity care: satisfaction or passivity?** Soc SciMed, **51**:103-113.

8- Garcia J, Garforth S. Parents and new-born babies in the labour ward. In: Garcia J, Kilpatrick R, Richards M (eds.) The Politics of Maternity Care. Services for Childbearing Women in Twentieth-Century Britain. Oxford: Oxford University Press, 1990: 163-182.

9- Rowe RE, Garcia J, Macfarlane AJ, et al. **Improving communication between health professional and women in maternity care: a structured review**. Health Expect2002; **5**: 63-83.

10-DeJong J, Bashour H, Kaddour A. Women's Health: progress and unaddressed issues. In: Jabbour S, Giacaman R, Khawaja M, Nuwayhid I (eds) Public Health in the Arab World. Cambridge University press 2012:

11- Bashour H, Abdulsalam A, Al-Faisal W, Cheikha S. **The patterns and determinants of maternity care in Damascus, Syria**. East Mediterr Health J. 2008; **14**: 398-405.

For peer review only - http://bmjopen.bmj.com/site/about/guidelines.xhtml

12- Hafez R, Bashour H, Abdulsalam A. Childbirth and its discontent: Experiences of Syrian Women. A paper presented at the Annual meeting of Reproductive Health Working Group (RHWG), Lebanon, July 11-13,2004.

~~1516~~ 13- Meakin R and Weinman J. **The Medical Interview Satisfaction Scale (MISS-21) adapted for British general practice**. Fam Pract 2002; **19**:257-263.

~~17 18~~ 14- Sholkamy H, Khalil K, Cherine M, Elnoury A, Breebaart M, Hassanein N. An observation checklist for facility-based normal labour and delivery practices: The Galaa Study. Monographs in Reproductive Health No. 5. Cairo; The Population Council, 2003.

15- Hussey A. and Hughes J.P. **Design and Analysis of stepped wedge cluster randomized trials**. Contemp Clin Trials2007; **28**:182-191.

~~14~~ 16- Brown CA and Lilford RJ. **The stepped wedge trial design: a systematic review**. BMC Med Res Methodol2006, **6**:54.

~~15~~- 17 Syrian Ministry of Health. www.moh.gov.sy

18- Holloway S, Porteous M, Cetnarskyj R, Anderson E, Rush R, Fry A, Gorman D, Steel M, Campbell H. 2004. **Patient satisfaction with two different models of cancer genetic services in south-east Scotland**. *Br J Cancer***90**: 582–589.

19-Moher D, Hopewell S, Schulz KF, Montori V, Gøtzsche PC, Devereaux PJ, Elbourne D, Egger M, Altman DG, for the CONSORT Group. CONSORT 2010 Explanation and Elaboration: updated guidelines for reporting parallel group randomised trial. BMJ 2010;340:c869.

20- Maguire P and Pitceathly C**. Key communication skills and how to acquire them**. BMJ 2002; **325**:697-700.

21- Brown JB, Boles M, Mullooly JP, Levinson W.**Effect of clinician communication skills training on patient satisfaction. A randomized, controlled trial**. Ann Intern Med 1999; **131**:822–9.

22- Abdulasalam A. **Communication Skills! What does it mean to doctors and women: A qualitative study in Damascus maternity teaching hospitals**. Damascus University Journal for Health Sciences 2009;**25**:189-203. (In Arabic)

For peer review only

23- Berridge E, Mackintosh N and Freeth D. **Supporting patient safety: examining communication within delivery suite teams through contrasting approaches to research observation**. Midwifery 2010; **26**: 512-519.

24- Bingham D. **Helping hospitals change. Part 2: Childbirth education techniques to empower women**. JPerinatal Edu 2005; **14**: 51-55.

25- Choices and Challenges in Changing Childbirth Research Network. **Routines in facility-based maternity care: evidence from the Arab World**. BJOG: an International Journal of Obstetrics and Gynaecology 2005; **112**, 1270-1276.

26- Audit Commission. What seems to be the matter: Communication between hospitals and patients. HMSO, London. 1993.

For peer review only

For peer review only - http://bmjopen.bmj.com/site/about/guidelines.xhtml

Figures:

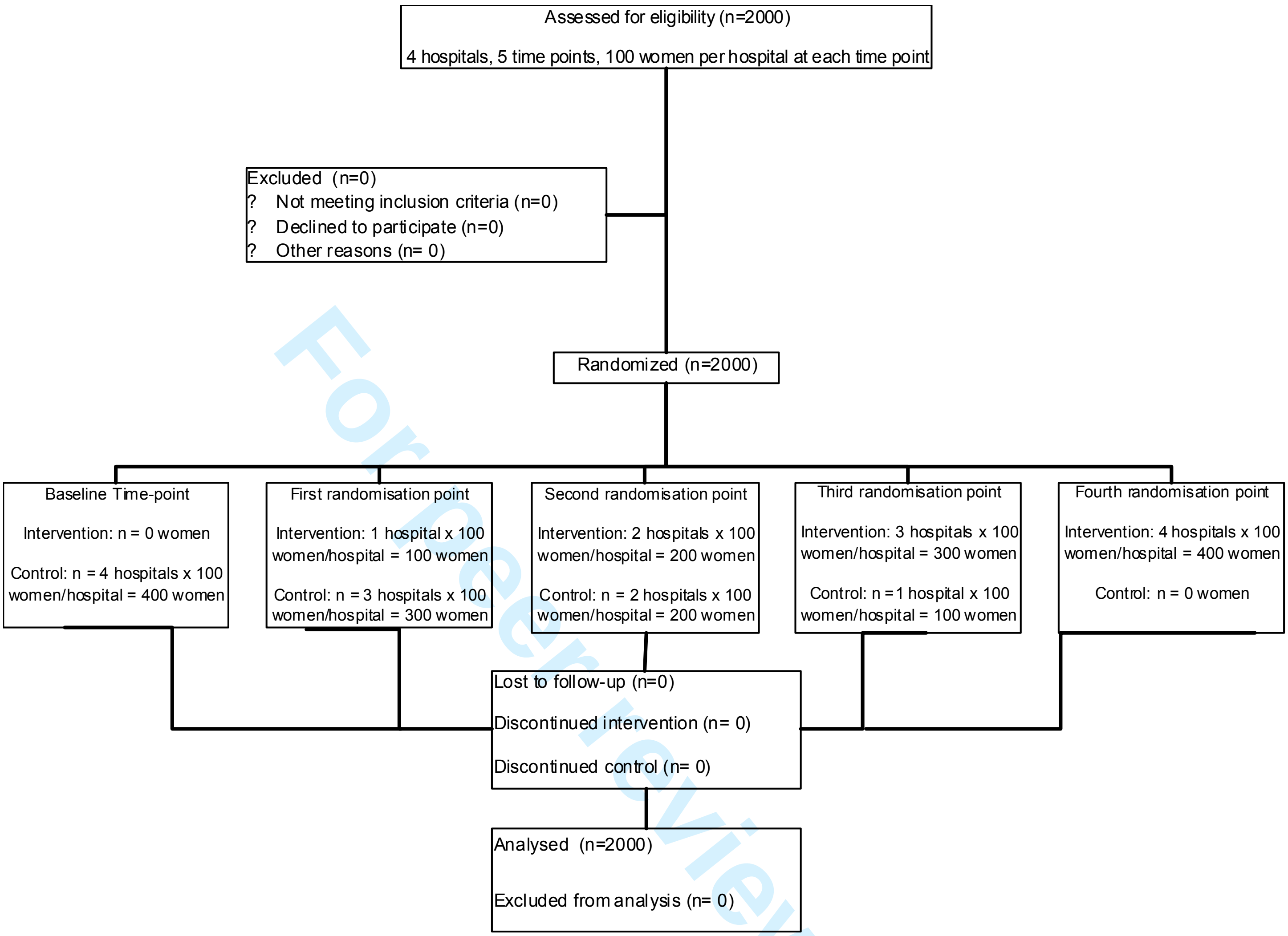

For peer review only

Figure 1: Flow of Participants

Tables:

For peer review only

For peer review only - http://bmjopen.bmj.com/site/about/guidelines.xhtml

Table 1: Demographic characteristics of the women by intervention arm. Numbers represent counts (percentage) unless otherwise stated.

	Control N = 1000	Intervention N = 1000		Control N = 1000	Intervention N = 1000
Mean age (SD)	25.02 (6.11)	25.10 (5.89)	Woman's Education		
Occupation: Housewife	949 (95)	953 (95)	Illiterate/Read and Write	140 (14)	102 (10)
Home Ownership: Share	488 (49)	470 (48)	Primary	745 (74)	761 (77)
Parity: Nulliparous	266 (27)	263 (27)	High School +	110 (11)	128 (13)
New born gender: Male	507 (52)	480 (49)	Husband's Education		
Mean husband's age (SD)	30.90 (6.91)	30.85 (6.62)	Illiterate/Read and Write	113 (11)	101 (10)
Husband's Occupation			Primary	781 (79)	743 (76)
Labourer (manual worker)	359 (36)	337 (34)	High School +	100 (10)	135 (14)
Clerk	160 (16)	198 (20)			
Self-employed	391 (40)	432 (44)			
Other	80 (8)	21 (2)			

For peer review only

Table 2: Numbers (percentages) of women who agreed/strongly agreed with various questions concerning their labour/delivery experience. The numbers compare all the intervention clusters to the control clusters.*

Item	Control Period N=1000	Intervention Period N=1000	Item	Control Period N=1000	Intervention Period N=1000
Overall satisfaction with doctors' communication skills during labour/delivery	619 (62)	713 (72)	Did the doctor listen to you with concern and without interrupting you?	413 (46)	551 (58)
Did the doctors identify themselves?	132 (13)	118 (12)	Was the doctor engaged in other issues, so you felt being unattended and annoyed?	165 (17)	67 (7)
Did the doctor greet you?	208 (21)	323 (32)	Did you feel let down by the way he/she was dealing with you?	66 (7)	31 (3)
Did the doctor look at you when talking to you?	434 (43)	599 (60)	Did the doctor explain all the steps before doing the clinical exam?	249 (25)	278 (29)
Did the doctor show interest in you as a person?	393 (39)	329 (33)	Did the doctor explain the findings from the clinical exam?	326 (34)	399 (41)
Did the doctor insult you?	55 (6)	29 (3)	Were you annoyed by the doctor explaining all the findings?	40 (5)	73 (10)
Did the doctor use humour to comfort you?	78 (8)	118 (12)	Did the doctor explain all the alternative choices to you?	176 (23)	145 (17)
Did the doctor use any terms to calm you down?	363 (36)	419 (42)	Did the doctor help you in making decisions?	175 (29)	158 (23)
Did the doctor use his/her hands to assist you or to reassure you?	314 (32)	409 (42)	Was the doctor annoyed from your questions and avoided answering them?	122 (14)	54 (6)
Did the doctor shout or scream at you?	125 (13)	71 (7)	Did the doctor use medical terms in explanation, thus they were not understood?	77 (8)	48 (5)

* Due to missing information some of the percentages do not tie up with the total number given.

For peer review only

Table 3: Summary statistics for the outcome of women satisfaction computed as the average score and average score for Complete MMISS.Regression analysis estimates for the average score and average score for complete MMISS on intervention and time controlling for clustering by hospital using GLMM. Estimates are presented together with 95% confidence interval (CI) limits as subscripts (${}_{Lower}Est_{Upper}$).

	Average Score	Average Score Complete MMISS
Control: Median(Q1, Q3)	3.3(2.8,3.7)	3.4(3.0,3.9)
Intervention: Median(Q1, Q3)	3.3(3.0, 4.1)	3.3(3.1, 4.1)
GLMM Estimates	${}_{Lower}Est_{Upper}$	${}_{Lower}Est_{Upper}$
**Fixed-effects**		
intervention	${}_{-0.08}0.03_{0.15}$	${}_{-0.29}-0.13_{0.04}$
time: 2	${}_{-0.08}0.02_{0.12}$	${}_{-0.10}0.05_{0.19}$
time: 3	${}_{-0.14}-0.03_{0.08}$	${}_{-0.02}0.12_{0.27}$
time: 4	${}_{-0.08}0.05_{0.18}$	${}_{0.12}0.30_{0.49}$
time: 5	${}_{-0.15}0.01_{0.16}$	${}_{-0.03}0.17_{0.37}$
constant	${}_{3.16}3.32_{3.48}$	${}_{3.23}3.44_{3.64}$
**Random-effects**	Variance(SE)	Variance(SE)
**Hospital**	0. 02 (0.016)	0.03 (0.026)
**Residual**	0.51 (0.016)	0.45 (0.02)

For peer review only

Table 4: Numbers (and percentages) of negative responses for items on the observational checklist concerning communication between doctor and woman during labour/delivery, unless otherwise stated. Due to missing information some of the percentages do not tie up with the total number given. The columns compare the numbers all the intervention clusters to the control clusters.

	Control Period N = 565	Intervention Period N=631		Control Period N=565	Intervention Period N=631
Mean (SD) of the average score	0.34 (0.13)	0.39 (0.10)			
Mean (SD) of the average score for complete checklist	0.34 (0.10)	0.39 (0.09)			
Checklist Item			Checklist Item		
Did the doctor identify himself/herself?	539 (100)	621 (99)	Was the woman asked any questions at this stage?	168 (32)	94 (15)
Did the doctor call the woman by her name?	132 (24)	99 (16)	Did the woman ask any questions at this stage?	292 (56)	281 (46)
Did the doctor take the woman's permission to examine her?	326 (61)	305 (49)	Did the doctor respond to her queries?	275 (63)	342 (64)
Did the doctor explain the plan he/she will follow?	497 (92)	577 (92)	Was the doctor responsive to the woman's pain?	285 (59)	333 (57)
Before starting the vaginal examination, did the doctor			Was the woman encouraged at this stage?	186 (40)	239 (42)
take her permission for the exam?	335 (62)	262 (42)	Was the woman told about the proximity of labour?	242 (55)	363 (66)
close the door?	472 (88)	568 (91)	Was the woman told about the stages of labour?	399 (95)	533 (98)
cover the woman during the exam?	315 (59)	116 (18)	Was the woman told about her role during labour?	347 (83)	508 (94)
ask the woman to bend her knees?	210 (39)	79 (13)	During delivery, was the woman asked to push?	26 (6)	23 (4)
After completing the vaginal examination, did the doctor			Did they explain to the woman when and how to push?	237 (57)	230 (42)
tell the woman the results of the exam?	418 (78)	504 (81)	Did the woman ask any questions at this stage?	284 (68)	296 (55)
explain the next steps to be followed?	486 (91)	586 (94)	Did the doctor respond to her queries?	264 (77)	330 (72)
relay the findings to other team members?	241 (45)	102 (16)	Did the doctor inform the mother about her baby's status?	307 (75)	386 (72)
relay the findings to the midwives/nurses?	393 (80)	579 (93)	Was the woman congratulated after the delivery?	217 (53)	214 (40)
Did the doctor give instructions about eating and drinking?	463 (87)	516 (83)	Was the woman instructed about the process after the delivery?	392 (95)	515 (96)
Did the doctor give instructions about movement?	335 (63)	428 (70)	Was the woman instructed about how to care for her newborn?	409 (99)	534 (99)

For peer review only

For peer review only - http://bmjopen.bmj.com/site/about/guidelines.xhtml

Appendix:

Figure 2 gives the distribution of the average score by treatment arm.

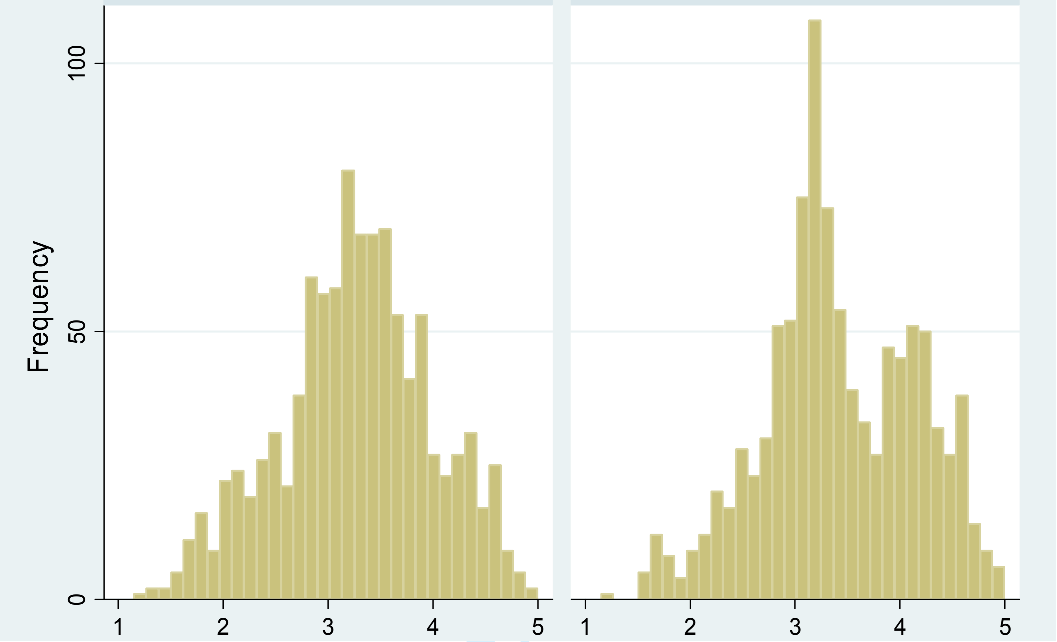

(a) (b)

Figure 2: Distribution of the average score by treatment arm (a) Control and (b) Intervention.

For peer review only - http://bmjopen.bmj.com/site/about/guidelines.xhtml

Description of supporting files

Additional File one: **Diagram of the study design**

Additional File two: **A graphical representation of the overall satisfaction outcome score of the women stratified by hospital and time of intervention. Data presented are for complete MMISS only**

Additional File three: **Results by intervention period.**

Additional File four**: Trial Protocol.**

For peer review only

For peer review only - http://bmjopen.bmj.com/site/about/guidelines.xhtml

The effect of training doctors in communication skills on women's satisfaction with doctor-woman relationship during labour and delivery~~satisfaction with labour and delivery~~: a stepped wedge cluster randomised trial in Damascus

Formatted: Font: (Default) Arial, 16 pt

Formatted: Font: (Default) Arial, 16 pt

Hyam N Bashour[1] *, Mona Kanaan[2], Mayada H Kharouf[1], AsmaaA Abdulsalam[3], Mohammed A Tabbaa[3], Salah A Cheikha[3]

[1]Department of Family and Community Medicine, Faculty of Medicine, Damascus University, Damascus, Syria,

[2] Department of Health Sciences, University of York, York, United Kingdom

[3]Departmentof Obstetrics and Gynaecology, Faculty of Medicine, Damascus University, Damascus, Syria

* Corresponding author: hbashour@scs-net.org

Details:

HNB: PhD, hbashour@scs-net.org

MK: PhD, mona.kanaan@york.ac.uk

MHK: MSc, mkharouf@scs-net.org

AAA: MD, asma@scs-net.org

MT: MD, mmtabbaa@hotmail.com

SAC:MD, senioreyad@hotmail.com

For peer review only

Abstract

Objectives: To determine the effect of training residents in interpersonal and communication skills on women's satisfaction with doctor-woman relationship in labour and delivery rooms.

Design: Astepped wedge cluster randomised trial

Setting: Four tertiary-care teaching maternity hospitals in Damascus, Syria

Participants: Two thousand women who gave birth to a living baby in the four study hospitals and consented to participate in the intervention took part in the study. Women with difficult labour and high-risk pregnancies were excluded. All were interviewed at home after discharge.

Interventions: A specially designed training package in communication skills that was delivered to all resident doctors at the four hospitals.

Primary outcome measures: The main outcome measure was women's satisfaction with interpersonal relationships in labour and delivery rooms measured via a series of questions on a Likert scale modified from the Medical Interview Satisfaction Scale.

Results: At the individual level, the mean for the average satisfaction score was 3.23 (SD: 0.72) out of a possible score of a 5 in the control group and 3.42 (SD: 0.73) in the intervention group. Using generalised linear mixed models, we were not able to detect a difference between the mean for the average satisfaction score of women in the intervention arm and that of women in the control arm; the 95% confidence interval associated with the effect of the intervention ranged from-0.08 to 0.15.

Conclusions: In spite of the slight changes in the observed residents' communication skills, the training package in communication skills does not seem to be associated with higher satisfaction scores of women. This raises the question whether training individuals without further structural changes in the delivery of care and without further reinforcement of the training can have an impact on improving the quality of doctor-patient communication.

Trial rRegistration Number: ISRCTN80243969

For peer review only

Funding: Wellcome Trust

Key words: Women's satisfaction, Communication skills, Training, Stepped Wedge Cluster Randomised trial

For peer review only

For peer review only - http://bmjopen.bmj.com/site/about/guidelines.xhtml

Article Summary

'Article ~~f~~Focus'

- The study aims to determine the effect of training residents in interpersonal and communication skills on women's satisfaction with the doctor-woman relationship in labour and delivery rooms
- The context of the trial focuses on ~~busy~~ teaching maternity hospitals with high patient volume
- There have been few RCTs testing interventions to improve maternal health care in the Arab region and in Syria in particular.

~~There have been few RCTs testing~~

~~interventions to improve maternal health care in the Arab region and in Syria in particular~~

'Key ~~m~~Messages'

- In the context of a highly crowded and stressful environment where middle and low class Syrian women give birth ~~deliver~~, a specially designed training package in interpersonal and communication skills for residents did not achieve an overall improvement in women's satisfaction with the doctor-woman relationship in labour and delivery.~~with labour and delivery.~~
- This study raises questions as to whether training individuals without more structural changes in the delivery of care can have an impact on improving the quality of doctor-patient communication. These are important questions to address and call for further research.
- Despite the lack of evidence from this study, the need to improve the interpersonal skills of medical doctors and obstetricians specifically should be reinforced, as good communication is central to quality health care.

Strengths and ~~l~~Limitations of ~~this~~ the ~~s~~Study

The key strength of the study was in its stepped-wedge cluster randomised design, ~~characterized~~ which is known to be ~~by being~~ ethical and practical. The primary limitation of the study~~One of the study limitations is in~~ was its inability to ~~study the change inbeing unable to~~ link women's satisfaction, or dissatisfaction,

Formatted: Justified, Space After: 6 pt

Formatted: Font: 12 pt

Formatted: List Paragraph, Bulleted + Level: 1 + Aligned at: 0.25" + Indent at: 0.5"

Formatted: Font: 12 pt

Formatted: Font: 12 pt

Formatted: Font: (Default) +Headings CS (Times New Roman), English (U.S.)

Formatted: Font: (Default) +Headings CS (Times New Roman), English (U.S.)

Formatted: Font: (Default) +Headings CS (Times New Roman), English (U.S.)

For peer review only - http://bmjopen.bmj.com/site/about/guidelines.xhtml

with the behaviour of a specific doctor or specific doctors ~~with a specific doctor's behaviour relating to doctor-womanrelationship as~~ —; for ethical and practical reasons, we observed actions rather than the behaviours of individual residents ~~individual resident's behaviour~~. ~~for ethical as well as practical reasons~~. This was ~~is~~ unavoidable due to the design's key considerations.

For peer review only - http://bmjopen.bmj.com/site/about/guidelines.xhtml

Background

There is a growing body of literature linking health providers' communication skills to a host of valued patient outcomes, including satisfaction, adherence, and positive health indicators [1]. As a result, communication in health-care, particularly between health-care professionals and patients, has attracted an increasing amount of attention at official and professional levels in recent years. Many academic and statutory bodies have been involved in initiatives to promote good communication including that for maternity care [2-3]. These initiatives are in part a response to increasing evidence from researchers that the quality of the interaction between patients and their care providers may have a significant effect on a variety of aspects of patient's well-being. These include satisfaction, knowledge and understanding, adherence with advice or treatment, quality of life, and psychological and other health outcomes [4-5].

Maternity care is an area of health-care in which the importance of good communication has received particular attention [3]. Studies of women's views of maternity care suggest that good communication is central in determining whether women are satisfied with the care that they receive or not; as communication gives them the opportunity to be better informed and, ask questions, and it signals understanding and respect[6-7]. Observational studies of communication between midwives and women during labour have highlighted the misunderstandings that can occur when communication is poor at this time and have identified areas where communication could be improved [8].

Despite the acknowledged importance of communication in maternity care and the official recognition that communication is not always as good as it should be, there have been few evaluations of strategies to improve communication between women and their care providers. Previous reviews of doctor-patient communication have not included any studies carried out in maternity care [4-5]. The review carried out by Rowe in 2002 [9] aimed to fill in that gap and thus identified and reviewed trials of the effectiveness of interventions aimed at improving communication between health professionals and women in maternity care. The review identified trials largely in the area of antenatal care but also identified a major gap in knowledge relating to

For peer review only - http://bmjopen.bmj.com/site/about/guidelines.xhtml

communication in a number of key areas of labour, delivery and postnatal care. They recommended that trials of interventions to improve communication between carers and women in labour and in the postnatal period would be particularly useful. All the studies included in that review were carried out in developed countries, mainly in the United Kingdom.

Although good progress in reducing maternal mortality has been achieved in Syria; maternity care is still characterised by fragmentation of care, lack of protocols including those related to pain relief in labour, over-medicalisation of care, strong and even informal role of private sector, complex relationships between skilled attendants, and variation of quality of care received [10].

In Syria, ~~-~~previous work ~~in~~ at both public hospitals and ~~at the~~ community level showed that continuous support of women during labour and delivery is virtually non-existing. Companionship is not allowed at hospitals, however, ~~-~~in a population~~-~~-based study~~,~~ women reported the existence of a companion at delivery in 37% of the cases [11]. Syrian women expressed their dissatisfaction with being left alone and ~~with~~ being attended ~~to~~ by care providers who are not equipped with good communication skills. It was evident that Syrian women taking part in the study were left without any social and emotional support during a critical period in their lives. In most occasions, they were not allowed to be accompanied by their relatives when in labour and delivery. There were many instances in which doctors and midwives themselves were the source~~s~~ of dissatisfaction due to lack of respect for patients and discrimination which is mainly related to the patient's socioeconomic status [12].

Furthermore, labour and delivery care is largely provided in overcrowded hospitals mainly by medical graduates with little or no training in communication and interpersonal skills and where midwives and nurses roles are typically marginal. These poor conditions might well impact ~~on~~ women's satisfaction in the delivery process. This is added to the fact that ~~in addition to the following issues:~~ Syrian public hospital do not have a policy to administer ~~policies ofnot administering~~ pain relief; the low societal status of women especially those from lower socioeconomic backgrounds; and the restrictive relations between females and males outside the family context. For example, previous research findings showed that in the delivery suites, ~~-~~eye-to-eye contact is not acceptable if the gender of the service provider was ~~is~~ male [12].

For peer review only - http://bmjopen.bmj.com/site/about/guidelines.xhtml

Therefore, the main objective of this trial was to evaluate the effect of training resident doctors, as the main providers of care in teaching public maternity hospitals, in interpersonal and communication skills on women's satisfaction with doctor-woman relationship in labour and delivery rooms using a modified version of the Medical Interview Satisfaction Scale(MISS-21) [13]. The secondary objective of this study was to measure the change in communicative behaviour of residents at labour and delivery rooms level using items of the Al-Galaa observational checklist[14].

For peer review only

Methods

Study design:

A stepped wedge cluster randomised design was used in this trial [15-16]. This particular design as reviewed by Brown and Lilford [16] involves sequential roll-out of the intervention to individuals or clusters over a number of time periods but the order in which participants receive the intervention is determined at random. In this trial, the roll-out of the training package was carried out at four time-points separated by two months each in addition to a baseline time-point; see supporting file one for a diagram of the trial design.

~~This resulted in observing 10 clusters under the control armand another 10 clusters under the intervention arm. Each hospital contributed5 clusters of participants.~~ At baseline each hospital contributed a cluster to the control arm whereas at the last time-period each hospital contributed a cluster to the intervention arm. The timing of the implementation of the training package in each hospital determined when clusters ~~from a hospital~~ switched from the control arm to the intervention arm. A coded list of the hospitals was produced and a statistician blinded to the coding allocated randomly the timing of the introduction of the intervention to each hospital. By the end of the study, all eligible resident doctors in the study hospitals received the intervention.

Participants:

Participants in this study included care providers and women delivering at four public maternity hospitals in Damascus and its surroundings, Syria. The study hospitals included three hospitals affiliated to the Ministry of Health (MOH) and one university teaching hospital. The hospitals were the Maternity Teaching hospital at Damascus University (15000 deliveries each year), Al-Zahrawi hospital, which is the largest MOH hospital in Damascus (13000 deliveries each year), Douma hospital, which is a MOH district hospital in rural Damascus (7000 deliveries per year), and Harasta hospital, a~~n~~ MOH district hospital in rural Damascus (5000 deliveries per year) [17].

For peer review only - http://bmjopen.bmj.com/site/about/guidelines.xhtml

Care providers to undertake the training intervention were all residents registered for the year of field work (2008-2009) in the four hospitals. Midwives were excluded from this study since they had no role in the delivery process, except for assisting doctors, according to policies at those hospitals.

Women were included if they had normal vaginal birth or by C-section and gave birth to a living baby at the four hospitals during the study period. Women using the study public hospitals come largely from middle and low socio-economic backgrounds.

Informed consents were ~~as~~ obtained from residents and women participating in the study. The study protocol was approved by the Institutional Review Board ~~of~~ at Damascus University. All study hospitals agreed to take part in this research.

Intervention

The intervention consisted of exposing all residents in the four study hospitals to a training package in interpersonal and communication skills, using a specially designed training package that has been developed by the study team in cooperation with an international expert in the field. The objectives of the training package were: to recognize the impact of effective communication on women and child health during labour and delivery; to identify characteristics and principles of effective communication; to recognize and be able to overcome barriers to effective communication; ~~and~~ to enhance and reinforce the interpersonal communication skills of health providers, and improve their interactions with patients in general and women in labour in particular.

The content of the package included a story from a labour room. Issues regarding attitudes of health providers, overview of doctor-woman communication, non-verbal communication, building rapport, listening skills, effective interviewing, counselling and persuasion, and the ideal maternity ward were all explored. The methodology of the workshop was based on sharing concepts and ideas; self-assessment by means of tests and checklists; brainstorming, group work and plenary discussion; learning by experience; role-play; games and ice breakers. The training package is available for readers upon request.

For peer review only

All residents at ~~in~~ the four hospitals received the intervention; their total number was 137. The training was carried out by a national trainer with experience~~d~~ in communication skills together with members of the research team who observed and facilitated the training. In total, nine training workshops took place at the Faculty of Medicine, Damascus University. The duration of each workshop was 20 hours in total, delivered over three days. The rate of daily attendance ranged from 78 to 100%. A formal evaluation of the training workshops was carried out. The feedback from the evaluation was very positive with 97% of respondents saying that they would recommend the workshop. However, 82% envisaged barriers to implementation such as time pressure, overloaded work, and hospital's routine.

Data Collection

Data on women's satisfaction were ~~as~~ collected prior to the intervention and at each instance of the four randomisation points from all hospitals. Data collection took place between April 2008 and January 2009. Participating women were interviewed at their homes within two weeks after delivery. Data were collected on demographic and socio-economic variables ~~information~~ (age, education, work status, husband's age, husband's education, husband's occupation and home ownership), ~~information~~ and about the pregnancy and delivery (whether the mother is multiparous/nulliparous, gender of the newborn, type of delivery), also ~~and~~ women were asked to complete a modified version of the Medical Interview Satisfaction Scale [13].

Furthermore, at each randomisation point data were collected to describe the communicative behaviour of care-providers. Trained observers were asked to fill in a checklist recording observations of collective communicative behaviour of residents at the labour and delivery rooms ~~level~~ rather than the individual residents throughout the different hospital shifts using the Al-Galaa checklist [14]. Three shifts were observed per day which amounted to a total of 24~~-~~ hours observation of each delivery room at each point of time. We have chosen this approach as we were more interested in the change of the behaviour at the service level rather than the actual change in the behaviour of an individual resident. Furthermore, this approach was expected to reduce problems that could arise if residents were observed individually. ~~–~~Thus, scores from observation were collected at the level of ~~a~~ the shift's round rather than scores for each resident. Pre- and post-training

For peer review only
For peer review only - http://bmjopen.bmj.com/site/about/guidelines.xhtml

measurements were carried out in all four hospitals at each point of time, thus the observation was carried out on an average of two to three weeks after the implementation of the training package in each hospital, with some variance between large and small hospitals. The training took longer time at ~~in the~~ large hospitals because of the nature of the workshop and the necessity to implement it two to three times to cover all residents. Observers and field workers were blind~~ed~~ to the specific objectives of the study.

Qualitative data collection ~~through~~ using six focus group discussions ~~was~~ also ~~carried~~ took place ~~out~~ prior to the field work to serve the design phase of this project. The qualitative methods aimed to understand the work environment of the residents and get their views concerning the importance of designing a training package in communication skills.

Outcomes

The primary outcome of this study was women's satisfaction with interpersonal and communication skills of doctors working in labour and delivery rooms, which could be seen as a patient outcome in the Ong's model [4]. The theoretical model for doctor-patient/woman communication as suggested by Ong and colleagues (1995) is a useful model to conceptualize an interventional research that aims to improve communication. It consists of background variables, actual content of communication as process indicators as well as patient outcomes including satisfaction in the short time. Women's satisfaction was measured using a Likert scale questionnaire investigating the communication skills of residents attending her. The questionnaire was based on the patient satisfaction questionnaire MISS-21 [13, 18]; the adapted questionnaire is referred to as the Modified Medical Interview Satisfaction Scale (MMISS).The validation exercise of our measurement tool which was implemented in Arabic language is to be reported elsewhere. The questionnaire had a total of 21 questions looking at the communication skills of the doctors in ~~this~~ our setup. Women were asked to indicate their levels of satisfaction with communicative practices of the attending resident on a 5-point Likert scale with 1 indicating strongly disagree to 5 indicating strongly agree. There were ~~8~~ eight negatively worded questions; the codes of these questions were reversed for analyses purposes. A score was then calculated by summing up the women's answers to the MMISS questions except for the first one, which was a general satisfaction

For peer review only

question. Therefore, the highest possible score was one hundred ~~a 100~~ and the lowest was 20, where higher values indicated that women were satisfied with the services provided to them. An average score was then calculated by dividing the score by the number of questions. Two scenarios were investigated. In the first scenario, the average score was computed for participants who provided information for the twenty items on the MMISS questionnaire and is referred to as the average score for complete MMISS. In the second scenario, the average score was computed for items with a response and is referred to as the average score only.

The secondary outcome measured the communicative behaviour of residents serving in labour and delivery rooms using sections related to communication of the observational checklist developed by Al-Galaa study in Egypt by Sholkamy and colleagues in 2003 [14]. The Al-Galaa study checklist was developed to document normal labour and delivery practices in an Egyptian hospital and covered areas such as management of labour, communication, postpartum and neonatal care; the instrument was tested and validated [14]. The checklist could be considered as a process evaluation in this work. It constituted of 31 questions and included items such as whether the attending doctor greeted the woman, identified himself/herself, explained the procedures of the medical examination he/she was about to undertake and communicated the findings, responded to the woman's questions, gave clear instructions about the different stages of labour/~~,~~ delivery and what ~~is~~ was expected from the woman and whether the doctor gave any guidance with regard to post-delivery.

Sample size

Power calculations were carried out as in Hussey and Hughes (2007) [15]. A pilot study of 10 women resulted in a mean score of 3.165 (63.3 points on the satisfaction scale) and a standard deviation of 0.71. Based on a cluster size of a 100 at each step and hospital, a mean of 3.15 (63 points) and a standard deviation of 0.75 points, a difference of 0.2 (4 points) could be detected with 90% power given a significance level of 0.05. Therefore, the total sample size needed for the study was 2000 women (100 women x 4 hospitals x 5 time points) not accounting for non-response rate.

For peer review only

For peer review only - http://bmjopen.bmj.com/site/about/guidelines.xhtml

The total number of all residents in the four hospitals was 137. They were distributed as follows: 85 in the Maternity Teaching Hospital; 31 in Al-Zahrawi Hospital; 7 in Douma Hospital; and 14 in Harasta Hospital. They all received the intervention when it was offered at their respective hospitals.

Statistical methods

Baseline characteristics of the women were summarised using counts (percentages) for categorical variables and means (standard deviations (SD)) for continuous variables.

Statistical analysis was based on the principle of intention to treat. Comparison of the main and secondary outcomes between the control and intervention arms of the study was carried out at the individual level. A Generalized Linear Mixed Model (GLMM) was used to determine the size and direction of the difference between the control and intervention arms for the main and secondary outcomes. Estimates of the difference and 95% confidence intervals were calculated. All analyses were adjusted for clustering and time of the intervention. Additional analyses of the main outcome were conducted controlling for demographic variables. Furthermore, we used multiple imputations in order to address missing data.

Statistical analyses were carried out using STATA software (College Station, Texas) and the R language (www.r-project.org).

For peer review only

For peer review only - http://bmjopen.bmj.com/site/about/guidelines.xhtml

Results

A total of 2000 women delivering in the four hospitals were assessed for their satisfaction with ~~patient~~doctor-woman relationship. Figure1 describes the flow of participants using a CONSORT diagram [19]. All women approached agreed to participate in the trial.

[Figure 1: here]

Table 1 shows the characteristics of the women by treatment arm. The mean age of the women was 25 years (SD: 6 years). Approximately 95% were housewives. Three quarters had primary education only, 13% were illiterate whereas 12% had high school or further education qualifications. However, there was a differential in the distribution between hospitals with the percent illiterate ranging from 3.6% to 23%. This reflected the fact that two of the hospitals served an urban population whereas the other two served a rural population. The husband's mean age was 31 years (SD: 6.76 years). The vast majority of husbands were self-employed (41%), followed by being a manual worker (35%), and clerk (18%). The distribution of the husband's education was similar to that of the woman's education. Almost half owned their house whereas the other half lived in shared accommodation. A quarter of the women were nulliparous and only 2 women had a C-section

[Table 1 here]

The percentage of women agreed or strongly agreed with the statement of overall satisfaction with doctors' communication skills during labour/delivery ranged from 51% to 83% between hospitals in the control arm and 58% to 85% between hospitals in the intervention arm. These percentages were higher in the intervention arm for all hospitals except for Hospital 2 which decreased from 83% to 67% in the intervention arm. For presentation purposes, the hospitals were coded such that Hospital 1 represented the hospital that had the intervention delivered first and so forth.

For peer review only

For peer review only - http://bmjopen.bmj.com/site/about/guidelines.xhtml

Table 2 details the views of women on specific questions with regard to doctors' communication skills. The majority of women pointed-out that doctors did not identify themselves prior to the medical examination; the percentages were similar between the control and intervention arm. One third of the women in the intervention arm agreed/strongly agreed that the doctors' greeted them at the onset of the consultation compared to a fifth in the control arm. Around 40% of the women agreed/strongly agreed that the doctor looked at them when he/she talked to the woman in the control arm compared to 60% in intervention arm. Similar percentages were observed for whether the doctor listened to the woman with concern and without interruption. Almost 40% of the women agreed/strongly agreed that the doctor showed an interest in them in the control arm versus a third in the intervention arm. Forty-two percent of the women in the intervention arm agreed/strongly agreed that the doctor used some terms to calm them down and used his/her hands to assist/reassure them compared with 36% and 32% in the control arm, respectively. A small percentage reported that the doctor insulted them (6% control arm versus 3% intervention arm) or shouted/screamed at them (13% control arm versus 7% intervention arm). Only a quarter of the women agreed/strongly agreed that the doctor explained the examination steps before the clinical exams in the control arm compared to 29% in the intervention arm. A third agreed/strongly agreed that the doctor explained the results from the clinical exam in the control arm compared to a 41% in the intervention arm, however, Hospitals 1 and 2 had a decrease in the percentages who agreed/strongly agreed whereas Hospitals 3 and 4 had an increase. Only a quarter of those who responded to the questions whether the doctors explained alternative choices or helped the woman in making decisions agreed/strongly agreed to these statements.

[Table 2 here]

Table 3 provides summary statistics for the intervention and control arm for the two outcome scores: average score for MMISS and average score for complete MMISS. The median and interquartile range (Q1,Q3) score for the average score was 3.3 (2.8,3.7) in the control arm and 3.3 (3.0, 4.1) in the intervention arm; see Appendix for a graphical representation of the average score. Furthermore, the median and interquartile range

For peer review only - http://bmjopen.bmj.com/site/about/guidelines.xhtml

score for the average score of the complete MMISS was 3.4 (3.0, 3.9) in the control arm and 3.3 (3.1, 4.1) in the intervention arm (See additional file 2 for a graphical representation of the average score for complete MMISS).

[Table 3 here]

Regression estimates for the average score on intervention and time using GLMM are given in Table 3. The effect of the intervention was an average increase of 0.03 points; however, there was no evidence for statistical significance (95% CI: -0.08, 0.15). The estimates using complete MMISS implied that an average decrease of 0.13 in the intervention arm compared to the control arm (equivalent to 2.6 points on the original scale). Nevertheless, the effect was not statistically significant with a 95% CI (-0.29, 0.04).We also carried additional analyses adjusting for the woman's demographic characteristics. The results of the intervention effect were similar to the main analysis and none of the demographic variables was a significant predictor. We also used multiple imputations to account for missing data. The estimates of this analysis (data not shown) were similar to those reported here.

Table 4 reports on the secondary outcome, the observational checklist, and gives the numbers and percentages of when an item on the observational checklist, concerning communication between doctor and woman during labour/delivery, was not observed. Based on Table 4, the majority of doctors did not identify themselves, did not explain the steps of labour and delivery, did not explain to the woman the stages of labour and her role in it, and did not give any instructions about the steps that follow delivery or how to care for her new born. In nine out of the 31 items, the proportion of negative responses was consistently lower in the intervention arm compared to the control arm across the hospitals; data shown in supplementary file. In the intervention arm the proportion of doctors who called the woman by her name or title increased from 75% to 85%, those who asked for permission to examine the woman from 39% to 51%, and before the vaginal examination from 38% to 58%

For peer review only - http://bmjopen.bmj.com/site/about/guidelines.xhtml

compared to the control arm. The practice of covering the woman during delivery increased in the intervention arm to 81% from 41% though there were variations across hospitals; asking the woman to bend her knees increased to 85% from 61%; relaying information to other doctors in the team increased to 83% from 54%; and congratulated the woman on her delivery increased to 60% from 47% in the control arm.

Using GLMM regression, scores of the observational checklist at the shift level were compared pre and post intervention adjusting for clustering at the hospital level. This showed no evidence of statistical significance with an estimate of -0.01 (95% CI: -0.03, 0.02) for the intervention parameter.

[Table 4here]

Discussion

Maternity care is an area of healthcare in which the importance of good communication has received particular attention as good communication skills are essential for high quality, effective and safe medical practice [1-3].This trial was designed to determine the effectiveness of training residents at maternity wards in interpersonal and communication skills on women's satisfaction with doctor-woman relationship in labour and delivery rooms in four teaching hospitals in Damascus. Syrian women are largely delivered by medical graduates with little or no training in communication and interpersonal skills. Results of this trial did not provide evidence that the intervention improved women's satisfaction as measured by a modified version of the Medical Interview Satisfaction Scale. Communicative behaviour of the residents measured by an observational checklist was poor before the intervention and showed a slight improvement though not statistically significant after training on some selected items such as asking the woman's permission for the exam or asking her to bend her knees. Even violent behaviours were also noted and reported such as screaming and shouting.

The reasons for those negative findings of our trial could be multifaceted. First, we have no reason to think that the negative findings are due to measurement errors having validated the tool and trained the field workers.

For peer review only - http://bmjopen.bmj.com/site/about/guidelines.xhtml

However, the large numbers of care providers at maternity wards including midwives, nurses and junior doctors and even ~~servants~~ cleaners in the wards could have played a role on the overall satisfaction of women, given that midwives and nurses were not part of our target group for training as their role is mainly to assist doctors rather than taking a full charge of delivery. Furthermore, the policy implemented at Syrian public hospitals of not administering pain relief might have ~~could~~ also confound the impact of the intervention on satisfaction as women would definitely prefer it [11]. The training package was tailored to our setting and was informed by previous research findings; it allowed for Ong's useful model to conceptualize an interventional research that aimed~~s~~ to improve communication and it ensured that the key tasks in communication are covered using effective teaching methods [4, 20]. However, lack of opportunity to reinforce the training could have played a negative role. Brown and colleagues suggested that communication skills programmes may need to be longer, more intensive, teach a broader range of skills, provide ongoing performance feedback, utilize patient feedback and use a variety of instruments to measure change in communication skills [21]. Furthermore, the working environment in these maternity wards is well characterized by long hours, crowded wards, high volume of patients, pressure due to organizational hierarchy at hospital, and tension in work relationships between residents, nurses and midwives [22]. All those factors could have left our target group of residents under pressure, but more importantly could have left women feeling unsatisfied. A recent article by Berridge et al (2010) highlighted how poor communication in the delivery suites can compromise safe and efficient care and humane relationships [23].

The interpretation of the findings should also consider the women's status in a community ~~within~~ in which the expectations of the women are very low but also where culture and gender issues are critical. Previous research findings showed that some means of communication, for example eye-to-eye communication, are not acceptable if gender of the provider is different from that of woman [12]. We also argue that the overall positive satisfaction of women in our observation can be due to the childbirth event by itself as being a pleasing experience as well as ~~to~~ getting the service free of charge in public hospitals by those women who come from a ~~rather~~ poor socio-economic class [12]. It was surprising to see that the very positive evaluation of the training

For peer review only

sessions by the trainees ~~did~~ was not translated to improved satisfaction of the women. However, we fully support Bingham [24] ~~that~~ who stressed the importance of carrying out patient satisfaction surveys to help hospitals change or improve their childbirth policy ~~[24]~~.

Our study enrolled all public teaching maternity hospitals in Damascus. Therefore, our findings can be generalisable to overcrowded public delivery settings in Syria and to ~~in~~ settings in developing countries with similar characteristics of over crowdedness, over worked residents who are not trained in good communication skills and where the role of nurses and midwives is marginal. These findings suggest that training residents only without a structural change in the system and without further reinforcement might not be sufficient to improve women's satisfaction with the service provided.

Strengths and limitations

The key strength of the study was in its stepped-wedge cluster randomised design characterized by being ethical and practical [16], which resulted in that the training package being delivered to all residents serving at the hospital thus complementing their medical training in communication skills that is missing from their curriculum. Furthermore, the study had a 100% response rate among women but there were some missing values on some of the questions. High response rate is very common in our culture. Conducting the trial in a developing country certainly contributes to the field as compared to the literature that comes mainly from developed countries [1-3, 9]. The primary limitation of the study was its inability to link women's satisfaction, or dissatisfaction, with the behaviour of a specific doctor or specific doctors; for ethical and practical reasons, we observed actions rather than the behaviours of individual residents. ~~One of the study limitations is being unable to link women satisfaction with a specific doctor's behaviour of doctor-woman relationshipas we observed actions rather than individual resident's behaviour for ethical as well as practical reasons.~~ This is unavoidable due to the design's key considerations that all hospitals would receive the intervention eventually. Residents are the sole providers of care at labour and delivery in the teaching public hospitals in our setting and midwives are only marginally involved in care. Therefore, exclusions of nurses and midwives in this study should not be a major limitation in the current setting. The relatively short duration of the training and the lack

For peer review only

of follow up within the hospital setting could have contributed to the negative findings in this study. Furthermore, the selection of public hospitals only in this study might have impacted the generalisability of the findings to other settings; however this setting is common in our region [25].

Conclusions

In the context of a highly crowded and stressful environment where middle and low class Syrian women deliver, a specially designed training package in interpersonal and communication skills for residents did not achieve an overall improvement in women's satisfaction with doctor-woman relationship in labour and delivery rooms~~satisfaction with labour and delivery~~. However, certain items in doctors' behaviour have improved. It would be worth investigating whether the package would improve women's satisfaction in less stressful settings, but also it is worth looking at other possible interventions in maternity care practice such as doctor-midwife collaboration or attendance of birth companion in such settings.

Despite the lack of evidence from this study, the need to improve the interpersonal skills of medical doctors and obstetricians specifically should be reinforced, as good communication is central to quality health care [26].

Competing Interest

The authors declare no competing interests.

Authors' contributions

HNB and AAA developed the study concept and aims then initiated the project. MKH, MT, SAC and MK assisted further in the refinement of the concept and development of the protocol. HNB, MKH and AAA oversaw collection of data. MK did the statistical analysis. HNB and MK drafted the manuscript. All authors read and approved the final manuscript.

For peer review only

Acknowledgments

Our thanks are due to Mrs Hoda Hallab who worked with the team to develop the training package and deliver the training at inception. We are also grateful to the directors of the hospitals for facilitating the field work. The effort of Nick Bashour in reviewing the English language of this paper is highly appreciated.

This paper is part of a larger regional research project on Choices and Challenges in Changing Childbirth in the Arab Region sponsored by the Center for Research on Population and Health at the American University of Beirut, Lebanon, with generous support from the Wellcome Trust (UK)[Grant Ref:074986].

For peer review only - http://bmjopen.bmj.com/site/about/guidelines.xhtml

References

1- Teutsch C. **Patient-doctor communication.** Med Clin North Am 2003;**87**:1115-45.

2- Department of Health. Changing childbirth. Part 1. Report of the Expert Maternity Group. London: HMSO, 1993.

3- Department of Health. Changing childbirth. Part 2: Survey of good communication practice in maternity services. London: HMSO, 1993.

4- Ong LML, de Haes JCJM, Hoos AM, Lammes FB. **Doctor-patient communication: a review of the literature**. Soc Sci Med1995; **40**: 903-918.

5- Stewart MA. **Effective physician-patient communication and health outcomes: a review**. CanMedAssJ 1995; **152**: 1423-1433.

6- Garcia J, Redshaw M, Fitzsimons B, Keene J. First Class Delivery. A National Survey of Women's Views of Maternity Care. London: Audit Commission, 1998.

7- Kabakian-Khasholian T, Campbell O, Shediac-Rizkallah M, Ghorayeb F. (2000) **Women's experiences with maternity care: satisfaction or passivity?** Soc SciMed, **51**:103-113.

8- Garcia J, Garforth S. Parents and new-born babies in the labour ward. In: Garcia J, Kilpatrick R, Richards M (eds.) The Politics of Maternity Care. Services for Childbearing Women in Twentieth-Century Britain. Oxford: Oxford University Press, 1990: 163-182.

9- Rowe RE, Garcia J, Macfarlane AJ, et al. **Improving communication between health professional and women in maternity care: a structured review**. Health Expect2002; **5**: 63-83.

10-DeJong J, Bashour H, Kaddour A. Women's Health: progress and unaddressed issues. In: Jabbour S, Giacaman R, Khawaja M, Nuwayhid I (eds) Public Health in the Arab World. Cambridge University press 2012:

11- Bashour H, Abdulsalam A, Al-Faisal W, Cheikha S. **The patterns and determinants of maternity care in Damascus, Syria**. East Mediterr Health J. 2008; **14**: 398-405.

For peer review only - http://bmjopen.bmj.com/site/about/guidelines.xhtml

12- Hafez R, Bashour H, Abdulsalam A. Childbirth and its discontent: Experiences of Syrian Women. A paper presented at the Annual meeting of Reproductive Health Working Group (RHWG), Lebanon, July 11-13,2004.

~~1516~~ 13- Meakin R and Weinman J. **The Medical Interview Satisfaction Scale (MISS-21) adapted for British general practice**. Fam Pract 2002; **19**:257-263.

~~17 18~~ 14- Sholkamy H, Khalil K, Cherine M, Elnoury A, Breebaart M, Hassanein N. An observation checklist for facility-based normal labour and delivery practices: The Galaa Study. Monographs in Reproductive Health No. 5. Cairo; The Population Council, 2003.

15- Hussey A. and Hughes J.P. **Design and Analysis of stepped wedge cluster randomized trials**. Contemp Clin Trials2007; **28**:182-191.

~~14~~ 16- Brown CA and Lilford RJ. **The stepped wedge trial design: a systematic review**. BMC Med Res Methodol2006, **6**:54.

~~15-~~ 17 Syrian Ministry of Health. www.moh.gov.sy

18- Holloway S, Porteous M, Cetnarskyj R, Anderson E, Rush R, Fry A, Gorman D, Steel M, Campbell H. 2004. **Patient satisfaction with two different models of cancer genetic services in south-east Scotland**. *Br J Cancer***90**: 582–589.

19-Moher D, Hopewell S, Schulz KF, Montori V, Gøtzsche PC, Devereaux PJ, Elbourne D, Egger M, Altman DG, for the CONSORT Group. CONSORT 2010 Explanation and Elaboration: updated guidelines for reporting parallel group randomised trial. BMJ 2010;340:c869.

20- Maguire P and Pitceathly C**. Key communication skills and how to acquire them**. BMJ 2002; **325**:697-700.

21- Brown JB, Boles M, Mullooly JP, Levinson W.**Effect of clinician communication skills training on patient satisfaction. A randomized, controlled trial**. Ann Intern Med 1999; **131**:822–9.

22- Abdulasalam A. **Communication Skills! What does it mean to doctors and women: A qualitative study in Damascus maternity teaching hospitals**. Damascus University Journal for Health Sciences 2009;**25**:189-203. (In Arabic)

For peer review only

23- Berridge E, Mackintosh N and Freeth D. **Supporting patient safety: examining communication within delivery suite teams through contrasting approaches to research observation**. Midwifery 2010; **26**: 512-519.

24- Bingham D. **Helping hospitals change. Part 2: Childbirth education techniques to empower women**. JPerinatal Edu 2005; **14**: 51-55.

25- Choices and Challenges in Changing Childbirth Research Network. **Routines in facility-based maternity care: evidence from the Arab World**. BJOG: an International Journal of Obstetrics and Gynaecology 2005; **112**, 1270-1276.

26- Audit Commission. What seems to be the matter: Communication between hospitals and patients. HMSO, London. 1993.

For peer review only

For peer review only - http://bmjopen.bmj.com/site/about/guidelines.xhtml

Figures:

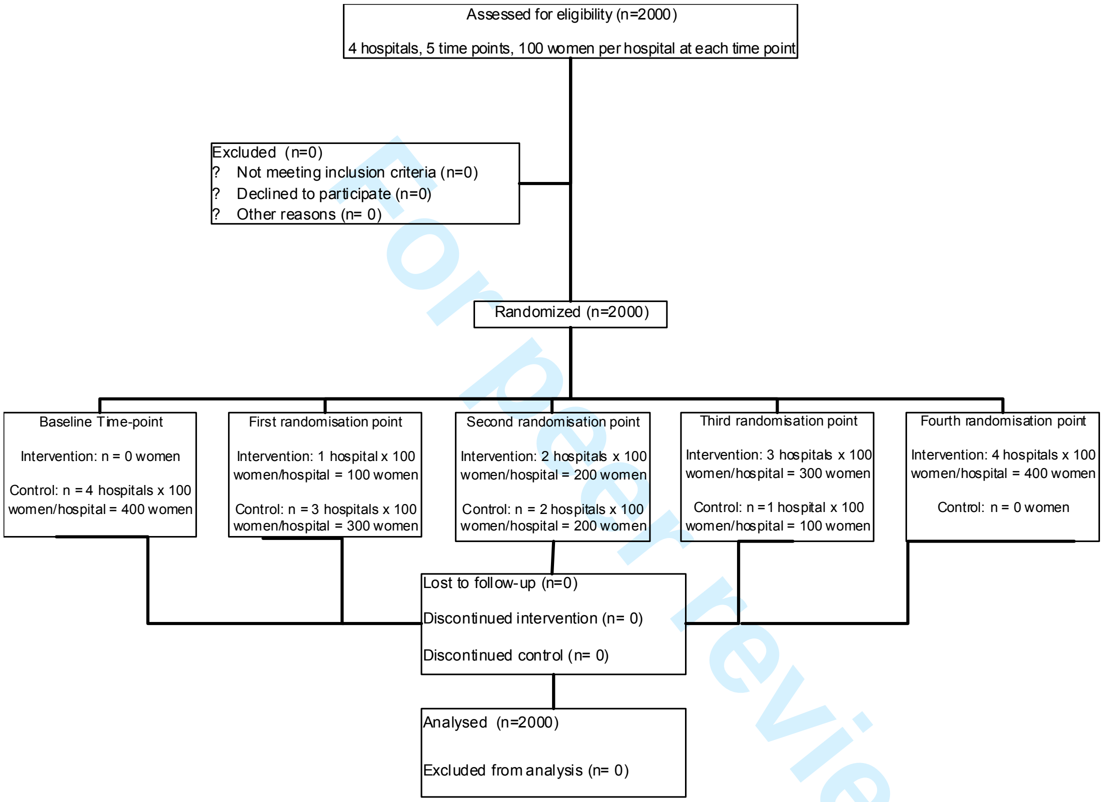

Figure 1: Flow of Participants

For peer review only

For peer review only - http://bmjopen.bmj.com/site/about/guidelines.xhtml

Tables:

For peer review only

Table 1: Demographic characteristics of the women by intervention arm. Numbers represent counts (percentage) unless otherwise stated.

	Control N = 1000	Intervention N = 1000		Control N = 1000	Intervention N = 1000
Mean age (SD)	25.02 (6.11)	25.10 (5.89)	Woman's Education		
Occupation: Housewife	949 (95)	953 (95)	Illiterate/Read and Write	140 (14)	102 (10)
Home Ownership: Share	488 (49)	470 (48)	Primary	745 (74)	761 (77)
Parity: Nulliparous	266 (27)	263 (27)	High School +	110 (11)	128 (13)
New born gender: Male	507 (52)	480 (49)	Husband's Education		
Mean husband's age (SD)	30.90 (6.91)	30.85 (6.62)	Illiterate/Read and Write	113 (11)	101 (10)
Husband's Occupation			Primary	781 (79)	743 (76)
Labourer (manual worker)	359 (36)	337 (34)	High School +	100 (10)	135 (14)
Clerk	160 (16)	198 (20)			
Self-employed	391 (40)	432 (44)			
Other	80 (8)	21 (2)			

For peer review only

Table 2: Numbers (percentages) of women who agreed/strongly agreed with various questions concerning their labour/delivery experience. The numbers compare all the intervention clusters to the control clusters.*

Item	Control Period N=1000	Intervention Period N=1000	Item	Control Period N=1000	Intervention Period N=1000
Overall satisfaction with doctors' communication skills during labour/delivery	619 (62)	713 (72)	Did the doctor listen to you with concern and without interrupting you?	413 (46)	551 (58)
Did the doctors identify themselves?	132 (13)	118 (12)	Was the doctor engaged in other issues, so you felt being unattended and annoyed?	165 (17)	67 (7)
Did the doctor greet you?	208 (21)	323 (32)	Did you feel let down by the way he/she was dealing with you?	66 (7)	31 (3)
Did the doctor look at you when talking to you?	434 (43)	599 (60)	Did the doctor explain all the steps before doing the clinical exam?	249 (25)	278 (29)
Did the doctor show interest in you as a person?	393 (39)	329 (33)	Did the doctor explain the findings from the clinical exam?	326 (34)	399 (41)
Did the doctor insult you?	55 (6)	29 (3)	Were you annoyed by the doctor explaining all the findings?	40 (5)	73 (10)
Did the doctor use humour to comfort you?	78 (8)	118 (12)	Did the doctor explain all the alternative choices to you?	176 (23)	145 (17)
Did the doctor use any terms to calm you down?	363 (36)	419 (42)	Did the doctor help you in making decisions?	175 (29)	158 (23)
Did the doctor use his/her hands to assist you or to reassure you?	314 (32)	409 (42)	Was the doctor annoyed from your questions and avoided answering them?	122 (14)	54 (6)
Did the doctor shout or scream at you?	125 (13)	71 (7)	Did the doctor use medical terms in explanation, thus they were not understood?	77 (8)	48 (5)

* Due to missing information some of the percentages do not tie up with the total number given.

For peer review only - http://bmjopen.bmj.com/site/about/guidelines.xhtml

Table 3: Summary statistics for the outcome of women satisfaction computed as the average score and average score for Complete MMISS.Regression analysis estimates for the average score and average score for complete MMISS on intervention and time controlling for clustering by hospital using GLMM. Estimates are presented together with 95% confidence interval (CI) limits as subscripts ($_{Lower}Est_{Upper}$).

	Average Score	Average Score Complete MMISS
Control: Median(Q1, Q3)	3.3(2.8,3.7)	3.4(3.0,3.9)
Intervention: Median(Q1, Q3)	3.3(3.0, 4.1)	3.3(3.1, 4.1)
GLMM Estimates	$_{Lower}Est_{Upper}$	$_{Lower}Est_{Upper}$
**Fixed-effects**		
intervention	$_{-0.08}0.03_{0.15}$	$_{-0.29}-0.13_{0.04}$
time: 2	$_{-0.08}0.02_{0.12}$	$_{-0.10}0.05_{0.19}$
time: 3	$_{-0.14}-0.03_{0.08}$	$_{-0.02}0.12_{0.27}$
time: 4	$_{-0.08}0.05_{0.18}$	$_{0.12}0.30_{0.49}$
time: 5	$_{-0.15}0.01_{0.16}$	$_{-0.03}0.17_{0.37}$
constant	$_{3.16}3.32_{3.48}$	$_{3.23}3.44_{3.64}$
**Random-effects**	Variance(SE)	Variance(SE)
**Hospital**	0. 02 (0.016)	0.03 (0.026)
**Residual**	0.51 (0.016)	0.45 (0.02)

For peer review only

Table 4: Numbers (and percentages) of negative responses for items on the observational checklist concerning communication between doctor and woman during labour/delivery, unless otherwise stated. Due to missing information some of the percentages do not tie up with the total number given. The columns compare the numbers all the intervention clusters to the control clusters.

	Control Period N = 565	Intervention Period N=631		Control Period N=565	Intervention Period N=631
Mean (SD) of the average score	0.34 (0.13)	0.39 (0.10)			
Mean (SD) of the average score for complete checklist	0.34 (0.10)	0.39 (0.09)			
Checklist Item			Checklist Item		
Did the doctor identify himself/herself?	539 (100)	621 (99)	Was the woman asked any questions at this stage?	168 (32)	94 (15)
Did the doctor call the woman by her name?	132 (24)	99 (16)	Did the woman ask any questions at this stage?	292 (56)	281 (46)
Did the doctor take the woman's permission to examine her?	326 (61)	305 (49)	Did the doctor respond to her queries?	275 (63)	342 (64)
Did the doctor explain the plan he/she will follow?	497 (92)	577 (92)	Was the doctor responsive to the woman's pain?	285 (59)	333 (57)
Before starting the vaginal examination, did the doctor			Was the woman encouraged at this stage?	186 (40)	239 (42)
take her permission for the exam?	335 (62)	262 (42)	Was the woman told about the proximity of labour?	242 (55)	363 (66)
close the door?	472 (88)	568 (91)	Was the woman told about the stages of labour?	399 (95)	533 (98)
cover the woman during the exam?	315 (59)	116 (18)	Was the woman told about her role during labour?	347 (83)	508 (94)
ask the woman to bend her knees?	210 (39)	79 (13)	During delivery, was the woman asked to push?	26 (6)	23 (4)
After completing the vaginal examination, did the doctor			Did they explain to the woman when and how to push?	237 (57)	230 (42)
tell the woman the results of the exam?	418 (78)	504 (81)	Did the woman ask any questions at this stage?	284 (68)	296 (55)
explain the next steps to be followed?	486 (91)	586 (94)	Did the doctor respond to her queries?	264 (77)	330 (72)
relay the findings to other team members?	241 (45)	102 (16)	Did the doctor inform the mother about her baby's status?	307 (75)	386 (72)
relay the findings to the midwives/nurses?	393 (80)	579 (93)	Was the woman congratulated after the delivery?	217 (53)	214 (40)
Did the doctor give instructions about eating and drinking?	463 (87)	516 (83)	Was the woman instructed about the process after the delivery?	392 (95)	515 (96)
Did the doctor give instructions about movement?	335 (63)	428 (70)	Was the woman instructed about how to care for her newborn?	409 (99)	534 (99)

For peer review only

For peer review only - http://bmjopen.bmj.com/site/about/guidelines.xhtml

Appendix:

Figure 2 gives the distribution of the average score by treatment arm.

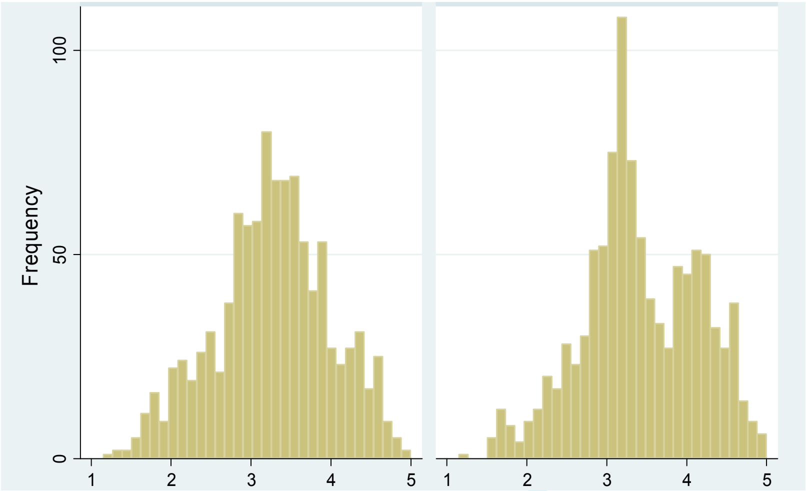

(a) (b)

Figure 2: Distribution of the average score by treatment arm (a) Control and (b) Intervention.

For peer review only - http://bmjopen.bmj.com/site/about/guidelines.xhtml

Description of supporting files

Additional File one: **Diagram of the study design**

Additional File two: **A graphical representation of the overall satisfaction outcome score of the women stratified by hospital and time of intervention. Data presented are for complete MMISS only**

Additional File three: **Results by intervention period.**

Additional File four**: Trial Protocol.**

For peer review only

For peer review only - http://bmjopen.bmj.com/site/about/guidelines.xhtml

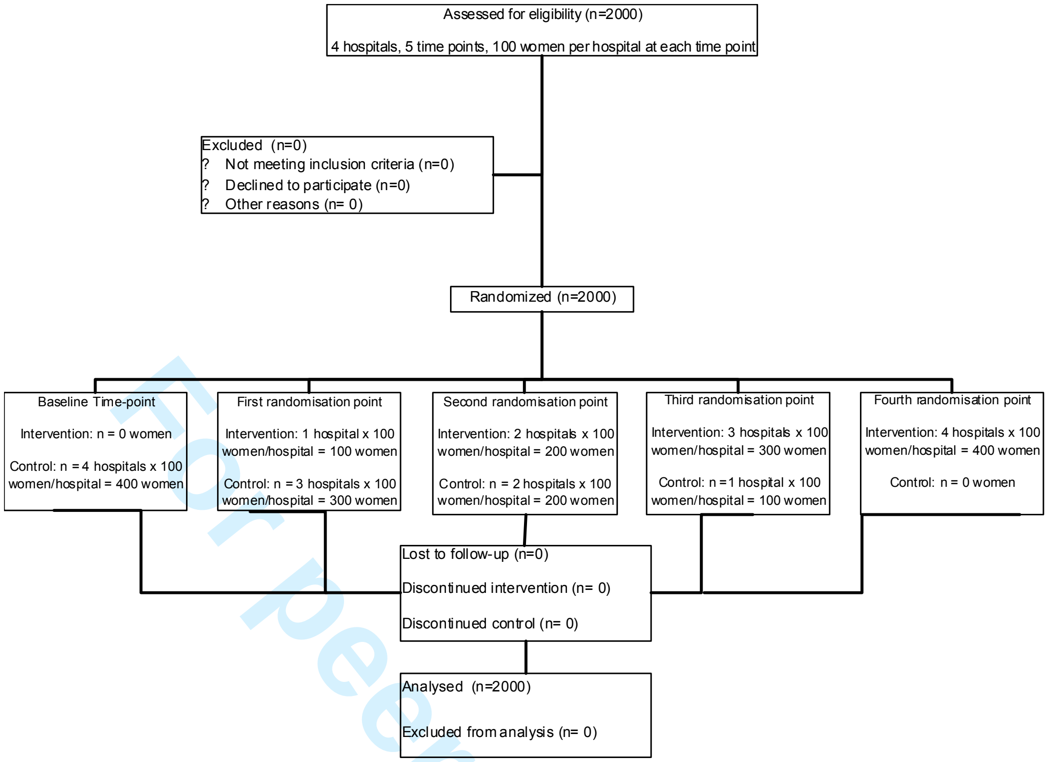

Figure 1: Flow of Participants

For peer review only

Figure 1: A graphical representation of the overall satisfaction question of the women stratified by hospital. Data presented represent proportion in each category of overall satisfaction at the different time periods.

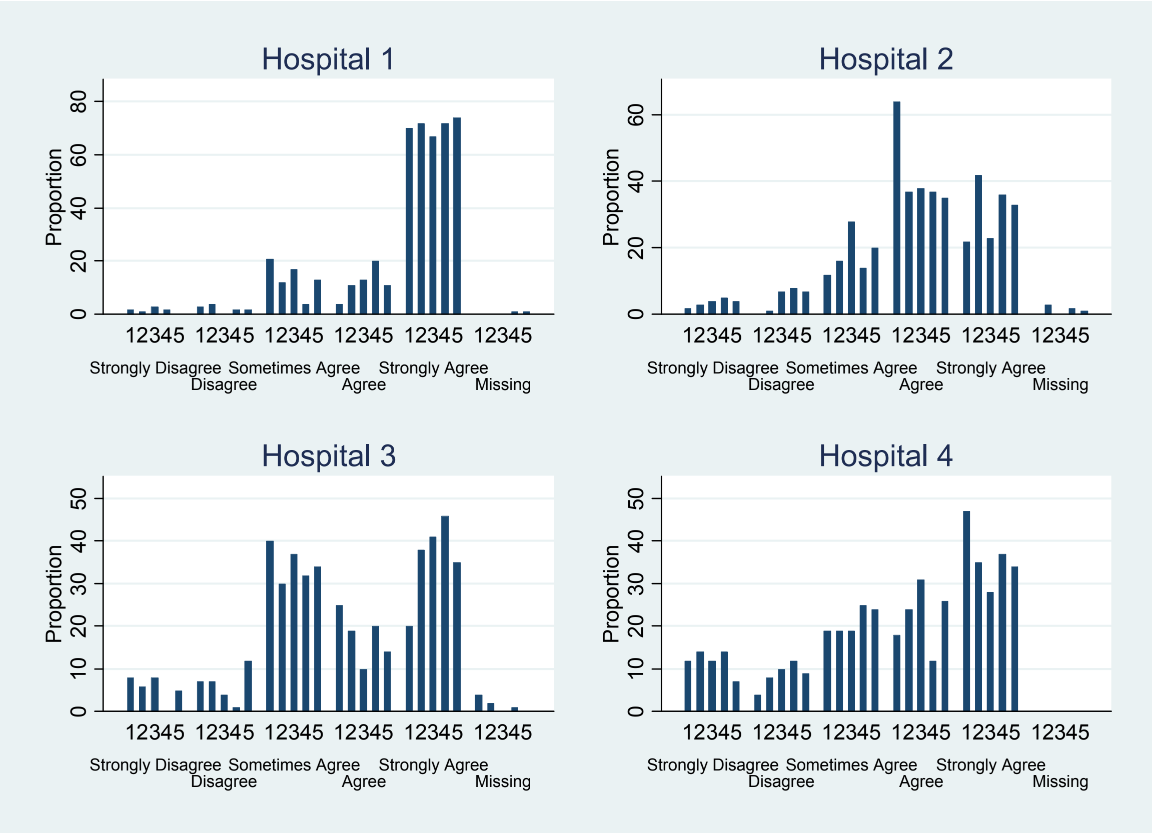

For peer review only - http://bmjopen.bmj.com/site/about/guidelines.xhtml

Additional File One: Schema of the study design*

Hospital	Time				
	1	2	3	4	5
1					
2					
3					
4					

Time 1: Baseline measurement
Time 2: Follow the implementation of intervention at randomly selected hospitals 1
Time 3: Follow the implementation of intervention at randomly selected hospitals 2
Time 4: Follow the implementation of intervention at randomly selected hospitals 3
Time 5: Follow the implementation of intervention at randomly selected hospitals 4

* Shaded area refer to post-intervention

For peer review only

Additional File Two:

A graphical representation of the overall satisfaction outcome score of the women stratified by hospital and time of intervention. Data presented are for complete MMISS only. The rows represent the four hospitals and the columns the five randomisation points. For presentation purposes, the hospitals were coded such that Hospital 1 represented the hospital that had the intervention delivered first which was "time 2" and so forth; "time 1" was the baseline. The title of each graph indicates the hospital and time point presented, for example, a title of 2,3 indicates that this represents the score distribution in Hospital 2 at time 3 (the first intervention period for this hospital).

For peer review only

For peer review only - http://bmjopen.bmj.com/site/about/guidelines.xhtml

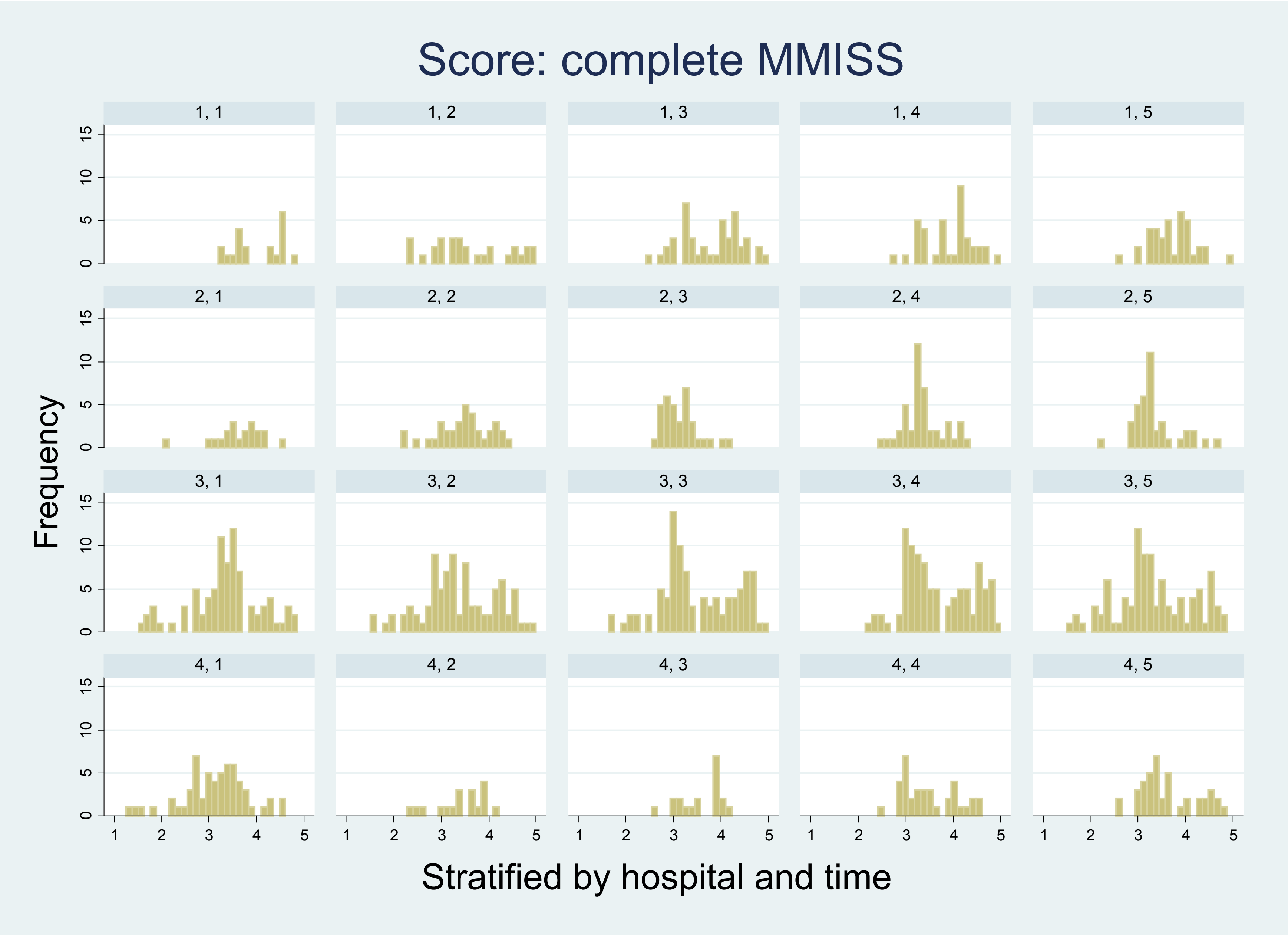
Score: complete MMISS
1, 1
1, 2
1, 3
1, 4
1, 5
2, 1
2, 2
2, 3
2, 4
2, 5
3, 1
3, 2
3, 3
3, 4
3, 5
4, 1
4, 2
4, 3
4, 4
4, 5
Frequency
0
5
10
15
1
2
3
4
5
Stratified by hospital and time

For peer review only - http://bmjopen.bmj.com/site/about/guidelines.xhtml

Table 1: Demographic Characteristics of the women across the time periods of the study[!]

	1	2	3	4	5
N	400	400	400	400	400
Mean age (SD)	24.79 (5.71)	25.12 (6.11)	25.07 (6.4)	24.97 (5.74)	25.37(6.04)
Median age (Q1, Q3)*	24 (20, 28)	24 (20, 29)	24 (20, 29.5)	24 (20, 29)	24 (20, 29)
Occupation: Housewife	377 (94)	377 (94)	387 (97)	378 (94)	383 (96)
Education					
Illiterate/Read and Write	53 (13)	51 (13)	43 (11)	53 (13)	42 (11)
Primary	291 (74)	306 (77)	304 (76)	305 (77)	300 (76)
High School +	51 (13)	42 (11)	53 (13)	38 (10)	54 (14)
Mean husband's age (SD)	30.98 (6.89)	31.19 (7.18)	31 (6.82)	30.16 (6.35)	31.07 (6.53)
Median husband's age (Q1, Q3)	30 (26, 35)	30 (26, 35)	30 (26, 35)	29 (26, 34)	30 (26, 35)
Husband's Occupation					
Labourer (manual worker)	152 (39)	131 (33)	134 (34)	143 (36)	136 (34)
Clerk	72 (18)	71 (18)	64 (16)	80 (20)	71 (18)
(Self-employed)	144 (37)	163 (41)	175 (44)	166 (42)	175 (44)
Other	24 (6)	33 (8)	24 (6)	7 (2)	13 (3)
Husband's Education					
Illiterate/Read and Write	55 (14)	48 (12)	41 (10)	37 (9)	33 (8)
Primary	299 (76)	295 (74)	310 (78)	310 (79)	310 (79)

For peer review only - http://bmjopen.bmj.com/site/about/guidelines.xhtml

High School +	41 (10)	53 (13)	45 (11)	46 (12)	50 (13)
Home Ownership					
Owns home	192 (49)	197 (49)	217 (55)	214 (55)	197 (50)
Share	201 (51)	202 (51)	179 (45)	178 (45)	198 (50)
Parity: Nulliparous	100 (25)	120 (30)	124 (31)	97 (24)	88 (22)
New born gender: Male	215 (56)	193 (49)	179 (45)	198 (52)	202 (52)

* Q1 is the first quartile and Q3 is the third quartile.

! Numbers represent counts (percentage) unless otherwise stated.

For peer review only

For peer review only - http://bmjopen.bmj.com/site/about/guidelines.xhtml

Table 2: Numbers (and percentages) of women who agreed/strongly agreed with various questions concerning their labour/delivery experience. The first two columns give the numbers comparing all the intervention clusters to the control clusters; the other columns give the corresponding numbers stratified by hospital.*

	Control Period	Intervention Period	Control Period				Intervention Period			
	All	All	1	2	3	4	1	2	3	4
N	1000	1000	100	200	300	400	400	300	200	100
Overall satisfaction with doctors' communication skills during labour/delivery	619 (62)	713 (72)	74 (74)	163 (83)	150 (51)	232 (58)	338 (85)	200 (67)	115 (58)	60 (60)
Did the doctors identify themselves?	132 (13)	118 (12)	1 (1)	11 (6)	94 (31)	26 (7)	35 (9)	7 (2)	62 (31)	14 (14)
Did the doctor greet you?	208 (21)	323 (32)	36 (36)	45 (23)	86 (29)	41 (10)	183 (46)	55 (19)	63 (32)	22 (22)
Did the doctor look at you when talking to you?	434 (43)	599 (60)	61 (61)	158 (79)	94 (31)	121 (30)	305 (76)	181 (61)	78 (39)	35 (35)
Did the doctor show interest in you as a person?	393 (39)	329 (33)	47 (47)	42 (21)	139 (46)	165 (41)	188 (47)	21 (7)	83 (42)	37 (37)
Did the doctor insult you?	55 (6)	29 (3)	2 (2)	3 (2)	15 (5)	35 (9)	5 (1)	19 (6)	1 (1)	4 (4)
Did the doctor use humour to comfort you?	78 (8)	118 (12)	19 (19)	13 (7)	34 (11)	12 (3)	70 (18)	7 (2)	34 (17)	7 (7)
Did the doctor use any terms to calm you down?	363 (36)	419 (42)	44 (44)	102 (51)	109 (36)	108 (27)	200 (50)	116 (39)	73 (37)	30 (30)

For peer review only

Did the doctor use his/her hands to assist you or to reassure you?	314 (32)	409 (42)	52 (54)	115 (58)	79 (26)	68 (17)	200 (52)	137 (46)	57 (29)	15 (16)
Did the doctor shout or scream at you?	125 (13)	71 (7)	6 (6)	15 (8)	28 (9)	76 (19)	18 (5)	31 (10)	10 (5)	12 (12)
Did the doctor listen to you with concern and without interrupting you?	413 (46)	551 (58)	57 (62)	108 (65)	98 (33)	150 (44)	313 (79)	116 (45)	73 (37)	49 (50)
Was the doctor engaged in other issues, so you felt being unattended and annoyed?	165 (17)	67 (7)	3 (3)	20 (10)	75 (25)	67 (17)	12 (3)	19 (6)	27 (14)	9 (9)
Did you feel let down by the way he/she was dealing with you?	66 (7)	31 (3)	2 (2)	10 (5)	10 (3)	44 (11)	10 (3)	19 (6)	1 (1)	1 (1)
Did the doctor explain all the steps before doing the clinical exam?	249 (25)	278 (29)	17 (17)	88 (45)	41 (14)	103 (26)	70 (18)	128 (47)	46 (23)	34 (35)
Did the doctor explain the findings from the clinical exam?	326 (34)	399 (41)	42 (68)	82 (42)	61 (20)	141 (36)	181 (46)	101 (37)	63 (32)	54 (56)
Were you annoyed by the doctor explaining all the findings?	40 (5)	73 (10)	4 (8)	18 (13)	3 (1)	15 (5)	29 (11)	39 (18)	2 (1)	3 (3)
Did the doctor explain all the alternative choices to you?	176 (23)	145 (17)	12 (27)	44 (35)	94 (31)	26 (9)	37 (10)	31 (16)	66 (33)	11 (13)
Did the doctor help you in	175	158	12	25	97	41	52	16	75	15

For peer review only - http://bmjopen.bmj.com/site/about/guidelines.xhtml

For peer review only

making decisions?	(29)	(23)	(44)	(29)	(33)	(21)	(19)	(9)	(38)	(35)
Was the doctor annoyed from your questions and avoided answering them?	122 (14)	54 (6)	9 (11)	14 (9)	35 (12)	64 (19)	20 (6)	21 (8)	6 (3)	7 (7)
Did the doctor use medical terms in explanation, thus they were not understood?	77 (8)	48 (5)	0 (0)	6 (3)	55 (19)	16 (5)	1 (0)	5 (2)	37 (19)	5 (6)
Did the doctor give you conflicting medical information?	31 (3)	12 (1)	1 (1)	3 (2)	6 (2)	21 (6)	0 (0)	9 (3)	2 (1)	1 (1)

* Due to missing information some of the percentages do not tie up with the total number given.

Table 3: Summary statistics for the outcome of women satisfaction computed as the average score and Average Score for Complete MMISS . The first two columns give the numbers comparing all the intervention clusters to the control clusters; the other columns give the corresponding numbers stratified by hospital

For peer review only

1

	Control Period	Intervention Period	Control Period				Intervention Period			
	All	All	1	2	3	4	1	2	3	4
Average Score										
N	1000	1000	100	200	300	400	400	300	200	100
Mean (SD)	3.23 (0.72)	3.42 (0.73)	3.57 (0.72)	3.43 (0.59)	3.33 (0.78)	3.06 (0.67)	3.54 (0.73)	3.25 (0.66)	3.48 (0.77)	3.33 (0.71)
Median (Q1, Q3)	3.3 (2.8,3.7)	3.3 (3.0, 4.1)	3.6 (3.1, 4.3)	3.5 (3.1, 3.8)	3.3 (2.9, 3.9)	3.2 (2.6, 3.5)	3.5 (3.0, 4.2)	3.2 (2.9, 3.7)	3.4 (3.0, 4.2)	3.3 (3.0, 3.7)
Average Score for Complete MMISS										
N	472	498	20	55	265	132	148	118	191	41
Mean (SD)	3.43 (0.71)	3.54 (0.66)	4.04 (0.54)	3.52 (0.56)	3.42 (0.77)	3.33 (0.62)	3.77 (0.62)	3.29 (0.43)	3.50 (0.77)	3.62 (0.60)
Median (Q1, Q3)	3.4 (3.0,3.9)	3.3 (3.1, 4.1)	4.1 (3.6,4.6)	3.6 (3.2, 3.9)	3.3 (2.9, 4.1)	3.4 (3.0, 3.8)	3.8 (3.3, 4.2)	3.2 (3.0, 3.4)	3.35 (3.1, 4.2)	3.4 (3.2, 4)

For peer review only

Table 4: Regression analysis estimates for the average score and average score for complete MMISS on intervention and time controlling for clustering by hospital using three estimation procedures LMM, GLAMM and GEE. Estimates are presented together with 95% confidence interval (CI) limits as subscripts ($_{Lower}$ Est$_{Upper}$).

	Average Score			Average Score Complete MMISS		
	LMM	GLAMM	GEE	LMM	GLAMM	GEE
	$_{Lower}$Est$_{Upper}$	$_{Lower}$Est$_{Upper}$	$_{Lower}$Est$_{Upper}$	$_{Lower}$Est$_{Upper}$	$_{Lower}$Est$_{Upper}$	$_{Lower}$Est$_{Upper}$
**Fixed-effects**						
intervention	$_{-0.09}0.03_{0.14}$	$_{-0.08}0.03_{0.15}$	$_{-0.08}0.03_{0.14}$	$_{-0.29}-0.13_{0.03}$	$_{-0.29}-0.13_{0.04}$	$_{-0.26}-0.10_{0.06}$
time: 2	$_{-0.08}0.02_{0.13}$	$_{-0.08}0.02_{0.12}$	$_{-0.08}0.02_{0.12}$	$_{-0.10}0.04_{0.19}$	$_{-0.10}0.05_{0.19}$	$_{-0.10}0.05_{0.19}$
time: 3	$_{-0.14}-0.03_{0.09}$	$_{-0.14}-0.03_{0.08}$	$_{-0.14}-0.03_{0.08}$	$_{-0.02}0.12_{0.27}$	$_{-0.02}0.12_{0.27}$	$_{-0.03}0.12_{0.27}$
time: 4	$_{-0.08}0.05_{0.18}$	$_{-0.08}0.05_{0.18}$	$_{-0.08}0.05_{0.18}$	$_{0.13}0.31_{0.49}$	$_{0.12}0.30_{0.49}$	$_{0.10}0.28_{0.47}$
time: 5	$_{-0.14}0.01_{0.16}$	$_{-0.15}0.01_{0.16}$	$_{-0.14}0.01_{0.15}$	$_{-0.02}0.18_{0.38}$	$_{-0.03}0.17_{0.37}$	$_{-0.05}0.15_{0.35}$
constant	$_{3.14}3.32_{3.50}$	$_{3.16}3.32_{3.48}$	$_{3.16}3.32_{3.48}$	$_{3.20}3.84_{3.67}$	$_{3.23}3.44_{3.64}$	$_{3.28}3.43_{3.59}$
**Random-effects**						
	SD	Var		SD	Var	
**hospital**	$_{0.07}0.17_{0.40}$	0. 02 (0.016)		$_{0.09}0.22_{0.51}$	0.03 (0.026)	
**Residual**	$_{0.68}0.71_{0.73}$	0.51 (0.016)		$_{0.64}0.67_{0.70}$	0.45 (0.02)	

For peer review only - http://bmjopen.bmj.com/site/about/guidelines.xhtml

Table 5: Numbers (and percentages) of "No" for items on the observational checklist concerning communication between doctor and woman during labour/delivery, unless otherwise stated. Due to missing information some of the percentages do not tie up with the total number given. The first two columns give the numbers comparing all the intervention clusters to the control clusters; the other columns give the corresponding numbers stratified by hospital.

	Control Period	Intervention Period	Control Period				Intervention Period			
Hospital	All	All	1	2	3	4	1	2	3	4
N	565	631	98	194	106	167	326	218	38	49
1. Did the doctor identify himself/herself?	539 (100)	621 (99)	90 (100)	177 (99)	105 (100)	167 (100)	320 (99)	215 (100)	38 (100)	48 (98)
2. Did the doctor call the woman by her name?	132 (24)	99 (16)	22 (24)	48 (27)	22 (21)	40 (24)	52 (16)	30 (14)	6 (16)	11 (22)
3. Did the doctor take the woman's permission to examine her?	326 (61)	305 (49)	53 (59)	113 (64)	61 (58)	99 (60)	183 (57)	77 (36)	18 (47)	27 (55)
4. Did the doctor explain the plan he/she will follow?	497 (92)	577 (92)	81 (90)	168 (95)	98 (93)	150 (90)	296 (92)	203 (94)	35 (92)	43 (88)
Before starting the vaginal examination, did the doctor										
5. take her permission for the exam?	335 (62)	262 (42)	58 (65)	115 (65)	58 (55)	104 (63)	155 (48)	68 (32)	14 (37)	25 (51)
6. close the door?	472 (88)	568 (91)	85 (96)	136 (77)	87 (83)	164 (99)	313 (97)	180 (84)	26 (68)	49 (100)

For peer review only

7. cover the woman during the exam?	315 (59)	116 (18)	20 (23)	97 (55)	79 (75)	119 (72)	32 (10)	21 (10)	21 (55)	42 (91)
8. ask the woman to bend her knees?	210 (39)	79 (13)	34 (38)	76 (43)	31 (30)	69 (42)	48 (15)	34 (16)	6 (16)	7 (14)
After completing the vaginal examination, did the doctor										
9. tell the woman the results of the exam?	418 (78)	504 (81)	73 (82)	157 (89)	74 (70)	114 (69)	270 (84)	174 (81)	31 (82)	29 (59)
10. explain the next steps to be followed?	486 (91)	586 (94)	73 (83)	168 (95)	98 (94)	147 (89)	302 (94)	204 (95)	34 (89)	46 (94)
11. relay the findings to other team members?	241 (45)	102 (16)	17 (19)	87 (49)	45 (43)	92 (59)	41 (13)	30 (14)	12 (36)	19 (39)
12. relay the findings to the midwives/nurses?	393 (80)	579 (93)	60 (83)	154 (87)	70 (71)	109 (75)	312 (97)	211 (98)	32 (84)	24 (49)
13. Did the doctor give instructions about eating and drinking?	463 (87)	516 (83)	77 (89)	148 (84)	90 (86)	148 (90)	261 (81)	183 (85)	33 (87)	39 (80)
14. Did the doctor give instructions about movement?	335 (63)	428 (70)	65 (77)	112 (62)	59 (57)	99 (60)	222 (71)	155 (72)	26 (68)	25 (51)
15. Was the woman asked any questions at this stage?	168 (32)	94 (15)	11 (14)	82 (46)	26 (25)	49 (30)	43 (14)	38 (18)	6 (16)	7 (14)
16. Did the woman ask any questions at this stage?	292 (56)	281 (46)	34 (44)	123 (68)	52 (52)	83 (51)	152 (49)	93 (44)	13 (35)	23 (47)
17. Did the doctor respond to her queries?	275 (63)	342 (64)	32 (44)	96 (70)	59 (65)	88 (65)	168 (65)	124 (64)	21 (64)	29 (63)
18. Was the doctor responsive to the woman's pain?	285 (59)	333 (57)	35 (47)	95 (58)	54 (59)	101 (65)	162 (53)	119 (58)	22 (73)	30 (63)

For peer review only - http://bmjopen.bmj.com/site/about/guidelines.xhtml

19. Was the woman encouraged at this stage?	186 (40)	239 (42)	17 (25)	59 (38)	33 (36)	77 (52)	115 (40)	85 (42)	16 (53)	23 (48)
20. Was the woman told about the proximity of labour?	242 (55)	363 (66)	25 (39)	79 (54)	49 (55)	89 (63)	180 (65)	136 (68)	20 (71)	27 (60)
21. Was the woman told about the stages of labour?	399 (95)	533 (98)	49 (83)	133 (96)	85 (97)	132 (96)	269 (98)	197 (99)	28 (100)	39 (98)
22. Was the woman told about her role during labour?	347 (83)	508 (94)	29 (51)	114 (83)	80 (90)	124 (91)	256 (93)	190 (95)	25 (89)	37 (93)
23. During delivery, was the woman asked to push?	26 (6)	23 (4)	6 (11)	6 (4)	3 (3)	11 (8)	13 (5)	7 (4)	3 (11)	0 (0)
24. Did they explain to the woman when and how to push?	237 (57)	230 (42)	30 (54)	76 (55)	48 (55)	83 (61)	114 (41)	85 (43)	15 (54)	16 (40)
25. Did the woman ask any questions at this stage?	284 (68)	296 (55)	35 (63)	100 (73)	59 (67)	90 (66)	166 (61)	95 (48)	15 (54)	20 (50)
26. Did the doctor respond to her queries?	264 (77)	330 (72)	36 (68)	77 (77)	66 (81)	85 (78)	160 (71)	127 (73)	17 (81)	26 (68)
27. Did the doctor inform the mother about her baby's status?	307 (75)	386 (72)	26 (49)	92 (68)	72 (83)	117 (86)	171 (63)	161 (82)	25 (89)	29 (74)
28. Was the woman congratulated after the delivery?	217 (53)	214 (40)	26 (47)	63 (46)	49 (58)	79 (58)	102 (37)	81 (41)	10 (36)	21 (53)
29. Was the woman instructed about the process after the delivery?	392 (95)	515 (96)	51 (93)	127 (93)	82 (95)	132 (97)	267 (97)	182 (92)	27 (96)	39 (98)
30. Was the woman instructed about how to care for her newborn?	409 (99)	534 (99)	53 (98)	134 (98)	86 (100)	136 (100)	273 (100)	194 (98)	28 (100)	39 (100)
31. Was the woman reassured the she is going to be fine?	373 (90)	511 (95)	34 (62)	125 (91)	82 (95)	132 (97)	261 (96)	184 (93)	28 (100)	38 (95)
Mean (SD) of the average score	0.34	0.39	0.41	0.31	0.35	0.31	0.39	0.40	0.38	0.38

For peer review only

	(0.13)	(0.10)	(0.13)	(0.13)	(0.10)	(0.13)	(0.10)	(0.10)	(0.13)	(0.10)
Mean (SD) of the average score for complete checklist	0.34 (0.10)	0.39 (0.09)	0.40 (0.13)	0.36 (0.10)	0.35 (0.10)	0.32(0 .10)	0.39 (0.09)	0.39(0 .08)	0.38 (0.08)	0.38 (0.11)

For peer review only

For peer review only - http://bmjopen.bmj.com/site/about/guidelines.xhtml

Research Proposal
Presented to CCCC/AUB from the Syrian Team
June 2007

Team members
Hyam Bashour
Asmaa Abdulsalam
Mayada Kharouf
Salah Cheikha
Mohammed Tabbaa
In collaboration with ***Mona Kanaan*** from FHS, AUB

For peer review only

Title:

Effect of doctors' training in interpersonal and communication skills on women's satisfaction at labor and delivery: a stepped wedge cluster randomized trial in Damascus

Introduction

The growing body of literature linking communication skills to a host of valued patient outcomes, including satisfaction, adherence, and positive health indicators, is drawing increasing attention to the centrality of the interpersonal skills of healthcare providers to the delivery of quality medical care. As a result, communication in health-care, particularly between health-care professionals and patients, has attracted an increasing amount of attention at official and professional levels in recent years. Many academic and statutory bodies have all been involved in initiatives to promote good communication (Teutsch, 2003). In the United Kingdom, guidance from the General Medical Council on undergraduate medical education places communication skills training at the heart of the curriculums although the extent of compliance with these recommendations is variable. More recently, in the National Health System (NHS) Plan, the UK Department of Health announced plans for new training in communication skills across all professions in the NHS. These initiatives are in part a response to increasing evidence from researchers that the quality of the interaction

between patients and their care providers may have a significant effect on a variety of aspects of patient well being. These include satisfaction, knowledge and understanding, compliance with advice or treatment, quality of life, and psychological and other health outcomes (Ong 1995, Stewart, 1995). In the United States of America, bodies such as the National Board of Medical Examiners and the Educational Commission for Foreign Medical graduates have proposed an examination in communication skills of the medical students (Teutsch, 2003).

In primary care literature, there are many examples of positive benefits for patients when physicians engage in a patient-centered approach to treatment and care (Jenkins and Fallowfield, 2002). Beck et al (2002) argue that a communicative provider-patient relationship is especially important in the management of chronic disease. They have classified behaviour into three key functions of the interaction: data gathering to understand the patient (gathering information), development of rapport and responding to the patient's emotions (developing a therapeutic relationship), and patient education and behavioural management (decision making and management). Figure 1 provides a schematic of the model developed by them. Beck et al in their work concluded that existing research is limited because of lack of consensus of what to measure.

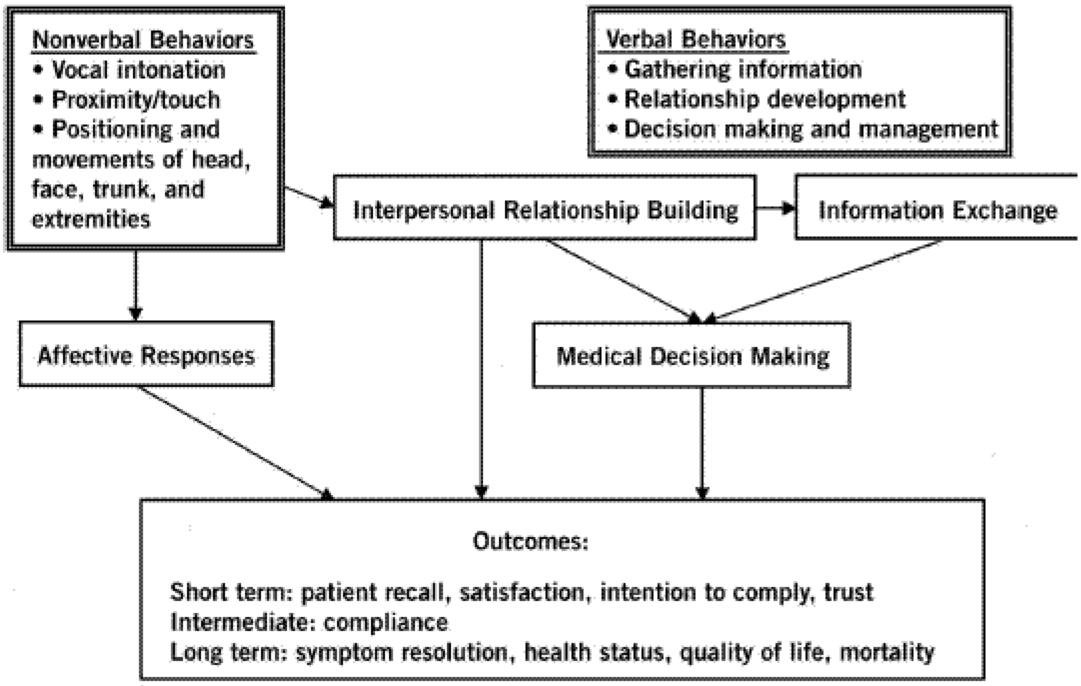

Figure 1. Domains of communication in the provider-patient relationship.

Maternity care is an area of health-care in which the importance of good communication has received particular attention. *Changing Childbirth*, the report of the Expert Maternity Group, commissioned in 1992 by the Department of Health to review maternity care in England and Wales, highlighted the importance of good communication and recognized that in many cases communication is not as good as it should be (Dept. of Health 1993). A separate volume outlined the principles of good communication and detailed examples of good communication practice (Dept. of Health , 1993).

Studies of women's views of maternity care suggest that good communication is central in determining whether women are satisfied with the care that they receive or not (Garcia, 1998, Kabakian-Khasholian et al, 2000). In Changing Childbirth, the provision of appropriate and accessible information was recognized as an important aspect of communication. Listening to women's preferences and concerns is also central (Dept of Health, 1993). Changing Childbirth was explicit in recognizing that there is no template for effective communication and good communication will always be difficult to define, but studies of women's views of maternity care support the idea that good communication is essentially a two-way process. Observational studies of communication between midwives and women during labour have highlighted the misunderstandings that can occur when communication is poor at this time and identified areas where communication could be improved (Garcia, 1990).

The report of the second national US survey of women childbearing's experiences showed the nearly all women reported receiving supportive care (comfort, emotional support, information) for some period while in labour by at least one person, mostly husbands (Childbirth Connection, 2006). Bingham (2005) stressed the importance of carrying out patient satisfaction surveys to help hospitals change its childbirth policy. The Cochrane authors, however, found that features of modern hospital environments, as currently organized, make it challenging for hospital staff to provide continuous labor support, in spite of the importance of continuous care (Hodnett *et al*, 2003).

Despite the acknowledged importance of communication in maternity care, and the official recognition that communication is not always as good as it should be, there have been few evaluations of strategies to improve communication between women and their care providers. Previous reviews of doctor-patient communication have not included any studies carried out in maternity care (Ong 1995, Stewart 1995). The review carried out by Rowe (2002) aimed

For peer review only - http://bmjopen.bmj.com/site/about/guidelines.xhtml

to fill in that gap and thus identified and reviewed trials of the effectiveness of interventions aimed at improving communication between health professionals and women in maternity care. The review identified no trials in the area of antenatal care, but also identified a major gap in knowledge relating to communication in a number of key areas of delivery and postnatal care. They recommended that trials of interventions to improve communication between carers and women in labor and in the postnatal period would be particularly useful. All the studies under review were carried out in developed countries, mainly in the United Kingdom.

The theoretical model for doctor-patient communication suggested by Ong and colleagues (1995) is a useful model to conceptualize an interventional research that aims to improve communication.

**Background variables**	**Actual content of communication (Process)**	**Patient outcomes**
Disease characteristics<br><br>Culture<br><br>Type of patient personality<br><br>Type of doctor personality<br><br>Doctor patient relationship	Communicative behavior including privacy<br><br>Instrumental (cure-oriented) vs. affective behaviors (care-oriented).	Short term: satisfaction, compliance, recall, denial, understanding, hope, etc<br><br>Intermediate and Long term: health status, psychological reaction

Rationale

In Syria, previous work by the researchers both in hospitals and at the community level, very clearly showed that continuous support of women during labor and delivery does not exist. Companionship is not allowed at hospitals, however in a population based study, women reported the existence of a companion at delivery in 37% of the cases (Bashour et al, in press). In that study, it was also evident that women do receive a very fragmented maternal care (only 37% of the studied women attended at delivery by the same person cared the antenatal period), which means that women are usually confronted with birth attendants

For peer review only

whom they meet for the first time at the time of delivery. Furthermore, the length of stay at the hospital for delivery was very short (one fourth of the women stayed for 1-2 hours only), and most probably this is due to the lack of comfort at the hospital.

Also Syrian women expressed very clearly their dissatisfaction with being left alone and they also expressed their negative experiences with being attended by care providers who are not equipped with good communication skills (Hafez *et al*, forthcoming publication). It was very clear that Syrian women are left without any social and emotional support during a critical period in their lives. In most occasions, they are not allowed to be accompanied by their relatives when in labor and delivery, but worse than this is that they seem to be cared of by doctors and midwives with poor communication skills and little interpersonal competences. There were many instances in which doctors and midwives themselves were sources of dissatisfaction.

Apart from women's views, we are fully aware that medical students in Syria, whether in the undergraduate or postgraduate levels, do not receive any formal teaching in communication skills (FOM, 2005). Furthermore, the importance of good communication is not a part of the clinical and ward training for postgraduate students in Obstetrics and Gynecology (Department of Ob/GYN, 2005).

A small scale qualitative study that was carried out in the Maternity Hospitals highlighted the importance of tackling the issue of communication skills in those hospitals (Abdusalam et al, 2006). It was evident that things might be better in private hospitals as compared to public hospitals, as doctors themselves stated. Doctors were themselves perpetrators of violence against women (verbal and physical). Although the word "violence" did not came in that study (described later in details in the annex), but this is indeed a very important aspect of violence against women in general terms.

Sethuraman 2001, described many clinical settings where patients are often considered as "non-persons". The setting we will work in study fits with this description. We will implement the intervention to improve the doctor-patient relationship and we will measure both the patient outcomes that is the woman's satisfaction, as well as the process (communicative behavior of the care provider) according to Ong's Model.

Methods

Participants:

Care providers including mainly residents working in the public maternity hospitals in Damascus and its surroundings. The hospitals are the Maternity Teaching hospital in Damascus University (15000 deliveries each year), Al-Zahrawi hospital, which is the largest Ministry of Health's hospital in Damascus (13000 deliveries each year), Douma hospital, which is a MOH district hospital in rural Damascus (7000 deliveries per year), Harasta hospital, a MOH district hospital in rural Damascus (5000 deliveries per year). Eligible participants in the intervention are residents registered for the year of field work in those hospitals. The total number of residents in the four hospitals is about 125 residents. They are distributed as follows: 75 in the Maternity Teaching Hospital; 24 in Al-Zahrawi Hospital; 12 in Douma Hospital; and 12 in Harasta Hospital.

Midwives were excluded from this study since they have no role in the delivery process, according to the directors at hospitals. As we work in teaching hospitals, we learnt that midwives are not allowed to participate in the delivery, to give the chance for residents to do all the work themselves; this is the training policy in those hospitals.

Women as potential beneficiaries from our intervention would include all women using the hospitals under study. We will target in our measurement all women with living babies who agreed to participate in the study, whether the delivery was normal vaginal or by C-section. Women using those hospitals are largely from middle and low socio-economic class.

Intervention:

The main and the only intervention in this study is the exposure of study participants (residents in the hospitals) to a training package in interpersonal and communication skills using a specially designed training package which will make sure that the key tasks in communication are covered using effective teaching methods (Maguire 2002). The training package will be developed by the study team in cooperation with experts in the field (one International expert and an Arab-speaking one). The training will be designed and presented as a package which is rather relevant to doctors in their future career. It will be advocated as

For peer review only

an essential training for doctors that teaches them the "how to" in the field of communication skill.

Objectives:

The main objective of this study is to test the effect of training residents in interpersonal and communication skills on women's satisfaction with patient-doctor relationship in labor and delivery rooms.

The secondary objective of this study is to measure the change in communicative behavior of the residents after the training in communication skills.

Outcomes:

Two outcomes will be measured in this research. Those are the women's satisfaction (short term outcome according to Ong model) as well as the communicative behaviour of doctors (a process indicator in Ong Model).

The main outcome of this study is the increased women's satisfaction with interpersonal relationships of doctors working in labor and delivery rooms. The secondary outcome is the positive change in communicative behavior of the care providers (residents) serving in labor and delivery rooms. This is also could be considered as a process evaluation, but we are hypothesizing that the training might not change the communicative behavior if constrained by environmental factors such as work load and time pressure.

The first outcome will be measured using a Likert scale questionnaire on women's satisfaction investigating the communication skills of residents attending her (See the attached in annex 1). The second outcome will be measured using an observation checklist (See the attached in annex 2). A specially designed questionnaire will also be used to collect data on potential factors that could determine the communicative behavior of the resident. (See the attached in annex 3).

Tools that were consulted to develop our tools in this study include: the patient satisfaction questionnaire, the MISS-21 (Meakin and Weinman, 2002). The observation checklist developed by Al-Galaa study in Egypt that was tested and validated (Sholkamy *et al*, 2003).

For peer review only - http://bmjopen.bmj.com/site/about/guidelines.xhtml

A validation exercise of the measurement tools is planned.. This will be carried out on a sample of 100 women and on 10 shifts of observation. It is planned to interview 100 women twice within a period of 15 days and also to have two observers in the same room during 10 shifts of observation

A baseline study will take place in all the hospitals to describe the communicative behavior of care-providers. Observation will be based on rooms (labour and delivery) rather than individual residents, throughout the shifts. This means that we are more interested in the service aspect rather than the actual change in the behavior of the resident; but also it means less vulnerability to problems that could arise if some one is under observation. Pre and post (training) measurements will be carried out for hospitals, based on scores collected from those who were observed in observation round, rather than on scores from each resident. It is also planned to repeat the observation two months as well 6 months after the intervention to evaluate waning of the intervention effect. The last part is an additional part of the study and not an actual part of it.

A baseline study will take place to collect data on women's satisfaction prior to the intervention. Then women will be recruited from all hospitals after each time point where the intervention is implemented in a randomly selected a hospital. Thus a total of five measurements of women's satisfaction are a must part of this study. In addition, it is planned to carry out one extra measurement 6 months after the last point of time of intervention. This will also allow an evaluation of the waning of the intervention.

<u>Design:</u>

A stepped wedge randomized design will be used in this study. This design involves sequential roll-out of an intervention to participants in our hospitals over a number of time periods. We embarked on this design for the following reasons: a) we are interested in measuring the impact of training at different points of time, thus to measure the sustainability of the effect or the waning of the intervention's effect. Stepped wedge designs offer a number of opportunities for data analysis, particularly for modeling the effect of time. Such an effect is expected, if "Training" was the intervention. b) Practically, it would be more practical to implement the intervention in stages to groups of doctors rather than having to deliver the intervention simultaneously for all the participants. c) Stepped Wedge designs as reviewed recently by Brown and Lilford (2006) are known for their ethical sensitivity. Although there

For peer review only

is no proof that our suggested intervention will work positively, it is expected that the intervention will do more good than harm, making this design more relevant.

All eligible doctors in the hospitals (clusters) included in this study (public hospitals in Damascus and its surrounding i.e., rural Damascus) will receive the intervention by the end of the study, but the intervention will be phased in over time, and thus the timing of the implementation of the training package in different hospitals will be randomized.

The design is illustrated in the following figure, where shaded cells represent the intervention periods and the blank cells represent the control periods. Timings in which each hospital will receive the intervention will be randomized. Thus hospitals 1, 2, 3, and 4 in this illustration have no real order. Their names will only be known after the randomization is done and communicated. It is expected that two months would be the time periods between intervention implementation and measurements. This estimate is based on the number of doctors in the hospitals as well as the number of women to be recruited each point of time for the outcome measurement.

**Hospital**	**Time**				
	1	2	3	4	5
1					
2					
3					
4					

Sample size

Based on the power calculation graphs presented in Annex 4 and the likelihood that a within-cluster analysis might be employed, we will consider a sample size of 100 per cluster (hospital) at a given time point. Therefore, the total sample size needed for the study is 2000 (= 100 women * 4 hospitals (clusters) * 5 (points of time)) not accounting for non-response rate. If we allow for a 10% non-response rate then the required sample size will be 2223 women. The details of power calculation are shown in annex 4.

For peer review only

Randomization:

The timing in which each hospital will receive the intervention (Stepping) will be randomized. A statistician who is not part of the study team will do the randomization. This will be done in a concealed manner as to prevent any problems from knowing in which hospital training would take place in the next stage.

Blinding

All observers and field workers will be blind to the objectives of the study. The objectives as well as the plan of analysis of the study will not be communicated with the observers or with the field workers.

Statistical Methods

The analysis plan will include the following activities:

a. Preliminary Analysis

A series of tables will be constructed which summarize the basic characteristics of the data collected. Examples of such tables are:

1. Tables that compare baseline characteristics at the individual, cluster level and at different time points will be provided. For continuous variables means and standard errors will be reported. Furthermore, for categorical variables numbers and percentages will be given. In addition, graphical presentations of the outcome variable will be plotted for a visual assessment of any trends within and between clusters.
2. Tables that describe the baseline demographic characteristics of the Physicians will be provided.
3. Tables that compare the scores of the Physicians prior and post training.
4. The intra-cluster correlation coefficient for the main outcome (possibly for other variables) will be reported.

b. Comparing the main outcome between intervention and control groups.

i. At the Hospital –level

For peer review only

Let Y_{ct} denote the mean of cluster *c* at time *t*. Based on equation (1) presented in Annex 4,

$$Y_{ct} = \mu + \alpha_c + \beta_t + I_{ct}\theta + \varepsilon_{ct} \qquad (3)$$

for c in 1, …, C, t in 1, …, T, and ε_{ct} are independently and identically distributed random errors that follow a $N(0, \sigma^2)$, see Hussey and Hughes (2007). Since the size of clusters is similar, then a Linear Mixed Model (LMM) approach will be used to investigate whether there is a treatment effect (H_0: $\theta = 0$). In addition, temporal changes in the response can be investigated by checking whether all the β_t's are significantly different than zero.

ii. **At the Individual-level**

A LMM can be used to estimate the parameters in equation (1) in Annex 4 and study their statistical significance. However, in case the responses are not normally distributed then a Generalized Estimating Equations (GEE), see Zeger and Liang (1986), and a Generalized Linear Mixed Model (GLMM), see McCullagh and Nelder (1989), will be used to estimate the above parameters. In case we decide to categorise the outcome variable, both GEE and GLMM can handle a categorical outcome.

Using GEE will require specifying the working correlation matrix, which will be estimated once the data is available. Other working correlation matrices will also be examined. However, it is known that GEE analysis is robust with respect to the choice of the correlation structure, see Diggle, Heagerty, Liang and Zeger (2002).

c. Adjusting for baseline characteristics and other covariates

Models (1) and (2) can be easily extended to account for baseline characteristics and other covariates including time-varying ones. Similar approaches to those outlined in part b. will be followed. Covariates will include the type of delivery whether normal or C-section.

d. Comparing the physicians' outcome pre and post intervention

Using a regression approach similar to that outlined above. Scores for the physicians will be compared pre and post intervention adjusting for clustering at the hospital level.

e. Software

The statistical analyses will be carried out using both the STATA package software (College Station, Texas) and the R language (www.r-project.org).

For peer review only

f. Missing data

In case of missing data, statistical tests will be carried out to assess whether the missing data are completely missing at random, missing at random, or informative. In addition, the data will be multiply imputed according to one or two available imputation techniques and the results will be compared, see Little and Rubin (2002).

Ethical considerations

The study protocol was submitted to the Institutional Review Board of the Damascus University and it was approved by them. Administrative approval will be taken from all hospital directors. Informed consent will be taken from both residents and all women participating in the study. We think that the choice of the design is relevant in terms of meeting the ethical obligation as to train all care providers. The informed consents are attached (annex 5).

Field work and practical issues

Preparatory work

The team did the following tasks in preparation of the proposal. Those tasks were carried out in 2005-2006.

1- Visited all the hospitals and got their preliminary approval. A formal letter will be sent at the start of the field work. All hospitals' directors received the idea very positively.
2- A small scale qualitative study was carried out to understand the context. A summary of the study is attached (Annex 6).
3- The observation checklist and the satisfaction questionnaire were tested in the field (See attached in Annex 7). Necessary modifications were made as well as time of implementation was calculated.

Data Collection

The study has two main phases; a preparatory one and the main intervention phase.

In the preparatory phase the following will be done:

1- Continue to meet the directors of hospitals to seek their support and explain the study objective and also to get the full description about the organization of the work in labor and

For peer review only

delivery rooms. Routine statistics will be reviewed as for the number of deliveries, number of residents and the job description of each one in terms of childbirth.

2- Prepare for the baseline survey in all hospitals. This survey will use the observation checklists but also will collect background information on the hospital and the workload of the doctors, relationship with colleagues and doctors and perception of the importance of communication skills. This background data will be analyzed by hospital and will not be linked with the observation checklist. Another survey on women satisfaction will be carried out at homes of women who gave birth at the hospitals.

3- Finalize the design of the training package with the help of international experts. The main principal investigator has already got in touch with Dr. Portela from WHO, who has developed a handbook to train care providers in communication skills and counseling as related to maternal and newborn care. The handbook and its accompanying training sessions were tested in developing countries settings. An Arab speaking expert will also be consulted (Dr. Tamar Kabakian from AUB has offered help in developing the training package), and the implementation of the intervention will be carried out by Arab speaking expert (Dr Tarif Bakdash from Syria who is a pediatrician with experience in communication skills teaching).

As for the main intervention phase the following will be done:

1- The randomization as to decide on the timing for the intervention implementation.

2- The implementation of the training. This will be carried out by the Arab speaking expert. The training will take place in three days. The training will be organized according to the number of trainees from doctors by the study team in cooperation with a focal point n each hospital named by the director. All residents in the study hospitals will be exposed to training, not necessary at a single point of time as the number of residents in some hospitals is very big to be trained at one time. The training will cover the following main themes:

- An overview of the doctor-patient communication
- Questioning skills
- Listening skills
- Non-verbal communication
- Answering skills
- Rapport building skills
- Counseling skills
- Skills of persuasion

Effective teaching methods will be used as suggested by Maguire and Pitceathly (2002).

The following steps will be carried out:

For peer review only

1- The Information Phase: Collecting evidence on deficiencies in communication skills (this will be based on the baseline observation)
2- The Sensitization Phase: Sensitizing the participants by using verbal and written examples to elicit their reaction.
3- The Practice Phase: Offering an opportunity to practice the skills under controlled conditions
4- The Feedback phase: Offering constructive feedback on the performance of participants.

3- measurement of outcomes: The observation will be carried out after the training in each hospital as well as 2 and 6 months afterwards. Rounds of observation will be done, where each round will include an observation in all rooms, for 3 days and for all shifts including day and night shifts. As for the women satisfaction questionnaire; this will be implemented as well in 4 points of times excluding the baseline. Nearly one hundred women will be recruited from each hospital. Also, future measurements at 2 and 6 months after the study are considered but will not be part of this study.

Problems anticipated

We are not very worried in this design about the spillover (contamination) effect. The reason for that is indeed the lack of opportunities for doctors from different hospitals to meet as the hospitals are separated by place as well as affiliation; and there is no common activities between them. So there is no need for a washout time between the intervention phases. However, we think that the management of the research will be difficult.

Importance of the study and expected outcomes

This study is important as it is the first to assess the effectiveness of training care providers in interpersonal and communication skills on women's satisfaction with the communicative behavior of doctors attending delivery in Syria. The study will not only provide evidence as to what is the effect of the training but also it will provide an opportunity to train residents in communication skills and of course will raise awareness among all concerned about the importance of this issue as a major part of better quality of care. It is hoped that the dissemination process that will follow this study will also give an opportunity as to communicate the matter with policy makers, academia and professional bodies about the great importance of communication skills in the field of maternity care.

For peer review only

Duration of the study

It is expected that the study will take about 18 months.

References

Bashour H, Abdulsalam A, Al-Faisal W and Cheikha S. The patterns and determinants of maternity care in Damascus, Syria. Eastern Mediterranean Health Journal (in Press).

Beck RS, Daughtridge R, Sloane PD. Physician-patient communication in the primary care office:a systematic review. J Am Board Fam Pract 2002;15(1):25-38.

Bingham D. Helping hospitals change. Part 2: Childbirth education techniques to empower women Journal of Perinatal education2005; 14: 51-55.

Brown CA and Lilford RJ. The stepped wedge trial design: a systematic review. BMC Medical Research Methodology 2006, 6:54.

Department of Health. Changing childbirth. Part 1. Report of the Expert Maternity Group. London: HMSO, 1993.

Department of Health. Changing childbirth. Part 2: Survey of good communication practice in maternity services. London: HMSO, 1993.

Diggle P.J., Heagerty P.J., Liang K.Y., and Zeger S.L. Analysis of longitudinal data. Second edition. Oxford University Press. 2002

Garcia J, Garforth S. Parents and new-born babies in the labour ward. In: Garcia J, Kilpatrick R, Richards M (eds.) The Politics of Maternity Care. Services for Childbearing Women in Twentieth-Century Britain. Oxford: Oxford University Press, 1990: 163-182.

Garcia J, Redshaw M, Fitzsimons B, Keene J. First Class Delivery. A National Survey of Women's Views of Maternity Care. London: Audit Commission, 1998.

Hodnett, E.D., Gates, S., Hofmeyr, G.J., & Sakala, C. Continuous support for women during childbirth (Cochrane Review). The Cochrane Library, 3. Oxford: Update Software, 2003.

Hussey A. and Hughes J.P. Design and Analysis of stepped wedge cluster randomized trials. Contemporary clinical trials 2007; 28:182-191.

Jenkins V, Fallowfield L. Can communication skills training alter physicians' beliefs and behavior in clinics? J Clin Oncol 2002;20(3):765-769.

For peer review only

Kabakian-Khasholian T, Campbell O, Shediac-Rizkallah M, Ghorayeb F. (2000) Women's experiences with maternity care: satisfaction or passivity? Social Science & Medicine, 51:103-113.

Little R.J.A. and Rubin D.B. Statistical Analysis with Missing data. Wiley & Sons. 2002

McCullagh, P. and Nelder J.A. Generalized Linear Models. Second edition. Chapman & Hall. 1989

Maguire P and Pitceathly C. Key communication skills and how to acquire them. BMJ 2002; 325:697-700.

Meakin R and Weinman J. The Medical Interview Satisfaction Scale (MISS-21) adapted for British general practice. Family Practice 2002; 19:257-263.

O' Cathain A, Walters SJ, Nicholl JP, Thomas KJ. Cluster randomized trial of Informed Choice leaflets. In: NHS Centre for Reviews and Dissemination Informed Choice in Maternity Care: an Evaluation of Evidence-Based Leaflets. York: NHS Centre for Reviews and Dissemination, University of York, 2001: 33-44.

Ong LML, de Haes JCJM, Hoos AM, Lammes FB. Doctor-patient communication: a review of the literature. Social Science and Medicine, 1995; 40: 903-918.

Rowe RE, Garcia J, Macfarlane AJ, et al. Improving communication between health professional and women in maternity care: a structured review. Health Expectations 2002; 5: 63-83.

Sethuraman KR. Communication skills in clinical practice. Jaypee 2001: 7.

Sholkamy H, Khalil K, Cherine M, Elnoury A, Breebaart M, Hassanein N. An observation checklist for facility-based normal labor and delivery practices: The Galaa Study. Monographs in Reproductive Health No. 5. Cairo; The Population Council, 2003.

Stewart MA. Effective physician-patient communication and health outcomes: a review. Canadian Medical Association Journal, 1995; 152: 1423-1433.

Zeger S.L. and Liang K.Y. Longitudinal data analysis using generalized linear models. Biometrika 1986; 73, 13-22.

For peer review only

Time frame

The following chart shows the time frame for the proposed study.

	Year 1												Year 2*							
	1	2	3	4	5	6	7	8	9	10	11	12	1	2	3	4	5	6	7	8
Preparatory	X	X																		
Base line survey (women And observation)			X	X																
Training 1					X															
Observation 1						X														
Women Survey1						X		X		X		X								
Training 2							X													
Observation 2								X												
Women Survey						X		X		X		X								
Training 3									X											
Observation 3										X										
Women Survey						X		X		X		X								
Training 4											X									
Observation 4												X								
Women Survey						X		X		X		X								
Data entry													X	X						
Data Analysis															X	X	X			
Writing and dissemination																	X	X		

* possible another measurement for women's satisfaction and observation at 2 and 6 months but not as part of the project.

For peer review only

Estimated Budget

For peer review only

Appendices

1- **Questionnaire for Women's satisfaction with doctor's communication in Maternity hospitals (Arabic and English)**
2- **Observation Checklist (Arabic and English)**
3- ***Power calculation***
4- ***Summary from preparatory qualitative work***
5- ***Informed consents for women and doctors***

For peer review only
